# Supplementary material for: Population Health Management to identify and characterise ongoing health need for high-risk individuals shielded from COVID-19: a cross-sectional cohort study
Source: BMJ Open. 2020 Sep 28;10(9):e041370. doi: 10.1136/bmjopen-2020-041370 (PMC7523155; doi:10.1136/bmjopen-2020-041370)
Supplement: Author's manuscript [file bmjopen-2020-041370.draft_revisions.pdf]

BMJ Open

BMJ Open is committed to open peer review. As part of this commitment we make the peer review history of every article we publish publicly available.

When an article is published we post the peer reviewers' comments and the authors' responses online. We also post the versions of the paper that were used during peer review. These are the versions that the peer review comments apply to.

The versions of the paper that follow are the versions that were submitted during the peer review process. They are not the versions of record or the final published versions. They should not be cited or distributed as the published version of this manuscript.

BMJ Open is an open access journal and the full, final, typeset and author-corrected version of record of the manuscript is available on our site with no access controls, subscription charges or pay-per-view fees (http://bmjopen.bmj.com).

If you have any questions on BMJ Open's open peer review process please email info.bmjopen@bmj.com

Population Health Management to identify and characterise ongoing health need for high-risk individuals shielded from COVID-19: A Cross Sectional Cohort Study

Journal:	*BMJ Open*
Manuscript ID	bmjopen-2020-041370
Article Type:	Original research
Date Submitted by the Author:	05-Jun-2020

Complete List of Authors:	Kenward, Charlie; NHS Bristol North Somerset and South Gloucestershire Clinical Commissioning Group<br>Pratt, Adrian; NHS Bristol North Somerset and South Gloucestershire Clinical Commissioning Group, Modelling and Analytics<br>Creavin, Sam T; University of Bristol, Department of Population Health Sciences<br>Wood, Richard; NHS Bristol North Somerset and South Gloucestershire Clinical Commissioning Group, Modelling and Analytics; University of Bath, School of Management<br>Cooper, Jennifer; University of Bristol, Department of Population Health Sciences; NHS Bristol North Somerset and South Gloucestershire Clinical Commissioning Group, Modelling and Analytics

Keywords:	Public health < INFECTIOUS DISEASES, PRIMARY CARE, HEALTH SERVICES ADMINISTRATION & MANAGEMENT, Risk management < HEALTH SERVICES ADMINISTRATION & MANAGEMENT, EPIDEMIOLOGY, Health informatics < BIOTECHNOLOGY & BIOINFORMATICS

SCHOLARONE™
Manuscripts

I, the Submitting Author has the right to grant and does grant on behalf of all authors of the Work (as defined in the below author licence), an exclusive licence and/or a non-exclusive licence for contributions from authors who are: i) UK Crown employees; ii) where BMJ has agreed a CC-BY licence shall apply, and/or iii) in accordance with the terms applicable for US Federal Government officers or employees acting as part of their official duties; on a worldwide, perpetual, irrevocable, royalty-free basis to BMJ Publishing Group Ltd ("BMJ") its licensees and where the relevant Journal is co-owned by BMJ to the co-owners of the Journal, to publish the Work in this journal and any other BMJ products and to exploit all rights, as set out in our licence.

The Submitting Author accepts and understands that any supply made under these terms is made by BMJ to the Submitting Author unless you are acting as an employee on behalf of your employer or a postgraduate student of an affiliated institution which is paying any applicable article publishing charge ("APC") for Open Access articles. Where the Submitting Author wishes to make the Work available on an Open Access basis (and intends to pay the relevant APC), the terms of reuse of such Open Access shall be governed by a Creative Commons licence – details of these licences and which Creative Commons licence will apply to this Work are set out in our licence referred to above.

Other than as permitted in any relevant BMJ Author's Self Archiving Policies, I confirm this Work has not been accepted for publication elsewhere, is not being considered for publication elsewhere and does not duplicate material already published. I confirm all authors consent to publication of this Work and authorise the granting of this licence.

For peer review only

Population Health Management to identify and characterise ongoing health need for high-risk individuals shielded from COVID-19: A Cross Sectional Cohort Study

Charlie Kenward[1,*], Adrian Pratt[1], Sam Creavin[2], Richard M Wood[1,3], Jennifer A Cooper[1,2]

[1] Bristol, North Somerset and South Gloucestershire CCG; UK National Health Service; Bristol; UK.
[2] Population Health Sciences; Bristol Medical School; University of Bristol; Bristol.
[3] School of Management; University of Bath; Bath; UK.

* Corresponding author. Correspondence to Charlie Kenward; charlie.kenward@nhs.net

For peer review only

Abstract

Objectives: To use Population Health Management (PHM) methods to identify and characterise individuals at high-risk of severe COVID-19 for which shielding is required, for the purposes of managing ongoing health needs and mitigating potential shielding-induced harm.

Design: Individuals at 'high-risk' of COVID-19 were identified using the published national 'Shielded Patient List' criteria. Individual level information including current chronic conditions, historical healthcare utilisation, and demographic and socioeconomic status were used for descriptive analyses of this group using PHM methods. Segmentation used *k*-prototypes cluster analysis.

Setting: A major healthcare system in the South West of England, for which linked primary, secondary, community and mental health data is available in a system-wide dataset. The study was performed at a time considered to be relatively early in the COVID-19 pandemic in the UK.

Participants: 1,013,940 individuals from 78 contributing GP practices.

Results: Compared to the groups considered at 'low' and 'moderate' risk (i.e. eligible for the annual influenza vaccination), individuals at high-risk were older (median 68 years [IQR: 55-77] *c.f.* 30 [18-44] and 63 [38-73], respectively), with more healthcare contacts in the previous year (median 11 [4-26] *c.f.* 0 [0-2] and 2 [0-9]) and had a higher burden of comorbidity (median Charlson score 4 [3-6], *c.f.* 0 [0-0] and 2 [1-4]). Geospatial analyses revealed that 3.3% of rural and semi-rural residents were in the high-risk group compared to 2.91% of urban and inner-city residents (p<0.001). Segmentation uncovered six distinct clusters comprising the high-risk population, with key differentiation based upon age and presence of cancer, respiratory, and mental health conditions.

Conclusions: PHM methods are useful in characterising the needs of individuals requiring shielding. Segmentation of the high-risk population identified groups with distinct characteristics that may benefit from a more tailored response from health and care providers and policy makers.

Keywords: COVID-19, Population Health Management, Cluster Analysis, Delivery of Health Care, severe acute respiratory syndrome coronavirus 2, Unsupervised Machine Learning

Word Count: 4244

Article Summary

Strengths and limitations of this study

- Based upon a linked dataset for one million individuals combining information across primary, secondary, community, and mental health services.
- Criteria used for identifying high-risk individuals were subject to double clinical review and are fully documented to aid reproducibility.
- Results can facilitate health and care interventions that may be tailored for sub-groups within the high-risk population.
- Results reflect the local population where the study was performed, which may limit portability to other settings.

For peer review only

For peer review only - http://bmjopen.bmj.com/site/about/guidelines.xhtml

1. Introduction

Severe acute respiratory syndrome coronavirus 2 (SARS-CoV-2), responsible for the disease known as COVID-19, was declared a pandemic by the World Health Organisation on 11th March 2020. It has threatened to overwhelm, and in some cases has overwhelmed, health systems regardless of the economic status of the affected nation.[1 2] SARS-CoV-2 has the ability to spread quickly, partly owing to infected individuals being contagious during the early and asymptomatic phase.[3-5] While COVID-19 is a mild or flu-like illness in most people, for an estimated 14% the disease can be considered 'severe', with an estimated 6% of cases becoming 'critical' (involving life-threatening pneumonia and respiratory failure).[6] Combined with an estimated case fatality rate of approximately 1.4%,[7] this has led many countries to take drastic policy measures to suppress and contain viral transmission such as lockdown, social distancing and mask wearing, and to shield those at highest risk of severe illness through self-isolation.[8-10]

Faced with early projections that approximately 80% of the population would contract the virus over the course of the epidemic,[8] the UK Government has sought to shield the most vulnerable members of the population. In England, efforts were made to identify and inform an estimated 1.5 million people considered to be high-risk,[11] of the need to self-isolate within their homes for at least 12 weeks. However, this policy comes at the potential cost of shielding-induced harm such as negative mental health effects, reduced exercise, and barriers in accessing health services; which people are likely to require due to the nature of the condition(s) that has led to their shielding. Furthermore, the strategy puts a further strain on local health and care services having to meet the often complex needs of this group outside of normal ways of working. Responding to this challenge requires a detailed understanding of individuals at high-risk, including a characterisation of their health and social needs and the ways in which they normally interact with health and care services.

Population Health Management (PHM) approaches can enable greater proactivity in the response of health and care services to emerging or otherwise unrecognised need, and allow for more personalised and preventative healthcare interventions.[12] As an emerging concept involving an integrated consideration of health determinants, outcomes and interventions,[13] PHM has a substantial presence in the published Long Term Plan for England's National Health Service (NHS).[12] Yet a key challenge to embedding PHM is the ability to source and link record-level data across the health and care divide, with primary care of particular importance given the range of information relating to diagnoses, comorbidities, social status and prescriptions.[14 15] This breadth of data is required to accurately identify the high-risk individuals that require shielding (in a similar manner to its use in previous studies to identify individuals at risk of developing long-term conditions.[14 16] In addition to *identifying* the high-risk group, PHM approaches can also be leveraged to provide a more holistic *understanding* of these individuals in informing an effective response to their needs. As part of a range of descriptive analyses associated with PHM, population segmentation supports this by cutting through the complexity of large and multivariate linked datasets in determining a manageable number of population groups separable by differences in health determinants or outcomes.[17-19] This may be an important tool in understanding the composition of the high-risk group considered here.

The objective of this study was to use Population Health Management (PHM) methods to identify and characterise a high-risk population for which shielding is required, for the purposes of managing ongoing health needs and mitigating potential shielding-induced harm. Set in a large healthcare system in South West England during the early stages of the COVID-19 pandemic, this study makes use of a linked dataset containing healthcare activity and clinical, demographic and social attributes for one million individuals.

For peer review only - http://bmjopen.bmj.com/site/about/guidelines.xhtml

2. Materials and methods

The Reporting of studies Conducted using Observational Routinely-collected Data (RECORD)[20] statement was used as an extension of the STROBE guidelines[21] (Supplementary Material A).

2.1 Data, application and setting

The application of this study was to the Bristol, North Somerset and South Gloucestershire (BNSSG) healthcare system, which is a one million resident health economy across a mixture of urban and rural geographies. Within the system, there are 82 GP practices and three major hospitals. This cross sectional cohort study took place at a time understood to be early in the pandemic, shortly after the non-pharmaceutical interventions (social distancing and isolation strategies) were nationally implemented on 23 March 2020.[22]

The BNSSG system-wide dataset[23] was used to support the analysis contained in this study. This dataset forms part of the PHM infrastructure within the BNSSG healthcare system, and contains information for 1,013,940 registered individuals (from 78 contributing practices) across two tables. The first table consists of individual attributes including presence of clinical conditions, lifestyle factors, and demographic and socioeconomic information. This data is principally derived from GP patient administration systems (all GP practices use EMIS Web). The second table contains information for various patient-related activities such as GP consultations, hospital admissions, mental health appointments, community visits, and prescriptions. This data is sourced across primary and secondary care, mental health and community services and contains information such as specialty and relevant dates and times. A unique individual identifier is used to link the data between the tables. Eligibility criteria included all individuals registered to a contributing practice within BNSSG.

Under General Data Protection Regulation, the Data Controller was BNSSG Clinical Commissioning Group. Data from GP electronic medical records were extracted according to existing data sharing agreements and in line with GDPR and Caldicott principles under pandemic conditions and in accordance to government guidelines and the Control of information (COPI) directive for COVID-19 related analyses.[24]

Patient and public involvement statement

Patients and/or the public were not involved in the design, conduct, reporting or dissemination plans of this research.

2.2 Identifying high-risk individuals

In the UK, construction of the Shielded Patient List (SPL) was led by the Chief Medical Officers (CMOs) of the four home nations, with support from the various medical Royal Colleges and Societies. From the outset, individuals were considered to require shielding if they were a member of one or more of six outlined groups. This included those who have received an organ transplant; those with specific cancers, severe respiratory conditions, and rare diseases that increase the risk of infection; and those who are on immunosuppression therapies or are pregnant with significant congenital heart disease.[25] In developing the specific criteria used to define membership of these groups, a set of central database searches was first published by NHS Digital in March 2020. This accounted for approximately 900,000 of the total 1.5 million people originally estimated to require shielding. In seeking to address this shortfall, the membership criteria has since evolved through additional incorporation of patient lists sent by hospital consultants and GPs, and self-reporting by the public.[11]

Given the manner in which the SPL has incrementally advanced, there are a number of issues affecting its ability to effectively identify the high-risk population for consideration within this study. First, it is exposed to variation in clinical assessment and citizen reporting, i.e. there are elements of

For peer review only - http://bmjopen.bmj.com/site/about/guidelines.xhtml

subjectivity. And second, there is anecdotal evidence emerging from frontline clinicians regarding concerns around over and under reporting of high-risk individuals contained on the SPL, i.e. it is to some degree incomplete. Ultimately, this approach has not resulted in a comprehensive database aligned to the SPL for the purposes of secondary uses as required by healthcare planners.

Consequently, the high-risk group of the BNSSG population considered in this study was identified through the merger of two constituent lists. The first of these is a BNSSG-level subset of the aforementioned SPL originally released by NHS Digital,[11] accounting for approximately 900,000 individuals nationally and 14,388 individuals for the BNSSG population. This was complemented with a list of individuals as identified through a number of local searches of the BNSSG system-wide dataset created in order to match as closely as possible the definitions of the six groups as originally outlined by the CMO (identifying 24,894). Combining these lists gave 29,798 high-risk individuals. Where an exact match in search field was not possible then it was either omitted, or a proxy search term was constructed upon review by two clinicians.[26] To reduce potential misclassification and information bias, high-risk criteria and coding lists were subject to double clinical review. Full criteria are documented in Supplementary Material B.

2.3 Analysing the high-risk group

The high-risk group was firstly analysed through comparison against the remaining population, partitioned to 'low' and 'moderate' risk groups. Moderate risk is defined by eligibility for the annual injectable influenza vaccination[11] and low, or baseline, risk is defined through membership of neither the moderate nor the high-risk groups. Descriptive analyses were performed on the basis of demographics, comorbidities, healthcare utilisation and geographical distribution. Pyramid plots were used to understand the differences in age and sex between individuals of these three groups. The high-risk group was then assessed against the other groups through a comparative analysis examining summary information for setting-level healthcare utilisation, prevalence of individual chronic conditions and non-specific mortality risk (through the Charlson score[27]), in addition to other demographic and socioeconomic variables. Geospatial analyses mapped the proportion of high-risk individuals onto the BNSGG geography at Lower Super Output Area (LSOA) level, in order to identify any differences by rurality or deprivation (as measured by the Index of Multiple Deprivation (IMD) which ranks LSOAs from most to least deprived on a scale from one to ten). There were very few instances of missing data since the data were derived from GP practices and other national data (any variables with missing data accounted <1% of the data). Where required, missing data were handled through a complete cases approach.

The high-risk group was segmented using cluster analysis, which identifies similar groups within a population through the use of statistical methods to maximise the difference between groups according to a given set of features. The features considered here relate to the range of demographic, clinical, and social attributes that could be of interest in gaining a high-level understanding of the clusters, and moreover that would make the clusters 'actionable' in terms of the nature of possible interventions applied to mitigate shielding-induced harm. Given the range of categorical and continuous features considered (Supplementary Material C), the *k*-prototypes clustering method[28] was selected due to its flexibility to accommodate such mixed data types. The number of clusters was selected based on a scree plot of the total within sum of squares versus the number of clusters, the Silhouette index[29] and based on the 'identifability' and 'actionability' of the clusters in a healthcare context[18] (Supplementary Material C). Both clustering and non-clustering variables were assessed for whether they were statistically different from other clusters, an approach used by recent studies to verify results and to indicate how segments differ on each clustering variable.[30 31] Firstly an omnibus statistical test was applied (ANOVA, Kruskal-Wallis, Chi square test) to confirm differences across clusters followed by 15 pairwise tests (t-test, Mann-Whitney U test and z-test for proportions) between all other clusters. A Bonferroni adjustment was made to the significance level of 0.05 due to using multiple test comparisons.

For peer review only - http://bmjopen.bmj.com/site/about/guidelines.xhtml

3. Results

A total of 1,013,940 individuals (50.02% female, median age 37 [IQR: 21-56]) were included within these analyses, with 29,798 (2.9%) identified as 'high-risk', 32.79% as moderate-risk, and the remaining 67.01% as low (baseline) risk. An age and sex breakdown for individuals at these various levels of risk is provided in Figure 1. Figure 1 shows that, according to the definitions used, younger and middle-aged individuals are generally at relatively low-risk, with females of typical reproductive age (20-44 years) at comparatively higher risk compared to males due to the additional risk borne through pregnancy (thus warranting their inclusion for the annual injectable influenza vaccine). In contrast, high-risk individuals are mostly aged between 40 years and 85 years. Figure 2 shows that the long-term conditions used to define the 'high-risk' group (Section 2.2) are more common with increasing age.

Figure 1. Population pyramid showing absolute numbers of the population in 5-year age bands stratified by high-risk (red), moderate risk (orange) and low risk (yellow).

Figure 2. Number of long-term conditions by 5-year age band, with long-term conditions assessed through the Quality and Outcomes Framework (QOF) definitions.

A comparative analysis of the low, moderate and high-risk groups is provided in Table 1 with regard to demographic and socioeconomic information, historical healthcare utilisation, and burden of comorbidity. The high-risk group, as expected through definition, are on average older, have a higher comorbidity rate (Charlson score increases across risk stratas, from a median of 0 [IQR: 0-0] for low-risk to 4 [3-6] for high-risk) and have greater healthcare utilisation for all appointment types. For instance, compared to the group at low-risk, the high-risk group have on average 6 times the number of primary care and community contacts over the preceding calendar year and twice the number compared to the moderate-risk group. In terms of comorbidities, the high-risk group have a much higher proportion of individuals diagnosed with cancer in the past 5 years (24.03%) and with COPD (34.84%) compared to the rest of the cohort. In addition, the proportion of those with cardiovascular conditions (19.59%) in the high-risk group is higher compared to the rest of the cohort (3.48%) and for those who are on medication which requires monitoring (24.13% versus 1.52%). Further comparisons in Supplementary Material D.

For peer review only

Table 1. Comparative analysis of the low (n=679,457), moderate (n=304,685), and high risk (n=29,798) groups with regard to demographic and socioeconomic information, historical healthcare utilisation, and burden of comorbidity.

Variable	Low risk	Moderate risk	High risk
**Demographic and socioeconomic**			
Age (median, IQR)	30 (18-44)	63 (38-73)	68 (55-77)
Female (%)	48.25%	53.82%	51.77%
Deprivation by IMD decile* (median, IQR)	6 (3-8)	6 (4-9)	6 (3-8)
Urban/rural (%)<br>-Rural town and fringe<br>-Rural village and dispersed<br>-Urban city and town	 2.34%<br>3.85%<br>93.81%	 3.13%<br>5.19%<br>91.68%	 2.97%<br>4.81%<br>92.22%
Local authority (%)<br>-Bristol<br>-North Somerset<br>-South Gloucestershire	 52.24%<br>19.79%<br>27.97%	 42.58%<br>26.40%<br>31.02%	 43.74%<br>27.14%<br>29.12%
Has a carer (%)	0.31%	1.46%	2.89%
Housebound (%)	0.04%	1.61%	3.73%
**Healthcare utilisation (calendar year 2019)**			
Primary and community care contacts (median, IQR)<br>*Mean contacts per 1,000 population*	0 (0-2)<br>*1,620*	2 (0-5)<br>*4,753*	5 (2-10)<br>*9,766*
Mental health attendances (median, IQR)<br>*Mean contacts per 1,000 population*	0 (0-0)<br>*226*	0 (0-0)<br>*432*	0 (0-0)<br>*622*
Secondary care elective consultations and admissions (median, IQR)<br>*Mean contacts per 1,000 population*	0 (0-0)<br>*966*	0 (0-4)<br>*2,958*	6 (2-14)<br>*10,756*
Secondary care emergency attendances and admissions (median, IQR)<br>*Mean contacts per 1,000 population*	0 (0-0)<br>*295*	0 (0-0)<br>*527*	0 (0-2)<br>*1,184*
**Comorbidities**			
Cardiovascular condition (%)	0.068%	11.09%	19.59%
Cancer diagnosed** (%)	0.29%	1.85%	24.03%
Mental health condition (%)	9.44%	12.54%	16.96%
Diabetes (%)	0.07%	14.94%	19.95%
Dementia (%)	0.017%	2.31%	2.68%
Asthma (%)	0.82%	17.03%	22.27%
COPD (%)	0.04%	3.05%	34.84%
**Other**			
Drugs that require monitoring (%)	0.64%	3.49%	24.13%
Charlson Score (median, IQR)	0 (0-0)	2 (1-4)	4 (3-6)
Smoker/Non Smoker (%)	12.94%	10.21%	16.21%

** For Index of Multiple Deprivation (IMD), note that 1 is the most deprived decile and 10 is least deprived decile.*
*** Diagnosis in past five years.*

For peer review only - http://bmjopen.bmj.com/site/about/guidelines.xhtml

The concentration of high-risk individuals within the various LSOAs of the BNSSG healthcare system is illustrated in Figure 3. Given that each LSOA contains an average 1,500 individuals, those LSOAs larger in size represent rural geographies while those smaller in size and clustered together represent the greater population densities associated with urban conurbations. The large town of Weston-Super-Mare (population 80,000) appears in the lower area of the figure and the city of Bristol (population 463,000) appears towards the centre on the eastern side. Of the six localities comprising the BNSSG system, the centre and eastern areas of Bristol have the lowest proportion at high-risk (2.29%); and people in this area were younger compared to the other five localities (overall median age 32 years c.f. 38 years; 'high-risk' median age: 64 years c.f. 69 years). The greatest concentration of high-risk individuals (3.88%) is for the locality including Weston-Super-Mare and surrounding areas. The rural areas outside of Bristol account for 55% of the high-risk group (16,255 out of 29,798). Rural areas have a higher proportion of the population at of high-risk (3.33%) compared to urban city areas (2.91%) (P <0.001, test of two proportions).

Figure 3. Geographical map of the Bristol, North Somerset and South Gloucestershire healthcare system illustrating the concentration of high-risk individuals at Lower Super Output Areas (LSOA) level.

Table 2 shows the results of the segmentation analysis of the high-risk group (29,798) into six sub-groups. 224 individuals did not have an LSOA within the BNSSG area but were registered to a BNSSG practice and were removed from the analyses. The top utilisers of the high-risk group were removed (0.4%) leaving a high-risk population of 29,454. Clustering variables differ significantly across the segments as expected since the algorithm inherently creates clusters to differ as much as possible based on the variables. Non-clustering variables were also significantly different across groups. Pairwise comparisons between segments identify which segments differ from others, for instance, for cluster 2 the cardiovascular proportion (44.24%) differs significantly from all other clusters.

Cluster 1 contains only 0.57% of the high-risk group and accounts for those with the highest need in regard of mental health services (79% had a mental health diagnosis). Those with dementia in this cluster had a median age of 78 versus 56 for those without dementia. Of this cluster, 11% were housebound and 6% had a carer yet average Charlson comorbidity index was lower than the high-risk average. Cluster 2 has the greatest age (median 76 years) and most frequent utilisation of primary, secondary and community care. Clinically this segment is characterised by a high prevalence of COPD (58%), cardiovascular disease (44%), diabetes (31%), and dementia (8%). Cluster 3 has the lowest age (median 51 years) and highest asthma prevalence (76%). Cluster 4 has the highest use of elective secondary care appointments with 88% having had a diagnosis of cancer in the past five years. Cluster 5 is characterised by the majority (78%) requiring physiological, biochemical or pharmacological monitoring of prescribed medication. Finally, Cluster 6 is characterised by an 85% COPD prevalence and the second highest smoking prevalence (25%). This cluster is also notable from having lower than average healthcare utilisation across all considered settings.

For peer review only - http://bmjopen.bmj.com/site/about/guidelines.xhtml

Table 2. Segmentation of high-risk group (*n*=29,454), detailing attributes of the six sub-groups as identified through cluster analysis.

Cluster	1. Complex Mental Health (*n*=170)	2. Older Complex (*n*=1372)	3. Younger Asthma (*n*=5327)	4. Recent Cancer (*n*=6612)	5. Drug Monitoring (*n*=6892)	6. Low Utilisation COPD (*n*=9171)	Overall omnibus test
**Clustering variables**							
Age, mean	58.4^{a}	72.7^{b}	48.3^{a}	65.3^{b}	65.1^{b}	71.2^{b}	AN <0.000
Median (IQR)	*58 (50.25-70.00)*	*76 (66.00 – 84.00)*	*51 (34.00 – 64.00)*	*68 (57.00 – 76.00)*	*69 (57.00 – 77.00)*	*72 (64.00 – 80.00)*	
Female, *n* (%)	105 (61.76)	871^{b} (63.48)	3892^{b} (74.32)	2665^{a} (40.31)	4850^{b} (70.37)	2891^{a} (31.52)	X^2 <0.000
Primary and community care contacts, median (IQR)	10^{a} (5-20)	49^{a} (25-83)	4 (2-9)	4 (2-9)	4 (2-8)	4 (2-9)	KW <0.000
Mean contacts per 1,000 population	*16,006*	*61,847*	*6,721*	*6,808*	*6,493*	*6,733*	
Mental health attendances, median (IQR)	45.5^{a} (34-62)	0^{a} (0-0)	0^{c} (0-0)	0^{a} (0-0)	0^{c} (0-0)	0^{c} (0-0)	KW <0.000
Mean contacts per 1,000 population	*49,876*	*612*	*175*	*53*	*155*	*155*	
Secondary care elective consultations and admissions, median (IQR)	6^{c} (2-13)	11^{a} (5-20)	5^{b} (1-11)	16^{a} (8-28)	7^{b} (3-13)	3^{a} (0-7)	KW <0.000
Mean contacts per 1,000 population	*10,288*	*15,136*	*7,860*	*21,332*	*9,020*	*4,818*	
Secondary care emergency attendances and admissions, median (IQR)	2^{a} (0-3)	6^{a} (4-9)	0^{a} (0-2)	0^{a} (0-1)	0^{a} (0-1)	0^{a} (0-1)	KW <0.000
Mean contacts per 1,000 population	*2,365*	*6,820*	*1,089*	*940*	*680*	*786*	
Cardiovascular condition (current), *n* (%)	27 (15.88)	607^{a} (44.24)	435^{b} (8.31)	1018^{c} (15.4)	1093^{c} (15.86)	2569^{b} (28.01)	X^2 <0.000
Cancer diagnosed in the past 5 years, *n* (%)	25^{b} (14.71)	230^{b} (16.76)	358^{a} (6.84)	5813^{a} (87.92)	224^{a} (3.25)	431^{a} (4.7)	X^2 <0.000
Mental Health, *n* (%)	134^{a} (78.82)	308^{b} (22.45)	1181^{b} (22.55)	766^{a} (11.58)	1067^{b} (15.48)	1516^{b} (16.53)	X^2 <0.000
Diabetes, *n* (%)	39 (22.94)	419^{b} (30.54)	746^{c} (14.24)	1098^{c} (16.61)	1384^{b} (20.08)	2168^{b} (23.64)	X^2 <0.000
Dementia, *n* (%)	18^{b} (10.59)	112^{b} (8.16)	35^{a} (0.67)	91^{a} (1.38)	192^{b} (2.79)	329^{b} (3.59)	X^2 <0.000
Asthma, *n* (%)	54^{b} (31.76)	322^{b} (23.47)	3970^{a} (75.81)	358^{a} (5.41)	507^{a} (7.36)	1358^{a} (14.81)	X^2 <0.000
COPD, *n* (%)	78^{b} (45.88)	800^{b} (58.31)	1053^{a} (20.11)	277^{a} (4.19)	404^{a} (5.86)	7677^{a} (83.71)	X^2 <0.000
Drugs that require monitoring, *n* (%)	67^{a} (39.41)	243^{a} (17.71)	640^{a} (12.22)	351^{b} (5.31)	5396^{a} (78.29)	403^{b} (4.39)	X^2 <0.000
**Non-clustering variables**							
Smoking, *n* (%)<br>-Non smoker<br>-Current smoker	63^{b} (37.06)	211^{b} (15.38)	888^{b} (16.96)	626^{b} (9.47)	679^{b} (9.85)	2317^{b} (25.26)	X^2 <0.000
Urban/rural, *n* (%)<br>-Rural town and fringe (rural)<br>-Rural village and dispersed (rural)<br>-Urban city and town (urban)	6 (3.53)<br>5 (2.94)<br>159 (93.53)	45 (3.28)<br>41 (2.99)<br>1286 (93.73)	125 (2.39)<br>200 (3.82)<br>4912 (93.79)	226 (3.42)<br>408 (6.17)<br>5978 (90.41)	234 (3.40)<br>407 (5.91)<br>6251 (90.70)	237 (2.58)<br>359 (3.91)<br>8575 (93.5)	For Urban versus Rural: X^2 <0.000

For peer review only

IMD decile, median (IQR)	4 (2-7)	5 (3-8)	5 (3-8)	7[a] (4-9)	7[a] (4-9)	5 (2-8)	KW <0.000
Learning disabilities and autism, *n* (%)	7[c] (4.12)	20[b] (1.46)	105[c] (2.00)	28[c] (0.42)	32[c] (0.46)	38[c] (0.41)	X^2 <0.000
Housebound, *n* (%)	19[a] (11.18)	328[a] (23.91)	56[b] (1.07)	104[b] (1.57)	181[a] (2.63)	393[a] (4.29)	X^2 <0.000
Has a carer, *n* (%)	10 (5.88)	134[b] (9.77)	75[c] (1.43)	137 (2.07)	148 (2.15)	341[b] (3.72)	X^2 <0.000
Is a carer, *n* (%)	3 (1.76)	68 (4.96)	162 (3.09)	210 (3.18)	271 (3.93)	343 (3.74)	X^2 0.002
Charlson Score, median (IQR)	3[b] (2-5)	6[a] (4-7)	2[a] (1-4)	5[a] (3-6)	4[b] (2-5)	5[a] (3-6)	KW <0.000

[a] Significantly different from 5 other clusters (all other clusters)
[b] Significantly different from 4 other clusters
[c] Significantly different from 3 other clusters
Bonferroni adjustment to significance level: 0.05/15 = 0.0033 (based on 15 pairwise tests)

For peer review only

4. Discussion

4.1 Summary of main findings

In aggregated routine cross-sectional data from a single healthcare system (1,013,940 population), 2.9% (29,798) of the population met the UK NHS criteria for being at high-risk of severe COVID-19 illness. The proportion of people at high-risk of COVID-19 increased from 2.88% of those aged 50-54 years to 13.21% of those aged 80-84 years, whereas 9.46% of those aged 90-94 years and only 6.79% of those aged 90+ were at 'high-risk'. Compared to those who were not at high-risk of COVID-19, the high-risk group were older, more frequently attended healthcare, and scored highly on the Charlson co-morbidity index. Rural and semi-rural areas, and coastal towns had higher proportions of the population at high-risk of severe COVID-19 illness than urban and inner-city areas. Segmentation of the high-risk population identified six clusters reflecting the distinct characteristics of types of individual that may benefit from a more tailored response from health and care providers and policy makers. At the time of writing, this is the first study in the published literature to evaluate the attributes and needs of a shielded population through the application of PHM approaches.

4.2 Strengths and limitations

A key strength is the large dataset combining individual level data available from the electronic medical record with granular data on healthcare utilisation, demography and geography across a population over one million people. In particular, the dataset includes healthcare utilisation which covers community, primary and secondary care, and mental health services. A further strength is the collaborative co-production of findings drawing on a multi-disciplinary team comprising clinicians, data-scientists and commissioners. This has been possible through the PHM infrastructure embedded at the healthcare system under study. Finally, we demonstrated the potential to develop a comprehensive and accurate search based on this rich, linked data. The criteria used for identifying high-risk individuals were subject to double clinical review and were fully documented to aid reproducibility.

The study had its limitations. Firstly, the findings were derived from a single health and care system which may hinder generalisability. However, the BNSSG population closely matches the age and sex distribution for England using ONS data and PHE data.[32,33] The results may therefore be portable to other English systems. Additionally, the principle that population segmentation can be rapidly used to identify actionable insights and support a commissioning response to a healthcare emergency is broadly applicable. Secondly, the findings rely on routinely coded data, and do not utilise free text in the medical record which may introduce information bias. While reflecting clinical practice, the ascertainment of clinical characteristics (especially diseases) by coded data is likely to both over and under classify people as having disease. Data on medication and prescriptions are likely to be more completely ascertained but

may not reflect medication usage. In contrast, healthcare utilisation data are likely to be most completely ascertained (although difficult to ascertain DNAs and administrative errors). There is also the possibility for differential-coding, i.e. people in certain groups may be less likely to be correctly coded than others. Examples include people who are homeless, experiencing socio-economic disadvantage or language barriers, or those in the travelling community. This may result in differential misclassification especially towards people who are at high-risk of COVID-19 illness.

4.3 Interpretation

The resulting size of the derived BNSSG high-risk population is comparable to an estimated expected number of around 27,500 (1.5 million national high-risk individuals out of 55 million population applied to our cohort). This verifies the search criteria developed to identify these high-risk individuals. Through leveraging more granular data as contained in established PHM infrastructure, this approach both promotes enhanced risk-sensitivity through reducing the risk of over and under reporting (as is possible through coarser central database searches) and enables a comprehensive specification of the search criteria.

Of the six clusters, four were determined by criteria related to the definition of high-risk, i.e. 'younger **asthma'** (n=5,327), 'recent **cancer'** (n=6,612), **drug** monitoring' (n=6,892) and 'low utilisation **COPD'** (n=9,171). Four of the six clusters had a higher proportion of women (Younger Asthma 74%; Drug monitoring 70%; Older complex 63%; Mental health 62%) than men, whereas the COPD cluster and recent cancer had a higher proportion of men (68% and 60%). This reflects known demographics: women are more likely than men to have asthma and autoimmune disease (which often requires drug monitoring), live longer than men, and are more likely to be diagnosed with mental health disorders.[34 35 36 37]. In contrast men are more often diagnosed with cancer and COPD than women.[38] An interesting finding was the small (n=170) but important complex mental health cluster, the result of a high prevalence of comorbidities.

4.4 Implications

Our findings are mostly relevant to healthcare planners because they offer insights at the level of the population. There are three important implications. Firstly, the population at high-risk of COVID-19 reflects a heterogeneous group of people who will require different interventions and response to mitigate shielding-induced risk. Mitigating interventions should be targeted at clusters among other important groups such as those with learning disabilities. For instance, targeted smoking cessation advice by text message for people in the low utilisation COPD group, targeted medication reviews for those in the drug monitoring group; and an individualised, proactive multi-disciplinary care plan for the 0.02% of the population in the complex mental health group.

A second implication of our analysis is that population segmentation can be used to target resources to geographic areas of greatest need, drawing parallels with the long term challenges facing rural and coastal town health highlighted by England's CMO.[39] Policy-makers and clinicians can use these findings to understand how the capacity of healthcare systems reflect the likely demand, for instance ambulance conveyance requirements, primary community and secondary care capacity and target capacity to match demand. A third important implication is that local and national policy makers must explicitly consider the risk of systematic misclassification, even in times of pandemic. The shielding policy is based on at least two assertions: that the criteria as operationalised identify the group at highest risk of serious illness (a classification question), and that shielding reduces the risk of serious illness to an extent that outweighs any harms (a complex-intervention question). The former is supported by a large UK-based study using the OpenSAFELY Platform, identifying independent risk factors for severe COVID-19 disease which was consistent with the criteria used to determine the SPL,[40] although it is notable that older age was the strongest predictor of in-hospital death and was not an explicit factor in the criteria. The extent to which the latter assertion is true is as yet unknown, which is important because there are potential harms to

For peer review only - http://bmjopen.bmj.com/site/about/guidelines.xhtml

individuals from both the misclassification in both directs and to society from misallocation of resources.

Algorithms and artificial intelligence are at risk of exacerbating health inequalities by systematic misclassification.[41] We found that in our population, the proportion classified as high-risk according to national criteria was similar for those aged over 95 years as in those aged 65-69 years. Given that the proportion at high-risk increases monotonically until age 85 there is a concern that this may reflect systematic under-classification of risk in the oldest-old of our population, especially because the proportion at low-risk paradoxically increases from age 85 years onwards. Systematic misclassification could also partly explain the lower proportion of people at 'high-risk' in our deprived populations who may have differential ascertainment of underlying disease and higher risk of transmission due to living arrangements. Our concerns about systematic misclassification are especially relevant given the high excess death rate in care facilities and in people of black and minority ethnic populations.[40 42] Broadening the high-risk criteria would however induce a further significant personal and societal cost; including people with a Charlson score of six and greater would result in 45,000 people classified as high-risk, adding an additional ~15,000 to the current shielded BNSSG population.

4.5 Future work

The two key priorities for further work are to firstly apply these clusters to other geographic settings to determine whether the clusters hold in other healthcare settings and secondly to prospectively determine the actual risk of severe COVID-19 illness in people who are determined to be at high-risk. The high-risk definition was developed at pace and with the evidence and expert opinion available at the time, however the evidence base for risk of severe COVID-19 is still emerging. Potential risk factors that were not included were age, ethnicity, male sex, multi-morbidity or other single comorbidities such as diabetes or heart disease, each of which has been shown to be associated with increased risk of severe disease [7 40 43-46] As we move into a new phase of the pandemic,[47] where we may have to manage further case surges, the development of accurate risk prediction and risk scoring systems are going to be essential to maximising the effectiveness of health and care responses.[48] In addition, research is needed to understand and maximise the classification accuracy of search criteria used for population level datasets, as has been performed in the clinical practice research database.[49]

4.6 Conclusions

PHM methods applied to a system-wide linked dataset are useful in identifying and characterising a population at high-risk of COVID-19 for which shielding is required. Cluster analysis of the high-risk group revealed heterogeneous groups that may benefit from a more tailored response from health and care providers and policy makers.

Acknowledgements
Support in developing and providing the searches of primary care data was provided by Rhys Lewis and Dr Jacob Lee, OneCare. Geographical distribution map of COVID-19 high-risk proportions was produced by Abbie Reynolds-Beer, BNSSG CCG.

Contributors
CK designed the study, constructed the high-risk criteria using the BNSSG system-wide dataset and drafted and revised the paper. JC designed the study, carried out data analysis including cluster analysis modelling, and drafted and revised the paper. RW contributed to study design and data analytics and drafted and revised the paper. SC constructed the high-risk criteria, provided clinical input and interpretation and reviewed the paper. AP contributed to data analysis, construction of the dataset/variables and drafted the manuscript. All authors have approved the final version of the manuscript for publication.

For peer review only

Funding
This study was supported by the NIHR Bristol Biomedical Research Centre at University Hospitals Bristol NHS Foundation Trust at the University of Bristol.

Competing Interests
The authors report no conflicts of interest.

Patient and public involvement
Patients and/or the public were not involved in the design, or conduct, or reporting, or dissemination plans of this research.

Patient consent for publication
Not required.

Ethics Approval
Under General Data Protection Regulation, the Data Controller was BNSSG Clinical Commissioning Group. Data from GP electronic medical records were extracted according to existing data sharing agreements and in line with GDPR and Caldicott principles under pandemic conditions and in accordance to government guidelines and the Control of information (COPI) directive for COVID-19 related analyses.

Data availability statement
Technical appendix and coding lists available in online supplementary files. Local data used for the study are not publicly available.

For peer review only

For peer review only - http://bmjopen.bmj.com/site/about/guidelines.xhtml

References

1. Nacoti M, Ciocca A, Giupponi A, et al. At the Epicenter of the Covid-19 Pandemic and Humanitarian Crises in Italy: Changing Perspectives on Preparation and Mitigation. *Catalyst non-issue content* 2020;1(2) doi: 10.1056/CAT.20.0080
2. Stefanini GG, Azzolini E, Condorelli G. Critical Organizational Issues for Cardiologists in the COVID-19 Outbreak: A Frontline Experience From Milan, Italy. *Circulation* 2020 doi: 10.1161/circulationaha.120.047070 [published Online First: 2020/03/26]
3. To KK, Tsang OT, Leung WS, et al. Temporal profiles of viral load in posterior oropharyngeal saliva samples and serum antibody responses during infection by SARS-CoV-2: an observational cohort study. *Lancet Infect Dis* 2020 doi: 10.1016/s1473-3099(20)30196-1 [published Online First: 2020/03/28]
4. Zhang J, Litvinova M, Wang W, et al. Evolving epidemiology and transmission dynamics of coronavirus disease 2019 outside Hubei province, China: a descriptive and modelling study. *Lancet Infect Dis* 2020 doi: 10.1016/s1473-3099(20)30230-9 [published Online First: 2020/04/06]
5. Wei WE, Li Z, Chiew CJ, et al. Presymptomatic Transmission of SARS-CoV-2 — Singapore, January 23–March 16, 2020. Morbidity and Mortality Weekly Report (MMWR), 2020:411-15.
6. WHO-China. Report of the WHO-China Joint Mission on Coronavirus Disease 2019 (COVID-19), 2020:11.
7. Verity R, Okell LC, Dorigatti I, et al. Estimates of the severity of coronavirus disease 2019: a model-based analysis. *The Lancet Infectious Diseases* 2020 doi: 10.1016/S1473-3099(20)30243-7
8. Neil M Ferguson, Daniel Laydon, Gemma Nedjati-Gilani, et al. Impact of non-pharmaceutical interventions (NPIs) to reduce COVID19 mortality and healthcare demand: WHO Collaborating Centre for Infectious Disease Modelling; 2020 [Available from: https://www.imperial.ac.uk/mrc-global-infectious-disease-analysis/news--wuhan-coronavirus/.
9. Tanne JH, Hayasaki E, Zastrow M, et al. Covid-19: how doctors and healthcare systems are tackling coronavirus worldwide. *BMJ* 2020;368:m1090. doi: 10.1136/bmj.m1090
10. Mulheirn I, Alvis S, Insall L, et al. A Sustainable Exit Strategy: Managing Uncertainty, Minimising Harm Tony Blair Institute for Global Change2020 [Available from: https://institute.global/policy/sustainable-exit-strategy-managing-uncertainty-minimising-harm accessed 1st May 2020 2020.
11. NHS Digital. COVID-19 – high risk shielded patient list identification methodology 2020 [Available from: https://digital.nhs.uk/coronavirus/shielded-patient-list/methodology accessed 1st May 2020 2020.
12. NHS England. Population Health and the Population Health Management Programme 2020 [Available from: https://www.england.nhs.uk/integratedcare/building-blocks/phm/.
13. Kindig D, Stoddart G. What is population health? *Am J Public Health* 2003;93(3):380-83. doi: 10.2105/ajph.93.3.380
14. Feldman K, Stiglic G, Dasgupta D, et al. Insights into Population Health Management Through Disease Diagnoses Networks. *Scientific Reports* 2016;6(1):30465. doi: 10.1038/srep30465
15. Asbridge J, Davies M. Clinical care in the home and population health management: improving the care of patients with long-term health conditions. *European Journal for Person Centered Healthcare*;5(1):148-53.
16. Hibbard JH, Greene J, Sacks RM, et al. Improving Population Health Management Strategies: Identifying Patients Who Are More Likely to Be Users of Avoidable Costly Care and Those More Likely to Develop a New Chronic Disease. *Health services research* 2017;52(4):1297-309. doi: 10.1111/1475-6773.12545 [published Online First: 2016/08/22]
17. Yan S, Seng BJJ, Kwan YH, et al. Identifying heterogeneous health profiles of primary care utilizers and their differential healthcare utilization and mortality - a retrospective cohort study. *BMC Fam Pract* 2019;20(1):54. doi: 10.1186/s12875-019-0939-2 [published Online First: 2019/04/25]

For peer review only - http://bmjopen.bmj.com/site/about/guidelines.xhtml

18. Chong JL, Lim KK, Matchar DB. Population segmentation based on healthcare needs: a systematic review. *Syst Rev* 2019;8(1):202. doi: 10.1186/s13643-019-1105-6 [published Online First: 2019/08/15]
19. Wood RM, Murch BJ, Betteridge RC. A comparison of population segmentation methods. *Operations Research for Health Care* 2019
20. Benchimol EI, Smeeth L, Guttmann A, et al. The REporting of studies Conducted using Observational Routinely-collected health Data (RECORD) statement. *PLoS Med* 2015;12(10):e1001885. doi: 10.1371/journal.pmed.1001885 [published Online First: 2015/10/07]
21. von Elm E, Altman DG, Egger M, et al. Strengthening the Reporting of Observational Studies in Epidemiology (STROBE) statement: guidelines for reporting observational studies. *Bmj* 2007;335(7624):806-8. doi: 10.1136/bmj.39335.541782.AD [published Online First: 2007/10/20]
22. Iacobucci G. Covid-19: UK lockdown is "crucial" to saving lives, say doctors and scientists. *BMJ* 2020;368:m1204. doi: 10.1136/bmj.m1204 [published Online First: 2020/03/27]
23. Healthier Together. A system-wide dataset for Bristol, North Somerset, and South Gloucestershire 2020 [Available from: https://bnssghealthiertogether.org.uk/population-health-management/.
24. Department of Health and Social Care. Coronavirus (COVID-19): notification to organisations to share information, 2020.
25. NHS Digital. RETIRED - shielded patient list identification methodology Version 1 2020 [Available from: https://digital.nhs.uk/coronavirus/shielded-patient-list/methodology/shielded-patient-list-methodology-version-1 accessed 1st May 2020 2020.
26. NHS Digital. Coronavirus (COVID-19): Shielded patients list 2020 [Available from: https://digital.nhs.uk/coronavirus/shielded-patient-list.
27. Charlson M, Szatrowski TP, Peterson J, et al. Validation of a combined comorbidity index. *J Clin Epidemiol* 1994;47(11):1245-51. doi: 10.1016/0895-4356(94)90129-5 [published Online First: 1994/11/01]
28. Huang Z. Extensions to the k-Means Algorithm for Clustering Large Data Sets with Categorical Values. *Data Mining and Knowledge Discovery* 1998;2(3):283-304. doi: 10.1023/A:1009769707641
29. Rousseeuw PJ. Silhouettes: A graphical aid to the interpretation and validation of cluster analysis. *Journal of Computational and Applied Mathematics* 1987;20:53-65. doi: https://doi.org/10.1016/0377-0427(87)90125-7
30. Vuik SI, Mayer E, Darzi A. Enhancing risk stratification for use in integrated care: a cluster analysis of high-risk patients in a retrospective cohort study. *BMJ Open* 2016;6(12):e012903. doi: 10.1136/bmjopen-2016-012903
31. Everitt BS, Landau S, Leese M, et al. Cluster analysis. 5th ed. Chichester: John Wiley & Sons 2011.
32. Office for National Statistics. Estimates of the population for the UK, England and Wales, Scotland and Northern Ireland. Mid-2019. 2020 [Available from: https://www.ons.gov.uk/peoplepopulationandcommunity/populationandmigration/populationestimates/datasets/populationestimatesforukenglandandwalesscotlandandnorthernireland accessed 1st June 2020.
33. Public Health England. Public Health Profile: NHS Bristol, North Somerset and South Gloucestershire CCG 2020 [Available from: https://fingertips.phe.org.uk/search/population#page/12/gid/1/pat/46/par/E39000043/ati/165/are/E38000222/cid/4/tbm/1/page-options/ovw-tdo-0 accessed 1st June 2020.
34. Fairweather D, Frisancho-Kiss S, Rose NR. Sex differences in autoimmune disease from a pathological perspective. *Am J Pathol* 2008;173(3):600-9. doi: 10.2353/ajpath.2008.071008 [published Online First: 2008/08/09]
35. Altemus M, Sarvaiya N, Neill Epperson C. Sex differences in anxiety and depression clinical perspectives. *Front Neuroendocrinol* 2014;35(3):320-30. doi: 10.1016/j.yfrne.2014.05.004 [published Online First: 2014/06/03]
36. World Health Organisation. Gender and Mental Health, 2002.

For peer review only - http://bmjopen.bmj.com/site/about/guidelines.xhtml

37. Leynaert B, Sunyer J, Garcia-Esteban R, et al. Gender differences in prevalence, diagnosis and incidence of allergic and non-allergic asthma: a population-based cohort. *Thorax* 2012;67(7):625. doi: 10.1136/thoraxjnl-2011-201249
38. Smittenaar CR, Petersen KA, Stewart K, et al. Cancer incidence and mortality projections in the UK until 2035. *Br J Cancer* 2016;115(9):1147-55. doi: 10.1038/bjc.2016.304 [published Online First: 2016/10/26]
39. Nuffield Trust. Summit 2020 session: Prof Chris Whitty on health trends and projections over the next 20 years 2020 [Available from: https://www.nuffieldtrust.org.uk/media/summit-2020-session-prof-chris-whitty-on-the-direction-of-health-trends-over-the-next-20-years accessed 1st June 2020.
40. Williamson E, Walker AJ, Bhaskaran KJ, et al. OpenSAFELY: factors associated with COVID-19-related hospital death in the linked electronic health records of 17 million adult NHS patients. *medRxiv* 2020:2020.05.06.20092999. doi: 10.1101/2020.05.06.20092999
41. Panch T, Mattie H, Atun R. Artificial intelligence and algorithmic bias: implications for health systems. *J Glob Health* 2019;9(2):010318-18. doi: 10.7189/jogh.09.020318
42. Public Health England. Disparities in the risk and outcomes of COVID-19, 2020.
43. Wang B, Li R, Lu Z, et al. Does comorbidity increase the risk of patients with COVID-19: evidence from meta-analysis. *Aging* 2020;12(7):6049-57. doi: 10.18632/aging.103000
44. Zhou F, Yu T, Du R, et al. Clinical course and risk factors for mortality of adult inpatients with COVID-19 in Wuhan, China: a retrospective cohort study. *Lancet* 2020 doi: 10.1016/s0140-6736(20)30566-3 [published Online First: 2020/03/15]
45. WHO. Coronavirus disease (COVID-2019) situation report—30. 2020
46. European Centre for Disease Prevention and Control S, ,. Novel coronavirus disease 2019 (COVID-19) pandemic: increased transmission in the EU/EEA and the UK – sixth update. 12th March 2020., 2020.
47. NHS England. Second phase of NHS response to COVID-19: Letter from Sir Simon Stevens and Amanda Pritchard 2020 [Available from: https://www.england.nhs.uk/coronavirus/publication/second-phase-of-nhs-response-to-covid-19-letter-from-simon-stevens-and-amanda-pritchard/ accessed 1st June 2020 2020.
48. Wynants L, Van Calster B, Bonten MMJ, et al. Prediction models for diagnosis and prognosis of covid-19 infection: systematic review and critical appraisal. *Bmj* 2020;369:m1328. doi: 10.1136/bmj.m1328 [published Online First: 2020/04/09]
49. Quint JK, Müllerova H, DiSantostefano RL, et al. Validation of chronic obstructive pulmonary disease recording in the Clinical Practice Research Datalink (CPRD-GOLD). *BMJ Open* 2014;4(7):e005540. doi: 10.1136/bmjopen-2014-005540

For peer review only

For peer review only - http://bmjopen.bmj.com/site/about/guidelines.xhtml

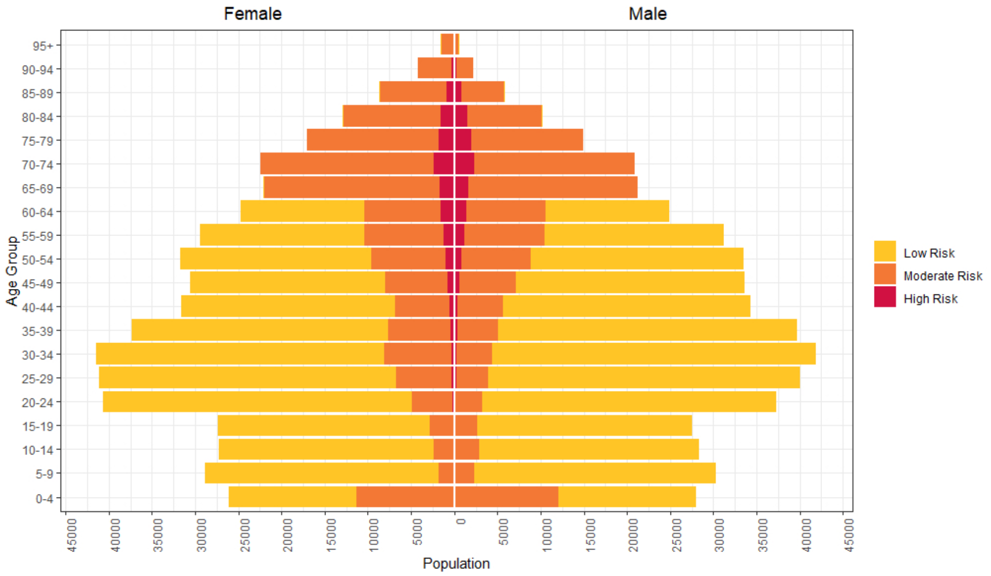

Figure 1. Population pyramid showing absolute numbers of the population in 5-year age bands stratified by high-risk (red), moderate risk (orange) and low risk (yellow).

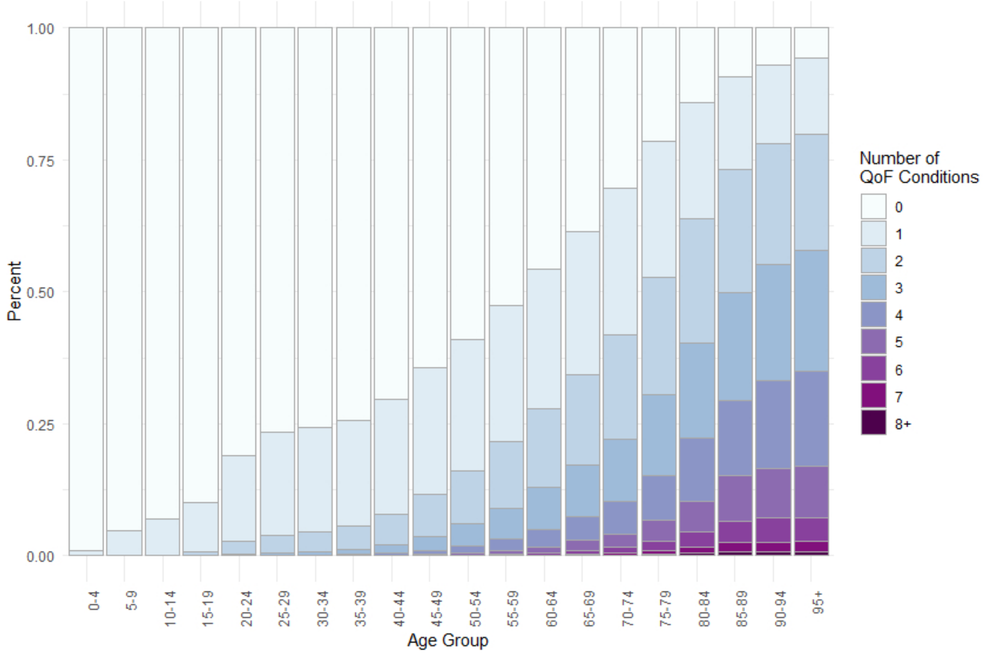

Figure 2. Number of long-term conditions by 5-year age band, with long-term conditions assessed through the Quality and Outcomes Framework (QOF) definitions.

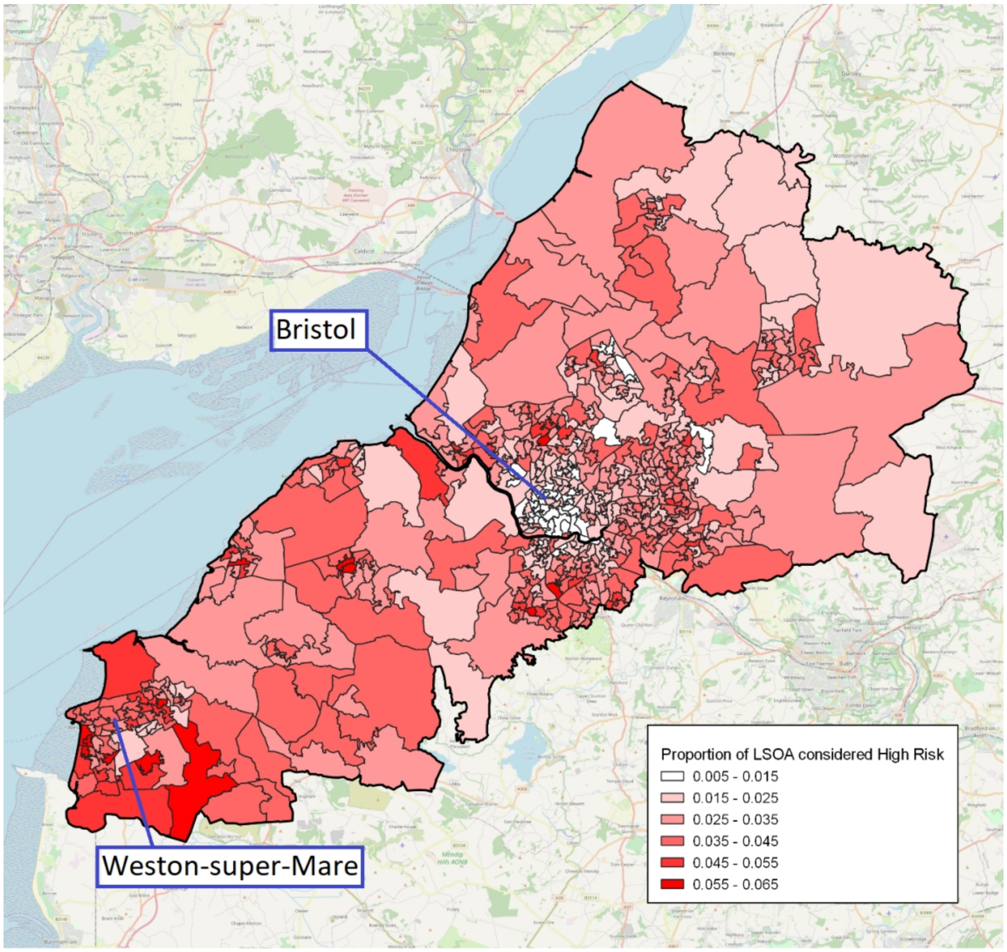

Figure 3. Geographical map of the Bristol, North Somerset and South Gloucestershire healthcare system illustrating the concentration of high-risk individuals at Lower Super Output Areas (LSOA) level.

Supplementary Material A: RECORD Statement

The RECORD statement – checklist of items, extended from the STROBE statement, that should be reported in observational studies using routinely collected health data.

	Item No.	STROBE items	Location in manuscript where items are reported	RECORD items	Location in manuscript where items are reported
**Title and abstract**					
	1	(a) Indicate the study's design with a commonly used term in the title or the abstract (b) Provide in the abstract an informative and balanced summary of what was done and what was found	Page 1 in title and abstract	RECORD 1.1: The type of data used should be specified in the title or abstract. When possible, the name of the databases used should be included.<br><br>RECORD 1.2: If applicable, the geographic region and timeframe within which the study took place should be reported in the title or abstract.<br><br>RECORD 1.3: If linkage between databases was conducted for the study, this should be clearly stated in the title or abstract.	1.1 Page 1 in the abstract.<br><br>1.2 Page 1 in the abstract.<br><br>1.3 Page 1 – data were linked in a system wide dataset.
**Introduction**					
Background rationale	2	Explain the scientific background and rationale for the investigation being reported	Page 3 under Introduction		
Objectives	3	State specific objectives, including any prespecified hypotheses	Page 3 at the bottom of the page.		
**Methods**					
Study Design	4	Present key elements of study design early in the paper	Page 4 under 2.1 Data, application and setting.		
Setting	5	Describe the setting, locations, and relevant dates, including periods of recruitment, exposure, follow-up, and data collection	Page 4		
Participants	6	*(a) Cohort study* - Give the eligibility criteria, and the sources and methods of selection of participants. Describe methods of follow-up<br>*Case-control study* - Give the eligibility criteria, and the sources and methods of case ascertainment and control selection. Give the rationale for the choice of cases and controls<br>*Cross-sectional study* - Give the eligibility criteria, and the sources and methods of selection of participants	Cross-sectional study – page 4 gives eligibility criteria and sources/method of selection.	RECORD 6.1: The methods of study population selection (such as codes or algorithms used to identify subjects) should be listed in detail. If this is not possible, an explanation should be provided.<br><br>RECORD 6.2: Any validation studies of the codes or algorithms used to select the population should be referenced. If validation was conducted for this study and not published elsewhere, detailed methods and results should be provided.<br><br>RECORD 6.3: If the study involved linkage of databases, consider use of a flow diagram or other graphical display to demonstrate the data linkage process, including the number	In this case all individuals within BNSSG registered to a contributing practice were included for analyses. Therefore codes to identify the overall study population were not used.<br><br>For the high-risk group population selection, all codes and criteria used are listed fully in Supplementary Material B and the methods described in section 2.2 (page 4).<br><br>Furthermore, the resulting list was compared with estimated expected numbers of high-risk individuals described in the Discussion under interpretation page 11.<br><br>The system-wide dataset at BNSSG CCG is already linked for analyses but a reference is included providing further

For peer review only - http://bmjopen.bmj.com/site/about/guidelines.xhtml

		(b) Cohort study - For matched studies, give matching criteria and number of exposed and unexposed *Case-control study* - For matched studies, give matching criteria and the number of controls per case		of individuals with linked data at each stage.	information to the dataset (Reference 25) and linking through a pseudonymised ID (unique individual identifier) was mentioned on page 4.
Variables	7	Clearly define all outcomes, exposures, predictors, potential confounders, and effect modifiers. Give diagnostic criteria, if applicable.	All outcomes and variables are fully defined including the use of algorithms and coding lists in Supplementary Material B for the high-risk criteria and Supplementary Material C for all clustering and non-clustering variables. Reference to the Supplementary material included on page 5 of the methods.	RECORD 7.1: A complete list of codes and algorithms used to classify exposures, outcomes, confounders, and effect modifiers should be provided. If these cannot be reported, an explanation should be provided.	All outcomes and variables are fully defined including the use of algorithms and coding lists in Supplementary Material B for the high-risk criteria and Supplementary Material C for all clustering and non-clustering variables.
Data sources/ measurement	8	For each variable of interest, give sources of data and details of methods of assessment (measurement). Describe comparability of assessment methods if there is more than one group	Table C.3 in Supplementary Material C.		
Bias	9	Describe any efforts to address potential sources of bias	To minimise misclassification and information bias, high risk criteria and coding lists were subject to double clinical review (page 5 Methods). The discussion page 10,11 discusses potential information bias as a limitation of using routine datasets.		
Study size	10	Explain how the study size was arrived at	Page 4 and 5		
Quantitative variables	11	Explain how quantitative variables were handled in the analyses. If applicable, describe which groupings were chosen, and why	How the variables were operationalised for analyses is detailed in Table C.3 Supplementary Material C.		
Statistical methods	12	(a) Describe all statistical methods, including those used to control for confounding (b) Describe any methods used to examine subgroups and interactions (c) Explain how missing data were addressed	a) Section 2.3 page 5. Further details on cluster analysis and selection of the number of clusters is included in Supplementary Material C.<br><br>b) Subgroups were investigated in Table		

For peer review only - http://bmjopen.bmj.com/site/about/guidelines.xhtml

		(d) *Cohort study* - If applicable, explain how loss to follow-up was addressed *Case-control study* - If applicable, explain how matching of cases and controls was addressed *Cross-sectional study* - If applicable, describe analytical methods taking account of sampling strategy (e) Describe any sensitivity analyses	1. Methods described on page 5.<br><br>c) Page 5<br><br>d) NA. The full BNSSG cohort from March 2019 was taken for analyses.<br><br>e) Sensitivity analyses in terms of the clustering solution chosen for segmentation is fully described in Supplementary Material C. Also described more briefly in the methods page 5.		
Data access and cleaning methods		..		RECORD 12.1: Authors should describe the extent to which the investigators had access to the database population used to create the study population.<br><br>RECORD 12.2: Authors should provide information on the data cleaning methods used in the study.	The full BNSSG database population for the SWD were used for analyses. This is included on page 4.<br><br>How variables were coded and operationalised is documented in Supplementary Material B and C and page 4 describes how a unique identifier is used to link data between the tables in the system wide dataset.
Linkage		..		RECORD 12.3: State whether the study included person-level, institutional-level, or other data linkage across two or more databases. The methods of linkage and methods of linkage quality evaluation should be provided.	Page 4 under section 2.1.
**Results**					
Participants	13	(a) Report the numbers of individuals at each stage of the study (*e.g.*, numbers potentially eligible, examined for eligibility, confirmed eligible, included in the study, completing follow-up, and analysed) (b) Give reasons for non-participation at each stage. (c) Consider use of a flow diagram	Page 6 for descriptive analyses. Page 8 for cluster analysis.	RECORD 13.1: Describe in detail the selection of the persons included in the study (*i.e.,* study population selection) including filtering based on data quality, data availability and linkage. The selection of included persons can be described in the text and/or by means of the study flow diagram.	Page 6 for descriptive analyses. Page 8 for cluster analysis.
Descriptive data	14	(a) Give characteristics of study participants (*e.g.*, demographic, clinical, social) and information on exposures and potential confounders (b) Indicate the number of participants with missing data for	Page 6 for descriptive analyses. Page 8 for cluster analysis.<br><br>Number of participants with missing data page 8.		

For peer review only - http://bmjopen.bmj.com/site/about/guidelines.xhtml

		each variable of interest<br>(c) *Cohort study* - summarise follow-up time (*e.g.*, average and total amount)			
Outcome data	15	*Cohort study* - Report numbers of outcome events or summary measures over time<br>*Case-control study* - Report numbers in each exposure category, or summary measures of exposure<br>*Cross-sectional study* - Report numbers of outcome events or summary measures	*Cross-sectional study* – Page 6, Table 1, Figure 1 and 2 report a comparative analysis of the low, moderate and high-risk groups with regard to demographic and socioeconomic information, historical healthcare utilisation, and burden of comorbidity. Figure 3 shows the geographical distribution of high risk individuals.		
Main results	16	(a) Give unadjusted estimates and, if applicable, confounder-adjusted estimates and their precision (e.g., 95% confidence interval). Make clear which confounders were adjusted for and why they were included<br>(b) Report category boundaries when continuous variables were categorized<br>(c) If relevant, consider translating estimates of relative risk into absolute risk for a meaningful time period	a) Main descriptive analyses reported on pages 6-8. Cluster analysis results reported on page 8-10.<br><br>b) Continuous variables kept as continuous and results reported as mean, median with interquartile range or proportion. Tables 1 and 2. Supplementary Material C discusses how variables are operationalised.<br><br>c) Not applicable.		
Other analyses	17	Report other analyses done—e.g., analyses of subgroups and interactions, and sensitivity analyses	Results are presented for low, moderate and high risk subgroups and for the segmentation analysis for clusters 1-6. Pages 6-8 for descriptive analyses and page 8-10 for cluster analyses. Sensitivity analyses in terms of the clustering solution were performed and documented in Supplementary Material C.		
**Discussion**					
Key results	18	Summarise key results with reference to study objectives	Page 10.		
Limitations	19	Discuss limitations of the study, taking into account sources of potential bias or imprecision. Discuss both direction and magnitude of any potential bias	Page 10-11.	RECORD 19.1: Discuss the implications of using data that were not created or collected to answer the specific research question(s). Include discussion of misclassification bias, unmeasured confounding, missing data, and changing eligibility over time, as they	Page 10-12 (i.e. misclassification bias and limitations relating to the data).

For peer review only - http://bmjopen.bmj.com/site/about/guidelines.xhtml

For peer review only

				pertain to the study being reported.	
Interpretation	20	Give a cautious overall interpretation of results considering objectives, limitations, multiplicity of analyses, results from similar studies, and other relevant evidence	Page 11-12.		
Generalisability	21	Discuss the generalisability (external validity) of the study results	Page 10-12.		
**Other Information**					
Funding	22	Give the source of funding and the role of the funders for the present study and, if applicable, for the original study on which the present article is based	Page 13.		
Accessibility of protocol, raw data, and programming code		..		RECORD 22.1: Authors should provide information on how to access any supplemental information such as the study protocol, raw data, or programming code.	Page 13. Technical appendix and coding lists available in online supplementary files. Local data used for the study are not publicly available.

*Reference: Benchimol EI, Smeeth L, Guttmann A, Harron K, Moher D, Petersen I, Sørensen HT, von Elm E, Langan SM, the RECORD Working Committee. The REporting of studies Conducted using Observational Routinely-collected health Data (RECORD) Statement. *PLoS Medicine* 2015; in press.

*Checklist is protected under Creative Commons Attribution (CC BY) license.

For peer review only - http://bmjopen.bmj.com/site/about/guidelines.xhtml

Supplementary Material B: High Risk Criteria Using BNSSG System Wide Dataset

1. High Risk Criteria Using BNSSG System Wide Dataset (SWD):

Local search terms are based on fields within the SWD. Disease search terms match to fields within the characteristics table of the SWD, which are based on READ code lists. These are either based the QOF business rules, indicated by 'qof_disease', or code sets published by the Cambridge CPRD group, (https://www.phpc.cam.ac.uk/pcu/research/research-groups/crmh/cprd_cam/codelists/) which were then clinically validated locally.

Category	Sub-category	Local search terms	Reference source
**Chronic liver disease, such as hepatitis**	Patients having undergone liver transplantation and taking immunosuppression	No local search, assumed picked up by NHS Digital central search.	https://www.bsg.org.uk/wp-content/uploads/2020/03/Hepatology-Risk-Groups-BSG-BASL-v2.0.pdf<br><br>https://www.bsg.org.uk/covid-19-advice/bsg-basl-advice-on-heptalogy-patient-risk-groups/
	Patients with autoimmune hepatitis taking immunosuppression	(cc_mild_liver_disease OR cc_moderate_or_severe_liver_disease) AND (IMMLIV OR IMMHiRS)	
	Any liver disease AND patients aged ≥70years of age	(cc_mild_liver_disease OR cc_moderate_or_severe_liver_disease) AND age >= 70 years	
	Any liver disease AND pregnancy	No local search, assumed picked up by NHS Digital central search.	
	Any liver disease AND patients with other co-morbidities: cardiac disease, respiratory disease, diabetes mellitus	(cc_mild_liver_disease OR cc_moderate_or_severe_liver_disease) AND (cc_myocardial_infarction OR cc_heart_failure OR qof_chd OR qof_hf OR cc_diabetes_no_complications OR cc_diabetes_end_complications OR qof_diabetes OR cc_ bronchiectasis OR cc_pulmonary_fibrosis OR ((cc_asthma OR qof_asthma) AND asthma products) OR cc_copd OR qof_copd)	
**Rheumatological conditions: Rheumatoid, inflammatory arthritis and other systemic inflammatory conditions.**	Corticosteroid dose of ≥20mg (0.5mg/kg) prednisolone (or equivalent) per day for more than four weeks	(inflam_arthritic OR qof_rheum) AND IMMHiRS	https://www.rheumatology.org.uk/News-Policy/Details/Covid19-Coronavirus-update-members

For peer review only - http://bmjopen.bmj.com/site/about/guidelines.xhtml

	Cyclophosphamide at any dose orally or within last six months IV	Cyclophosphamide >=1 within the last 6 months	
	Corticosteroid dose of ≥5mg prednisolone (or equivalent) per day for more than four weeks plus at least one other immunosuppressive medication*, biologic/monoclonal** or small molecule immunosuppressant (e.g. JAK inhibitors)***	IMMMoRS AND (IMMIMM OR IMMBIO)	
	Any two agents among immunosuppressive medications, biologics/monoclonals** or small molecule immunosuppressants with any co-morbidity****	any two separate drugs within: IMMIMM OR IMMBIO	
**Inflammatory bowel disease (IBD)**	AND a co-morbidity (respiratory, cardiac, hypertension or diabetes mellitus) and/or are ≥70 years old AND one of: Ustekinumab; Vedolizumab; Methotrexate; Anti-TNF alpha monotherapy (infliximab, adalimumab, golimumab); Thiopurines (azathioprine, mercaptopurine, tioguanine); Calcineurin inhibitors (tacrolimus or ciclosporin); Janus kinase (JAK) inhibition (tofacitinib); Combination therapy in stable patients**; Immunosuppressive/biologic trial medication	(age >=70 years OR cc_diabetes_no_complications OR cc_diabetes_end_complications OR qof_diabetes OR qof_ht OR cc_ bronchiectasis OR cc_pulmonary_fibrosis OR cc_copd OR ((qof_asthma OR cc_asthma) AND asthma products) OR qof_copd OR cc_cystic_fibrosis OR cc_myocardial_infarction OR cc_heart_failure OR qof_chd OR qof_hf OR (cc_egfr <30)) AND (IMMIMM OR IMMBIO)	https://www.bsg.org.uk/wp-content/uploads/2020/03/Hepatology-Risk-Groups-BSG-BASL-v2.0.pdf

For peer review only - http://bmjopen.bmj.com/site/about/guidelines.xhtml

	AND who meet one or more of the following criteria: on oral or intravenous prednisolone ≥20 mg per day (only while on this dose); new induction therapy with combo therapy (starting biologic within previous 6 weeks); moderate-to-severely active disease despite immunosuppression/ biologics; short gut syndrome requiring nutritional support; requirement for parenteral nutrition	ibd AND (IMMHiRS OR (first IMMBIO <started within most recent 6 weeks AND (IMMIMM OR IMMMoRS OR IMMHiRS)	
**Solid organ transplant recipients**		No local search, assumed picked up by NHS Digital central search.	https://digital.nhs.uk/coronavirus/shielded-patient-list/methodology
**Cancer**	active chemotherapy	(cancer_lung_year OR cancer_breast_year OR cancer_bowel_year OR cancer_prostate_year OR cancer_cervical_year OR cancer_ovarian_year OR cancer_melanoma_year OR cancer_headneck_year OR cancer_giliver_year OR cancer_other_year OR cancer_bladder_year OR cancer_kidney_year OR cancer_metase_year) AND any of the 'inpatient or outpatient oncology' codes occuring >=3 times in the past year	https://digital.nhs.uk/coronavirus/shielded-patient-list/methodology
	radical radiotherapy for lung cancer	Covered by other categories	
	leukaemia, lymphoma or myeloma who are at any stage of treatment	cancer_leuklymph_year	
	immunotherapy or other continuing antibody treatments for cancer	Covered by other categories	
	other targeted cancer treatments which can affect the immune system, such as protein kinase inhibitors or PARP inhibitors.	Covered by other categories	

For peer review only

	bone marrow or stem cell transplants in the last 6 months, or who are still taking immunosuppression drugs.	('blood and marrow transplantation code' EVER AND (at least one of: azathioprine, Leflunomide, methotrexate, mycophenolate mofetil or mycophenolic acid, ciclosporin, cyclophosphamide, tacrolimus, sirolimus, Rituximab prescribed in the last 6 months)) OR 'blood and marrow transplantation code' in the last 6 months.	
**Severe respiratory conditions**	Severe asthma	((qof_asthma OR cc_asthma) AND asthma product codes)) AND (4 or more prescriptions for prednisolone in last 6 months of 2019) **OR** Omalizumab with >=1 in most revent 6 months)	https://digital.nhs.uk/coronavirus/shielded-patient-list/methodology, plus local meds op advice
	Patients with Chronic Obstructive Pulmonary Disease (COPD) who have required hospitalisation in the last 12 months or patients who have required 2 or more courses of steroids and/or antibiotics in the last 12 months	(LAMA AND LABA AND ICS) OR home_oxygen OR ((qof_copd OR cc_copd) AND (prescribed Roflumilast (<6 months) OR fev1 <50% predicted OR MRC dyspnoea score 4-5))	
	Severe bronchiectasis	cc_bronchiectasis AND (MRC dyspnoea score 5 OR >=2 in-patient respiratory code in the last 12 months OR fev1 <30% predicted OR age >80 years)	https://www.bronchiectasis.eu/severity-assessment, http://www.bronchiectasisseverity.com/15-2/, https://thorax.bmj.com/content/74/Suppl_1/1
	Interstitial lung diseases	lung_restrict	https://www.brit-thoracic.org.uk/media/455101/bts-management-advice-for-ild-patients-v10-23-march-2020.pdf
	Cystic fibrosis	cc_cystic_fibrosis	https://digital.nhs.uk/coronavirus/shielded-patient-list/methodology

For peer review only - http://bmjopen.bmj.com/site/about/guidelines.xhtml

**Raare diseases and inborn errors of metabolism that significantly increase the risk of infections (such as SCID, homozygous sickle cell disease)**	Homozygous sickle cell disease	No local search, assumed picked up by NHS Digital central search.	https://digital.nhs.uk/coronavirus/shielded-patient-list/methodology
**Rare diseases and inborn errors of metabolism that significantly increase the risk of infections (such as SCID, homozygous sickle cell disease)**	Other including SCID	No local search, assumed picked up by NHS Digital central search.	
**People on immunosuppression therapies sufficient to significantly increase risk of infection**		Covered by other categories	https://digital.nhs.uk/coronavirus/shielded-patient-list/methodology
**Pregnant**	AND with significant heart disease, congenital or acquired	No local search, assumed picked up by NHS Digital central search.	https://digital.nhs.uk/coronavirus/shielded-patient-list/methodology

Gastroenterology Notes
No specific recommendations are being made regarding IBD and pregnancy, and pregnant women with IBD are encouraged to follow the guidance available from the UK government for pregnant women in the general population.
* i.e. at least one of (comorbidity listed above or age≥70) plus at least one therapy from the middle column ** Combination therapy may increase risk over monotherapy
These guidelines were formulated by the UK IBD COVID-19 working group on 20/03/2020 and were based on expert opinion and the available evidence at the time. Column headings updated 22/03/2020 to reflect latest government terminology. but there is no specific evidence for this situation
Rheumatology Notes
*Immunosuppressive medications include: Azathioprine, Leflunomide, methotrexate, Mycophenolate (mycophenolate mofetil or mycophenolic acid), ciclosporin, cyclophosphamide, tacrolimus, sirolimus. It does NOT include Hydroxychloroquine or Sulphasalazine either alone or in combination
** Biologic/monoclonal includes: Rituximab within last 12 months; all anti-TNF drugs (etanercept, adalimumab, infliximab, golimumab, certolizumab and biosimilar variants of all of these); Tociluzimab; Abatacept; Belimumab; Anakinra; Seukinumab; Ixekizumab; Ustekinumab; Sarilumumab; canakinumab
*** Small molecules includes: all JAK inhibitors – baracitinib, tofacitinib etc
**** Co-morbidity includes: age >70, Diabetes Mellitus, any pre-existing lung disease, renal impairment, any history of Ischaemic Heart Disease or hypertension NB This advice applies to both adults, children and young people with rheumatic disease. We do NOT advise that patients increase steroid dose if they become unwell
IMMIMM – at least one in the last 6 months
IMMLIV – at least one in the last 6 months
Roflumilast – at least one in the last 6 months

For peer review only - http://bmjopen.bmj.com/site/about/guidelines.xhtml

2. Prescriptions/drug lists to accompany the criteria above

Drug Name	Drug Type	Drug List		Temporal Aspect
**IMMIMM**	Immunosuppressant/immunomodulator	**Immunosuppressant/immunomodulator**	**Drug**	At least one prescription in the last 6 months
		Azathioprine	Thiopurines	
		mercaptopurine	Thiopurines	
		Tioguanine	Thiopurines	
		Leflunomide	Other	
		Thalidomide	Other	
		Methotrexate	Other	
		mycophenolate mofetil	Other	
		mycophenolic acid	Other	
		cyclophosphamide	Other	
		Tacrolimus	Calcineurin inhibitors	
		sirolimus	Calcineurin inhibitors	
		ciclosporin	Calcineurin inhibitors	
**IMMLIV**	Liver Transplant Drugs	Azathioprine<br>Mycophenolate Mofetil<br>Mycophenolic<br>Ciclosporin<br>Sirolimus<br>Tacrolimus		At least one prescription in the last 6 months
**IMMHiRS**	High Risk Steroids	Prednisolone AND (20mg OR 25mg OR 30mg)<br>Dexamethasone AND >=4mg AND tablets<br>Methylprednisolone AND (16mg OR 100mg OR injection)		2 or more in the past 3 months
**IMMMoRS**	Moderate Risk Steroids	Prednisolone AND 5mg<br>Betamethasone AND tablets<br>Deflazacort<br>Dexamethasone AND tablets AND <4mg<br>Hydrocortisone AND tablets and 20mg<br>Methylprednisolone AND (2mg OR 4mg)		2 or more in the past 3 months
**IMMBIO**	Biologic/monoclonal	**Biologic/monoclonal**	**Drug**	Rituximab one or more in the past 12 months<br><br>All other drugs one or more in the past 6 months
		rituximab	Other	
		tociluzimab	Other	
		abatacept	Other	
		belimumab	Other	
		anakinra	Other	
		seukinumab	Other	
		ixekizumab	Other	
		ustekinumab	Other	
		sarilumumab	Other	
		canakinumab	Other	
		etanercept	Anti-TNF	
		adalimumab	Anti-TNF	
		infliximab	Anti-TNF	
		golimumab	Anti-TNF	
		certolizumab	Anti-TNF	
		baricitinib	JAK_inhibitors	
		tofacitinib	JAK_inhibitors	
		vedolizumab	Other	
		apremilast	Other	
		brodalumab	Other	
		dupilumab	Other	
		guselkumab	Other	
**IMMBIOAST**	Biologic asthma	Omalizumab		One or more in the past 6 months
**Asthma COPD Products**	Asthma and COPD product name list	Product name list given below along with a label for LAMA, LABA and ICS.		One or more in the past 6 months

For peer review only

For peer review only - http://bmjopen.bmj.com/site/about/guidelines.xhtml

3. Product name list for Asthma and COPD drugs along with the type of medicine (LAMA, LABA or ICS) and their combinations

#	Product Name	Type Label
1	Accolate 20mg tablets (AstraZeneca UK Ltd)	nil
2	Aclidinium brom 396mcg/dose / Formoterol 11.8mcg/dose DP inh	LAMA_LABA
3	Aclidinium bromide 375micrograms/dose dry powder inhaler	LAMA
4	Aclidinium bromide 396micrograms/dose / Formoterol 11.8micrograms/dose dry powder inhaler	LAMA_LABA
5	Aclidinium bromide 396micrograms/dose / Formoterol 11.8micrograms/dose dry powder inhaler (Colorama Pharmaceuticals Ltd)	LAMA_LABA
6	Adrenaline and Atropine comp spray	other
7	Adrenaline and atropine inhalation solution	other
8	Aerivio Spiromax 50microg / 500microg/dose dry pdr inh	ICS_LABA
9	Aerivio Spiromax 50micrograms/dose / 500micrograms/dose dry powder inhaler (Teva UK Ltd)	ICS_LABA
10	AeroBec 100 Autohaler (Meda Pharmaceuticals Ltd)	nil
11	Aerobec 250microgram/actuation Pressurised inhalation (Meda Pharmaceuticals Ltd)	nil
12	AeroBec 50 Autohaler (Meda Pharmaceuticals Ltd)	nil
13	AeroBec Forte 250 Autohaler (Meda Pharmaceuticals Ltd)	nil
14	Aerolin 400 100microgram/actuation Inhalation powder (3M Health Care Ltd)	ICS
15	Aerolin autohaler 100microgram/actuation Pressurised inhalation (3M Health Care Ltd)	ICS
16	AirFluSal 25micrograms/dose / 125micrograms/dose inhaler	ICS_LABA
17	AirFluSal 25micrograms/dose / 250micrograms/dose inhaler	ICS_LABA
18	AirFluSal 25micrograms/dose / 250micrograms/dose inhaler (Sandoz Ltd)	ICS_LABA
19	AirFluSal Forspiro 50microg / 500microg/dose dry pdr inh	ICS_LABA
20	AirFluSal Forspiro 50micrograms/dose / 500micrograms/dose dry powder inhaler (Sandoz Ltd)	ICS_LABA
21	Airomir 100micrograms/dose Autohaler (Teva UK Ltd)	SABA
22	Airomir 100micrograms/dose inhaler (Teva UK Ltd)	SABA
23	AirSalb 100micrograms/dose inhaler CFC free (Sandoz Ltd)	SABA
24	Aloflute 25micrograms/dose / 125micrograms/dose inhaler	ICS_LABA
25	Aloflute 25micrograms/dose / 250micrograms/dose inhaler	ICS_LABA
26	Alupent 750microgram/inhalation Aerosol refill (Boehringer Ingelheim Ltd)	nil
27	Alupent 750microgram/inhalation Inhalation powder (Boehringer Ingelheim Ltd)	nil
28	Alupent expectorant Mixture (Boehringer Ingelheim Ltd)	nil
29	Alupent expectorant Tablet (Boehringer Ingelheim Ltd)	nil
30	Alvesco 160 inhaler	ICS
31	Alvesco 160 inhaler (AstraZeneca UK Ltd)	ICS
32	Alvesco 80 inhaler	ICS
33	Alvesco 80 inhaler (AstraZeneca UK Ltd)	ICS
34	Aminophylline 100mg modified-release tablets	other
35	Aminophylline 200mg tablets	other
36	Aminophylline 225mg Modified-release tablet (Actavis UK Ltd)	other
37	Aminophylline 225mg Modified-release tablet (Hillcross Pharmaceuticals Ltd)	other
38	Aminophylline 250mg/10ml injection	other
39	Aminophylline 250mg/10ml solution for injection ampoules	other
40	Aminophylline 250mg/10ml solution for injection ampoules (A A H Pharmaceuticals Ltd)	other
41	Aminophylline 250mg/10ml solution for injection ampoules (AMCo)	other
42	Aminophylline 250mg/10ml solution for injection ampoules (Martindale Pharmaceuticals Ltd)	other
43	Aminophylline 250mg/ml injection	other

For peer review only - http://bmjopen.bmj.com/site/about/guidelines.xhtml

44	Aminophylline 25mg/ml Injection (Celltech Pharma Europe Ltd)	other
45	Aminophylline 350mg modified-release tablets	other
46	Aminophylline 360mg suppositories	other
47	Aminophylline 360mg suppositories (Special Order)	other
48	Aminophylline hydrate 225mg modified-release tablets	other
49	Aminophylline hydrate 350mg modified-release tablets	other
50	Aminophylline sr 225mg Modified-release tablet (IVAX Pharmaceuticals UK Ltd)	other
51	Anoro Ellipta 55microg/dose / 22microg/dose dry pdr inh	LAMA_LABA
52	Anoro Ellipta 55micrograms/dose / 22micrograms/dose dry powder inhaler (GlaxoSmithKline UK Ltd)	LAMA_LABA
53	Asmabec 100 Clickhaler	ICS
54	Asmabec 100 Clickhaler (Focus Pharmaceuticals Ltd)	ICS
55	Asmabec 100microgram/actuation Spacehaler (Celltech Pharma Europe Ltd)	ICS
56	Asmabec 250 Clickhaler (Focus Pharmaceuticals Ltd)	ICS
57	Asmabec 250microgram/actuation Spacehaler (Celltech Pharma Europe Ltd)	ICS
58	Asmabec 50 Clickhaler (Focus Pharmaceuticals Ltd)	ICS
59	Asmabec 50microgram/actuation Spacehaler (Celltech Pharma Europe Ltd)	ICS
60	Asmanex 200micrograms/dose Twisthaler	ICS
61	Asmanex 200micrograms/dose Twisthaler (Merck Sharp & Dohme Ltd)	ICS
62	Asmanex 400micrograms/dose Twisthaler	ICS
63	Asmanex 400micrograms/dose Twisthaler (Merck Sharp & Dohme Ltd)	ICS
64	Asmasal 100microgram/inhalation Spacehaler (Celltech Pharma Europe Ltd)	nil
65	Asmasal 95micrograms/dose Clickhaler (Focus Pharmaceuticals Ltd)	nil
66	Asmaven 100microgram Inhalation powder (Berk Pharmaceuticals Ltd)	nil
67	Asmavent 100micrograms/dose inhaler CFC free (Kent Pharmaceuticals Ltd)	nil
68	Atimos Modulite 12micrograms/dose inhaler	LABA
69	Atimos Modulite 12micrograms/dose inhaler (Chiesi Ltd)	LABA
70	Atropine methonitrate with papaverine and adrenaline inhalation	other
71	Atrovent 20micrograms/dose Autohaler (Boehringer Ingelheim Ltd)	ipratropium
72	Atrovent 20micrograms/dose inhaler (Boehringer Ingelheim Ltd)	ipratropium
73	Atrovent 20micrograms/dose inhaler CFC free (Boehringer Ingelheim Ltd)	ipratropium
74	Atrovent 20micrograms/dose inhaler CFC free (DE Pharmaceuticals)	ipratropium
75	Atrovent 20micrograms/dose inhaler CFC free (Lexon (UK) Ltd)	ipratropium
76	Atrovent 20micrograms/dose inhaler CFC free (Sigma Pharmaceuticals Plc)	ipratropium
77	Atrovent 250micrograms/1ml nebuliser liquid UDVs (Boehringer Ingelheim Ltd)	ipratropium
78	Atrovent 40microgram Aerocaps (Boehringer Ingelheim Ltd)	ipratropium
79	Atrovent 40microgram Aerocaps with Aerohaler (Boehringer Ingelheim Ltd)	ipratropium
80	Atrovent 500micrograms/2ml nebuliser liquid UDVs (Boehringer Ingelheim Ltd)	ipratropium
81	Atrovent 500micrograms/2ml nebuliser liquid UDVs (Waymade Healthcare Plc)	ipratropium
82	Atrovent aerocaps 40microgram Inhalation powder (Boehringer Ingelheim Ltd)	ipratropium
83	Atrovent aerohaler 40microgram Inhalation powder (Boehringer Ingelheim Ltd)	ipratropium
84	Atrovent Forte 40micrograms/dose inhaler (Boehringer Ingelheim Ltd)	ipratropium
85	Atrovent udv 0.25mg/ml Nebuliser liquid (Boehringer Ingelheim Ltd)	ipratropium
86	Bambec 10mg tablets	LABA
87	Bambec 10mg tablets (AstraZeneca UK Ltd)	LABA
88	Bambec 20mg tablets	LABA
89	Bambec 20mg tablets (AstraZeneca UK Ltd)	LABA

For peer review only

90	Bambuterol 10mg tablets	LABA
91	Bambuterol 20mg tablets	LABA
92	Bdp 100microgram/actuation Spacehaler (Celltech Pharma Europe Ltd)	ICS
93	Bdp 250microgram/actuation Spacehaler (Celltech Pharma Europe Ltd)	ICS
94	Bdp 50microgram/actuation Spacehaler (Celltech Pharma Europe Ltd)	ICS
95	Beclazone 100 Easi-Breathe inhaler (Teva UK Ltd)	ICS
96	Beclazone 100 inhaler	ICS
97	Beclazone 100 inhaler (Teva UK Ltd)	ICS
98	Beclazone 100microgram/actuation Inhalation powder (Actavis UK Ltd)	ICS
99	Beclazone 200 inhaler	ICS
100	Beclazone 200 inhaler (Teva UK Ltd)	ICS
101	Beclazone 250 Easi-Breathe inhaler (Teva UK Ltd)	ICS
102	Beclazone 250 inhaler	ICS
103	Beclazone 250 inhaler (Teva UK Ltd)	ICS
104	Beclazone 250microgram/actuation Inhalation powder (Actavis UK Ltd)	ICS
105	Beclazone 50 Easi-Breathe inhaler	ICS
106	Beclazone 50 Easi-Breathe inhaler (Teva UK Ltd)	ICS
107	Beclazone 50 inhaler (Teva UK Ltd)	ICS
108	Beclazone 50microgram/actuation Inhalation powder (Actavis UK Ltd)	ICS
109	Beclazone easi-breathe (roi) 100microgram/actuation Pressurised inhalation (Ivax Pharmaceuticals Ireland)	ICS
110	Beclazone easi-breathe (roi) 250microgram/actuation Pressurised inhalation (Ivax Pharmaceuticals Ireland)	ICS
111	Becloforte 250micrograms/dose inhaler (Dowelhurst Ltd)	ICS
112	Becloforte 250micrograms/dose inhaler (GlaxoSmithKline UK Ltd)	ICS
113	Becloforte 400microgram disks (GlaxoSmithKline UK Ltd)	ICS
114	Becloforte 400microgram disks with Diskhaler (GlaxoSmithKline UK Ltd)	ICS
115	Becloforte easi-breathe 250microgram/actuation Pressurised inhalation (Allen & Hanburys Ltd)	ICS
116	Becloforte integra 250microgram/actuation Inhaler with compact spacer (Glaxo Laboratories Ltd)	ICS
117	Becloforte vm 250microgram/actuation VM pack (Allen & Hanburys Ltd)	ICS
118	Beclometasone 100 micrograms/actuation vortex inhaler	ICS
119	Beclometasone 100mcg/dose breath actuated inhaler CFC free	ICS
120	Beclometasone 100microg/Formoterol 6microg/dose dry pdr inh	ICS_LABA
121	Beclometasone 100microg/Formoterol 6microg/dose inh CFCfree	ICS_LABA
122	Beclometasone 100microgram inhalation powder blisters	ICS
123	Beclometasone 100microgram inhalation powder blisters with device	ICS
124	Beclometasone 100microgram inhalation powder capsules	ICS
125	Beclometasone 100microgram/actuation Inhalation powder (Actavis UK Ltd)	ICS
126	Beclometasone 100microgram/actuation Inhalation powder (Neo Laboratories Ltd)	ICS
127	Beclometasone 100microgram/actuation Pressurised inhalation (Approved Prescription Services Ltd)	ICS
128	Beclometasone 100micrograms disc	ICS
129	Beclometasone 100micrograms with Salbutamol 200micrograms inhalation capsules	ICS_SABA
130	Beclometasone 100micrograms/actuation extrafine particle cfc free inhaler	ICS
131	Beclometasone 100micrograms/dose / Formoterol 6micrograms/dose dry powder inhaler	ICS_LABA
132	Beclometasone 100micrograms/dose / Formoterol 6micrograms/dose inhaler CFC free	ICS_LABA
133	Beclometasone 100micrograms/dose breath actuated inhaler	ICS
134	Beclometasone 100micrograms/dose breath actuated inhaler CFC free	ICS

For peer review only - http://bmjopen.bmj.com/site/about/guidelines.xhtml

135	Beclometasone 100micrograms/dose dry powder inhaler	ICS
136	Beclometasone 100micrograms/dose inhaler	ICS
137	Beclometasone 100micrograms/dose inhaler (A A H Pharmaceuticals Ltd)	ICS
138	Beclometasone 100micrograms/dose inhaler (Mylan)	ICS
139	Beclometasone 100micrograms/dose inhaler (Teva UK Ltd)	ICS
140	Beclometasone 100micrograms/dose inhaler CFC free	ICS
141	Beclometasone 100micrograms/dose inhaler CFC free (Ennogen Healthcare Ltd)	ICS
142	Beclometasone 200 Cyclocaps (Teva UK Ltd)	ICS
143	Beclometasone 200microg/Formoterol 6microg/dose dry pdr inh	ICS_LABA
144	Beclometasone 200microg/Formoterol 6microg/dose inh CFC free	ICS_LABA
145	Beclometasone 200microgram inhalation powder blisters	ICS
146	Beclometasone 200microgram inhalation powder blisters with device	ICS
147	Beclometasone 200microgram inhalation powder capsules	ICS
148	Beclometasone 200micrograms disc	ICS
149	Beclometasone 200micrograms with Salbutamol 400micrograms inhalation capsules	ICS_SABA
150	Beclometasone 200micrograms/dose / Formoterol 6micrograms/dose dry powder inhaler	ICS_LABA
151	Beclometasone 200micrograms/dose / Formoterol 6micrograms/dose inhaler CFC free	ICS_LABA
152	Beclometasone 200micrograms/dose dry powder inhaler	ICS
153	Beclometasone 200micrograms/dose inhaler	ICS
154	Beclometasone 200micrograms/dose inhaler (A A H Pharmaceuticals Ltd)	ICS
155	Beclometasone 200micrograms/dose inhaler CFC free	ICS
156	Beclometasone 250microgram/actuation Inhalation powder (Actavis UK Ltd)	ICS
157	Beclometasone 250microgram/actuation Inhalation powder (Neo Laboratories Ltd)	ICS
158	Beclometasone 250microgram/actuation Pressurised inhalation (Approved Prescription Services Ltd)	ICS
159	Beclometasone 250micrograms/actuation inhaler and compact spacer	ICS
160	Beclometasone 250micrograms/actuation vortex inhaler	ICS
161	Beclometasone 250micrograms/dose breath actuated inhaler	ICS
162	Beclometasone 250micrograms/dose dry powder inhaler	ICS
163	Beclometasone 250micrograms/dose inhaler	ICS
164	Beclometasone 250micrograms/dose inhaler (A A H Pharmaceuticals Ltd)	ICS
165	Beclometasone 250micrograms/dose inhaler (Mylan)	ICS
166	Beclometasone 250micrograms/dose inhaler (Teva UK Ltd)	ICS
167	Beclometasone 250micrograms/dose inhaler CFC free	ICS
168	Beclometasone 400 Cyclocaps (Teva UK Ltd)	ICS
169	Beclometasone 400microgram disc	ICS
170	Beclometasone 400microgram inhalation powder blisters	ICS
171	Beclometasone 400microgram inhalation powder blisters with device	ICS
172	Beclometasone 400microgram inhalation powder capsules	ICS
173	Beclometasone 400micrograms/actuation inhaler	ICS
174	Beclometasone 400micrograms/dose dry powder inhaler	ICS
175	Beclometasone 50microgram/actuation Inhalation powder (Actavis UK Ltd)	ICS
176	Beclometasone 50microgram/actuation Inhalation powder (Neo Laboratories Ltd)	ICS
177	Beclometasone 50microgram/actuation Pressurised inhalation (Approved Prescription Services Ltd)	ICS
178	Beclometasone 50micrograms with salbutamol 100micrograms/inhalation inhaler	ICS_SABA
179	Beclometasone 50micrograms/actuation extrafine particle cfc free inhaler	ICS
180	Beclometasone 50micrograms/dose breath actuated inh CFC free	ICS

For peer review only - http://bmjopen.bmj.com/site/about/guidelines.xhtml

181	Beclometasone 50micrograms/dose breath actuated inhaler	ICS
182	Beclometasone 50micrograms/dose breath actuated inhaler CFC free	ICS
183	Beclometasone 50micrograms/dose dry powder inhaler	ICS
184	Beclometasone 50micrograms/dose inhaler	ICS
185	Beclometasone 50micrograms/dose inhaler (A A H Pharmaceuticals Ltd)	ICS
186	Beclometasone 50micrograms/dose inhaler (Almus Pharmaceuticals Ltd)	ICS
187	Beclometasone 50micrograms/dose inhaler (Mylan)	ICS
188	Beclometasone 50micrograms/dose inhaler (Teva UK Ltd)	ICS
189	Beclometasone 50micrograms/dose inhaler CFC free	ICS
190	Beclometasone 50micrograms/ml nebuliser suspension	ICS
191	Beclometasons 50 micrograms/actuation vortex inhaler	ICS
192	BECLOMETHASONE /SALBUTAMOL	ICS_SABA
193	Becodisks 100microgram	ICS
194	Becodisks 100microgram (GlaxoSmithKline UK Ltd)	ICS
195	Becodisks 100microgram Disc (Allen & Hanburys Ltd)	ICS
196	Becodisks 100microgram with Diskhaler (GlaxoSmithKline UK Ltd)	ICS
197	Becodisks 200microgram (GlaxoSmithKline UK Ltd)	ICS
198	Becodisks 200microgram (Lexon (UK) Ltd)	ICS
199	Becodisks 200microgram (Mawdsley-Brooks & Company Ltd)	ICS
200	Becodisks 200microgram Disc (Allen & Hanburys Ltd)	ICS
201	Becodisks 200microgram with Diskhaler (GlaxoSmithKline UK Ltd)	ICS
202	Becodisks 400microgram (GlaxoSmithKline UK Ltd)	ICS
203	Becodisks 400microgram (Waymade Healthcare Plc)	ICS
204	Becodisks 400microgram Disc (Allen & Hanburys Ltd)	ICS
205	Becodisks 400microgram with Diskhaler (GlaxoSmithKline UK Ltd)	ICS
206	BECOTIDE 100	ICS
207	Becotide 100 inhaler (GlaxoSmithKline UK Ltd)	ICS
208	Becotide 100 inhaler (Waymade Healthcare Plc)	ICS
209	Becotide 100microgram Rotacaps (GlaxoSmithKline UK Ltd)	ICS
210	Becotide 200 inhaler (GlaxoSmithKline UK Ltd)	ICS
211	Becotide 200microgram Rotacaps (GlaxoSmithKline UK Ltd)	ICS
212	Becotide 400microgram Rotacaps (GlaxoSmithKline UK Ltd)	ICS
213	BECOTIDE 50	ICS
214	Becotide 50 inhaler (Dowelhurst Ltd)	ICS
215	Becotide 50 inhaler (GlaxoSmithKline UK Ltd)	ICS
216	Becotide 50microgram/ml Nebuliser liquid (Allen & Hanburys Ltd)	ICS
217	Becotide easi-breathe 100microgram/actuation Pressurised inhalation (Allen & Hanburys Ltd)	ICS
218	Becotide easi-breathe 50microgram/actuation Pressurised inhalation (Allen & Hanburys Ltd)	ICS
219	BECOTIDE FOR NEBULISER	ICS
220	BECOTIDE ROTACAPS	ICS
221	Becotide Rotahaler (GlaxoSmithKline UK Ltd)	ICS
222	BECOTIDE SUSP FOR NEBULISATION	ICS
223	Berotec 100microgram/actuation Inhalation powder (Boehringer Ingelheim Ltd)	nil
224	Berotec 5mg/ml Nebuliser liquid (Boehringer Ingelheim Ltd)	nil
225	Betamethasone valerate 100micrograms/actuation inhaler	ICS
226	Bextasol Inhalation powder (Allen & Hanburys Ltd)	ICS

For peer review only - http://bmjopen.bmj.com/site/about/guidelines.xhtml

227	Biophylline 125mg/5ml Oral solution (Lorex Synthelabo Ltd)	other
228	Biophylline 350mg Tablet (Lorex Synthelabo Ltd)	other
229	Biophylline 500mg Tablet (Lorex Synthelabo Ltd)	other
230	Braltus 10microgram inhalation pdr caps with Zonda inhaler	LAMA
231	Braltus 10microgram inhalation powder capsules with Zonda inhaler (Teva UK Ltd)	LAMA
232	Brelomax 2mg Tablet (Abbott Laboratories Ltd)	nil
233	Bricanyl 1.5mg/5ml syrup (AstraZeneca UK Ltd)	SABA
234	Bricanyl 2.5mg/5ml solution for injection ampoules (AstraZeneca UK Ltd)	SABA
235	Bricanyl 500microgram/ml Injection (AstraZeneca UK Ltd)	SABA
236	Bricanyl 500micrograms/1ml solution for injection ampoules (AstraZeneca UK Ltd)	SABA
237	Bricanyl 500micrograms/dose Turbohaler (AstraZeneca UK Ltd)	SABA
238	Bricanyl 500micrograms/dose Turbohaler (DE Pharmaceuticals)	SABA
239	Bricanyl 500micrograms/dose Turbohaler (Necessity Supplies Ltd)	SABA
240	Bricanyl 500micrograms/dose Turbohaler (Waymade Healthcare Plc)	SABA
241	Bricanyl 5mg tablets (AstraZeneca UK Ltd)	SABA
242	Bricanyl 5mg/2ml Nebuliser liquid (AstraZeneca UK Ltd)	SABA
243	Bricanyl 5mg/2ml Respules (AstraZeneca UK Ltd)	SABA
244	Bricanyl Oral solution (AstraZeneca UK Ltd)	SABA
245	Bricanyl Refill canister (AstraZeneca UK Ltd)	SABA
246	Bricanyl Tablet (AstraZeneca UK Ltd)	SABA
247	Bricanyl turbohaler 500 500microgram Turbohaler (AstraZeneca UK Ltd)	SABA
248	Bronchilator Aerosol generator (Sanofi-Synthelabo Ltd)	nil
249	Bronchodil 10mg/5ml Oral solution (Viatris Pharmaceuticals Ltd)	nil
250	Bronchodil 20mg Tablet (Viatris Pharmaceuticals Ltd)	nil
251	Bronchodil 500microgram/dose Inhalation powder (Viatris Pharmaceuticals Ltd)	nil
252	Brovon Inhalation vapour (Torbet Laboratories Ltd)	nil
253	Brovon midget Inhalation powder (Torbet Laboratories Ltd)	nil
254	Budelin Novolizer 200micrograms/dose inhalation pdr refill	ICS
255	Budelin Novolizer 200micrograms/dose inhalation powder	ICS
256	Budelin Novolizer 200micrograms/dose inhalation powder (Meda Pharmaceuticals Ltd)	ICS
257	Budelin Novolizer 200micrograms/dose inhalation powder refill (Meda Pharmaceuticals Ltd)	ICS
258	Budesonide 100microg / Formoterol 6microg/dose dry pdr inh	ICS_LABA
259	Budesonide 100micrograms/actuation inhaler	ICS
260	Budesonide 100micrograms/dose / Formoterol 6micrograms/dose dry powder inhaler	ICS_LABA
261	Budesonide 100micrograms/dose dry powder inhaler	ICS
262	Budesonide 1mg/2ml nebuliser liquid unit dose vials	ICS
263	Budesonide 1mg/2ml nebuliser liquid unit dose vials (A A H Pharmaceuticals Ltd)	ICS
264	Budesonide 200mcg/dose / Formoterol 6mcg/dose dry powder inh	ICS_LABA
265	Budesonide 200microg / Formoterol 6microg/dose inh CFC free	ICS_LABA
266	Budesonide 200microg/dose dry pdr inhalation cart with dev	ICS
267	Budesonide 200micrograms/actuation breath actuated powder inhaler	ICS
268	Budesonide 200micrograms/actuation refill canister	ICS
269	Budesonide 200micrograms/dose / Formoterol 6micrograms/dose dry powder inhaler	ICS_LABA
270	Budesonide 200micrograms/dose / Formoterol 6micrograms/dose inhaler CFC free	ICS_LABA
271	Budesonide 200micrograms/dose dry pdr inhalation cartridge	ICS
272	Budesonide 200micrograms/dose dry powder inhalation cartridge	ICS

For peer review only - http://bmjopen.bmj.com/site/about/guidelines.xhtml

273	Budesonide 200micrograms/dose dry powder inhalation cartridge with device	ICS
274	Budesonide 200micrograms/dose dry powder inhaler	ICS
275	Budesonide 200micrograms/dose inhaler	ICS
276	Budesonide 200micrograms/dose inhaler CFC free	ICS
277	Budesonide 400mcg/dose /Formoterol 12mcg/dose dry powder inh	ICS_LABA
278	Budesonide 400micrograms/actuation inhaler	ICS
279	Budesonide 400micrograms/dose / Formoterol 12micrograms/dose dry powder inhaler	ICS_LABA
280	Budesonide 400micrograms/dose dry powder inhaler	ICS
281	Budesonide 400micrograms/dose Turbohaler (Waymade Healthcare Plc)	ICS
282	Budesonide 500micrograms/2ml neb liq unit dose vials	ICS
283	Budesonide 500micrograms/2ml nebuliser liquid unit dose vials	ICS
284	Budesonide 500micrograms/2ml nebuliser liquid unit dose vials (Almus Pharmaceuticals Ltd)	ICS
285	Budesonide 50micrograms/actuation refill canister	ICS
286	Budesonide 50micrograms/dose inhaler	ICS
287	CAM Mixture (Cambridge Healthcare Supplies Ltd)	nil
288	Choledyl 100mg Tablet (Parke-davis Research Laboratories)	nil
289	Choledyl 200mg Tablet (Parke-davis Research Laboratories)	nil
290	Choledyl 62.5mg/5ml Oral solution (Parke-davis Research Laboratories)	nil
291	Choline theophyllinate 100mg tablets	other
292	Choline theophyllinate 200mg tablets	other
293	Choline theophyllinate 62.5mg/5ml oral solution	other
294	Ciclesonide 160micrograms/dose inhaler CFC free	ICS
295	Ciclesonide 80micrograms/dose inhaler CFC free	ICS
296	Clenil Modulite 100micrograms/dose inhaler	ICS
297	Clenil Modulite 100micrograms/dose inhaler (Chiesi Ltd)	ICS
298	Clenil Modulite 100micrograms/dose inhaler (Mawdsley-Brooks & Company Ltd)	ICS
299	Clenil Modulite 200micrograms/dose inhaler	ICS
300	Clenil Modulite 200micrograms/dose inhaler (Chiesi Ltd)	ICS
301	Clenil Modulite 200micrograms/dose inhaler (Mawdsley-Brooks & Company Ltd)	ICS
302	Clenil Modulite 250micrograms/dose inhaler	ICS
303	Clenil Modulite 250micrograms/dose inhaler (Chiesi Ltd)	ICS
304	Clenil Modulite 250micrograms/dose inhaler (Waymade Healthcare Plc)	ICS
305	Clenil Modulite 50micrograms/dose inhaler	ICS
306	Clenil Modulite 50micrograms/dose inhaler (Chiesi Ltd)	ICS
307	Clenil Modulite 50micrograms/dose inhaler (Mawdsley-Brooks & Company Ltd)	ICS
308	Cobutolin 2mg Tablet (Actavis UK Ltd)	nil
309	Combisal 25micrograms/dose / 125micrograms/dose inhaler	ICS_LABA
310	Combisal 25micrograms/dose / 250micrograms/dose inhaler	ICS_LABA
311	Combisal 25micrograms/dose / 50micrograms/dose inhaler	ICS_LABA
312	Combivent nebuliser liquid 2.5ml UDVs (Boehringer Ingelheim Ltd)	other
313	Combivent nebuliser liquid 2.5ml UDVs (DE Pharmaceuticals)	other
314	Combivent nebuliser liquid 2.5ml UDVs (Lexon (UK) Ltd)	other
315	Dexamethasone 100microgram capsules	ICS
316	Dexamethasone 10mg capsules	ICS
317	Dexamethasone 10mg/5ml oral solution	ICS
318	Dexamethasone 10mg/5ml oral solution sugar free	ICS

For peer review only - http://bmjopen.bmj.com/site/about/guidelines.xhtml

319	Dexamethasone 1mg/5ml oral solution	ICS
320	Dexamethasone 20mg/5ml oral solution sugar free	ICS
321	Dexamethasone 2mg/5ml oral solution	ICS
322	Dexamethasone 2mg/5ml oral solution sugar free	ICS
323	Dexamethasone 2mg/5ml oral solution sugar free (A A H Pharmaceuticals Ltd)	ICS
324	Dexamethasone 2mg/5ml oral suspension	ICS
325	Dexamethasone 4mg/5ml oral suspension	ICS
326	Dexamethasone 500micrograms/5ml oral solution	ICS
327	Dexamethasone 5mg/5ml oral solution	ICS
328	Dexamethasone 5mg/5ml oral suspension	ICS
329	Dexsol 2mg/5ml oral solution (Rosemont Pharmaceuticals Ltd)	ICS
330	Duaklir 340micrograms/dose / 12micrograms/dose Genuair	LAMA_LABA
331	Duaklir 340micrograms/dose / 12micrograms/dose Genuair (AstraZeneca UK Ltd)	LAMA_LABA
332	Duo-autohaler Inhalation powder (3M Health Care Ltd)	ICS_LABA
333	DuoResp Spiromax 160microg/dose / 4.5microg/dose dry pdr inh	ICS_LABA
334	DuoResp Spiromax 160micrograms/dose / 4.5micrograms/dose dry powder inhaler (Teva UK Ltd)	ICS_LABA
335	DuoResp Spiromax 320microg/dose / 9microg/dose dry pdr inh	ICS_LABA
336	DuoResp Spiromax 320micrograms/dose / 9micrograms/dose dry powder inhaler (Teva UK Ltd)	ICS_LABA
337	Duovent UDVs nebuliser liquid 4ml (Boehringer Ingelheim Ltd)	other
338	Easyhaler Beclometasone 200micrograms/dose dry pdr inhaler	ICS
339	Easyhaler Beclometasone 200micrograms/dose dry powder inhaler (Orion Pharma (UK) Ltd)	ICS
340	Easyhaler Budesonide 100micrograms/dose dry powder inhaler	ICS
341	Easyhaler Budesonide 100micrograms/dose dry powder inhaler (Orion Pharma (UK) Ltd)	ICS
342	Easyhaler Budesonide 200micrograms/dose dry powder inhaler	ICS
343	Easyhaler Budesonide 200micrograms/dose dry powder inhaler (Orion Pharma (UK) Ltd)	ICS
344	Easyhaler Budesonide 400micrograms/dose dry powder inhaler	ICS
345	Easyhaler Budesonide 400micrograms/dose dry powder inhaler (Orion Pharma (UK) Ltd)	ICS
346	Easyhaler Salbutamol sulfate 100micrograms/dose dry powder inhaler (Orion Pharma (UK) Ltd)	SABA
347	Easyhaler Salbutamol sulfate 200micrograms/dose dry powder inhaler (Orion Pharma (UK) Ltd)	SABA
348	Eklira 322micrograms/dose Genuair	LAMA
349	Eklira 322micrograms/dose Genuair (AstraZeneca UK Ltd)	LAMA
350	Eklira 322micrograms/dose Genuair (Waymade Healthcare Plc)	LAMA
351	Ephedrine 15mg tablets	other
352	Ephedrine 15mg/5ml oral solution	other
353	Ephedrine 30mg tablets	other
354	Ephedrine 4mg/5ml oral solution sugar free	other
355	Ephedrine 60mg tablets	other
356	Ephedrine HCl 15mg Tablet (C P Pharmaceuticals Ltd)	other
357	Ephedrine HCl 30mg Tablet (C P Pharmaceuticals Ltd)	other
358	Ephedrine HCl with Chlorphenamine 4mg with 1mg/5ml oral solution sugar free	other
359	Ephedrine hydrochloride 15mg tablets	other
360	Ephedrine hydrochloride 15mg tablets (Wockhardt UK Ltd)	other
361	Ephedrine hydrochloride 30mg tablets	other
362	Ephedrine hydrochloride 30mg tablets (Wockhardt UK Ltd)	other
363	Exirel 10mg Capsule (3M Health Care Ltd)	nil

For peer review only - http://bmjopen.bmj.com/site/about/guidelines.xhtml

364	Exirel 15mg Capsule (3M Health Care Ltd)	nil
365	Exirel 7.5mg/5ml Oral solution (3M Health Care Ltd)	nil
366	Exirel Inhalation powder (3M Health Care Ltd)	nil
367	Expulin decongestant sugar free Oral solution (Shire Pharmaceuticals Ltd)	nil
368	Fenoterol 1.25mg/4ml / Ipratropium 500micrograms/4ml nebuliser liquid unit dose vials	other
369	Fenoterol 100microgram/actuation inhaler	other
370	Filair 100 inhaler (Meda Pharmaceuticals Ltd)	nil
371	Filair 50 inhaler (Meda Pharmaceuticals Ltd)	nil
372	Filair Forte 250micrograms/dose inhaler (Meda Pharmaceuticals Ltd)	nil
373	Flixotide 0.5mg/2ml Nebules	ICS
374	Flixotide 0.5mg/2ml Nebules (GlaxoSmithKline UK Ltd)	ICS
375	Flixotide 0.5mg/2ml Nebules (Waymade Healthcare Plc)	ICS
376	Flixotide 100microgram Disc (Allen & Hanburys Ltd)	ICS
377	Flixotide 100microgram disks (GlaxoSmithKline UK Ltd)	ICS
378	Flixotide 100microgram disks with Diskhaler (GlaxoSmithKline UK Ltd)	ICS
379	Flixotide 100micrograms/dose Accuhaler	ICS
380	Flixotide 100micrograms/dose Accuhaler (GlaxoSmithKline UK Ltd)	ICS
381	Flixotide 100micrograms/dose Accuhaler (Waymade Healthcare Plc)	ICS
382	Flixotide 125microgram/actuation Inhalation powder (Allen & Hanburys Ltd)	ICS
383	Flixotide 125micrograms/dose Evohaler	ICS
384	Flixotide 125micrograms/dose Evohaler (DE Pharmaceuticals)	ICS
385	Flixotide 125micrograms/dose Evohaler (Dowelhurst Ltd)	ICS
386	Flixotide 125micrograms/dose Evohaler (GlaxoSmithKline UK Ltd)	ICS
387	Flixotide 125micrograms/dose Evohaler (Lexon (UK) Ltd)	ICS
388	Flixotide 250microgram Disc (Allen & Hanburys Ltd)	ICS
389	Flixotide 250microgram disks (GlaxoSmithKline UK Ltd)	ICS
390	Flixotide 250microgram disks with Diskhaler (GlaxoSmithKline UK Ltd)	ICS
391	Flixotide 250microgram/actuation Inhalation powder (Allen & Hanburys Ltd)	ICS
392	Flixotide 250micrograms/dose Accuhaler	ICS
393	Flixotide 250micrograms/dose Accuhaler (GlaxoSmithKline UK Ltd)	ICS
394	Flixotide 250micrograms/dose Accuhaler (Stephar (U.K.) Ltd)	ICS
395	Flixotide 250micrograms/dose Accuhaler (Waymade Healthcare Plc)	ICS
396	Flixotide 250micrograms/dose Evohaler	ICS
397	Flixotide 250micrograms/dose Evohaler (GlaxoSmithKline UK Ltd)	ICS
398	Flixotide 250micrograms/dose Evohaler (Waymade Healthcare Plc)	ICS
399	Flixotide 25micrograms/dose inhaler (GlaxoSmithKline UK Ltd)	ICS
400	Flixotide 2mg/2ml Nebules	ICS
401	Flixotide 2mg/2ml Nebules (GlaxoSmithKline UK Ltd)	ICS
402	Flixotide 500microgram Disc (Allen & Hanburys Ltd)	ICS
403	Flixotide 500microgram disks (GlaxoSmithKline UK Ltd)	ICS
404	Flixotide 500microgram disks with Diskhaler (GlaxoSmithKline UK Ltd)	ICS
405	Flixotide 500micrograms/dose Accuhaler	ICS
406	Flixotide 500micrograms/dose Accuhaler (GlaxoSmithKline UK Ltd)	ICS
407	Flixotide 500micrograms/dose Accuhaler (Waymade Healthcare Plc)	ICS
408	Flixotide 50microgram Disc (Allen & Hanburys Ltd)	ICS
409	Flixotide 50microgram disks (GlaxoSmithKline UK Ltd)	ICS

For peer review only - http://bmjopen.bmj.com/site/about/guidelines.xhtml

410	Flixotide 50microgram disks with Diskhaler (GlaxoSmithKline UK Ltd)	ICS
411	Flixotide 50microgram/actuation Inhalation powder (Allen & Hanburys Ltd)	ICS
412	Flixotide 50micrograms/dose Accuhaler	ICS
413	Flixotide 50micrograms/dose Accuhaler (DE Pharmaceuticals)	ICS
414	Flixotide 50micrograms/dose Accuhaler (GlaxoSmithKline UK Ltd)	ICS
415	Flixotide 50micrograms/dose Accuhaler (Mawdsley-Brooks & Company Ltd)	ICS
416	Flixotide 50micrograms/dose Accuhaler (Sigma Pharmaceuticals Plc)	ICS
417	Flixotide 50micrograms/dose Evohaler	ICS
418	Flixotide 50micrograms/dose Evohaler (GlaxoSmithKline UK Ltd)	ICS
419	Flixotide 50micrograms/dose Evohaler (Lexon (UK) Ltd)	ICS
420	Flixotide accuhaler 100 100microgram/inhalation Inhalation powder (Allen & Hanburys Ltd)	ICS
421	Flixotide accuhaler 250 250microgram/inhalation Inhalation powder (Allen & Hanburys Ltd)	ICS
422	Flixotide accuhaler 50 50microgram/inhalation Inhalation powder (Allen & Hanburys Ltd)	ICS
423	Flixotide accuhaler 500 500microgram/inhalation Inhalation powder (Allen & Hanburys Ltd)	ICS
424	Fluticasone 100microgram Disc	ICS
425	Fluticasone 125mcg/dose / Salmeterol 25mcg/dose inh CFC free	ICS_LABA
426	Fluticasone 125microg / Formoterol 5microg/dose inh CFC free	ICS_LABA
427	Fluticasone 125microg/Formoterol 5microg/dose BA inh CFCfree	ICS_LABA
428	Fluticasone 125microgram/actuation Pressurised inhalation	ICS
429	Fluticasone 125micrograms/dose / Formoterol 5micrograms/dose inhaler CFC free	ICS_LABA
430	Fluticasone 125micrograms/dose / Salmeterol 25micrograms/dose inhaler CFC free	ICS_LABA
431	Fluticasone 125micrograms/dose inhaler CFC free	ICS
432	Fluticasone 250mcg/dose / Salmeterol 25mcg/dose inh CFC free	ICS_LABA
433	Fluticasone 250microg / Formoterol 10microg/dose inh CFCfree	ICS_LABA
434	Fluticasone 250microgram Disc	ICS
435	Fluticasone 250microgram/actuation Pressurised inhalation	ICS
436	Fluticasone 250micrograms/dose / Formoterol 10micrograms/dose inhaler CFC free	ICS_LABA
437	Fluticasone 250micrograms/dose / Salmeterol 25micrograms/dose inhaler CFC free	ICS_LABA
438	Fluticasone 250micrograms/dose / Salmeterol 25micrograms/dose inhaler CFC free (A A H Pharmaceuticals Ltd)	ICS_LABA
439	Fluticasone 250micrograms/dose Evohaler (Sigma Pharmaceuticals Plc)	ICS_LABA
440	Fluticasone 250micrograms/dose inhaler CFC free	ICS
441	Fluticasone 25micrograms/dose inhaler	ICS
442	Fluticasone 2mg/2ml nebuliser liquid unit dose vials	ICS
443	Fluticasone 500microgram Disc	ICS
444	Fluticasone 500micrograms/2ml neb liq unit dose vials	ICS
445	Fluticasone 500micrograms/2ml nebuliser liquid unit dose vials	ICS
446	Fluticasone 50microg / Formoterol 5microg/dose inh CFC free	ICS_LABA
447	Fluticasone 50microg / Salmeterol 25microg/dose inh CFC free	ICS_LABA
448	Fluticasone 50microg/Formoterol 5microg/dose BA inh CFC free	ICS_LABA
449	Fluticasone 50microgram Disc	ICS
450	Fluticasone 50microgram/actuation Pressurised inhalation	ICS
451	Fluticasone 50micrograms/dose / Formoterol 5micrograms/dose inhaler CFC free	ICS_LABA
452	Fluticasone 50micrograms/dose / Salmeterol 25micrograms/dose inhaler CFC free	ICS_LABA
453	Fluticasone 50micrograms/dose inhaler CFC free	ICS
454	Fluticasone furoate 184microg/Vilanterol 22microg/dose	ICS_LABA
455	Fluticasone furoate 184micrograms/dose / Vilanterol 22micrograms/dose dry powder inhaler	ICS_LABA

For peer review only - http://bmjopen.bmj.com/site/about/guidelines.xhtml

456	Fluticasone furoate 92microg/Vilanterol 22microg/dose	ICS_LABA
457	Fluticasone furoate 92micrograms/dose / Vilanterol 22micrograms/dose dry powder inhaler	ICS_LABA
458	Fluticasone propion 500mcg/Salmeterol 50mcg/dose dry pdr inh	ICS_LABA
459	Fluticasone propionate 100microg/Salmeterol 50microg/dose	ICS_LABA
460	Fluticasone propionate 100microgram inhalation powder blisters	ICS
461	Fluticasone propionate 100microgram inhalation powder blisters with device	ICS
462	Fluticasone propionate 100micrograms/dose / Salmeterol 50micrograms/dose dry powder inhaler	ICS_LABA
463	Fluticasone propionate 100micrograms/dose dry powder inhaler	ICS
464	Fluticasone propionate 250microg/Salmeterol 50microg/dose	ICS_LABA
465	Fluticasone propionate 250microgram inhalation powder blisters	ICS
466	Fluticasone propionate 250microgram inhalation powder blisters with device	ICS
467	Fluticasone propionate 250micrograms/dose / Salmeterol 50micrograms/dose dry powder inhaler	ICS_LABA
468	Fluticasone propionate 250micrograms/dose dry powder inhaler	ICS
469	Fluticasone propionate 500microgram inhalation powder blisters	ICS
470	Fluticasone propionate 500microgram inhalation powder blisters with device	ICS
471	Fluticasone propionate 500micrograms/dose / Salmeterol 50micrograms/dose dry powder inhaler	ICS_LABA
472	Fluticasone propionate 500micrograms/dose / Salmeterol 50micrograms/dose dry powder inhaler (A A H Pharmaceuticals Ltd)	ICS_LABA
473	Fluticasone propionate 500micrograms/dose dry powder inhaler	ICS
474	Fluticasone propionate 50microgram inhalation powder blisters	ICS
475	Fluticasone propionate 50microgram inhalation powder blisters with device	ICS
476	Fluticasone propionate 50micrograms/dose dry powder inhaler	ICS
477	Flutiform 125micrograms/dose / 5micrograms/dose inhaler	ICS_LABA
478	Flutiform 125micrograms/dose / 5micrograms/dose inhaler (Napp Pharmaceuticals Ltd)	ICS_LABA
479	Flutiform 125micrograms/dose / 5micrograms/dose inhaler (Waymade Healthcare Plc)	ICS_LABA
480	Flutiform 250micrograms/dose / 10micrograms/dose inhaler	ICS_LABA
481	Flutiform 250micrograms/dose / 10micrograms/dose inhaler (Napp Pharmaceuticals Ltd)	ICS_LABA
482	Flutiform 250micrograms/dose / 10micrograms/dose inhaler (Waymade Healthcare Plc)	ICS_LABA
483	Flutiform 50micrograms/dose / 5micrograms/dose inhaler	ICS_LABA
484	Flutiform 50micrograms/dose / 5micrograms/dose inhaler (Napp Pharmaceuticals Ltd)	ICS_LABA
485	Flutiform 50micrograms/dose / 5micrograms/dose inhaler (Waymade Healthcare Plc)	ICS_LABA
486	Flutiform K-haler 125microg/dose / 5microg/dose BA inhaler	ICS_LABA
487	Flutiform K-haler 50microg/dose / 5microg/dose BA inh	ICS_LABA
488	Fobumix Easyhaler 160microg / 4.5microg/dose dry pdr inh	ICS_LABA
489	Fobumix Easyhaler 320microg/dose / 9microg/dose dry pdr inh	ICS_LABA
490	Fobumix Easyhaler 80microg/dose / 4.5microg/dose dry pdr inh	ICS_LABA
491	Foradil 12microgram inhalation powder capsules with device	LABA
492	Foradil 12microgram inhalation powder capsules with device (Novartis Pharmaceuticals UK Ltd)	LABA
493	Foradil 12microgram inhalation powder capsules with device (Sigma Pharmaceuticals Plc)	LABA
494	Formoterol 12microgram inhalation pdr caps with device	LABA
495	Formoterol 12microgram inhalation powder capsules with device	LABA
496	Formoterol 12micrograms/dose dry powder inhaler	LABA
497	Formoterol 12micrograms/dose inhaler CFC free	LABA
498	Formoterol 6micrograms/dose dry powder inhaler	LABA
499	Formoterol Easyhaler 12micrograms/dose dry powder inhaler	LABA
500	Formoterol Easyhaler 12micrograms/dose dry powder inhaler (Orion Pharma (UK) Ltd)	LABA

For peer review only - http://bmjopen.bmj.com/site/about/guidelines.xhtml

501	Fostair 100micrograms/dose / 6micrograms/dose inhaler	ICS_LABA
502	Fostair 100micrograms/dose / 6micrograms/dose inhaler (Chiesi Ltd)	ICS_LABA
503	Fostair 200micrograms/dose / 6micrograms/dose inhaler	ICS_LABA
504	Fostair 200micrograms/dose / 6micrograms/dose inhaler (Chiesi Ltd)	ICS_LABA
505	Fostair NEXThaler 100microg/dose / 6microg/dose dry pdr inh	ICS_LABA
506	Fostair NEXThaler 100micrograms/dose / 6micrograms/dose dry powder inhaler (Chiesi Ltd)	ICS_LABA
507	Fostair NEXThaler 200microg/dose / 6microg/dose dry pdr inh	ICS_LABA
508	Fostair NEXThaler 200micrograms/dose / 6micrograms/dose dry powder inhaler (Chiesi Ltd)	ICS_LABA
509	Fusacomb Easyhaler 50microg / 250microg/dose dry pdr inh	ICS_LABA
510	Fusacomb Easyhaler 50microg / 500microg/dose dry pdr inh	ICS_LABA
511	Glycopyrronium bromide 55microg inhalation pdr caps with dev	LAMA
512	Glycopyrronium bromide 55microgram inhalation powder capsules with device	LAMA
513	Glycopyrronium bromide 55microgram inhalation powder capsules with device (J M McGill Ltd)	LAMA
514	Incruse Ellipta 55micrograms/dose dry powder inhaler	LAMA
515	Incruse Ellipta 55micrograms/dose dry powder inhaler (GlaxoSmithKline UK Ltd)	LAMA
516	Indacaterol 150microgram inhalation pdr caps with device	LABA
517	Indacaterol 150microgram inhalation powder capsules with device	LABA
518	Indacaterol 300microgram inhalation pdr caps with device	LABA
519	Indacaterol 300microgram inhalation powder capsules with device	LABA
520	Indacaterol 85microg/Glycopyrronium brom 54microg/dose	LAMA_LABA
521	Indacaterol 85micrograms/dose / Glycopyrronium bromide 54micrograms/dose inhalation powder capsules with device	LAMA_LABA
522	Intal 20mg/2ml nebuliser solution unit dose vials (Aventis Pharma)	nil
523	Intal 5mg/dose inhaler CFC free (Sanofi)	nil
524	Intal autohaler 5 5mg/inhalation Pressurised inhalation (Aventis Pharma)	nil
525	Intal compound Capsule (Rhone-Poulenc Rorer Ltd)	nil
526	Ipramol nebuliser solution 2.5ml Steri-Neb unit dose vials (Teva UK Ltd)	other
527	Ipratropium 250micrograms/1ml nebuliser liquid Steri-Neb unit dose vials (Teva UK Ltd)	ipratropium
528	Ipratropium 250micrograms/1ml nebuliser liquid unit dose Steripoule vials (Galen Ltd)	ipratropium
529	Ipratropium 500micrograms/2ml nebuliser liquid Steri-Neb unit dose vials (Teva UK Ltd)	ipratropium
530	Ipratropium 500micrograms/2ml nebuliser liquid unit dose Steripoule vials (Galen Ltd)	ipratropium
531	Ipratropium bromide 0.25mg/ml	ipratropium
532	Ipratropium bromide 20micrograms/dose breath actuated inhaler	ipratropium
533	Ipratropium bromide 20micrograms/dose inhaler	ipratropium
534	Ipratropium bromide 20micrograms/dose inhaler CFC free	ipratropium
535	Ipratropium bromide 250microgram/ml	ipratropium
536	Ipratropium bromide 250microgram/ml Inhalation vapour (Galen Ltd)	ipratropium
537	Ipratropium bromide 250microgram/ml Nebuliser liquid (Approved Prescription Services Ltd)	ipratropium
538	Ipratropium bromide 250microgram/ml Nebuliser liquid (Galen Ltd)	ipratropium
539	Ipratropium bromide 250microgram/ml Nebuliser liquid (Hillcross Pharmaceuticals Ltd)	ipratropium
540	Ipratropium bromide 250micrograms/1ml nebuliser liquid unit dose vials	ipratropium
541	Ipratropium bromide 250micrograms/ml	ipratropium
542	Ipratropium bromide 40microgram inhalation powder capsules	ipratropium
543	Ipratropium bromide 40microgram inhalation powder capsules with device	ipratropium
544	Ipratropium bromide 40micrograms/dose inhaler	ipratropium
545	Ipratropium bromide 500micrograms/2ml nebuliser liquid unit dose vials	ipratropium

For peer review only - http://bmjopen.bmj.com/site/about/guidelines.xhtml

546	Ipratropium bromide 500micrograms/2ml nebuliser liquid unit dose vials (A A H Pharmaceuticals Ltd)	ipratropium
547	Ipratropium bromide with fenoterol hydrobromide 0micrograms + 100micrograms/actuation	other
548	Ipratropium bromide with fenoterol hydrobromide 40micrograms + 100micrograms/actuation	other
549	Ipratropium bromide with fenoterol hydrobromide 500micrograms + 1.25mg/4ml	other
550	Ipratropium bromide with salbutamol 20mcg + 100mcg	other
551	Ipratropium bromide with salbutamol 500micrograms + 2.5mg/2.5ml	other
552	Iso-autohaler Inhalation powder (3M Health Care Ltd)	other
553	Isoprenaline hc 500micrograms + 50micrograms/metered Pressurised inhalation	other
554	Isoprenaline sulphate 400micrograms/actuation Pressurised inhalation	other
555	Isoprenaline sulphate 80micrograms/actuation Pressurised inhalation	other
556	Isoprenaline sulphate with sodium cromoglicate inhalation Capsule	other
557	Kelhale 100micrograms/dose inhaler	ICS
558	Kelhale 50micrograms/dose inhaler	ICS
559	Lasma 300mg Tablet (Pharmax Ltd)	other
560	Martapan 2mg/5ml oral solution (Martindale Pharmaceuticals Ltd)	Nil
561	Maxivent 100microgram/inhalation Inhalation powder (Ashbourne Pharmaceuticals Ltd)	SABA
562	Maxivent 2.5mg/2.5ml nebuliser liquid unit dose Steripoule vials (Ashbourne Pharmaceuticals Ltd)	SABA
563	Maxivent 5mg/2.5ml nebuliser liquid unit dose Steripoule vials (Ashbourne Pharmaceuticals Ltd)	SABA
564	Medihaler -duo Inhalation powder (3M Health Care Ltd)	SABA
565	Medihaler -iso Aerosol generator (3M Health Care Ltd)	SABA
566	Medihaler -iso forte Inhalation powder (3M Health Care Ltd)	SABA
567	Mometasone 200micrograms/dose dry powder inhaler	ICS
568	Mometasone 400micrograms/dose dry powder inhaler	ICS
569	Monovent 1.5mg/5ml Oral solution (Lagap)	nil
570	Montelukast 10mg tablets	montelukast
571	Montelukast 10mg tablets (A A H Pharmaceuticals Ltd)	montelukast
572	Montelukast 10mg tablets (Accord Healthcare Ltd)	montelukast
573	Montelukast 10mg tablets (Actavis UK Ltd)	montelukast
574	Montelukast 10mg tablets (Alliance Healthcare (Distribution) Ltd)	montelukast
575	Montelukast 10mg tablets (Dr Reddy's Laboratories (UK) Ltd)	montelukast
576	Montelukast 10mg tablets (Milpharm Ltd)	montelukast
577	Montelukast 10mg tablets (Ranbaxy (UK) Ltd)	montelukast
578	Montelukast 10mg tablets (Teva UK Ltd)	montelukast
579	Montelukast 4mg chewable tablets sugar free	montelukast
580	Montelukast 4mg chewable tablets sugar free (Accord Healthcare Ltd)	montelukast
581	Montelukast 4mg chewable tablets sugar free (Actavis UK Ltd)	montelukast
582	Montelukast 4mg chewable tablets sugar free (Alliance Healthcare (Distribution) Ltd)	montelukast
583	Montelukast 4mg granules sachets sugar free	montelukast
584	Montelukast 4mg granules sachets sugar free (A A H Pharmaceuticals Ltd)	montelukast
585	Montelukast 4mg granules sachets sugar free (Teva UK Ltd)	montelukast
586	Montelukast 5mg chewable tablets sugar free	montelukast
587	Montelukast 5mg chewable tablets sugar free (Accord Healthcare Ltd)	montelukast
588	Montelukast 5mg chewable tablets sugar free (Actavis UK Ltd)	montelukast
589	Montelukast 5mg chewable tablets sugar free (Teva UK Ltd)	montelukast
590	Nedocromil 2mg/dose inhaler CFC free	nil

For peer review only

For peer review only - http://bmjopen.bmj.com/site/about/guidelines.xhtml

591	Nedocromil sodium 1% nasal spray	nil
592	Nedocromil sodium 2mg/inhalation inhaler	nil
593	Neovent 25micrograms/dose inhaler CFC free (Kent Pharm)	LABA
594	Neovent 25micrograms/dose inhaler CFC free (Kent Pharmaceuticals Ltd)	LABA
595	Nethaprin dospan Tablet (Marion Merrell Dow Ltd)	nil
596	Noradran 7.5mg+15mg/5ml Oral solution (Norma Chemicals Ltd)	nil
597	Novolizer budesonide 200microgram/actuation Pressurised inhalation (Meda Pharmaceuticals Ltd)	nil
598	Nuelin 60mg/5ml liquid (3M Health Care Ltd)	nil
599	Nuelin SA 175mg tablets (Meda Pharmaceuticals Ltd)	nil
600	Nuelin SA 250 tablets (Meda Pharmaceuticals Ltd)	nil
601	Numotac 10mg Tablet (3M Health Care Ltd)	nil
602	Olodaterol 2.5microg/dose soln for inhalation CFCfree	LABA
603	Olodaterol 2.5micrograms/dose inhalation soln cart CFC free	LABA
604	Olodaterol 2.5micrograms/dose solution for inhalation cartridge with device CFC free	LABA
605	Onbrez Breezhaler 150microgram inhalation pdr caps with dev	LABA
606	Onbrez Breezhaler 150microgram inhalation powder capsules with device (Novartis Pharmaceuticals UK Ltd)	LABA
607	Onbrez Breezhaler 300microgram inhalation pdr caps with dev	LABA
608	Onbrez Breezhaler 300microgram inhalation powder capsules with device (Novartis Pharmaceuticals UK Ltd)	LABA
609	Orciprenaline 0.5mg/ml Injection	other
610	Orciprenaline 750micrograms/inhalation Aerosol refill	other
611	Orciprenaline 750micrograms/inhalation inhaler	other
612	Orciprenaline with bromhexine hcl mixture	other
613	Oxis 12 Turbohaler	LABA
614	Oxis 12 Turbohaler (AstraZeneca UK Ltd)	LABA
615	Oxis 12 Turbohaler (DE Pharmaceuticals)	LABA
616	Oxis 12 Turbohaler (Waymade Healthcare Plc)	LABA
617	Oxis 6 Turbohaler	LABA
618	Oxis 6 Turbohaler (AstraZeneca UK Ltd)	LABA
619	Oxis 6 Turbohaler (Lexon (UK) Ltd)	LABA
620	Pecram 225mg Modified-release tablet (Novartis Consumer Health UK Ltd)	nil
621	Phyllocontin Continus 225mg tablets (Napp Pharmaceuticals Ltd)	other
622	Phyllocontin continus 350mg Tablet (Napp Pharmaceuticals Ltd)	other
623	Phyllocontin Forte Continus 350mg tablets (Napp Pharmaceuticals Ltd)	other
624	Pirbuterol 10mg capsule	SABA
625	Pirbuterol 15mg capsule	SABA
626	Pirbuterol 7.5mg/5ml oral solution	SABA
627	Pirbuterol acetate inhaler	SABA
628	Prednisolone 1mg/ml oral solution (Logixx Pharma Solutions Ltd)	other
629	Prednisolone 5mg/5ml oral solution	other
630	Prednisolone 5mg/5ml oral solution unit dose	other
631	Prednisolone 5mg/5ml oral solution unit dose (A A H Pharmaceuticals Ltd)	other
632	Prednisolone 5mg/5ml oral solution unit dose (Logixx Pharma Solutions Ltd)	other
633	Prednisolone Dompe 5mg/5ml oral solution unit dose (Logixx Pharma Solutions Ltd)	other
634	Pro-vent 300mg Capsule (Wellcome Medical Division)	other
635	Pulmadil auto Inhalation powder (3M Health Care Ltd)	nil

For peer review only

For peer review only - http://bmjopen.bmj.com/site/about/guidelines.xhtml

636	Pulmadil Inhalation powder (3M Health Care Ltd)	nil
637	Pulmicort 0.5mg Respules	ICS
638	Pulmicort 0.5mg Respules (AstraZeneca UK Ltd)	ICS
639	Pulmicort 0.5mg Respules (Necessity Supplies Ltd)	ICS
640	Pulmicort 0.5mg Respules (Waymade Healthcare Plc)	ICS
641	Pulmicort 100 Turbohaler	ICS
642	Pulmicort 100 Turbohaler (AstraZeneca UK Ltd)	ICS
643	Pulmicort 100 Turbohaler (Waymade Healthcare Plc)	ICS
644	Pulmicort 1mg Respules	ICS
645	Pulmicort 1mg Respules (AstraZeneca UK Ltd)	ICS
646	Pulmicort 1mg Respules (Sigma Pharmaceuticals Plc)	ICS
647	Pulmicort 200 Turbohaler	ICS
648	Pulmicort 200 Turbohaler (AstraZeneca UK Ltd)	ICS
649	Pulmicort 200 Turbohaler (Dowelhurst Ltd)	ICS
650	Pulmicort 200 Turbohaler (Waymade Healthcare Plc)	ICS
651	Pulmicort 200microgram Inhaler (AstraZeneca UK Ltd)	ICS
652	Pulmicort 200microgram Refill canister (AstraZeneca UK Ltd)	ICS
653	Pulmicort 200micrograms/dose inhaler (AstraZeneca UK Ltd)	ICS
654	Pulmicort 200micrograms/dose inhaler CFC free	ICS
655	Pulmicort 200micrograms/dose inhaler CFC free (AstraZeneca UK Ltd)	ICS
656	Pulmicort 400 Turbohaler	ICS
657	Pulmicort 400 Turbohaler (AstraZeneca UK Ltd)	ICS
658	Pulmicort 400 Turbohaler (Waymade Healthcare Plc)	ICS
659	Pulmicort LS 50microgram Refill canister (AstraZeneca UK Ltd)	ICS
660	Pulmicort LS 50micrograms/dose inhaler (AstraZeneca UK Ltd)	ICS
661	Pulvinal Beclometasone Dipropionate 100micrograms/dose dry powder inhaler (Chiesi Ltd)	ICS
662	Pulvinal Beclometasone Dipropionate 200micrograms/dose dry powder inhaler (Chiesi Ltd)	ICS
663	Pulvinal Beclometasone Dipropionate 400micrograms/dose dry powder inhaler (Chiesi Ltd)	ICS
664	Pulvinal Salbutamol 200micrograms/dose dry powder inhaler (Chiesi Ltd)	SABA
665	Qvar 100 Autohaler	ICS
666	Qvar 100 Autohaler (DE Pharmaceuticals)	ICS
667	Qvar 100 Autohaler (Lexon (UK) Ltd)	ICS
668	Qvar 100 Autohaler (Sigma Pharmaceuticals Plc)	ICS
669	Qvar 100 Autohaler (Stephar (U.K.) Ltd)	ICS
670	Qvar 100 Autohaler (Teva UK Ltd)	ICS
671	Qvar 100 inhaler	ICS
672	Qvar 100 inhaler (DE Pharmaceuticals)	ICS
673	Qvar 100 inhaler (Sigma Pharmaceuticals Plc)	ICS
674	Qvar 100 inhaler (Teva UK Ltd)	ICS
675	Qvar 100 inhaler (Waymade Healthcare Plc)	ICS
676	Qvar 100micrograms/dose Easi-Breathe inhaler	ICS
677	Qvar 100micrograms/dose Easi-Breathe inhaler (DE Pharmaceuticals)	ICS
678	Qvar 100micrograms/dose Easi-Breathe inhaler (Sigma Pharmaceuticals Plc)	ICS
679	Qvar 100micrograms/dose Easi-Breathe inhaler (Teva UK Ltd)	ICS
680	Qvar 50 Autohaler	ICS
681	Qvar 50 Autohaler (Teva UK Ltd)	ICS

For peer review only - http://bmjopen.bmj.com/site/about/guidelines.xhtml

682	Qvar 50 inhaler	ICS
683	Qvar 50 inhaler (DE Pharmaceuticals)	ICS
684	Qvar 50 inhaler (Mawdsley-Brooks & Company Ltd)	ICS
685	Qvar 50 inhaler (Teva UK Ltd)	ICS
686	Qvar 50micrograms/dose Easi-Breathe inhaler	ICS
687	Qvar 50micrograms/dose Easi-Breathe inhaler (Sigma Pharmaceuticals Plc)	ICS
688	Qvar 50micrograms/dose Easi-Breathe inhaler (Teva UK Ltd)	ICS
689	Relvar Ellipta 184microg/dose / 22microg/dose dry pdr inh	ICS_LABA
690	Relvar Ellipta 184micrograms/dose / 22micrograms/dose dry powder inhaler (GlaxoSmithKline UK Ltd)	ICS_LABA
691	Relvar Ellipta 92microg/dose / 22microg/dose dry pdr inh	ICS_LABA
692	Relvar Ellipta 92micrograms/dose / 22micrograms/dose dry powder inhaler (GlaxoSmithKline UK Ltd)	ICS_LABA
693	Reproterol 10mg/5ml oral solution	SABA
694	Reproterol 10mg/ml respirator solution	SABA
695	Reproterol 500micrograms/dose inhaler	SABA
696	Respacal 2mg Tablet (UCB Pharma Ltd)	LABA
697	Respontin 250micrograms/1ml Nebules (GlaxoSmithKline UK Ltd)	ipratropium
698	Respontin 500micrograms/2ml Nebules (GlaxoSmithKline UK Ltd)	ipratropium
699	Respontin nebules 250microgram/ml Nebuliser liquid (Glaxo Wellcome UK Ltd)	ipratropium
700	Rimiterol inhaler	SABA
701	Rybarvin Inhalation vapour (Rybar Laboratories Ltd)	other
702	Salamol 100microgram/actuation Inhalation powder (IVAX Pharmaceuticals UK Ltd)	SABA
703	Salamol 100microgram/inhalation Inhalation powder (Kent Pharmaceuticals Ltd)	SABA
704	Salamol 100microgram/inhalation Inhalation powder (Sandoz Ltd)	SABA
705	Salamol 100micrograms/dose Easi-Breathe inhaler (DE Pharmaceuticals)	SABA
706	Salamol 100micrograms/dose Easi-Breathe inhaler (Teva UK Ltd)	SABA
707	Salamol 100micrograms/dose inhaler CFC free (Arrow Generics Ltd)	SABA
708	Salamol 100micrograms/dose inhaler CFC free (Teva UK Ltd)	SABA
709	Salamol 2.5mg/2.5ml nebuliser liquid Steri-Neb unit dose vials (Teva UK Ltd)	SABA
710	Salamol 5mg/2.5ml nebuliser liquid Steri-Neb unit dose vials (Teva UK Ltd)	SABA
711	Salamol easi-breathe 100microgram/actuation Pressurised inhalation (IVAX Pharmaceuticals UK Ltd)	SABA
712	Salamol steri-neb 2.5mg/2.5ml Nebuliser liquid (Numark Management Ltd)	SABA
713	Salamol steri-neb 5mg/2.5ml Nebuliser liquid (Numark Management Ltd)	SABA
714	Salapin 2mg/5ml syrup (Pinewood Healthcare)	nil
715	Salbulin 100micrograms/dose inhaler (3M Health Care Ltd)	nil
716	Salbulin 2mg Tablet (3M Health Care Ltd)	nil
717	Salbulin 2mg/5ml Oral solution (3M Health Care Ltd)	nil
718	Salbulin 4mg Tablet (3M Health Care Ltd)	nil
719	SALBULIN CFC FREE	nil
720	Salbulin Inhalation powder (3M Health Care Ltd)	nil
721	Salbulin Novolizer 100micrograms/dose inhalation powder (Meda Pharmaceuticals Ltd)	nil
722	Salbulin Novolizer 100micrograms/dose inhalation powder refill (Meda Pharmaceuticals Ltd)	nil
723	SALBUTAMOL	SABA
724	SALBUTAMOL .25 MG INJ	SABA
725	Salbutamol 0.05mg/ml injection	SABA
726	Salbutamol 100microgram/inhalation Inhalation powder (Berk Pharmaceuticals Ltd)	SABA

For peer review only - http://bmjopen.bmj.com/site/about/guidelines.xhtml

727	Salbutamol 100microgram/inhalation Inhalation powder (C P Pharmaceuticals Ltd)	SABA
728	Salbutamol 100microgram/inhalation Inhalation powder (Celltech Pharma Europe Ltd)	SABA
729	Salbutamol 100microgram/inhalation Inhalation powder (IVAX Pharmaceuticals UK Ltd)	SABA
730	Salbutamol 100microgram/inhalation Inhalation powder (Kent Pharmaceuticals Ltd)	SABA
731	Salbutamol 100microgram/inhalation Inhalation powder (Neo Laboratories Ltd)	SABA
732	Salbutamol 100microgram/inhalation Spacehaler (Celltech Pharma Europe Ltd)	SABA
733	Salbutamol 100micrograms/actuation breath actuated inhaler	SABA
734	Salbutamol 100micrograms/dose / Beclometasone 50micrograms/dose inhaler	ICS_SABA
735	Salbutamol 100micrograms/dose / Ipratropium 20micrograms/dose inhaler	other
736	Salbutamol 100micrograms/dose breath actuated inhaler	SABA
737	Salbutamol 100micrograms/dose breath actuated inhaler CFC free	SABA
738	Salbutamol 100micrograms/dose dry powder inhalation cartridge	SABA
739	Salbutamol 100micrograms/dose dry powder inhalation cartridge with device	SABA
740	Salbutamol 100micrograms/dose dry powder inhaler	SABA
741	Salbutamol 100micrograms/dose inhaler	SABA
742	Salbutamol 100micrograms/dose inhaler (A A H Pharmaceuticals Ltd)	SABA
743	Salbutamol 100micrograms/dose inhaler (Kent Pharmaceuticals Ltd)	SABA
744	Salbutamol 100micrograms/dose inhaler (Mylan)	SABA
745	Salbutamol 100micrograms/dose inhaler CFC free	SABA
746	Salbutamol 100micrograms/dose inhaler CFC free (A A H Pharmaceuticals Ltd)	SABA
747	Salbutamol 100micrograms/dose inhaler CFC free (Actavis UK Ltd)	SABA
748	Salbutamol 100micrograms/dose inhaler CFC free (Alliance Healthcare (Distribution) Ltd)	SABA
749	Salbutamol 100micrograms/dose inhaler CFC free (AM Distributions (Yorkshire) Ltd)	SABA
750	Salbutamol 100micrograms/dose inhaler CFC free (DE Pharmaceuticals)	SABA
751	Salbutamol 100micrograms/dose inhaler CFC free (Mawdsley-Brooks & Company Ltd)	SABA
752	Salbutamol 100micrograms/dose inhaler CFC free (Mylan)	SABA
753	Salbutamol 100micrograms/dose inhaler CFC free (Phoenix Healthcare Distribution Ltd)	SABA
754	Salbutamol 100micrograms/dose inhaler CFC free (Sandoz Ltd)	SABA
755	Salbutamol 100micrograms/dose inhaler CFC free (Teva UK Ltd)	SABA
756	Salbutamol 100micrograms/dose inhaler CFC free (Waymade Healthcare Plc)	SABA
757	Salbutamol 100micrograms/inhalation vortex inhaler	SABA
758	SALBUTAMOL 2 MG/5ML SYR	SABA
759	Salbutamol 2.5mg with ipratropium bromide 500micrograms/2.5ml unit dose nebuilser solution	other
760	Salbutamol 2.5mg/2.5ml / Ipratropium bromide 500micrograms/2.5ml nebuliser liquid ampoules	other
761	Salbutamol 2.5mg/2.5ml / Ipratropium bromide 500micrograms/2.5ml nebuliser liquid unit dose vials	other
762	Salbutamol 2.5mg/2.5ml Nebuliser liquid (Galen Ltd)	SABA
763	Salbutamol 2.5mg/2.5ml nebuliser liquid unit dose Steripoule vials (Galen Ltd)	SABA
764	Salbutamol 2.5mg/2.5ml nebuliser liquid unit dose vials	SABA
765	Salbutamol 2.5mg/2.5ml nebuliser liquid unit dose vials (A A H Pharmaceuticals Ltd)	SABA
766	Salbutamol 2.5mg/2.5ml nebuliser liquid unit dose vials (Alliance Healthcare (Distribution) Ltd)	SABA
767	Salbutamol 200 Cyclocaps (Teva UK Ltd)	SABA
768	Salbutamol 200microgram / Beclometasone 100microgram inhalation powder capsules	SABA
769	Salbutamol 200microgram inhalation powder blisters	SABA
770	Salbutamol 200microgram inhalation powder blisters with device	SABA
771	Salbutamol 200microgram inhalation powder capsules	SABA

For peer review only - http://bmjopen.bmj.com/site/about/guidelines.xhtml

772	Salbutamol 200micrograms disc	SABA
773	Salbutamol 200micrograms inahalation capsules	SABA
774	Salbutamol 200micrograms/dose dry powder inhaler	SABA
775	Salbutamol 2mg Tablet (C P Pharmaceuticals Ltd)	SABA
776	Salbutamol 2mg tablets	SABA
777	Salbutamol 2mg tablets (Actavis UK Ltd)	SABA
778	Salbutamol 2mg tablets (Approved Prescription Services Ltd)	SABA
779	Salbutamol 2mg/5ml Oral solution (Lagap)	SABA
780	Salbutamol 2mg/5ml oral solution sugar free	SABA
781	Salbutamol 2mg/5ml oral solution sugar free (A A H Pharmaceuticals Ltd)	SABA
782	Salbutamol 2mg/5ml oral solution sugar free (Pinewood Healthcare)	SABA
783	Salbutamol 2mg/5ml oral solution sugar free (Sandoz Ltd)	SABA
784	Salbutamol 400 Cyclocaps (Teva UK Ltd)	SABA
785	SALBUTAMOL 400MCG/BECLOMETH.100MCG R/CAP INH	ICS_SABA
786	Salbutamol 400microgram / Beclometasone 200microgram inhalation powder capsules	ICS_SABA
787	Salbutamol 400microgram inhalation powder blisters	SABA
788	Salbutamol 400microgram inhalation powder blisters with device	SABA
789	Salbutamol 400microgram inhalation powder capsules	SABA
790	Salbutamol 400micrograms disc	SABA
791	Salbutamol 400micrograms inahalation capsules	SABA
792	Salbutamol 4mg modified-release capsules	SABA
793	Salbutamol 4mg modified-release tablets	SABA
794	Salbutamol 4mg tablets	SABA
795	Salbutamol 4mg tablets (A A H Pharmaceuticals Ltd)	SABA
796	Salbutamol 4mg tablets (Actavis UK Ltd)	SABA
797	Salbutamol 500micrograms/1ml solution for injection ampoules	SABA
798	Salbutamol 5mg/2.5ml Nebuliser liquid (Galen Ltd)	SABA
799	Salbutamol 5mg/2.5ml Nebuliser liquid (Generics (UK) Ltd)	SABA
800	Salbutamol 5mg/2.5ml nebuliser liquid unit dose Steripoule vials (Galen Ltd)	SABA
801	Salbutamol 5mg/2.5ml nebuliser liquid unit dose vials	SABA
802	Salbutamol 5mg/2.5ml nebuliser liquid unit dose vials (Actavis UK Ltd)	SABA
803	Salbutamol 5mg/2.5ml nebuliser liquid unit dose vials (Alliance Healthcare (Distribution) Ltd)	SABA
804	Salbutamol 5mg/50ml solution for infusion vials	SABA
805	Salbutamol 5mg/5ml solution for infusion ampoules	SABA
806	Salbutamol 5mg/ml nebuliser liquid	SABA
807	Salbutamol 8mg modified-release capsules	SABA
808	Salbutamol 8mg modified-release tablets	SABA
809	Salbutamol 8mg tablet	SABA
810	Salbutamol 95micrograms/dose dry powder inhaler	SABA
811	SALBUTAMOL CFC/FREE B/A	SABA
812	Salbutamol cyclocaps 200microgram Inhalation powder (DuPont Pharmaceuticals Ltd)	SABA
813	Salbutamol cyclocaps 400microgram Inhalation powder (DuPont Pharmaceuticals Ltd)	SABA
814	Salbutamol cyclohaler	SABA
815	SALBUTAMOL INHALER	SABA
816	SALBUTAMOL RESPIRATOR SOLN	SABA
817	Salbutamol rondo 100micrograms/actuation inhaler and spacer	SABA

For peer review only - http://bmjopen.bmj.com/site/about/guidelines.xhtml

818	SALBUTAMOL ROTAHALER COMPLETE UNIT	SABA
819	SALBUTAMOL U.DOSE NEBULISING 2.5MG/2.5ML	SABA
820	Salbuvent 0.5mg/ml Injection (Pharmacia Ltd)	SABA
821	Salbuvent 100microgram/actuation Inhalation powder (Pharmacia Ltd)	SABA
822	Salbuvent 2mg Tablet (Pharmacia Ltd)	SABA
823	Salbuvent 2mg/5ml Oral solution (Pharmacia Ltd)	SABA
824	Salbuvent 4mg Tablet (Pharmacia Ltd)	SABA
825	Salbuvent 5mg/ml Respirator solution (Pharmacia Ltd)	SABA
826	SALBUVENT inh INH	SABA
827	SALBUVENT RONDO	SABA
828	Salipraneb 0.5mg/2.5mg nebuliser solution 2.5ml ampoules (Actavis UK Ltd)	other
829	Salipraneb 0.5mg/2.5mg nebuliser solution 2.5ml ampoules (Arrow Generics Ltd)	other
830	Salmeterol 25micrograms with fluticasone 125micrograms CFC free inhaler	ICS_LABA
831	Salmeterol 25micrograms with fluticasone 250micrograms CFC free inhaler	ICS_LABA
832	Salmeterol 25micrograms with fluticasone 50micrograms CFC free inhaler	ICS_LABA
833	Salmeterol 25micrograms/dose inhaler	LABA
834	Salmeterol 25micrograms/dose inhaler CFC free	LABA
835	Salmeterol 25micrograms/dose inhaler CFC free (A A H Pharmaceuticals Ltd)	LABA
836	Salmeterol 50microgram Diskhaler (Dowelhurst Ltd)	LABA
837	Salmeterol 50microgram inhalation pdr blist with device	LABA
838	Salmeterol 50microgram inhalation powder blisters	LABA
839	Salmeterol 50microgram inhalation powder blisters with device	LABA
840	Salmeterol 50micrograms disc	LABA
841	Salmeterol 50micrograms with fluticasone 100micrograms dry powder inhaler	ICS_LABA
842	Salmeterol 50micrograms with fluticasone 250micrograms CFC free inhaler	ICS_LABA
843	Salmeterol 50micrograms with fluticasone 500micrograms CFC free inhaler	ICS_LABA
844	Salmeterol 50micrograms/dose dry powder inhaler	LABA
845	Seebri Breezhaler 44microgram inhalation pdr caps with dev	LAMA
846	Seebri Breezhaler 44microgram inhalation powder capsules with device (Novartis Pharmaceuticals UK Ltd)	LAMA
847	Sereflo 25micrograms/dose / 125micrograms/dose inhaler	ICS_LABA
848	Sereflo 25micrograms/dose / 125micrograms/dose inhaler (Kent Pharmaceuticals Ltd)	ICS_LABA
849	Sereflo 25micrograms/dose / 250micrograms/dose inhaler	ICS_LABA
850	Sereflo 25micrograms/dose / 250micrograms/dose inhaler (Kent Pharmaceuticals Ltd)	ICS_LABA
851	Seretide 100 Accuhaler	ICS_LABA
852	Seretide 100 Accuhaler (DE Pharmaceuticals)	ICS_LABA
853	Seretide 100 Accuhaler (GlaxoSmithKline UK Ltd)	ICS_LABA
854	Seretide 100 Accuhaler (Waymade Healthcare Plc)	ICS_LABA
855	Seretide 125 Evohaler	ICS_LABA
856	Seretide 125 Evohaler (DE Pharmaceuticals)	ICS_LABA
857	Seretide 125 Evohaler (GlaxoSmithKline UK Ltd)	ICS_LABA
858	Seretide 125 Evohaler (Lexon (UK) Ltd)	ICS_LABA
859	Seretide 125 Evohaler (Mawdsley-Brooks & Company Ltd)	ICS_LABA
860	Seretide 125 Evohaler (Waymade Healthcare Plc)	ICS_LABA
861	Seretide 250 Accuhaler	ICS_LABA
862	Seretide 250 Accuhaler (DE Pharmaceuticals)	ICS_LABA
863	Seretide 250 Accuhaler (GlaxoSmithKline UK Ltd)	ICS_LABA

For peer review only - http://bmjopen.bmj.com/site/about/guidelines.xhtml

864	Seretide 250 Accuhaler (Lexon (UK) Ltd)	ICS_LABA
865	Seretide 250 Accuhaler (Sigma Pharmaceuticals Plc)	ICS_LABA
866	Seretide 250 Accuhaler (Waymade Healthcare Plc)	ICS_LABA
867	Seretide 250 Evohaler	ICS_LABA
868	Seretide 250 Evohaler (DE Pharmaceuticals)	ICS_LABA
869	Seretide 250 Evohaler (GlaxoSmithKline UK Ltd)	ICS_LABA
870	Seretide 250 Evohaler (Lexon (UK) Ltd)	ICS_LABA
871	Seretide 250 Evohaler (Necessity Supplies Ltd)	ICS_LABA
872	Seretide 250 Evohaler (Stephar (U.K.) Ltd)	ICS_LABA
873	Seretide 250 Evohaler (Waymade Healthcare Plc)	ICS_LABA
874	Seretide 50 Evohaler	ICS_LABA
875	Seretide 50 Evohaler (GlaxoSmithKline UK Ltd)	ICS_LABA
876	Seretide 500 Accuhaler	ICS_LABA
877	Seretide 500 Accuhaler (DE Pharmaceuticals)	ICS_LABA
878	Seretide 500 Accuhaler (GlaxoSmithKline UK Ltd)	ICS_LABA
879	Seretide 500 Accuhaler (Lexon (UK) Ltd)	ICS_LABA
880	Seretide 500 Accuhaler (Mawdsley-Brooks & Company Ltd)	ICS_LABA
881	Seretide 500 Accuhaler (Necessity Supplies Ltd)	ICS_LABA
882	Seretide 500 Accuhaler (Waymade Healthcare Plc)	ICS_LABA
883	Serevent 25micrograms/dose Evohaler	LABA
884	Serevent 25micrograms/dose Evohaler (GlaxoSmithKline UK Ltd)	LABA
885	Serevent 25micrograms/dose Evohaler (Lexon (UK) Ltd)	LABA
886	Serevent 25micrograms/dose Evohaler (Waymade Healthcare Plc)	LABA
887	Serevent 25micrograms/dose inhaler	LABA
888	Serevent 50microgram disks (GlaxoSmithKline UK Ltd)	LABA
889	Serevent 50microgram disks with Diskhaler	LABA
890	Serevent 50microgram disks with Diskhaler (GlaxoSmithKline UK Ltd)	LABA
891	Serevent 50micrograms/dose Accuhaler	LABA
892	Serevent 50micrograms/dose Accuhaler (DE Pharmaceuticals)	LABA
893	Serevent 50micrograms/dose Accuhaler (GlaxoSmithKline UK Ltd)	LABA
894	Serevent 50micrograms/dose Accuhaler (Mawdsley-Brooks & Company Ltd)	LABA
895	Serevent 50micrograms/dose Accuhaler (Waymade Healthcare Plc)	LABA
896	Serevent diskhaler 50microgram Inhalation powder (Glaxo Wellcome UK Ltd)	LABA
897	Singulair 10mg tablets (DE Pharmaceuticals)	montelukast
898	Singulair 10mg tablets (Merck Sharp & Dohme Ltd)	montelukast
899	Singulair 10mg tablets (Necessity Supplies Ltd)	montelukast
900	Singulair Paediatric 4mg chewable tablets (Merck Sharp & Dohme Ltd)	montelukast
901	Singulair Paediatric 4mg granules sachets (Mawdsley-Brooks & Company Ltd)	montelukast
902	Singulair Paediatric 4mg granules sachets (Merck Sharp & Dohme Ltd)	montelukast
903	Singulair Paediatric 5mg chewable tablets (Merck Sharp & Dohme Ltd)	montelukast
904	Sirdupla 25micrograms/dose / 125micrograms/dose inhaler	ICS_LABA
905	Sirdupla 25micrograms/dose / 125micrograms/dose inhaler (Mylan)	ICS_LABA
906	Sirdupla 25micrograms/dose / 250micrograms/dose inhaler	ICS_LABA
907	Sirdupla 25micrograms/dose / 250micrograms/dose inhaler (Mylan)	ICS_LABA
908	Sirdupla 25micrograms/dose / 250micrograms/dose inhaler (Waymade Healthcare Plc)	ICS_LABA
909	Slo-phyllin 125mg Capsule (Lipha Pharmaceuticals Ltd)	other

For peer review only - http://bmjopen.bmj.com/site/about/guidelines.xhtml

910	Slo-Phyllin 125mg capsules (Merck Serono Ltd)	other
911	Slo-phyllin 250mg Capsule (Lipha Pharmaceuticals Ltd)	other
912	Slo-Phyllin 250mg capsules (Merck Serono Ltd)	other
913	Slo-phyllin 60mg Capsule (Lipha Pharmaceuticals Ltd)	other
914	Slo-Phyllin 60mg capsules (Merck Serono Ltd)	other
915	Sodium cromoglicate 1mg/dose / Salbutamol 100micrograms/dose inhaler	other
916	Sodium cromoglicate 1mg/dose / Salbutamol 100micrograms/dose inhaler with spacer	other
917	Sodium cromoglicate 1mg/inhalation inhaler	other
918	Sodium cromoglicate 20mg/2ml nebuliser liquid unit dose vials	other
919	Sodium cromoglicate 5mg/dose inhaler CFC free	other
920	Sodium cromoglicate 5mg/inhalation inhaler & spacer	other
921	Sodium cromoglicate with isoprenaline inhalation capsules	other
922	Soltel 25micrograms/dose inhaler CFC free	LABA
923	Soltel 25micrograms/dose inhaler CFC free (Kent Pharmaceuticals Ltd)	LABA
924	Soprobec 100micrograms/dose inhaler	ICS
925	Soprobec 200micrograms/dose inhaler	ICS
926	Soprobec 250micrograms/dose inhaler	ICS
927	Soprobec 50micrograms/dose inhaler	ICS
928	Spacehaler BDP 100microgram/actuation Spacehaler (Celltech Pharma Europe Ltd)	other
929	Spacehaler BDP 250microgram/actuation Spacehaler (Celltech Pharma Europe Ltd)	other
930	Spacehaler BDP 50microgram/actuation Spacehaler (Celltech Pharma Europe Ltd)	other
931	Spacehaler salbutamol 100microgram/inhalation Spacehaler (Celltech Pharma Europe Ltd)	other
932	Spiolto Respimat 2.5microg/dose / 2.5microg/dose soln refill	LAMA_LABA
933	Spiolto Respimat 2.5microg/dose/2.5microg/dose soln	LAMA_LABA
934	Spiolto Respimat 2.5micrograms/dose / 2.5micrograms/dose solution for inhalation cartridge with device (Boehringer Ingelheim Ltd)	LAMA_LABA
935	Spiriva 18 microgram Capsule (Boehringer Ingelheim Ltd)	LAMA
936	Spiriva 18microgram inhalation pdr caps with HandiHaler	LAMA
937	Spiriva 18microgram inhalation powder capsules	LAMA
938	Spiriva 18microgram inhalation powder capsules (Boehringer Ingelheim Ltd)	LAMA
939	Spiriva 18microgram inhalation powder capsules (Mawdsley-Brooks & Company Ltd)	LAMA
940	Spiriva 18microgram inhalation powder capsules (Sigma Pharmaceuticals Plc)	LAMA
941	Spiriva 18microgram inhalation powder capsules with HandiHaler (Boehringer Ingelheim Ltd)	LAMA
942	Spiriva 18microgram inhalation powder capsules with HandiHaler (DE Pharmaceuticals)	LAMA
943	Spiriva 18microgram inhalation powder capsules with HandiHaler (Sigma Pharmaceuticals Plc)	LAMA
944	Spiriva 18microgram inhalation powder capsules with HandiHaler (Waymade Healthcare Plc)	LAMA
945	Spiriva Respimat 2.5microg/dose inhalation soln	LAMA
946	Spiriva Respimat 2.5microg/dose inhalation soln refill cart	LAMA
947	Spiriva Respimat 2.5micrograms/dose solution for inhalation cartridge with device (Boehringer Ingelheim Ltd)	LAMA
948	Spiriva Respimat 2.5micrograms/dose solution for inhalation cartridge with device (Waymade Healthcare Plc)	LAMA
949	Stalpex 50microg/dose / 500microg/dose dry powder inh	ICS_LABA
950	Steri-neb cromogen 10mg/ml Nebuliser liquid (IVAX Pharmaceuticals UK Ltd)	other
951	Steri-neb ipratropium 250microgram/ml Nebuliser liquid (IVAX Pharmaceuticals UK Ltd)	LAMA
952	Striverdi Respimat 2.5microg/dose inhalation soln	LABA
953	Striverdi Respimat 2.5microg/dose inhalation soln refill	LABA
954	Striverdi Respimat 2.5micrograms/dose solution for inhalation cartridge with device (Boehringer Ingelheim Ltd)	LABA

For peer review only - http://bmjopen.bmj.com/site/about/guidelines.xhtml

955	Symbicort 100/6 Turbohaler	ICS_LABA
956	Symbicort 100/6 Turbohaler (AstraZeneca UK Ltd)	ICS_LABA
957	Symbicort 100/6 Turbohaler (Mawdsley-Brooks & Company Ltd)	ICS_LABA
958	Symbicort 100/6 Turbohaler (Sigma Pharmaceuticals Plc)	ICS_LABA
959	Symbicort 200/6 Turbohaler	ICS_LABA
960	Symbicort 200/6 Turbohaler (AstraZeneca UK Ltd)	ICS_LABA
961	Symbicort 200/6 Turbohaler (DE Pharmaceuticals)	ICS_LABA
962	Symbicort 200/6 Turbohaler (Mawdsley-Brooks & Company Ltd)	ICS_LABA
963	Symbicort 200/6 Turbohaler (Necessity Supplies Ltd)	ICS_LABA
964	Symbicort 200/6 Turbohaler (Sigma Pharmaceuticals Plc)	ICS_LABA
965	Symbicort 200microg/dose / 6microg/dose pressurised inh	ICS_LABA
966	Symbicort 200micrograms/dose / 6micrograms/dose pressurised inhaler (AstraZeneca UK Ltd)	ICS_LABA
967	Symbicort 400/12 Turbohaler	ICS_LABA
968	Symbicort 400/12 Turbohaler (AstraZeneca UK Ltd)	ICS_LABA
969	Symbicort 400/12 Turbohaler (DE Pharmaceuticals)	ICS_LABA
970	Symbicort 400/12 Turbohaler (Mawdsley-Brooks & Company Ltd)	ICS_LABA
971	Tedral Oral solution (Parke-davis Research Laboratories)	other
972	Tedral Tablet (Parke-davis Research Laboratories)	other
973	Terbutaline 1.5mg/5ml Oral solution (Sandoz Ltd)	SABA
974	Terbutaline 1.5mg/5ml oral solution sugar free	SABA
975	Terbutaline 2.5mg/5ml solution for injection ampoules	SABA
976	Terbutaline 250micrograms/actuation refill canister	SABA
977	Terbutaline 500micrograms/1ml solution for injection ampoules	SABA
978	Terbutaline 500micrograms/dose dry powder inhaler	SABA
979	Terbutaline 500micrograms/ml injection	SABA
980	Terbutaline 5mg tablets	SABA
981	Terbutaline 5mg/2ml nebuliser liquid unit dose vials	SABA
982	Terbutaline with guafenesin expectorant	SABA
983	Theodrox Tablet (3M Health Care Ltd)	other
984	Theo-Dur 200mg tablets (AstraZeneca UK Ltd)	other
985	Theo-Dur 300mg tablets (AstraZeneca UK Ltd)	other
986	Theograd 350mg Tablet (Abbott Laboratories Ltd)	other
987	Theophylline 10mg/5ml SF elixir	other
988	Theophylline 125mg modified-release capsules	other
989	Theophylline 125mg/5ml syrup	other
990	Theophylline 175mg modified-release tablets	other
991	Theophylline 200mg modified-release tablets	other
992	Theophylline 250mg modified-release capsules	other
993	Theophylline 250mg modified-release tablets	other
994	Theophylline 250mg/5ml oral suspension	other
995	Theophylline 300mg modified release capsules	other
996	Theophylline 300mg modified-release tablets	other
997	Theophylline 350mg modified release tablets	other
998	Theophylline 400mg modified-release tablets	other
999	Theophylline 500mg modified release tablets	other
1000	Theophylline 60mg modified-release capsules	other

For peer review only - http://bmjopen.bmj.com/site/about/guidelines.xhtml

1001	Theophylline 60mg/5ml oral solution	other
1002	Theophylline 60mg/5ml oral suspension	other
1003	Theophylline with ephedrine & caffeine tablets	other
1004	Tilade 2mg/dose inhaler CFC free (Sanofi)	nil
1005	Tilade 2mg/inhalation Inhalation powder (Sanofi)	nil
1006	Tilarin 1% Nasal spray suspension (Rhone-Poulenc Rorer Ltd)	nil
1007	Tiotropium 18 microgram Capsule	LAMA
1008	Tiotropium brom 2.5microg/dose soln for inhalation CFCfree	LAMA
1009	Tiotropium brom 2.5microg/Olodaterol 2.5microg/dose soln	LAMA_LABA
1010	Tiotropium brom 2.5microg/Olodaterol 2.5microg/dose soln&dev	LAMA_LABA
1011	Tiotropium bromide 10microgram inhalation pdr caps with dev	LAMA
1012	Tiotropium bromide 10microgram inhalation powder capsules with device	LAMA
1013	Tiotropium bromide 18microgram inhalation pdr caps with dev	LAMA
1014	Tiotropium bromide 18microgram inhalation powder capsules	LAMA
1015	Tiotropium bromide 18microgram inhalation powder capsules with device	LAMA
1016	Tiotropium bromide 2.5micrograms/dose / Olodaterol 2.5micrograms/dose solution for inhalation cartridge with device CFC free	LAMA_LABA
1017	Tiotropium bromide 2.5micrograms/dose inhalation soln cart	LAMA
1018	Tiotropium bromide 2.5micrograms/dose solution for inhalation cartridge with device CFC free	LAMA
1019	Tiotropium bromide 2.5micrograms/dose solution for inhalation cartridge with device CFC free (AM Distributions (Yorkshire) Ltd)	LAMA
1020	Trelegy Ellipta 92microg/55microg/22microg/dose dry pdr inh	triple
1021	Trimbow 87microg/dose / 5microg/dose / 9microg /dose inh	triple
1022	Tropiovent steripoule 250microgram/ml Nebuliser liquid (Ashbourne Pharmaceuticals Ltd)	LAMA
1023	Tulobuterol 1mg/5ml sugar free syrup	LABA
1024	Tulobuterol 2mg	LABA
1025	Ultibro Breezhaler 85microg/43microg pdr caps with dev	LAMA_LABA
1026	Ultibro Breezhaler 85microgram/43microgram inhalation powder capsules with device (Novartis Pharmaceuticals UK Ltd)	LAMA_LABA
1027	Umeclidinium brom 65microg/Vilanterol 22microg/dose dry pdr	LAMA_LABA
1028	Umeclidinium bromide 65micrograms/dose / Vilanterol 22micrograms/dose dry powder inhaler	LAMA_LABA
1029	Umeclidinium bromide 65micrograms/dose dry powder inhaler	LAMA
1030	Uniphyllin Continus 200mg tablets (Napp Pharmaceuticals Ltd)	other
1031	Uniphyllin Continus 300mg tablets (Napp Pharmaceuticals Ltd)	other
1032	Uniphyllin Continus 400mg tablets (Napp Pharmaceuticals Ltd)	other
1033	Ventmax SR 4mg capsules (Chiesi Ltd)	SABA
1034	Ventmax SR 8mg capsules (Chiesi Ltd)	SABA
1035	Ventodisks 200microgram/blister Disc (Allen & Hanburys Ltd)	SABA
1036	Ventodisks 400microgram/blister Disc (Allen & Hanburys Ltd)	SABA
1037	VENTOLIN	SABA
1038	VENTOLIN .25 MG INJ	SABA
1039	Ventolin 100microgram/inhalation Inhalation powder (Glaxo Wellcome UK Ltd)	SABA
1040	Ventolin 100micrograms/dose Evohaler (DE Pharmaceuticals)	SABA
1041	Ventolin 100micrograms/dose Evohaler (GlaxoSmithKline UK Ltd)	SABA
1042	Ventolin 100micrograms/dose Evohaler (Mawdsley-Brooks & Company Ltd)	SABA
1043	Ventolin 100micrograms/dose Evohaler (Waymade Healthcare Plc)	SABA
1044	Ventolin 2.5mg Nebules (GlaxoSmithKline UK Ltd)	SABA
1045	Ventolin 2.5mg Nebules (Mawdsley-Brooks & Company Ltd)	SABA

For peer review only - http://bmjopen.bmj.com/site/about/guidelines.xhtml

1046	Ventolin 200microgram Rotacaps (GlaxoSmithKline UK Ltd)	SABA
1047	Ventolin 200micrograms/dose Accuhaler (DE Pharmaceuticals)	SABA
1048	Ventolin 200micrograms/dose Accuhaler (Dowelhurst Ltd)	SABA
1049	Ventolin 200micrograms/dose Accuhaler (GlaxoSmithKline UK Ltd)	SABA
1050	Ventolin 200micrograms/dose Accuhaler (Lexon (UK) Ltd)	SABA
1051	Ventolin 200micrograms/dose Accuhaler (Mawdsley-Brooks & Company Ltd)	SABA
1052	Ventolin 200micrograms/dose Accuhaler (Sigma Pharmaceuticals Plc)	SABA
1053	Ventolin 200micrograms/dose Accuhaler (Waymade Healthcare Plc)	SABA
1054	Ventolin 2mg Tablet (Allen & Hanburys Ltd)	SABA
1055	Ventolin 2mg/5ml syrup (GlaxoSmithKline UK Ltd)	SABA
1056	Ventolin 400microgram Rotacaps (GlaxoSmithKline UK Ltd)	SABA
1057	Ventolin 4mg Tablet (Allen & Hanburys Ltd)	SABA
1058	Ventolin 500micrograms/1ml solution for injection ampoules (GlaxoSmithKline UK Ltd)	SABA
1059	Ventolin 50microgram/ml Injection (Allen & Hanburys Ltd)	SABA
1060	Ventolin 5mg Nebules (GlaxoSmithKline UK Ltd)	SABA
1061	Ventolin 5mg/5ml solution for infusion ampoules (GlaxoSmithKline UK Ltd)	SABA
1062	Ventolin 5mg/ml respirator solution (GlaxoSmithKline UK Ltd)	SABA
1063	Ventolin accuhaler 200 200microgram/actuation Inhalation powder (Glaxo Wellcome UK Ltd)	SABA
1064	Ventolin cr 4mg Tablet (Allen & Hanburys Ltd)	SABA
1065	Ventolin cr 8mg Tablet (Allen & Hanburys Ltd)	SABA
1066	Ventolin easi-breathe 100microgram/actuation Pressurised inhalation (Allen & Hanburys Ltd)	SABA
1067	Ventolin evohaler 100 100microgram/inhalation Pressurised inhalation (Glaxo Wellcome UK Ltd)	SABA
1068	VENTOLIN I/V 5 MG INJ	SABA
1069	VENTOLIN NEBULES	SABA
1070	VENTOLIN RESPIRATOR	SABA
1071	VENTOLIN ROTACAPS	SABA
1072	VENTOLIN ROTAHALER	SABA
1073	Ventolin Rotahaler (GlaxoSmithKline UK Ltd)	SABA
1074	VENTOLIN S/R	SABA
1075	VENTOLIN S/R 8 MG SPA	SABA
1076	Vertine 25micrograms/dose inhaler CFC free	LABA
1077	Vertine 25micrograms/dose inhaler CFC free (Teva UK Ltd)	LABA
1078	Yanimo Respimat 2.5microg/dose / 2.5microg/dose soln	LAMA_LABA
1079	Zafirlukast 20mg tablets	other

For peer review only

For peer review only - http://bmjopen.bmj.com/site/about/guidelines.xhtml

Supplementary Material C: Detail of Approach to Cluster Analysis

Data were analysed in *RStudio* version 1.2.5033 (driven by *R* version 3.6.0) on a Windows 10 desktop computer.[1] Additional packages were loaded from The Comprehensive R Archive Network (https://cran.r-project.org/).[2]

There are a variety of methods and tools which can be used for population segmentation to help enable integrated healthcare models.[3] A comprehensive review of population segmentation methods is provided by Wood, Murch and Betteridge.[4] Data driven population segmentation has been applied for Population Health Management (PHM) for a wide variety of different clinical contexts.[5] The most commonly used data driven segmentation analysis methods are latent class, k-means cluster analysis and hierarchical analysis.[5] Cluster analysis is an unsupervised machine learning approach which can be used to identify patterns and attributes between different segments of people. Clustering is used to group data into similar groups which are designated as 'clusters'. Machine learning methods are only recently being applied to PHM. The linked dataset can be considered big data with different types of variables in which nonparametric machine learning approaches may be best placed for certain types of analyses. Although more statistical based approaches such as decision trees are recommended for utilisation based population segmentation studies,[4] cluster analysis is a useful non-objective exploratory tool when there are no a priori hypotheses or particular outcomes to model. There are several clustering methods which can be applied to larger datasets including k-means, k-modes and k-prototypes (which is in essence a combination of both the k-means and k-modes algorithms). K-means is typically used for continuous data types and requires the value of k (the number of clusters) to be determined upfront. K-modes is an analogue of k-means but requires categorical data.

The clustering method used for this analysis was k-prototypes. This method allows clustering on mixed data types (both continuous variables and categorical). This algorithm can also handle larger datasets where hierarchical clustering methods may be impractical. The R package 'clustMixType' was used for analyses which is based on Huang's k-prototypes algorithm.[2 6 7] This algorithm uses the Euclidean distance measure (a dissimilarity metric to measure similarity between observations) for numerical data and simple matching dissimilarity measures for categorical data. K-prototypes has been previously employed to identify vulnerable populations for malaria in the Yunnan Province of China.[8] A summary of the cluster analysis methods is given in Table C.1 below which covers the 5 reporting criteria by Aldenderfer and Blashfield.[9 10]

Reporting Criteria	
1 – Computer Software	R, clustMixType Package,[6] based on Huang's k-prototypes algorithm[7]
2 – Similarity Measure	The Euclidean distance measure for numerical data and simple matching dissimilarity measures for categorical data
3 – Cluster Method	k-prototypes
4 – Decision on number of clusters	Both data driven and through interpretation. Scree plot showing elbow point for total within sum of squares by number of clusters and Silhouette Index. Use of 6 criteria reported by Chong, Lim and Matchar for healthcare segmentation including actionability, identifiability and responsiveness.
5 – Evidence of Cluster Validity	Expert Review - Clinician Review of clusters produced for 6,7,8 and 9 cluster solutions, Silhouette Index, external comparison with literature.

Table C.1: 5 Reporting Criteria for cluster analysis by Aldenderfer and Blashfield.[9 10]

The number of clusters (or segments) was determined both empirically from the data and by interpretation of the segments and clinical context. The total within sum of squares was plotted across different numbers of clusters (1-10) to identify a point in which the curve begins to flatten out. In addition, the Silhouette index was applied to assess cluster validity where the maximum value of the index was used to determine the optimal number of clusters.[11] A consideration of the healthcare context was used in order that each segment has a 'relatively substantial' portion of the sample population as well as being responsive and actionable in order to benefit from service based intervention.[3] A population segmentation scheme for healthcare policy can be determined through 6

For peer review only - http://bmjopen.bmj.com/site/about/guidelines.xhtml

criteria described by Chong, Lim and Matchar (2019) as substantiality, responsiveness, actionability, identifiability and accessibility.[3]

The k-protoypes algorithm repeats until a stopping criterion is reached, therefore the maximum number of iterations used for the algorithm was 1,000,000 to enable the algorithm to converge. In addition, to overcome the random component of the algorithm (variation arising due to random choice of the initial prototype observations) it was run 1000 times with the best outcome across all runs being selected as the single outcome. As well as the number of clusters needing to be determined, another parameter 'λ' which represents the trade-off between numerical data (Euclidean distance) and categorical data (simple matching distance) also needs to be selected. 'λ' was determined as 3.06 based on the average variance over all numeric variables related to the average concentration of all categorical variables.[2]

The k-prototypes clustering method was applied to 6,7,8 and 9 clusters and these were reviewed by two clinicians and assessed for their usefulness in terms of the criteria discussed above including identifiability and actionability. Cluster profiles were also produced to provide further information on the homogeneity of clusters (cluster profiles for the 6 cluster solution is given in Figure C.2 below). Cluster stability was observed since 7 clusters reproduced the groups identified for the 6 cluster solution but with an additional cancer group with a higher occurrence of non-elective admissions. Similar groupings were found for 8 and 9 clusters, but they produced separate groups for the same condition for male and female which could be better captured within one cluster. Ultimately 6 clusters were selected based on a combination of these investigations (see Table C.4-C.6 for the results of this clustering).

Number of Clusters	Within Sum of Squares	Silhouette Index
2	309,757.1	0.160
3	286,488.8	0.174
4	264,354.7	0.190
5	243,689.9	0.187
6	227,313.3	0.192
7	217,342.0	0.196
8	206,578.7	0.178
9	196,894.5	0.193
10	191,006.9	0.184

Table C.2: Indexes to assess cluster validity. The maximum value of the Silhouette index is used to determine the optimal number of clusters (20 nstarts, 100,000 iterations). The within sum of squares values are plotted on a scree plot to determine an elbow point when the curve begins to flatten out.

Data were pre-processed before cluster analyses to ensure variables were on the same scale (continuous variables were scaled). A smaller proportion of the population use a large proportion of available healthcare which can skew the data. As such, the top 0.1% of people with the highest healthcare utilisation were excluded from the analyses to aid with the clustering process as clustering algorithms can be sensitive to outliers. Removing the top utilisers (0.4%) left a high-risk population of 29,454. The attributes considered included those cited as core indicators[12] and through discussion with clinicians. Previous work by the authors suggest including utilisation variables to add increased discriminative ability. Using the number of co-morbid conditions has been shown to act as a proxy for the care needs of a group.[13] Mental health needs of the high-risk group who have been asked to isolate for 12 weeks will be included by assessing number of previous mental health appointments in the past 12 months along with community support accessed during the last 12 months. Utilisation variables consisted of combined appointments since the more variables included in the clustering, the more they will dilute other variables and so the following strategy was employed to maintain a good balance of information: Primary care and community care contacts were combined, secondary care elective appointments included both outpatient and in-patient appointments and secondary care non-elective appointments included A&E and non-elective in- patient appointments. Clustering variables were included based on

For peer review only - http://bmjopen.bmj.com/site/about/guidelines.xhtml

whether the variable could assist the following: targeting advice/guidance, identification of those most in need to home deliveries of critical supplies, informing distribution of healthcare resources and services. Variables were grouped where appropriate to ensure there were not too many variables which may dilute the effects of others. Full List shown in **Table 1** below.

Variable	How the Variable is Operationalised
**Clustering Variables**	
**Demographics**	
Age	Continuous: Current age
Sex	Categorical: Male/Female
**Healthcare Utilisation (calendar year 2019)**	
Primary Care (GP) and Community contacts (previous calendar year 2019)	Continuous count
Mental health attendances (previous calendar year 2019)	Continuous count
Secondary care elective (outpatient and elective in patient appointments) (previous calendar year 2019)	Continuous count
Secondary care non-elective (accident and emergency and non-elective in patient appointments) (previous calendar year 2019)	Continuous count
**Co-morbidities and other health conditions**	
Cardiovascular condition (current)	Binary: Based on the presence of myocardial infarction or heart failure or peripheral vascular disease or stroke
Cancer diagnosed in the past 5 years	Binary: Based on the presence of the following in the past 5 years: lung cancer, breast cancer, bowel cancer, prostate cancer, leukaemia/lymphoma, cervical cancer, ovarian cancer, melanoma, head and neck cancer, upper GI/liver cancer, other cancer, metastatic cancer, bladder cancer, kidney cancer
Mental Health	Binary: Based on the presence of depression or QoF mental health
Diabetes	Binary: Based on diabetes no complications, diabetes end complications, QoF diabetes
Dementia	Binary: Based on a record of Dementia
Asthma	Binary: Based on if there is a record for asthma and an asthma related prescription in the preceding 12 months (March 2019 to March 2020)
COPD	Binary: Based on a record of COPD
**Other**	
Drugs that require monitoring or administrating	Binary: Based on a record of medication that requires monitoring in the previous 2 months (Drugs requiring monitoring)<br><br>Or a record of a prescription of an IMMIMM or IMMBIO in the previous 6 months (or previous 12 months for rituximab).<br><br>Full lists of drugs included in **Supplementary Material B**
**Non clustering variables**	
**Lifestyle Factors**	
Smoking	Categorical: current smoker, non-smoker
IMD Decile	Continuous: Based on current LSOA as recorded by GP practice<br>The Index of Multiple Deprivation (IMD) ranks LSOAs from most to least deprived on a scale from one to ten
Urban/Rural	Categorical: Urban City and Town/Rural town and fringe/Rural village and dispersed<br>Using the Rural Urban Classification (2011) of Lower Layer Super Output Areas in England and Wales.
Learning disabilities and autism	Based on a QoF record of a learning disability and a record of autism
Housebound	Binary variable
Has a carer	Binary variable
Is a carer	Binary variable
Charlson comorbidity score[14]	Continuous

Table C.3: Clustering and non-clustering variables used for analyses and how they are operationalised.

The resulting segments/clusters were then analysed by assessing the different attributes, comorbidities and utilisation variables. Additional factors of interest were also summarised for each of the clusters (non-cluster variables). These included social status factors such as whether someone is housebound or whether they have a carer to determine if a particular cluster may benefit from having medications or food delivered or to ensure they are still receiving appropriate healthcare. Both clustering variables and non-clustering variables were assessed for whether they were statistically different from other clusters,

For peer review only - http://bmjopen.bmj.com/site/about/guidelines.xhtml

an approach used by other studies to verify results and to indicate how segments differ on each clustering variable.[15 16] Firstly an omnibus statistical test was applied (ANOVA, Kruskal-Wallis, Chi square test) to confirm differences across clusters. ANOVA for example determines if at least one group mean differs from the others and the Kruskal-Wallis test is the non-parametric equivalent. This omnibus testing was followed by post hoc pairwise comparisons. 15 pairwise tests between all other clusters were used (t-test, Mann-Whitney U test and z-test for proportions) to assign whether the cluster was significantly different from 5, 4, or 3 other clusters indicated with a superscript a,b or c. A Bonferroni adjustment was made to the significance level of 0.05 due to using multiple test comparisons which increases the probability of at least one false rejection of the null hypothesis (0.05/15 = 0.0033 (based on 15 pairwise tests).

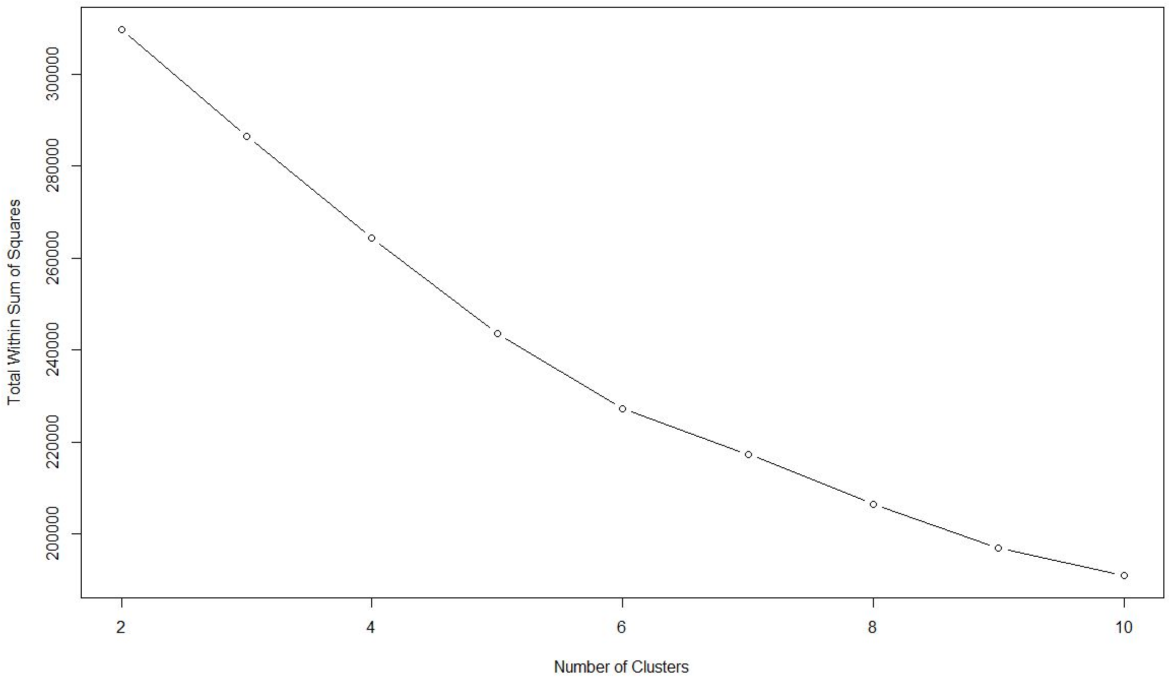

Figure C.1: Scree plot showing the Total within sum of squares for differing numbers of clusters used in the K-prototypes clustering model. There is an elbow point for 6 clusters and so this was investigated for population segmentation. The models used 50 nstarts (repetitive computations with random initialisations with selection of the result with minimum distance) and 100,000 iterations to produce this plot.

For peer review only - http://bmjopen.bmj.com/site/about/guidelines.xhtml

7 Clusters

Cluster	Cluster 1	Cluster 2	Cluster 3	Cluster 4	Cluster 5	Cluster 6	Cluster 7	Total Population
**Total**	**5066**	**1874**	**6772**	**8649**	**5684**	**1241**	**168**	**29,454**
**Proportion of population**	**17.20%**	**6.36%**	**22.99%**	**29.36%**	**19.30%**	**4.21%**	**0.57%**	**100.00%**
**Clustering Variables**								
Age	52	61	69	73	68	77	58	68
Median, (IQR)	35-65	49-71	57-77	65-80	56-76	68-84	49.75-70.25	55-77
Sex								
-Female	3760 (74.22)	1420 (75.77)	4725 (69.77)	2856 (33.02)	1626 (28.61)	782 (63.01)	105 (62.5)	15274 (51.86)
-Male	1306 (25.78)	454 (24.23)	2047 (30.23)	5793 (66.98)	4058 (71.39)	459 (36.99)	63 (37.5)	14180 (48.14)
Primary Care (GP) and Community contacts	4	6	4	5	4	54	10	5
Median, (IQR)	2-9	2.25-13	2-8	2-9	1-7	29-87	5-20.25	2-10
Mental health attendances	0	0	0	0	0	0	46	0
Median, (IQR)	0-0	0-0	0-0	0-0	0-0	0-0	34-62.5	0-0
Secondary care elective (outpatient and elective in patient appointments)	5	42	6	3	11	10	6	6
Median, (IQR)	1-11	32-54	3-12	0-7	5-17	5-19	2-13	2-14
Secondary care non-elective (accident and emergency and non-elective in patient appointments)	0	1	0	0	0	6	2	0
Median, (IQR)	0-2	0-3	0-1	0-1	0-1	3-9	0-3	0-2
Cardiovascular condition (current)	449 (8.86)	209 (11.15)	1082 (15.98)	2492 (28.81)	923 (16.24)	567 (45.69)	27 (16.07)	5749 (19.52)
Cancer diagnosed in the past 5 years	260 (5.13)	1397 (74.55)	198 (2.92)	360 (4.16)	4641 (81.65)	200 (16.12)	25 (14.88)	7081 (24.04)
Mental Health	1131 (22.33)	311 (16.6)	1034 (15.27)	1417 (16.38)	661 (11.63)	286 (23.05)	132 (78.57)	4972 (16.88)
Diabetes	734 (14.49)	259 (13.82)	1368 (20.2)	1959 (22.65)	1108 (19.49)	387 (31.18)	39 (23.21)	5854 (19.88)
Dementia	39 (0.77)	8 (0.43)	190 (2.81)	337 (3.9)	81 (1.43)	104 (8.38)	18 (10.71)	777 (2.64)
Asthma	3969 (78.35)	186 (9.93)	492 (7.27)	1331 (15.39)	240 (4.22)	297 (23.93)	54 (32.14)	6569 (22.3)
COPD	1076 (21.24)	134 (7.15)	400 (5.91)	7617 (88.07)	247 (4.35)	739 (59.55)	76 (45.24)	10289 (34.93)
Drugs that require monitoring	644 (12.71)	185 (9.87)	5330 (78.71)	404 (4.67)	241 (4.24)	229 (18.45)	67 (39.88)	7100 (24.11)
Smoking								
-Non smoker	4204 (82.98)	1691 (90.23)	6105 (90.15)	6442 (74.48)	5066 (89.13)	1056 (85.09)	106 (63.1)	24670 (83.76)
-Current smoker	862 (17.02)	183 (9.77)	667 (9.85)	2207 (25.52)	618 (10.87)	185 (14.91)	62 (36.9)	4784 (16.24)
**Non Clustering Variables**								
Urban/Rural								
-Rural town and fringe	125 (2.47)	60 (3.2)	227 (3.35)	226 (2.61)	191 (3.36)	38 (3.06)	6 (3.57)	873 (2.96)

For peer review only

For peer review only - http://bmjopen.bmj.com/site/about/guidelines.xhtml

-Rural village and dispersed	200 (3.95)	124 (6.62)	399 (5.89)	339 (3.92)	320 (5.63)	33 (2.66)	5 (2.98)	1420 (4.82)
-Urban city and town	4741 (93.58)	1690 (90.18)	6146 (90.76)	8084 (93.47)	5173 (91.01)	1170 (94.28)	157 (93.45)	27161 (92.21)
IMD Decile	5	7	7	5	7	5	4	6
Median, (IQR)	3-8	4-9	4-9	2-8	4-9	3-8	2-7	3-8
Learning disabilities and autism	100 (1.97)	9 (0.48)	30 (0.44)	31 (0.36)	34 (0.6)	19 (1.53)	7 (4.17)	230 (0.78)
Housebound	59 (1.16)	19 (1.01)	181 (2.67)	396 (4.58)	86 (1.51)	321 (25.87)	19 (11.31)	1081 (3.67)
Has a carer	75 (1.48)	40 (2.13)	146 (2.16)	340 (3.93)	107 (1.88)	127 (10.23)	10 (5.95)	845 (2.87)
Is a carer	161 (3.18)	64 (3.42)	266 (3.93)	331 (3.83)	169 (2.97)	63 (5.08)	3 (1.79)	1057 (3.59)
Charlson Score	2	4	4	5	5	6	3	4
Median, (IQR)	(1-4)	(2-6)	(2-5)	(4-6)	(3-6)	(4-7)	(2-5)	(3-6)

Table C.4: Segmentation of high-risk cohort (*n*=29,454) into 7 clusters.

For peer review only - http://bmjopen.bmj.com/site/about/guidelines.xhtml

8 Clusters

Cluster	Cluster 1	Cluster 2	Cluster 3	Cluster 4	Cluster 5	Cluster 6	Cluster 7	Cluster 8	Total Population
**Total**	**5853**	**1925**	**1038**	**5917**	**4893**	**172**	**4113**	**5543**	**29,454**
**Proportion of population**	**19.87%**	**6.54%**	**3.52%**	**20.09%**	**16.61%**	**0.58%**	**13.96%**	**18.82%**	**100.00%**
**Clustering Variables**									
Age	74	60	77	64	72	58	39	73	68
Median, (IQR)	67-81	49-71	68-84	54-73	63-79	49.75-70.25	26-49	65-80	55-77
Sex									
-Female	5853 (100)	1503 (78.08)	623 (60.02)	4358 (73.65)	1558 (31.84)	106 (61.63)	1273 (30.95)	0 (0)	15274 (51.86)
-Male	0 (0)	422 (21.92)	415 (39.98)	1559 (26.35)	3335 (68.16)	66 (38.37)	2840 (69.05)	5543 (100)	14180 (48.14)
Primary Care (GP) and Community contacts	6	7	61	4	4	10	3	4	5
Median, (IQR)	2-12	3-12	34-94.75	2-8	2-8	5-20.25	1-6	2-9	2-10
Mental health attendances	0	0	0	0	0	45	0	0	0
Median, (IQR)	0-0	0-0	0-0	0-0	0-0	32.75-62	0-0	0-0	0-0
Secondary care elective (outpatient and elective in patient appointments)	3	41	10.5	7	11	6	6	3	6
Median, (IQR)	0-8	31-53	5-19	3-13	6-18	2-13	2-12	0-7	2-14
Secondary care non-elective (accident and emergency and non-elective in patient appointments)	0	1	6	0	0	2	0	0	0
Median, (IQR)	0-2	0-3	3-10	0-1	0-1	0-3	0-2	0-1	0-2
Cardiovascular condition (current)	1307 (22.33)	202 (10.49)	566 (54.53)	798 (13.49)	884 (18.07)	28 (16.28)	246 (5.98)	1718 (30.99)	5749 (19.52)
Cancer diagnosed in the past 5 years	284 (4.85)	1435 (74.55)	169 (16.28)	189 (3.19)	4459 (91.13)	25 (14.53)	327 (7.95)	193 (3.48)	7081 (24.04)
Mental Health	1135 (19.39)	344 (17.87)	241 (23.22)	1084 (18.32)	473 (9.67)	136 (79.07)	740 (17.99)	819 (14.78)	4972 (16.88)
Diabetes	1112 (19)	280 (14.55)	340 (32.76)	1192 (20.15)	881 (18.01)	40 (23.26)	678 (16.48)	1331 (24.01)	5854 (19.88)
Dementia	276 (4.72)	8 (0.42)	88 (8.48)	90 (1.52)	88 (1.8)	18 (10.47)	4 (0.1)	205 (3.7)	777 (2.64)
Asthma	1698 (29.01)	257 (13.35)	249 (23.99)	1347 (22.76)	418 (8.54)	55 (31.98)	1304 (31.7)	1241 (22.39)	6569 (22.3)
COPD	4346 (74.25)	116 (6.03)	584 (56.26)	199 (3.36)	224 (4.58)	79 (45.93)	22 (0.53)	4719 (85.13)	10289 (34.93)
Drugs that require monitoring	247 (4.22)	174 (9.04)	210 (20.23)	4975 (84.08)	243 (4.97)	67 (38.95)	784 (19.06)	400 (7.22)	7100 (24.11)
Smoking									
-Non smoker	4573 (78.13)	1741 (90.44)	887 (85.45)	5244 (88.63)	4464 (91.23)	108 (62.79)	3453 (83.95)	4200 (75.77)	24670 (83.76)
-Current smoker	1280 (21.87)	184 (9.56)	151 (14.55)	673 (11.37)	429 (8.77)	64 (37.21)	660 (16.05)	1343 (24.23)	4784 (16.24)
**Non Clustering Variables**									
Urban/Rural									

For peer review only

For peer review only - http://bmjopen.bmj.com/site/about/guidelines.xhtml

-Rural town and fringe	143 (2.44)	60 (3.12)	35 (3.37)	190 (3.21)	183 (3.74)	6 (3.49)	93 (2.26)	163 (2.94)	873 (2.96)
-Rural village and dispersed	239 (4.08)	123 (6.39)	30 (2.89)	332 (5.61)	304 (6.21)	5 (2.91)	145 (3.53)	242 (4.37)	1420 (4.82)
-Urban city and town	5471 (93.47)	1742 (90.49)	973 (93.74)	5395 (91.18)	4406 (90.05)	161 (93.6)	3875 (94.21)	5138 (92.69)	27161 (92.21)
IMD Decile<br>Median, (IQR)	5<br>2-8	7<br>4-9	5<br>3-8	6<br>4-9	7<br>4-9	4<br>2-7	5<br>3-8	5<br>2-8	6<br>3-8
Learning disabilities and autism	9 (0.15)	8 (0.42)	15 (1.45)	28 (0.47)	12 (0.25)	7 (4.07)	125 (3.04)	26 (0.47)	230 (0.78)
Housebound	363 (6.2)	19 (0.99)	283 (27.26)	79 (1.34)	90 (1.84)	19 (11.05)	19 (0.46)	209 (3.77)	1081 (3.67)
Has a carer	230 (3.93)	40 (2.08)	111 (10.69)	89 (1.5)	113 (2.31)	10 (5.81)	36 (0.88)	216 (3.9)	845 (2.87)
Is a carer	289 (4.94)	68 (3.53)	53 (5.11)	225 (3.8)	168 (3.43)	4 (2.33)	60 (1.46)	190 (3.43)	1057 (3.59)
Charlson Score<br>Median, (IQR)	5<br>(4-6)	4<br>(2-6)	6<br>(5-7)	3<br>(2-5)	5<br>(4-7)	3<br>(2-5)	1<br>(0-2)	5<br>(4-6)	4<br>(3-6)

Table C.5: Segmentation of high-risk cohort (*n*=29,454) into 8 clusters.

For peer review only

9 Clusters

Variables	Cluster 1	Cluster 2	Cluster 3	Cluster 4	Cluster 5	Cluster 6	Cluster 7	Cluster 8	Cluster 9	Total Population
**Total**	**170**	**1744**	**3028**	**990**	**4890**	**3483**	**4857**	**4815**	**5477**	**29,454**
**Proportion of population**	**0.58%**	**5.92%**	**10.28%**	**3.36%**	**16.60%**	**11.83%**	**16.49%**	**16.35%**	**18.60%**	**100.00%**
**Clustering Variables**										
Age	58	61	35	77	68	56	75	71	73	68
Median, (IQR)	50.25-70	49-71	22-45	68-85	58-75	48-65	69-82	63-78	65-79	55-77
Sex										
-Female	105 (61.76)	1332 (76.38)	720 (23.78)	583 (58.89)	3400 (69.53)	2774 (79.64)	4857 (100)	1503 (31.21)	0 (0)	15274 (51.86)
-Male	65 (38.24)	412 (23.62)	2308 (76.22)	407 (41.11)	1490 (30.47)	709 (20.36)	0 (0)	3312 (68.79)	5477 (100)	14180 (48.14)
Primary Care (GP) and Community contacts	10	6	2	64	4	5	6	4	4	5
Median, (IQR)	5-20	3-13	1-5	36-96	2-8	2-11	2-12	2-8	1-9	2-10
Mental health attendances	45.5	0	0	0	0	0	0	0	0	0
Median, (IQR)	34-62	0-0	0-0	0-0	0-0	0-0	0-0	0-0	0-0	0-0
Secondary care elective (outpatient and elective in patient appointments)	6	43	6	11	7	5	3	12	3	6
Median, (IQR)	2-13	33-55	2-13	5-20	4-13	1-11	0-8	6-18	0-7	2-14
Secondary care non-elective (accident and emergency and non-elective in patient appointments)	2	1	0	6	0	0	0	0	0	0
Median, (IQR)	0-3	0-3	0-2	3-10	0-1	0-2	0-2	0-1	0-1	0-2
Cardiovascular condition (current)	27 (15.88)	187 (10.72)	172 (5.68)	542 (54.75)	763 (15.6)	335 (9.62)	1161 (23.9)	864 (17.94)	1698 (31)	5749 (19.52)
Cancer diagnosed in the past 5 years	25 (14.71)	1320 (75.69)	312 (10.3)	169 (17.07)	186 (3.8)	202 (5.8)	262 (5.39)	4409 (91.57)	196 (3.58)	7081 (24.04)
Mental Health	134 (78.82)	295 (16.92)	460 (15.19)	229 (23.13)	783 (16.01)	904 (25.95)	880 (18.12)	461 (9.57)	826 (15.08)	4972 (16.88)
Diabetes	39 (22.94)	242 (13.88)	476 (15.72)	331 (33.43)	1033 (21.12)	610 (17.51)	927 (19.09)	866 (17.99)	1330 (24.28)	5854 (19.88)
Dementia	18 (10.59)	5 (0.29)	2 (0.07)	86 (8.69)	92 (1.88)	29 (0.83)	256 (5.27)	89 (1.85)	200 (3.65)	777 (2.64)
Asthma	54 (31.76)	175 (10.03)	399 (13.18)	227 (22.93)	396 (8.1)	3192 (91.65)	807 (16.62)	231 (4.8)	1088 (19.86)	6569 (22.3)
COPD	78 (45.88)	112 (6.42)	22 (0.73)	562 (56.77)	175 (3.58)	531 (15.25)	3860 (79.47)	221 (4.59)	4728 (86.32)	10289 (34.93)
Drugs that require monitoring	67 (39.41)	169 (9.69)	763 (25.2)	203 (20.51)	4488 (91.78)	545 (15.65)	222 (4.57)	243 (5.05)	400 (7.3)	7100 (24.11)
Smoking										
-Non smoker	107 (62.94)	1577 (90.42)	2589 (85.5)	843 (85.15)	4391 (89.8)	2855 (81.97)	3793 (78.09)	4388 (91.13)	4127 (75.35)	24670 (83.76)
-Current smoker	63 (37.06)	167 (9.58)	439 (14.5)	147 (14.85)	499 (10.2)	628 (18.03)	1064 (21.91)	427 (8.87)	1350 (24.65)	4784 (16.24)
**Non Clustering Variables**										
Urban/Rural										

For peer review only - http://bmjopen.bmj.com/site/about/guidelines.xhtml

-Rural town and fringe	6 (3.53)	56 (3.21)	72 (2.38)	34 (3.43)	164 (3.35)	91 (2.61)	113 (2.33)	177 (3.68)	160 (2.92)	873 (2.96)
-Rural village and dispersed	5 (2.94)	118 (6.77)	108 (3.57)	27 (2.73)	295 (6.03)	143 (4.11)	200 (4.12)	294 (6.11)	230 (4.2)	1420 (4.82)
-Urban city and town	159 (93.53)	1570 (90.02)	2848 (94.06)	929 (93.84)	4431 (90.61)	3249 (93.28)	4544 (93.56)	4344 (90.22)	5087 (92.88)	27161 (92.21)
IMD Decile Median, (IQR)	4<br>2-7	7<br>4-9	5<br>3-8	5<br>3-8	7<br>4-9	5<br>3-8	5<br>3-8	7<br>4-9	5<br>2-8	6<br>3-8
Learning disabilities and autism	7 (4.12)	8 (0.46)	110 (3.63)	15 (1.52)	19 (0.39)	27 (0.78)	6 (0.12)	12 (0.25)	26 (0.47)	230 (0.78)
Housebound	19 (11.18)	17 (0.97)	13 (0.43)	276 (27.88)	73 (1.49)	45 (1.29)	342 (7.04)	90 (1.87)	206 (3.76)	1081 (3.67)
Has a carer	10 (5.88)	38 (2.18)	27 (0.89)	109 (11.01)	78 (1.6)	54 (1.55)	202 (4.16)	112 (2.33)	215 (3.93)	845 (2.87)
Is a carer	3 (1.76)	59 (3.38)	35 (1.16)	53 (5.35)	196 (4.01)	124 (3.56)	236 (4.86)	163 (3.39)	188 (3.43)	1057 (3.59)
Charlson Score Median, (IQR)	3 (2-5)	4 (2-6)	1 (0-2)	6 (5-7)	4 (2-5)	3 (2-4)	5 (4-6)	5 (4-7)	5 (4-6)	4 (3-6)

Table C.6: Segmentation of high-risk cohort (*n*=29,454) into 9 clusters.

For peer review only

For peer review only - http://bmjopen.bmj.com/site/about/guidelines.xhtml

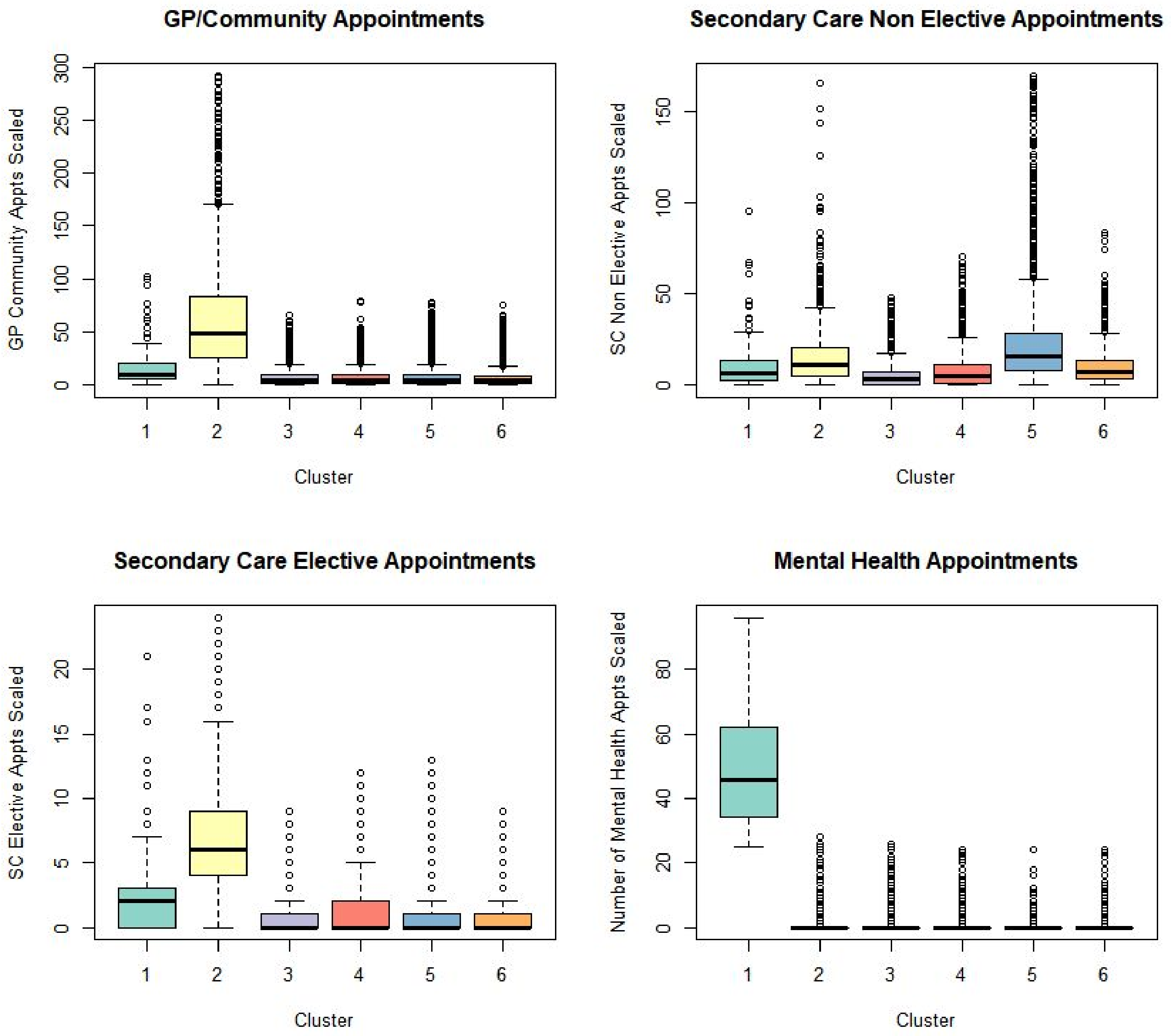

only

For peer review only - http://bmjopen.bmj.com/site/about/guidelines.xhtml

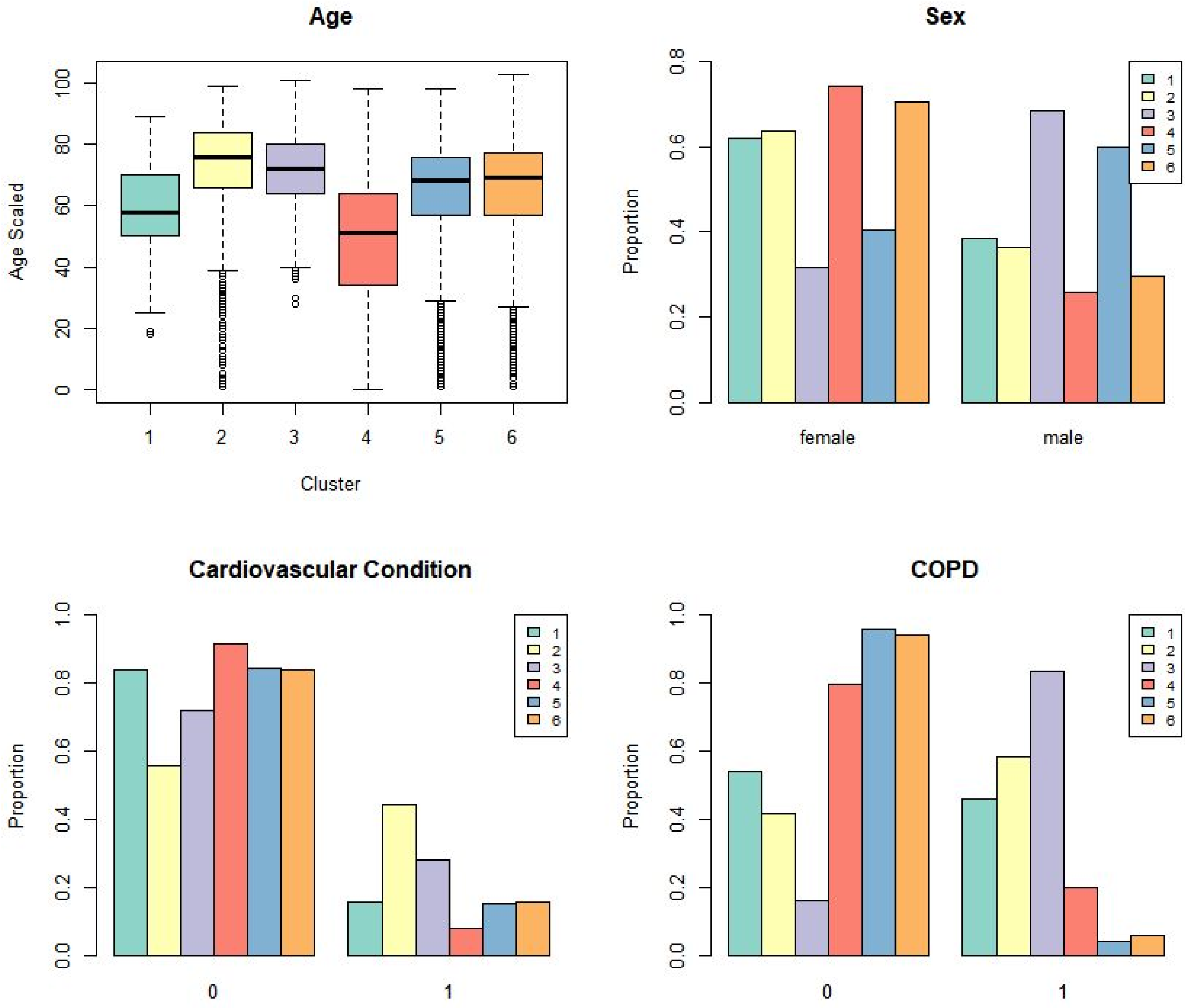

For peer review only - http://bmjopen.bmj.com/site/about/guidelines.xhtml

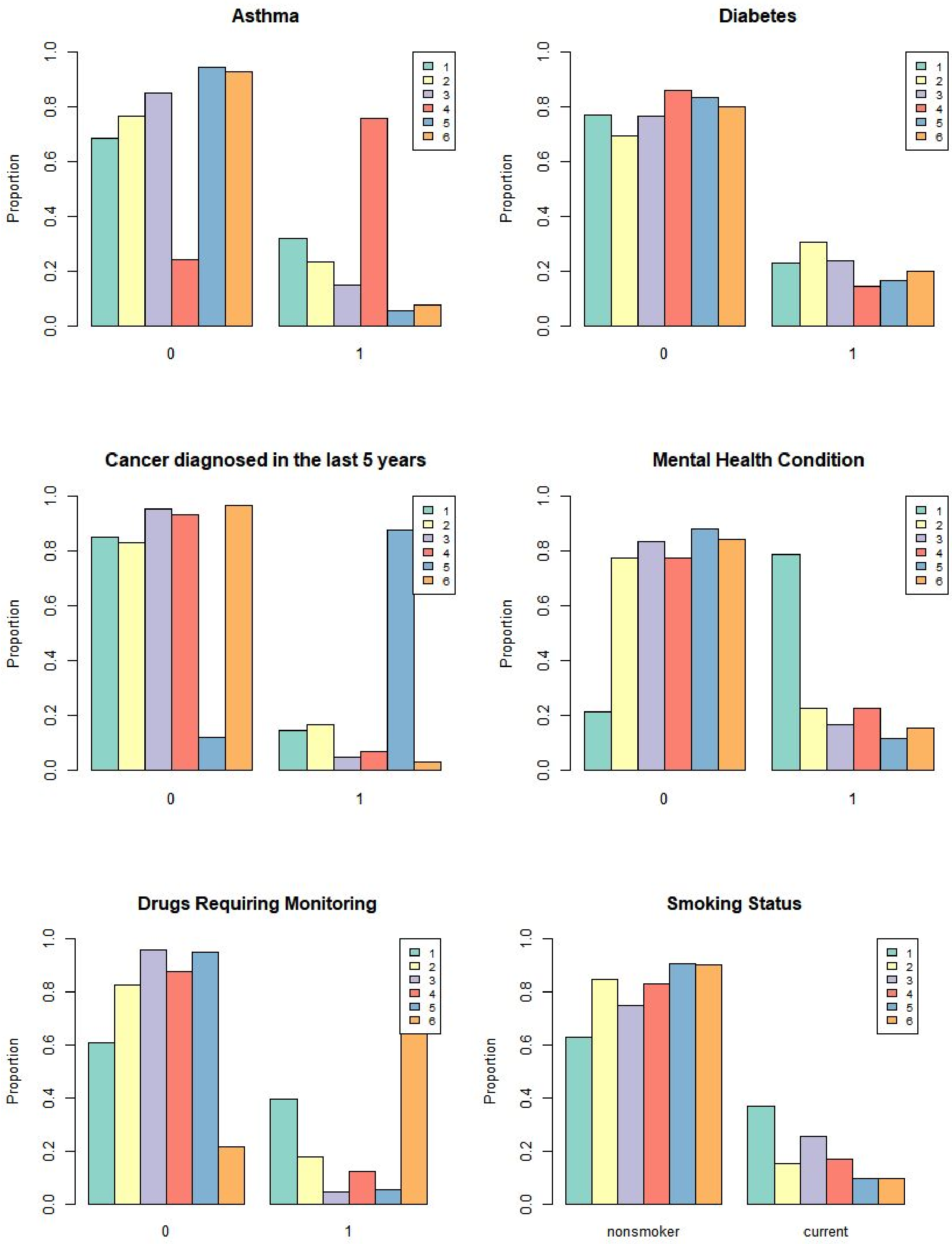

Figure C.2: Cluster profiles produced for the 6 cluster solution to aid interpretability and differentiation of segments. The proportions are shown for binary variables and boxplots for continuous variables.

For peer review only - http://bmjopen.bmj.com/site/about/guidelines.xhtml

References for Supplementary Material B:

1. R Core Team. R: A language and environment for statistical computing. R Foundation for Statistical Computing, Vienna, Austria. 2019
2. Szepannek G. clustMixType: User-Friendly Clustering of Mixed-Type Data in R. *The R Journal* 2018;10(2)
3. Chong JL, Lim KK, Matchar DB. Population segmentation based on healthcare needs: a systematic review. *Syst Rev* 2019;8(1):202. doi: 10.1186/s13643-019-1105-6 [published Online First: 2019/08/15]
4. Wood RM, Murch BJ, Betteridge RC. A comparison of population segmentation methods. *Operations Research for Health Care* 2019
5. Yan S, Kwan YH, Tan CS, et al. A systematic review of the clinical application of data-driven population segmentation analysis. *BMC Med Res Methodol* 2018;18(1):121. doi: 10.1186/s12874-018-0584-9 [published Online First: 2018/11/06]
6. Szepannek GA, R. clustMixType: k-Prototypes Clustering for Mixed Variable-Type Data. April 23 2020. *The Comprehensive R Archive Network* 2020
7. Huang Z. Extensions to the k-Means Algorithm for Clustering Large Data Sets with Categorical Values. *Data Mining and Knowledge Discovery* 1998;2(3):283-304. doi: 10.1023/A:1009769707641
8. Li C, Wu X, Cheng X, et al. Identification and analysis of vulnerable populations for malaria based on K-prototypes clustering. *Environmental Research* 2019;176:108568. doi: https://doi.org/10.1016/j.envres.2019.108568
9. Aldenderfer MS, Blashfield RK. Cluster Analysis. California: SAGE Publications Inc 1984.
10. Clatworthy J, Buick D, Hankins M, et al. The use and reporting of cluster analysis in health psychology: a review. *Br J Health Psychol* 2005;10(Pt 3):329-58. doi: 10.1348/135910705x25697 [published Online First: 2005/10/22]
11. Rousseeuw PJ. Silhouettes: A graphical aid to the interpretation and validation of cluster analysis. *Journal of Computational and Applied Mathematics* 1987;20:53-65. doi: https://doi.org/10.1016/0377-0427(87)90125-7
12. Yoon S, Goh H, Kwan YH, et al. Identifying optimal indicators and purposes of population segmentation through engagement of key stakeholders: a qualitative study. *Health Res Policy Syst* 2020;18(1):26. doi: 10.1186/s12961-019-0519-x [published Online First: 2020/02/23]
13. Vuik SI, Mayer E, Darzi A. A quantitative evidence base for population health: applying utilization-based cluster analysis to segment a patient population. *Popul Health Metr* 2016;14:44. doi: 10.1186/s12963-016-0115-z [published Online First: 2016/12/03]
14. Charlson M, Szatrowski TP, Peterson J, et al. Validation of a combined comorbidity index. *J Clin Epidemiol* 1994;47(11):1245-51. doi: 10.1016/0895-4356(94)90129-5 [published Online First: 1994/11/01]
15. Vuik SI, Mayer E, Darzi A. Enhancing risk stratification for use in integrated care: a cluster analysis of high-risk patients in a retrospective cohort study. *BMJ Open* 2016;6(12):e012903. doi: 10.1136/bmjopen-2016-012903
16. Everitt BS, Landau S, Leese M, et al. Cluster analysis. 5th ed. Chichester: John Wiley & Sons 2011.

For peer review only - http://bmjopen.bmj.com/site/about/guidelines.xhtml

Supplementary Material D: Comparison of the low, moderate and high risk groups

Age Group	Female			Male		
	% at high risk in this group	% at moderate risk in this group	% at low risk in this group	% at high risk in this group	% at moderate risk in this group	% at low risk in this group
0-4	0.3	43	56.8	0.3	42.7	57
5-9	0.3	5.8	93.9	0.4	7.3	92.3
10-14	0.4	8.2	91.4	0.4	10.1	89.6
15-19	0.4	9.7	89.8	0.4	9.4	90.2
20-24	0.4	11.6	88	0.4	8.4	91.2
25-29	0.6	15.8	83.6	0.5	9.4	90
30-34	0.9	18.8	80.3	0.5	9.8	89.6
35-39	1.2	19.4	79.5	0.8	12	87.2
40-44	1.6	20	78.4	1.2	15	83.7
45-49	2.4	23.6	74	1.7	19.4	78.9
50-54	3.3	27	69.8	2.5	23.7	73.8
55-59	4.3	30.8	64.8	3.9	29.7	66.4
60-64	6.1	35.8	58.1	5.6	36.9	57.5
65-69	7.6	92.3	0.1	7.9	92	0.1
70-74	10.3	89.5	0.1	11	88.8	0.1
75-79	10.5	89.4	0.2	13.3	86.4	0.3
80-84	12.2	87.5	0.3	14.5	85.1	0.4
85-89	10.8	88.5	0.6	14	85.2	0.8
90-94	8.3	90.4	1.3	11.7	86.6	1.7
95+	5.3	92.1	2.6	11	86.3	2.7

Table D.1: Percentage of high, moderate and low risk individuals by age group and sex.

For peer review only

For peer review only - http://bmjopen.bmj.com/site/about/guidelines.xhtml

Plots comparing the distribution of age and Charlson Score for low, moderate and high risk groups

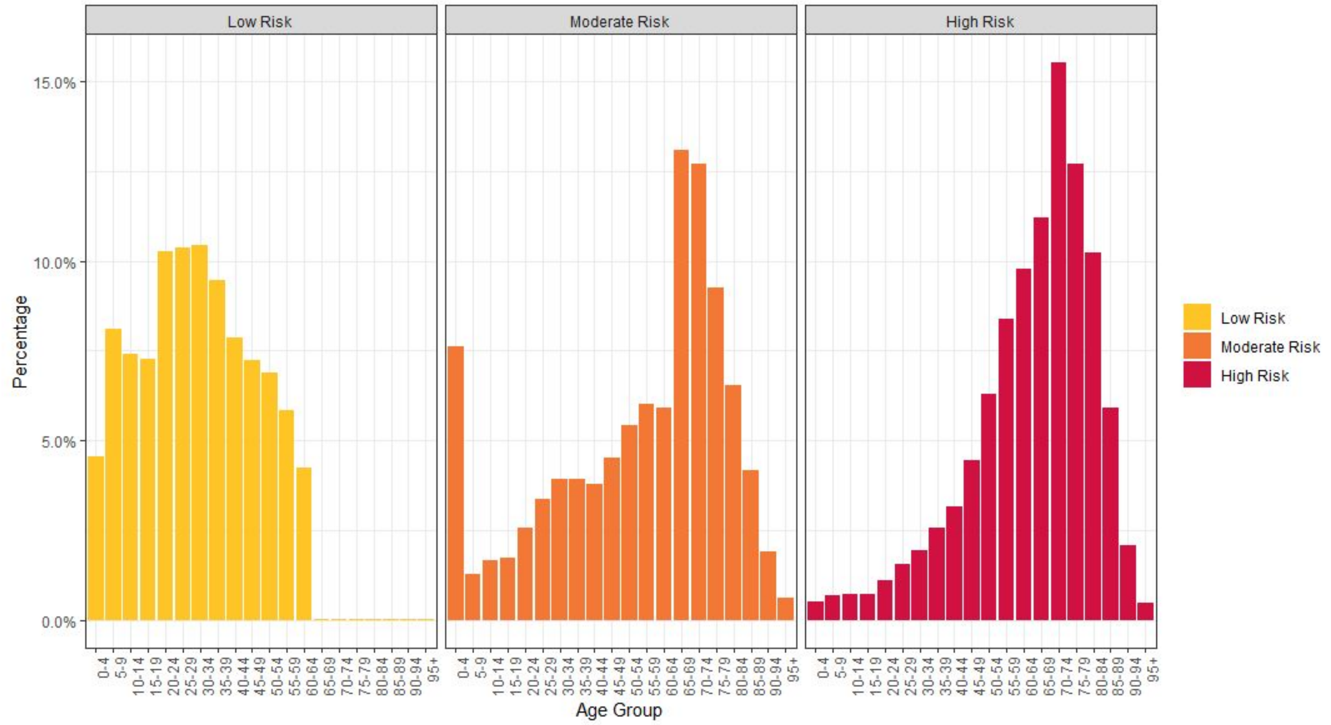

Figure D.1: Distribution of age groups by high, moderate and low risk groups.

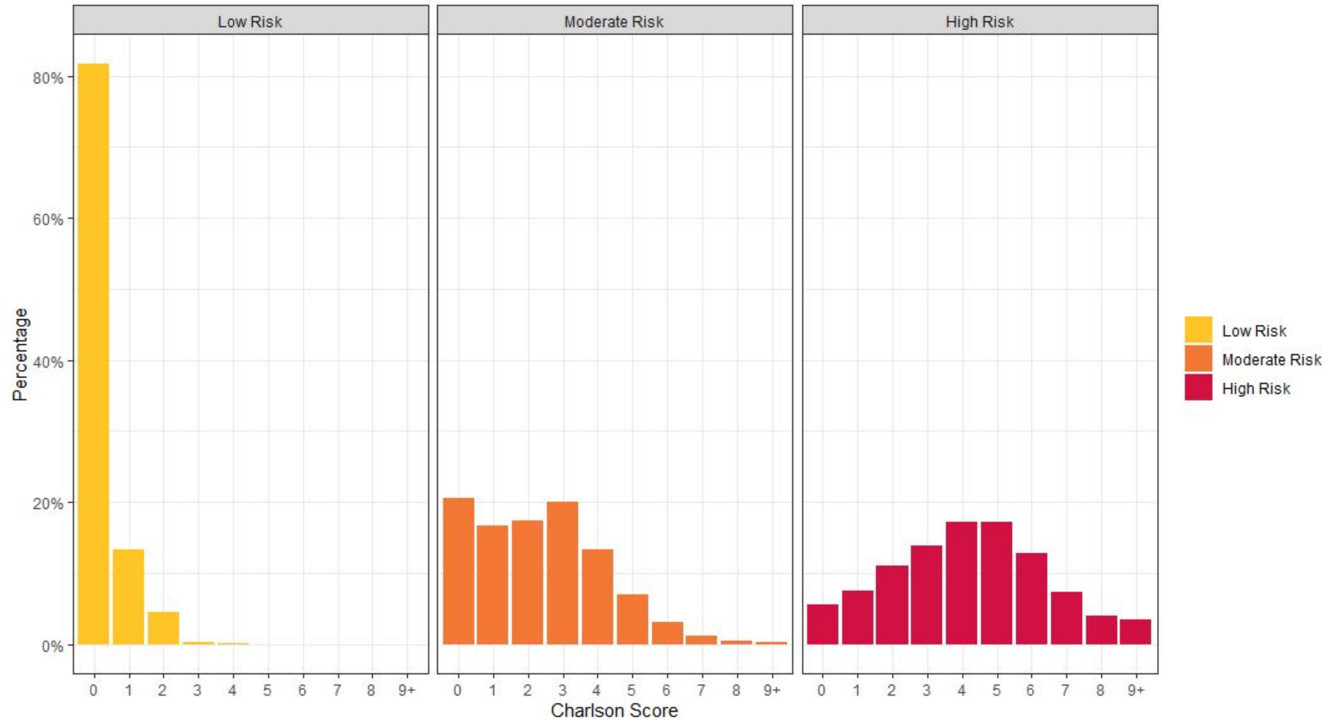

Figure D.2: Distribution of Charlson Score by high, moderate and low risk groups.

For peer review only - http://bmjopen.bmj.com/site/about/guidelines.xhtml

Supplementary Material A: RECORD Statement

The RECORD statement – checklist of items, extended from the STROBE statement, that should be reported in observational studies using routinely collected health data.

	Item No.	STROBE items	Location in manuscript where items are reported	RECORD items	Location in manuscript where items are reported
**Title and abstract**					
	1	(a) Indicate the study's design with a commonly used term in the title or the abstract (b) Provide in the abstract an informative and balanced summary of what was done and what was found	Page 1 in title and abstract	RECORD 1.1: The type of data used should be specified in the title or abstract. When possible, the name of the databases used should be included.<br><br>RECORD 1.2: If applicable, the geographic region and timeframe within which the study took place should be reported in the title or abstract.<br><br>RECORD 1.3: If linkage between databases was conducted for the study, this should be clearly stated in the title or abstract.	1.1 Page 1 in the abstract.<br><br>1.2 Page 1 in the abstract.<br><br>1.3 Page 1 – data were linked in a system wide dataset.
**Introduction**					
Background rationale	2	Explain the scientific background and rationale for the investigation being reported	Page 3 under Introduction		
Objectives	3	State specific objectives, including any prespecified hypotheses	Page 3 at the bottom of the page.		
**Methods**					
Study Design	4	Present key elements of study design early in the paper	Page 4 under 2.1 Data, application and setting.		
Setting	5	Describe the setting, locations, and relevant dates, including periods of recruitment, exposure, follow-up, and data collection	Page 4		
Participants	6	*(a) Cohort study* - Give the eligibility criteria, and the sources and methods of selection of participants. Describe methods of follow-up<br>*Case-control study* - Give the eligibility criteria, and the sources and methods of case ascertainment and control selection. Give the rationale for the choice of cases and controls<br>*Cross-sectional study* - Give the eligibility criteria, and the sources and methods of selection of participants	Cross-sectional study – page 4 gives eligibility criteria and sources/method of selection.	RECORD 6.1: The methods of study population selection (such as codes or algorithms used to identify subjects) should be listed in detail. If this is not possible, an explanation should be provided.<br><br>RECORD 6.2: Any validation studies of the codes or algorithms used to select the population should be referenced. If validation was conducted for this study and not published elsewhere, detailed methods and results should be provided.<br><br>RECORD 6.3: If the study involved linkage of databases, consider use of a flow diagram or other graphical display to demonstrate the data linkage process, including the number of individuals with linked data at each stage.	In this case all individuals within BNSSG registered to a contributing practice were included for analyses. Therefore codes to identify the overall study population were not used.<br><br>For the high-risk group population selection, all codes and criteria used are listed fully in Supplementary Material B and the methods described in section 2.2 (page 4).<br><br>Furthermore, the resulting list was compared with estimated expected numbers of high-risk individuals described in the Discussion under interpretation page 11.<br><br>The system-wide dataset at BNSSG CCG is already linked for analyses but a reference is included providing further information to the dataset

		(b) Cohort study - For matched studies, give matching criteria and number of exposed and unexposed<br>*Case-control study* - For matched studies, give matching criteria and the number of controls per case			(Reference 25) and linking through a pseudonymised ID (unique individual identifier) was mentioned on page 4.
Variables	7	Clearly define all outcomes, exposures, predictors, potential confounders, and effect modifiers. Give diagnostic criteria, if applicable.	All outcomes and variables are fully defined including the use of algorithms and coding lists in Supplementary Material B for the high-risk criteria and Supplementary Material C for all clustering and non-clustering variables. Reference to the Supplementary material included on page 5 of the methods.	RECORD 7.1: A complete list of codes and algorithms used to classify exposures, outcomes, confounders, and effect modifiers should be provided. If these cannot be reported, an explanation should be provided.	All outcomes and variables are fully defined including the use of algorithms and coding lists in Supplementary Material B for the high-risk criteria and Supplementary Material C for all clustering and non-clustering variables.
Data sources/ measurement	8	For each variable of interest, give sources of data and details of methods of assessment (measurement). Describe comparability of assessment methods if there is more than one group	Table C.3 in Supplementary Material C.		
Bias	9	Describe any efforts to address potential sources of bias	To minimise misclassification and information bias, high risk criteria and coding lists were subject to double clinical review (page 5 Methods). The discussion page 10,11 discusses potential information bias as a limitation of using routine datasets.		
Study size	10	Explain how the study size was arrived at	Page 4 and 5		
Quantitative variables	11	Explain how quantitative variables were handled in the analyses. If applicable, describe which groupings were chosen, and why	How the variables were operationalised for analyses is detailed in Table C.3 Supplementary Material C.		
Statistical methods	12	(a) Describe all statistical methods, including those used to control for confounding<br>(b) Describe any methods used to examine subgroups and interactions<br>(c) Explain how missing data were addressed	a) Section 2.3 page 5. Further details on cluster analysis and selection of the number of clusters is included in Supplementary Material C.<br><br>b) Subgroups were investigated in Table		

		(d) *Cohort study* - If applicable, explain how loss to follow-up was addressed<br>*Case-control study* - If applicable, explain how matching of cases and controls was addressed<br>*Cross-sectional study* - If applicable, describe analytical methods taking account of sampling strategy<br>(e) Describe any sensitivity analyses	1. Methods described on page 5.<br><br>c) Page 5<br><br>d) NA. The full BNSSG cohort from March 2019 was taken for analyses.<br><br>e) Sensitivity analyses in terms of the clustering solution chosen for segmentation is fully described in Supplementary Material C. Also described more briefly in the methods page 5.		
Data access and cleaning methods		..		RECORD 12.1: Authors should describe the extent to which the investigators had access to the database population used to create the study population.<br><br>RECORD 12.2: Authors should provide information on the data cleaning methods used in the study.	The full BNSSG database population for the SWD were used for analyses. This is included on page 4.<br><br>How variables were coded and operationalised is documented in Supplementary Material B and C and page 4 describes how a unique identifier is used to link data between the tables in the system wide dataset.
Linkage		..		RECORD 12.3: State whether the study included person-level, institutional-level, or other data linkage across two or more databases. The methods of linkage and methods of linkage quality evaluation should be provided.	Page 4 under section 2.1.
**Results**					
Participants	13	(a) Report the numbers of individuals at each stage of the study (*e.g.*, numbers potentially eligible, examined for eligibility, confirmed eligible, included in the study, completing follow-up, and analysed)<br>(b) Give reasons for non-participation at each stage.<br>(c) Consider use of a flow diagram	Page 6 for descriptive analyses.<br>Page 8 for cluster analysis.	RECORD 13.1: Describe in detail the selection of the persons included in the study (*i.e.,* study population selection) including filtering based on data quality, data availability and linkage. The selection of included persons can be described in the text and/or by means of the study flow diagram.	Page 6 for descriptive analyses.<br>Page 8 for cluster analysis.
Descriptive data	14	(a) Give characteristics of study participants (*e.g.*, demographic, clinical, social) and information on exposures and potential confounders<br>(b) Indicate the number of participants with missing data for	Page 6 for descriptive analyses.<br>Page 8 for cluster analysis.<br><br>Number of participants with missing data page 8.		

For peer review only

		each variable of interest<br>(c) *Cohort study* - summarise follow-up time (*e.g.*, average and total amount)			
Outcome data	15	*Cohort study* - Report numbers of outcome events or summary measures over time<br>*Case-control study* - Report numbers in each exposure category, or summary measures of exposure<br>*Cross-sectional study* - Report numbers of outcome events or summary measures	*Cross-sectional study* – Page 6, Table 1, Figure 1 and 2 report a comparative analysis of the low, moderate and high-risk groups with regard to demographic and socioeconomic information, historical healthcare utilisation, and burden of comorbidity. Figure 3 shows the geographical distribution of high risk individuals.		
Main results	16	(a) Give unadjusted estimates and, if applicable, confounder-adjusted estimates and their precision (e.g., 95% confidence interval). Make clear which confounders were adjusted for and why they were included<br>(b) Report category boundaries when continuous variables were categorized<br>(c) If relevant, consider translating estimates of relative risk into absolute risk for a meaningful time period	a) Main descriptive analyses reported on pages 6-8. Cluster analysis results reported on page 8-10.<br><br>b) Continuous variables kept as continuous and results reported as mean, median with interquartile range or proportion. Tables 1 and 2. Supplementary Material C discusses how variables are operationalised.<br><br>c) Not applicable.		
Other analyses	17	Report other analyses done—e.g., analyses of subgroups and interactions, and sensitivity analyses	Results are presented for low, moderate and high risk subgroups and for the segmentation analysis for clusters 1-6. Pages 6-8 for descriptive analyses and page 8-10 for cluster analyses. Sensitivity analyses in terms of the clustering solution were performed and documented in Supplementary Material C.		
**Discussion**					
Key results	18	Summarise key results with reference to study objectives	Page 10.		
Limitations	19	Discuss limitations of the study, taking into account sources of potential bias or imprecision. Discuss both direction and magnitude of any potential bias	Page 10-11.	RECORD 19.1: Discuss the implications of using data that were not created or collected to answer the specific research question(s). Include discussion of misclassification bias, unmeasured confounding, missing data, and changing eligibility over time, as they	Page 10-12 (i.e. misclassification bias and limitations relating to the data).

For peer review only

				pertain to the study being reported.	
Interpretation	20	Give a cautious overall interpretation of results considering objectives, limitations, multiplicity of analyses, results from similar studies, and other relevant evidence	Page 11-12.		
Generalisability	21	Discuss the generalisability (external validity) of the study results	Page 10-12.		
**Other Information**					
Funding	22	Give the source of funding and the role of the funders for the present study and, if applicable, for the original study on which the present article is based	Page 13.		
Accessibility of protocol, raw data, and programming code		..		RECORD 22.1: Authors should provide information on how to access any supplemental information such as the study protocol, raw data, or programming code.	Page 13. Technical appendix and coding lists available in online supplementary files. Local data used for the study are not publicly available.

*Reference: Benchimol EI, Smeeth L, Guttmann A, Harron K, Moher D, Petersen I, Sørensen HT, von Elm E, Langan SM, the RECORD Working Committee. The REporting of studies Conducted using Observational Routinely-collected health Data (RECORD) Statement. *PLoS Medicine* 2015; in press.

*Checklist is protected under Creative Commons Attribution (CC BY) license.

For peer review only

BMJ Open

Population Health Management to identify and characterise ongoing health need for high-risk individuals shielded from COVID-19: A Cross Sectional Cohort Study

Journal:	*BMJ Open*
Manuscript ID	bmjopen-2020-041370.R1
Article Type:	Original research
Date Submitted by the Author:	04-Aug-2020
Complete List of Authors:	Kenward, Charlie; NHS Bristol North Somerset and South Gloucestershire Clinical Commissioning Group<br>Pratt, Adrian; NHS Bristol North Somerset and South Gloucestershire Clinical Commissioning Group, Modelling and Analytics<br>Creavin, Sam T; University of Bristol, Department of Population Health Sciences<br>Wood, Richard; NHS Bristol North Somerset and South Gloucestershire Clinical Commissioning Group, Modelling and Analytics; University of Bath, School of Management<br>Cooper, Jennifer; University of Bristol, Department of Population Health Sciences; NHS Bristol North Somerset and South Gloucestershire Clinical Commissioning Group, Modelling and Analytics
**Primary Subject Heading**:	Epidemiology
Secondary Subject Heading:	Public health, Health informatics, Health policy, Infectious diseases
Keywords:	Public health < INFECTIOUS DISEASES, PRIMARY CARE, HEALTH SERVICES ADMINISTRATION & MANAGEMENT, Risk management < HEALTH SERVICES ADMINISTRATION & MANAGEMENT, EPIDEMIOLOGY, Health informatics < BIOTECHNOLOGY & BIOINFORMATICS

SCHOLARONE™
Manuscripts

I, the Submitting Author has the right to grant and does grant on behalf of all authors of the Work (as defined in the below author licence), an exclusive licence and/or a non-exclusive licence for contributions from authors who are: i) UK Crown employees; ii) where BMJ has agreed a CC-BY licence shall apply, and/or iii) in accordance with the terms applicable for US Federal Government officers or employees acting as part of their official duties; on a worldwide, perpetual, irrevocable, royalty-free basis to BMJ Publishing Group Ltd ("BMJ") its licensees and where the relevant Journal is co-owned by BMJ to the co-owners of the Journal, to publish the Work in this journal and any other BMJ products and to exploit all rights, as set out in our licence.

The Submitting Author accepts and understands that any supply made under these terms is made by BMJ to the Submitting Author unless you are acting as an employee on behalf of your employer or a postgraduate student of an affiliated institution which is paying any applicable article publishing charge ("APC") for Open Access articles. Where the Submitting Author wishes to make the Work available on an Open Access basis (and intends to pay the relevant APC), the terms of reuse of such Open Access shall be governed by a Creative Commons licence – details of these licences and which Creative Commons licence will apply to this Work are set out in our licence referred to above.

Other than as permitted in any relevant BMJ Author's Self Archiving Policies, I confirm this Work has not been accepted for publication elsewhere, is not being considered for publication elsewhere and does not duplicate material already published. I confirm all authors consent to publication of this Work and authorise the granting of this licence.

For peer review only

Population Health Management to identify and characterise ongoing health need for high-risk individuals shielded from COVID-19: A Cross Sectional Cohort Study

Charlie Kenward[1,*], Adrian Pratt[1], Sam Creavin[2], Richard M Wood[1,3], Jennifer A Cooper[1,2]

[1] Bristol, North Somerset and South Gloucestershire CCG; UK National Health Service; Bristol; UK.
[2] Population Health Sciences; Bristol Medical School; University of Bristol; Bristol.
[3] School of Management; University of Bath; Bath; UK.

* Corresponding author. Correspondence to Charlie Kenward; charlie.kenward@nhs.net

For peer review only

Abstract

Objectives: To use Population Health Management (PHM) methods to identify and characterise individuals at high-risk of severe COVID-19 for which shielding is required, for the purposes of managing ongoing health needs and mitigating potential shielding-induced harm.

Design: Individuals at 'high-risk' of COVID-19 were identified using the published national 'Shielded Patient List' criteria. Individual level information including current chronic conditions, historical healthcare utilisation, and demographic and socioeconomic status were used for descriptive analyses of this group using PHM methods. Segmentation used *k*-prototypes cluster analysis.

Setting: A major healthcare system in the South West of England, for which linked primary, secondary, community and mental health data is available in a system-wide dataset. The study was performed at a time considered to be relatively early in the COVID-19 pandemic in the UK.

Participants: 1,013,940 individuals from 78 contributing GP practices.

Results: Compared to the groups considered at 'low' and 'moderate' risk (i.e. eligible for the annual influenza vaccination), individuals at high-risk were older (median 68 years [IQR: 55-77] *c.f.* 30 [18-44] and 63 [38-73], respectively), with more healthcare contacts in the previous year (median 11 [4-26] *c.f.* 0 [0-2] and 2 [0-9]) and had a higher burden of comorbidity (median Charlson score 4 [3-6], *c.f.* 0 [0-0] and 2 [1-4]). Geospatial analyses revealed that 3.3% of rural and semi-rural residents were in the high-risk group compared to 2.91% of urban and inner-city residents ($p<0.001$). Segmentation uncovered six distinct clusters comprising the high-risk population, with key differentiation based upon age and presence of cancer, respiratory, and mental health conditions.

Conclusions: PHM methods are useful in characterising the needs of individuals requiring shielding. Segmentation of the high-risk population identified groups with distinct characteristics that may benefit from a more tailored response from health and care providers and policy makers.

Keywords: COVID-19, Population Health Management, Cluster Analysis, Delivery of Health Care, severe acute respiratory syndrome coronavirus 2, Unsupervised Machine Learning

Word Count: 4806

For peer review only - http://bmjopen.bmj.com/site/about/guidelines.xhtml

Article Summary

Strengths and limitations of this study

- Based upon a linked dataset for one million individuals combining information across primary, secondary, community, and mental health services.
- Criteria used for identifying high-risk individuals were subject to double clinical review and are fully documented to aid reproducibility.
- Results can facilitate health and care interventions that may be tailored for sub-groups within the high-risk population.
- Results reflect the local population where the study was performed, which may limit portability to other settings.

For peer review only

For peer review only - http://bmjopen.bmj.com/site/about/guidelines.xhtml

1. Introduction

Severe acute respiratory syndrome coronavirus 2 (SARS-CoV-2), responsible for the disease known as COVID-19, was declared a pandemic by the World Health Organisation on 11th March 2020. It has threatened to overwhelm, and in some cases has overwhelmed, health systems regardless of the economic status of the affected nation.[1 2] SARS-CoV-2 has the ability to spread quickly, partly owing to infected individuals being contagious during the early and asymptomatic phase.[3-5] While COVID-19 is a mild or flu-like illness in most people, for an estimated 14% the disease can be considered 'severe', with an estimated 6% of cases becoming 'critical' (involving life-threatening pneumonia and respiratory failure).[6] Combined with an estimated case fatality rate of approximately 1.4%,[7] this has led many countries to take drastic policy measures to suppress and contain viral transmission such as lockdown, social distancing and mask wearing, and to shield those at highest risk of severe illness through self-isolation.[8-10]

Faced with early projections that approximately 80% of the population would contract the virus over the course of the epidemic,[8] the UK Government has sought to shield the most vulnerable members of the population. In England, efforts were made to identify and inform an estimated 1.5 million people considered to be high-risk,[11] of the need to self-isolate within their homes for at least 12 weeks. However, this policy comes at the potential cost of shielding-induced harm such as negative mental health effects, reduced exercise, and barriers in accessing health services; which people are likely to require due to the nature of the condition(s) that has led to their shielding. Furthermore, the strategy puts a further strain on local health and care services having to meet the often complex needs of this group outside of normal ways of working. Responding to this challenge requires a detailed understanding of individuals at high-risk, including a characterisation of their health and social needs and the ways in which they normally interact with health and care services.

Population Health Management (PHM) approaches can enable greater proactivity in the response of health and care services to emerging or otherwise unrecognised need, and allow for more personalised and preventative healthcare interventions.[12] As an emerging concept involving an integrated consideration of health determinants, outcomes and interventions,[13] PHM has a substantial presence in the published Long Term Plan for England's National Health Service (NHS).[12] Yet a key challenge to embedding PHM is the ability to source and link record-level data across the health and care divide, with primary care of particular importance given the range of information relating to diagnoses, comorbidities, social status and prescriptions.[14 15] This breadth of data is required to accurately identify the high-risk individuals that require shielding (in a similar manner to its use in previous studies to identify individuals at risk of developing long-term conditions).[14 16] In addition to *identifying* the high-risk group, PHM approaches can also be leveraged to provide a more holistic *understanding* of these individuals in informing an effective response to their needs. As part of a range of descriptive analyses associated with PHM, population segmentation supports this by cutting through the complexity of large and multivariate linked datasets in determining a manageable number of population groups separable by differences in health determinants or outcomes.[17-19] This may be an important tool in understanding the composition of the high-risk group considered here.

The objective of this study was to use Population Health Management (PHM) methods to identify and characterise a high-risk population for which shielding is required, for the purposes of managing ongoing health needs and mitigating potential shielding-induced harm. Set in a large healthcare system in South West England during the early stages of the COVID-19 pandemic, this study makes use of a linked dataset containing healthcare activity and clinical, demographic and social attributes for one million individuals.

For peer review only - http://bmjopen.bmj.com/site/about/guidelines.xhtml

2. Materials and methods

The Reporting of studies Conducted using Observational Routinely-collected Data (RECORD)[20] statement was used as an extension of the STROBE guidelines[21] (Supplementary Material A).

2.1 Data, application and setting

The application of this study was to the Bristol, North Somerset and South Gloucestershire (BNSSG) healthcare system, which is a one million resident health economy across a mixture of urban and rural geographies. Within the system, there are 82 GP practices and three major hospitals. This cross sectional cohort study took place at a time understood to be early in the pandemic, shortly after the non-pharmaceutical interventions (social distancing and isolation strategies) were nationally implemented on 23 March 2020.[22]

The BNSSG system-wide dataset[23] was used to support the analysis contained in this study. This dataset forms part of the PHM infrastructure within the BNSSG healthcare system, and contains information for 1,013,940 registered individuals (from 78 contributing practices) across two tables. The first table consists of individual attributes including presence of clinical conditions, lifestyle factors, and demographic and socioeconomic information. This data is principally derived from GP patient administration systems (all GP practices use EMIS Web). The second table contains information for various patient-related activities such as GP consultations, hospital admissions, mental health appointments, community visits, and prescriptions. This data is sourced across primary and secondary care, mental health and community services and contains information such as specialty and relevant dates and times. A unique individual identifier is used to link the data between the tables. Eligibility criteria included all individuals registered to a contributing practice within BNSSG.

The Yorkshire & The Humber, Leeds East Research Ethics Committee granted NHS HRA Research Database approval for the Bristol, North Somerset & South Gloucestershire System Wide Database in 2020 (REC Reference Number: 20/YH/0185, Date: 28/07/2020). Ethical approval is given for processing of personal data by the Research Database team for research conducted on COVID-19 related priority areas identified by the wider health and care system (in accordance with the Health Service Control Of Patient Information Regulations 2002 (COPI) directive for COVID-19 related analyses).[24]

Patient and public involvement statement

Patients and/or the public were not involved in the design, conduct, reporting or dissemination plans of this research.

2.2 Identifying high-risk individuals

In the UK, construction of the Shielded Patient List (SPL) was led by the Chief Medical Officers (CMOs) of the four home nations, with support from the various medical Royal Colleges and Societies. From the outset, individuals were considered to require shielding if they were a member of one or more of six outlined groups. This included those who have received an organ transplant; those with specific cancers, severe respiratory conditions, and rare diseases that increase the risk of infection; and those who are on immunosuppression therapies or are pregnant with significant congenital heart disease.[25] In developing the specific criteria used to define membership of these groups, a set of central database searches was first published by NHS Digital in March 2020. This accounted for approximately 900,000 of the total 1.5 million people originally estimated to require shielding. In seeking to address this shortfall, the membership criteria has since evolved through additional incorporation of patient lists sent by hospital consultants and GPs, and self-reporting by the public.[11]

Given the manner in which the SPL has incrementally advanced, there are a number of issues affecting its ability to effectively identify the high-risk population for consideration within this study. First, it is

For peer review only - http://bmjopen.bmj.com/site/about/guidelines.xhtml

exposed to variation in clinical assessment and citizen reporting, i.e. there are elements of subjectivity. And second, there is anecdotal evidence emerging from frontline clinicians regarding concerns around over and under reporting of high-risk individuals contained on the SPL, i.e. it is to some degree incomplete. Ultimately, this approach has not resulted in a comprehensive database aligned to the SPL for the purposes of secondary uses as required by healthcare planners.

Consequently, the high-risk group of the BNSSG population considered in this study was identified through the merger of two constituent lists. The first of these is a BNSSG-level subset of the afore-mentioned SPL originally released by NHS Digital,[11] accounting for approximately 900,000 individuals nationally and 14,388 individuals for the BNSSG population. This was complemented with a list of individuals as identified through a number of local searches of the BNSSG system-wide dataset created in order to match as closely as possible the definitions of the six groups as originally outlined by the CMO (identifying 24,894). Combining these lists gave 29,798 high-risk individuals. Where an exact match in search field was not possible then it was either omitted, or a proxy search term was constructed upon review by two clinicians.[26] To reduce potential misclassification and information bias, high-risk criteria and coding lists were subject to double clinical review. Full criteria are documented in Supplementary Material B.

2.3 Analysing the high-risk group

The high-risk group was firstly analysed through comparison against the remaining population, partitioned to 'low' and 'moderate' risk groups. Moderate risk is defined by eligibility for the annual injectable influenza vaccination[11] and low, or baseline, risk is defined through membership of neither the moderate nor the high-risk groups. Descriptive analyses were performed on the basis of demographics, comorbidities, healthcare utilisation and geographical distribution. Pyramid plots were used to understand the differences in age and sex between individuals of these three groups. The high-risk group was then assessed against the other groups through a comparative analysis examining summary information for setting-level healthcare utilisation, prevalence of individual chronic conditions and non-specific mortality risk (through the Charlson score[27]), in addition to other demographic and socioeconomic variables. Geospatial analyses mapped the proportion of high-risk individuals onto the BNSSG geography at Lower Super Output Area (LSOA) level, in order to identify any differences by rurality or deprivation (as measured by the Index of Multiple Deprivation (IMD) which ranks LSOAs from most to least deprived on a scale from one to ten). There were very few instances of missing data since the data were derived from GP practices and other national data (any variables with missing data accounted <1% of the data). Where required, missing data were handled through a complete cases approach.

The high-risk group was segmented using cluster analysis, which identifies similar groups within a population through the use of statistical methods to maximise the difference between groups according to a given set of features. The features considered here relate to the range of demographic, clinical, and social attributes that could be of interest in gaining a high-level understanding of the clusters, and moreover that would make the clusters 'actionable' in terms of the nature of possible interventions applied to mitigate shielding-induced harm. Given the range of categorical and continuous features considered (Supplementary Material C), the *k*-prototypes clustering method[28] was selected due to its flexibility to accommodate such mixed data types. The number of clusters was selected based on a scree plot of the total within sum of squares versus the number of clusters, the Silhouette index[29] and based on the 'identifability' and 'actionability' of the clusters in a healthcare context[18] (Supplementary Material C). Both clustering and non-clustering variables were assessed for whether they were statistically different from other clusters, an approach used by recent studies to verify results and to indicate how segments differ on each clustering variable.[30 31] Firstly an omnibus statistical test was applied (ANOVA, Kruskal-Wallis, Chi square test) to confirm differences across clusters followed by 15 pairwise tests (t-test, Mann-Whitney U test and z-test for proportions) between all other clusters. A Bonferroni adjustment was made to the significance level of 0.05 due to using multiple test comparisons.

For peer review only - http://bmjopen.bmj.com/site/about/guidelines.xhtml

3. Results

A total of 1,013,940 individuals (50.02% female, median age 37 [IQR: 21-56]) were included within these analyses, with 29,798 (2.94%) identified as 'high-risk', 32.79% as moderate-risk, and the remaining 67.01% as low (baseline) risk. An age and sex breakdown for individuals at these various levels of risk is provided in Figure 1. Figure 1 shows that, according to the definitions used, younger and middle-aged individuals are generally at relatively low-risk, with females of typical reproductive age (20-44 years) at comparatively higher risk compared to males due to the additional risk borne through pregnancy (thus warranting their inclusion for the annual injectable influenza vaccine). In contrast, high-risk individuals are mostly aged between 40 years and 85 years. Figure 2 shows that the long-term conditions used to define the 'high-risk' group (Section 2.2) are more common with increasing age.

Figure 1. Population pyramid showing absolute numbers of the population in 5-year age bands stratified by high-risk (red), moderate risk (orange) and low risk (yellow).

Figure 2. Number of long-term conditions by 5-year age band, with long-term conditions assessed through the Quality and Outcomes Framework (QOF) definitions.

A comparative analysis of the low, moderate and high-risk groups is provided in Table 1 with regard to demographic and socioeconomic information, historical healthcare utilisation, and burden of comorbidity. The high-risk group, as expected through definition, are on average older, have a higher comorbidity rate (Charlson score increases across risk stratas, from a median of 0 [IQR: 0-0] for low-risk to 4 [3-6] for high-risk) and have greater healthcare utilisation for all appointment types. For instance, compared to the group at low-risk, the high-risk group have on average 6 times the number of primary care and community contacts over the preceding calendar year and twice the number compared to the moderate-risk group. In terms of comorbidities, the high-risk group have a much higher proportion of individuals diagnosed with cancer in the past 5 years (24.03%) and with COPD (34.84%) compared to the rest of the cohort. In addition, the proportion of those with cardiovascular conditions (19.59%) in the high-risk group is higher compared to the rest of the cohort (3.48%) and for those who are on medication which requires monitoring (24.13% versus 1.52%). Further comparisons in Supplementary Material D.

For peer review only

For peer review only - http://bmjopen.bmj.com/site/about/guidelines.xhtml

Table 1. Comparative analysis of the low (n=679,457), moderate (n=304,685), and high risk (n=29,798) groups with regard to demographic and socioeconomic information, historical healthcare utilisation, and burden of comorbidity.

Variable	Low risk	Moderate risk	High risk
**Demographic and socioeconomic**			
Age (median, IQR)	30 (18-44)	63 (38-73)	68 (55-77)
Female (%)	48.25%	53.82%	51.77%
Deprivation by IMD decile* (median, IQR)	6 (3-8)	6 (4-9)	6 (3-8)
Urban/rural (%)<br>-Rural town and fringe<br>-Rural village and dispersed<br>-Urban city and town	 2.34%<br>3.85%<br>93.81%	 3.13%<br>5.19%<br>91.68%	 2.97%<br>4.81%<br>92.22%
Local authority (%)<br>-Bristol<br>-North Somerset<br>-South Gloucestershire	 52.24%<br>19.79%<br>27.97%	 42.58%<br>26.40%<br>31.02%	 43.74%<br>27.14%<br>29.12%
Has a carer (%)	0.31%	1.46%	2.89%
Housebound (%)	0.04%	1.61%	3.73%
**Healthcare utilisation (calendar year 2019)**			
Primary and community care contacts (median, IQR)<br>*Mean contacts per 1,000 population*	0 (0-2)<br>*1,620*	2 (0-5)<br>*4,753*	5 (2-10)<br>*9,766*
Mental health attendances (median, IQR)<br>*Mean contacts per 1,000 population*	0 (0-0)<br>*226*	0 (0-0)<br>*432*	0 (0-0)<br>*622*
Secondary care elective consultations and admissions (median, IQR)<br>*Mean contacts per 1,000 population*	0 (0-0)<br>*966*	0 (0-4)<br>*2,958*	6 (2-14)<br>*10,756*
Secondary care emergency attendances and admissions (median, IQR)<br>*Mean contacts per 1,000 population*	0 (0-0)<br>*295*	0 (0-0)<br>*527*	0 (0-2)<br>*1,184*
**Comorbidities**			
Cardiovascular condition (%)	0.068%	11.09%	19.59%
Cancer diagnosed** (%)	0.29%	1.85%	24.03%
Mental health condition (%)	9.44%	12.54%	16.96%
Diabetes (%)	0.07%	14.94%	19.95%
Dementia (%)	0.017%	2.31%	2.68%
Asthma (%)	0.82%	17.03%	22.27%
COPD (%)	0.04%	3.05%	34.84%
**Other**			
Drugs that require monitoring (%)***	0.64%	3.49%	24.13%
Charlson Score (median, IQR)	0 (0-0)	2 (1-4)	4 (3-6)
Smoker/Non Smoker (%)	12.94%	10.21%	16.21%

** For Index of Multiple Deprivation (IMD), note that 1 is the most deprived decile and 10 is least deprived decile.*
*** Diagnosis in past five years.*
****Drugs that require monitoring include immunosuppressant/immunomodulators (IMMIMM) (previous 6 months), biologic/monoclonal (IMMBIO) (previous 6 months) including rituximab (previous 12 months) and other drugs requiring monitoring (previous 2 months), fully specified in Supplementary Material B*

For peer review only - http://bmjopen.bmj.com/site/about/guidelines.xhtml

The concentration of high-risk individuals within the various LSOAs of the BNSSG healthcare system is illustrated in Figure 3. Given that each LSOA contains an average 1,500 individuals, those LSOAs larger in size represent rural geographies while those smaller in size and clustered together represent the greater population densities associated with urban conurbations. The large town of Weston-Super-Mare (population 80,000) appears in the lower area of the figure and the city of Bristol (population 463,000) appears towards the centre on the eastern side. Of the six localities comprising the BNSSG system, the centre and eastern areas of Bristol have the lowest proportion at high-risk (2.29%); and people in this area were younger compared to the other five localities (overall median age 32 years c.f. 38 years; 'high-risk' median age: 64 years c.f. 69 years). The greatest concentration of high-risk individuals (3.88%) is for the locality including Weston-Super-Mare and surrounding areas. The rural areas outside of Bristol account for 55% of the high-risk group (16,255 out of 29,798). Rural areas have a higher proportion of the population at of high-risk (3.33%) compared to urban city areas (2.91%) (*P* <0.001, test of two proportions).

Figure 3. Geographical map of the Bristol, North Somerset and South Gloucestershire healthcare system illustrating the concentration of high-risk individuals at Lower Super Output Areas (LSOA) level.

Table 2 shows the results of the segmentation analysis of the high-risk group (29,798) into six sub-groups. 224 individuals did not have an LSOA within the BNSSG area but were registered to a BNSSG practice and were removed from the analyses. The top utilisers of the high-risk group were removed (0.41%) leaving a high-risk population of 29,454. Clustering variables differ significantly across the segments as expected since the algorithm inherently creates clusters to differ as much as possible based on the variables. Non-clustering variables were also significantly different across groups. Pairwise comparisons between segments identify which segments differ from others, for instance, for cluster 2 the cardiovascular proportion (44.24%) differs significantly from all other clusters.

Cluster 1 contains only 0.57% of the high-risk group and accounts for those with the highest need in regard of mental health services (79% had a mental health diagnosis). This cluster had the greatest proportion of individuals with dementia (10.59%). Those with dementia in this cluster had a mean age of 78 versus 56 for those without dementia. Half the individuals with dementia had a mental health condition, whereas 17.76% of individuals without dementia had a mental health condition. Although this cluster had the highest proportion of people with dementia, membership of this group was largely defined by 78.82% of individuals having a mental health condition. Median Charlson comorbidity index for those with dementia was 6 (IQR: 5-7.75), those without dementia was 3 (IQR: 2-4).

Cluster 2 has the greatest age (median 76 years) and most frequent utilisation of primary, secondary and community care. Clinically this segment is characterised by a high prevalence of COPD (58%), cardiovascular disease (44%), diabetes (31%), and dementia (8%). Cluster 3 has the lowest age (median 51 years) and highest asthma prevalence (76%). Cluster 4 has the highest use of elective secondary care appointments with 88% having had a diagnosis of cancer in the past five years. Cluster 5 is characterised by the majority (78%) requiring physiological, biochemical or pharmacological monitoring of prescribed medication. Of this cluster, 60% had an immunosuppressant/immunomodulator prescribed in the previous 6 months, 22% had a biologic/monoclonal medication prescribed in the previous 6 months and 11% had another type of drug requiring monitoring in the previous 2 months (e.g. lithium, warfarin, anti-psychotic medications - see Supplementary Material B and C). Finally, Cluster 6 is characterised by an 85% COPD prevalence and the second highest smoking prevalence (25%). This cluster is also notable from having lower than average healthcare utilisation across all considered settings.

For peer review only - http://bmjopen.bmj.com/site/about/guidelines.xhtml

Table 2. Segmentation of high-risk group (*n*=29,454), detailing attributes of the six sub-groups as identified through cluster analysis.

Cluster	1. Complex Mental Health (*n*=170)	2. Older Complex (*n*=1372)	3. Younger Asthma (*n*=5327)	4. Recent Cancer (*n*=6612)	5. Drug Monitoring (*n*=6892)	6. Low Utilisation COPD (*n*=9171)	Overall omnibus test
**Clustering variables**							
Age, mean<br><br>*Median (IQR)*	58.4[a]<br><br>*58 (50.25-70.00)*	72.7[b]<br><br>*76 (66.00 – 84.00)*	48.3[a]<br><br>*51 (34.00 – 64.00)*	65.3[b]<br><br>*68 (57.00 – 76.00)*	65.1[b]<br><br>*69 (57.00 – 77.00)*	71.2[b]<br><br>*72 (64.00 – 80.00)*	AN <0.000
Female, *n* (%)	105 (61.76)	871[b] (63.48)	3892[b] (74.32)	2665[a] (40.31)	4850[b] (70.37)	2891[a] (31.52)	X^2 <0.000
Primary and community care contacts, median (IQR)<br><br>*Mean contacts per 1,000 population*	10[a] (5-20)<br><br>*16,006*	49[a] (25-83)<br><br>*61,847*	4 (2-9)<br><br>*6,721*	4 (2-9)<br><br>*6,808*	4 (2-8)<br><br>*6,493*	4 (2-9)<br><br>*6,733*	KW <0.000
Mental health attendances, median (IQR)<br><br>*Mean contacts per 1,000 population*	45.5[a] (34-62)<br><br>*49,876*	0[a] (0-0)<br><br>*612*	0[c] (0-0)<br><br>*175*	0[a] (0-0)<br><br>*53*	0[c] (0-0)<br><br>*155*	0[c] (0-0)<br><br>*155*	KW <0.000
Secondary care elective consultations and admissions, median (IQR)<br><br>*Mean contacts per 1,000 population*	6[c] (2-13)<br><br>*10,288*	11[a] (5-20)<br><br>*15,136*	5[b] (1-11)<br><br>*7,860*	16[a] (8-28)<br><br>*21,332*	7[b] (3-13)<br><br>*9,020*	3[a] (0-7)<br><br>*4,818*	KW <0.000
Secondary care emergency attendances and admissions, median (IQR)<br><br>*Mean contacts per 1,000 population*	2[a] (0-3)<br><br>*2,365*	6[a] (4-9)<br><br>*6,820*	0[a] (0-2)<br><br>*1,089*	0[a] (0-1)<br><br>*940*	0[a] (0-1)<br><br>*680*	0[a] (0-1)<br><br>*786*	KW <0.000
Cardiovascular condition (current), *n* (%)	27 (15.88)	607[a] (44.24)	435[b] (8.31)	1018[c] (15.4)	1093[c] (15.86)	2569[b] (28.01)	X^2 <0.000
Cancer diagnosed in the past 5 years, *n* (%)	25[b] (14.71)	230[b] (16.76)	358[a] (6.84)	5813[a] (87.92)	224[a] (3.25)	431[a] (4.7)	X^2 <0.000
Mental Health, *n* (%)	134[a] (78.82)	308[b] (22.45)	1181[b] (22.55)	766[a] (11.58)	1067[b] (15.48)	1516[b] (16.53)	X^2 <0.000
Diabetes, *n* (%)	39 (22.94)	419[b] (30.54)	746[c] (14.24)	1098[c] (16.61)	1384[b] (20.08)	2168[b] (23.64)	X^2 <0.000
Dementia, *n* (%)	18[b] (10.59)	112[b] (8.16)	35[a] (0.67)	91[a] (1.38)	192[b] (2.79)	329[b] (3.59)	X^2 <0.000
Asthma, *n* (%)	54[b] (31.76)	322[b] (23.47)	3970[a] (75.81)	358[a] (5.41)	507[a] (7.36)	1358[a] (14.81)	X^2 <0.000
COPD, *n* (%)	78[b] (45.88)	800[b] (58.31)	1053[a] (20.11)	277[a] (4.19)	404[a] (5.86)	7677[a] (83.71)	X^2 <0.000
*Drugs that require monitoring, *n* (%)	67[a] (39.41)	243[a] (17.71)	640[a] (12.22)	351[b] (5.31)	5396[a] (78.29)	403[b] (4.39)	X^2 <0.000
**Non-clustering variables**							
Smoking, *n* (%)<br><br>-Non smoker<br>-Current smoker	63[b] (37.06)	211[b] (15.38)	888[b] (16.96)	626[b] (9.47)	679[b] (9.85)	2317[b] (25.26)	X^2 <0.000
Urban/rural, *n* (%)<br>-Rural town and fringe (rural)<br>-Rural village and dispersed (rural)<br>-Urban city and town (urban)	6 (3.53)<br>5 (2.94)<br>159 (93.53)	45 (3.28)<br>41 (2.99)<br>1286 (93.73)	125 (2.39)<br>200 (3.82)<br>4912 (93.79)	226 (3.42)<br>408 (6.17)<br>5978 (90.41)	234 (3.40)<br>407 (5.91)<br>6251 (90.70)	237 (2.58)<br>359 (3.91)<br>8575 (93.5)	For Urban versus Rural: X^2 <0.000

For peer review only

For peer review only - http://bmjopen.bmj.com/site/about/guidelines.xhtml

IMD decile, median (IQR)	4 (2-7)	5 (3-8)	5 (3-8)	7[a] (4-9)	7[a] (4-9)	5 (2-8)	KW <0.000
Learning disabilities and autism, *n* (%)	7[c] (4.12)	20[b] (1.46)	105[c] (2.00)	28[c] (0.42)	32[c] (0.46)	38[c] (0.41)	X^2 <0.000
Housebound, *n* (%)	19[a] (11.18)	328[a] (23.91)	56[b] (1.07)	104[b] (1.57)	181[a] (2.63)	393[a] (4.29)	X^2 <0.000
Has a carer, *n* (%)	10 (5.88)	134[b] (9.77)	75[c] (1.43)	137 (2.07)	148 (2.15)	341[b] (3.72)	X^2 <0.000
Is a carer, *n* (%)	3 (1.76)	68 (4.96)	162 (3.09)	210 (3.18)	271 (3.93)	343 (3.74)	X^2 0.002
Charlson Score, median (IQR)	3[b] (2-5)	6[a] (4-7)	2[a] (1-4)	5[a] (3-6)	4[b] (2-5)	5[a] (3-6)	KW <0.000

**Drugs that require monitoring include immunosuppressant/immunomodulators (IMMIMM) (previous 6 months), biologic/monoclonal (IMMBIO) (previous 6 months) including rituximab (previous 12 months) and other drugs requiring monitoring (previous 2 months), fully specified in Supplementary Material B*

[a] Significantly different from 5 other clusters (all other clusters)
[b] Significantly different from 4 other clusters
[c] Significantly different from 3 other clusters
Bonferroni adjustment to significance level: 0.05/15 = 0.0033 (based on 15 pairwise tests)

4. Discussion

4.1 Summary of main findings

In aggregated routine cross-sectional data from a single healthcare system (1,013,940 population), 2.94% (29,798) of the population met the UK NHS criteria for being at high-risk of severe COVID-19 illness. The proportion of people at high-risk of COVID-19 increased from 2.88% of those aged 50-54 years to 13.21% of those aged 80-84 years, whereas 9.46% of those aged 90-94 years and only 6.79% of those aged 90+ were at 'high-risk'. Compared to those who were not at high-risk of COVID-19, the high-risk group were older, more frequently attended healthcare, and scored highly on the Charlson co-morbidity index. Rural and semi-rural areas, and coastal towns had higher proportions of the population at high-risk of severe COVID-19 illness than urban and inner-city areas. Segmentation of the high-risk population identified six clusters reflecting the distinct characteristics of types of individual that may benefit from a more tailored response from health and care providers and policy makers. At the time of writing, this is the first study in the published literature to evaluate the attributes and needs of a shielded population through the application of PHM approaches.

4.2 Strengths and limitations

A key strength is the large dataset combining individual level data available from the electronic medical record with granular data on healthcare utilisation, demography and geography across a population over one million people. In particular, the dataset includes healthcare utilisation which covers community, primary and secondary care, and mental health services. A further strength is the collaborative co-production of findings drawing on a multi-disciplinary team comprising clinicians, data-scientists and commissioners. This has been possible through the PHM infrastructure embedded at the healthcare system under study. Finally, we demonstrated the potential to develop a comprehensive and accurate search based on this rich, linked data. The criteria used for identifying high-risk individuals were subject to double clinical review and were fully documented to aid reproducibility.

The study had its limitations. Firstly, the findings were derived from a single health and care system in one country (UK) which may hinder generalisability. However, the BNSSG population closely matches the age and sex distribution for England using ONS data and PHE data.[32 33] The results may therefore be portable to other English systems, or countries with similar demographics and health status. Additionally, the principle that population segmentation can be rapidly used to identify actionable insights and support a commissioning response to a healthcare emergency is broadly applicable.

For peer review only - http://bmjopen.bmj.com/site/about/guidelines.xhtml

For peer review only

Secondly, the findings rely on routinely coded data, and do not utilise free text in the medical record which may introduce information bias. While reflecting clinical practice, the ascertainment of clinical characteristics (especially diseases) by coded data is likely to both over and under classify people as having disease. Data on medication and prescriptions are likely to be more completely ascertained but may not reflect medication usage. In contrast, healthcare utilisation data are likely to be most completely ascertained (although difficult to ascertain DNAs and administrative errors).

There is also the possibility for differential-coding, i.e. people in certain groups may be less likely to be correctly coded than others. Examples include people who are homeless, experiencing socio-economic disadvantage or language barriers, or those in the travelling community. This may result in differential misclassification especially towards people who are at high-risk of COVID-19 illness. Finally, a computer driven approach can identify individuals quickly from large patient lists and records but additional benefit could be obtained by providing further support to practices to identify high risk individuals using their knowledge of individual patient health and care needs.

4.3 Interpretation

The resulting size of the derived BNSSG high-risk population is comparable to an estimated expected number of around 27,500 (1.5 million national high-risk individuals out of 55 million population applied to our cohort). This verifies the search criteria developed to identify these high-risk individuals. Through leveraging more granular data as contained in established PHM infrastructure, this approach both promotes enhanced risk-sensitivity through reducing the risk of over and under reporting (as is possible through coarser central database searches) and enables a comprehensive specification of the search criteria.

Of the six clusters, four were determined by criteria related to the definition of high-risk, i.e. 'younger **asthma'** (n=5,327), 'recent **cancer'** (n=6,612), **drug** monitoring' (n=6,892) and 'low utilisation **COPD'** (n=9,171). Four of the six clusters had a higher proportion of women (Younger Asthma 74%; Drug monitoring 70%; Older complex 63%; Mental health 62%) than men, whereas the COPD cluster and recent cancer had a higher proportion of men (68% and 60%). This reflects known demographics: women are more likely than men to have asthma and autoimmune disease (which often requires drug monitoring), live longer than men, and are more likely to be diagnosed with mental health disorders.[34 35 36 37]. In contrast men are more often diagnosed with cancer and COPD than women.[38] An interesting finding was the small (n=170) but important complex mental health cluster, the result of a high prevalence of comorbidities.

4.4 Implications

It is important to note that at the time of writing it is not known whether the UK shielding strategy has been effective in terms of reducing the burden of COVID-19 or overall harm for that defined population. Our findings are therefore relevant to healthcare planners and clinicians because they offer insights at the level of the population. There are three important implications for planners. Firstly, the population at high-risk of COVID-19 reflects a heterogeneous group of people who will require different interventions and response to mitigate the risk of shielding-induced harm. Mitigating interventions should be targeted at clusters among other important groups such as those with learning disabilities. For instance, targeted smoking cessation advice by text message for people in the low utilisation COPD group, targeted medication reviews for those in the drug monitoring group; and an individualised, proactive multi-disciplinary care plan for the 0.02% of the population in the complex mental health group.

Secondly our analysis shows that population segmentation can be used to highlight geographic areas of greatest need during a pandemic, drawing parallels with the long term challenges facing rural and coastal town health highlighted by England's CMO.[39] Policy-makers and clinicians can use these findings to understand how the capacity of healthcare systems reflect the likely demand. The third important implication is that local and national policy makers must explicitly consider the risk of systematic misclassification, even in times of pandemic. The shielding policy is based on at least two assertions: that the criteria as operationalised identify the group at highest risk of serious illness (a classification question),

For peer review only - http://bmjopen.bmj.com/site/about/guidelines.xhtml

For peer review only

which is likely to currently under-classify high risk populations, and that shielding reduces the risk of serious illness to an extent that outweighs any harms (a complex-intervention question), for which there is currently a lack of evidence. The former is now supported in part by a large UK-based study using the OpenSAFELY Platform, identifying independent risk factors for severe COVID-19 disease which was consistent with the criteria used to determine the SPL,[40] although it is notable that older age was the strongest predictor of in-hospital death and was not an explicit factor in the criteria. The extent to which the latter assertion is true is as yet unknown, which is important because there are potential harms to individuals from both the misclassification in both directs and to society from misallocation of resources.

Algorithms and artificial intelligence are at risk of exacerbating health inequalities by systematic misclassification.[41] We found that in our population, the proportion classified as high-risk according to national criteria was similar for those aged over 95 years as in those aged 65-69 years. Given that the proportion at high-risk increases monotonically until age 85 there is a concern that this may reflect systematic under-classification of risk in the oldest-old of our population, especially because the proportion at low-risk paradoxically increases from age 85 years onwards. Systematic misclassification could also partly explain the lower proportion of people at 'high-risk' in our deprived populations who may have differential ascertainment of underlying disease and higher risk of transmission due to living arrangements. Our concerns about systematic misclassification are especially relevant given the high excess death rate in care facilities and in people of black and minority ethnic populations.[40 42] Broadening the high-risk criteria would however induce a further significant personal and societal cost; including people with a Charlson score of six and greater would result in 45,000 people classified as high-risk, adding an additional ~15,000 to the current shielded BNSSG population.

There are also further limitations with the principle of shielding. Firstly, shielding has a very limited evidence base, and therefore, as with much of the policy response to COVID-19, the international implementation has been varied. For example, South Korea did not implement shielding and has a very low number of deaths reported from COVID-19. However, at this stage we believe that limitations in the comparability of the death rate (or indeed excess deaths) between international states preclude the comparison of death rates by interventions on a state-by-state basis. Secondly, shielding can be difficult to implement on a practical level for the individual. Further work could explore the strategies that individuals used to operationalise shielding on an everyday basis, the extent to which this adhered to national guidelines, and whether there were any barriers to implementation that could have been better mitigated by a more comprehensive response from the health system. However, regardless of these limitations our report demonstrates how PHM methods can be applied at pace, during a pandemic, to identify geographic areas and characterise clusters of people who likely have additional health needs and support actionable insights to safeguard population health.

4.5 Future work

Two initial priorities for further work are to apply these clusters to other geographic settings to determine whether the clusters hold in other healthcare settings and to prospectively determine the actual risk of severe COVID-19 illness and subsequent outcomes in people who are determined to be at high-risk. With planned developments of our dataset, a further study could examine in greater depth the observed risks and outcomes in terms of hospitalisation, discharges to care settings and mortality among the shielded population. The high-risk definition was developed at pace and with the limited evidence, and expert opinion available at the time. However, risk factors that were not included were age, ethnicity, male sex, multi-morbidity or other single comorbidities such as diabetes or heart disease, each of which has been shown to be independently associated with increased risk of severe disease [7 40 43-46]

As we move into a new phase of the pandemic,[47] which may involve further case surges, the development of accurate, risk prediction models are going to be an essential part of local health and care responses.[48] In addition, research is needed to understand and maximise the classification accuracy of search criteria used for population level datasets, as has been performed in the clinical practice research database,[49] and how

For peer review only - http://bmjopen.bmj.com/site/about/guidelines.xhtml

these can be integrated as support mechanisms for front-line clinicians, who ultimately hold responsibility for making decisions on who meets the criteria for shielding. Critically, it needs to be established whether shielding is effective. Shielding may be undermined by non-concordance with the rules, a reliance on social, medical and care interactions, or from being resident in a high risk setting such as a care home. This is especially important given the lack of evidence for the rest of the non-shielded population having acquired 'herd immunity'. Without such evidence there is a risk to the wellbeing of current and future shielded populations, induced by the shielding process. Finally, it should be examined whether there are more effective strategies such as widespread use of face coverings in public and effective 'track and trace' systems.

4.6 Conclusions

PHM methods applied to a system-wide linked dataset are useful in identifying and characterising a population at high-risk of COVID-19 for which shielding is required. Cluster analysis of the high-risk group revealed heterogeneous groups that may benefit from a more tailored response from health and care providers and policy makers and prompts further examination of a policy which is not without potential harm.

Acknowledgements
Support in developing and providing the searches of primary care data was provided by Rhys Lewis and Dr Jacob Lee, OneCare. Geographical distribution map of COVID-19 high-risk proportions was produced by Abbie Reynolds-Beer, BNSSG CCG.

Contributors
CK designed the study, constructed the high-risk criteria using the BNSSG system-wide dataset and drafted and revised the paper. JC designed the study, carried out data analysis including cluster analysis modelling, and drafted and revised the paper. RW contributed to study design and data analytics and drafted and revised the paper. SC constructed the high-risk criteria, provided clinical input and interpretation and reviewed the paper. AP contributed to data analysis, construction of the dataset/variables and drafted the manuscript. All authors have approved the final version of the manuscript for publication.

Funding
This study was supported by the NIHR Bristol Biomedical Research Centre at University Hospitals Bristol and Weston NHS Foundation Trust and the University of Bristol. The views expressed are those of the author(s) and not necessarily those of the NIHR or the Department of Health and Social Care.

Competing Interests
The authors report no conflicts of interest.

Patient and public involvement
Patients and/or the public were not involved in the design, or conduct, or reporting, or dissemination plans of this research.

Patient consent for publication
Not required.

Ethics Approval
The Yorkshire & The Humber – Leeds East Research Ethics Committee granted NHS HRA Research Database approval for the Bristol, North Somerset & South Gloucestershire System Wide Database in 2020 (REC Reference Number: 20/YH/0185, Date: 28/07/2020). Ethical approval is given for processing of personal data by the Research Database team for research conducted on COVID-19 related priority areas identified by the wider health and care system (in accordance with the Health Service Control Of Patient Information Regulations 2002 (COPI) directive for COVID-19 related

For peer review only - http://bmjopen.bmj.com/site/about/guidelines.xhtml

analyses). Under General Data Protection Regulation, the Data Controller was BNSSG Clinical Commissioning Group. Data from GP electronic medical records were extracted according to existing data sharing agreements and in line with GDPR and Caldicott principles under pandemic conditions and in accordance with government guidelines and the COPI directive.

Data availability statement

Technical appendix and coding lists available in online supplementary files. The R code and data variables used for analyses are included on the following GitHub page [https://github.com/nhs-bnssg-analytics/kprotocluster]. Local data used for the study are not publicly available.

References

1. Nacoti M, Ciocca A, Giupponi A, et al. At the Epicenter of the Covid-19 Pandemic and Humanitarian Crises in Italy: Changing Perspectives on Preparation and Mitigation. *Catalyst non-issue content* 2020;1(2) doi: 10.1056/CAT.20.0080
2. Stefanini GG, Azzolini E, Condorelli G. Critical Organizational Issues for Cardiologists in the COVID-19 Outbreak: A Frontline Experience From Milan, Italy. *Circulation* 2020 doi: 10.1161/circulationaha.120.047070 [published Online First: 2020/03/26]
3. To KK, Tsang OT, Leung WS, et al. Temporal profiles of viral load in posterior oropharyngeal saliva samples and serum antibody responses during infection by SARS-CoV-2: an observational cohort study. *Lancet Infect Dis* 2020 doi: 10.1016/s1473-3099(20)30196-1 [published Online First: 2020/03/28]
4. Zhang J, Litvinova M, Wang W, et al. Evolving epidemiology and transmission dynamics of coronavirus disease 2019 outside Hubei province, China: a descriptive and modelling study. *Lancet Infect Dis* 2020 doi: 10.1016/s1473-3099(20)30230-9 [published Online First: 2020/04/06]
5. Wei WE, Li Z, Chiew CJ, et al. Presymptomatic Transmission of SARS-CoV-2 — Singapore, January 23–March 16, 2020. Morbidity and Mortality Weekly Report (MMWR), 2020:411-15.
6. WHO-China. Report of the WHO-China Joint Mission on Coronavirus Disease 2019 (COVID-19), 2020:11.
7. Verity R, Okell LC, Dorigatti I, et al. Estimates of the severity of coronavirus disease 2019: a model-based analysis. *The Lancet Infectious Diseases* 2020 doi: 10.1016/S1473-3099(20)30243-7
8. Neil M Ferguson, Daniel Laydon, Gemma Nedjati-Gilani, et al. Impact of non-pharmaceutical interventions (NPIs) to reduce COVID19 mortality and healthcare demand: WHO Collaborating Centre for Infectious Disease Modelling; 2020 [Available from: https://www.imperial.ac.uk/mrc-global-infectious-disease-analysis/news--wuhan-coronavirus/.
9. Tanne JH, Hayasaki E, Zastrow M, et al. Covid-19: how doctors and healthcare systems are tackling coronavirus worldwide. *BMJ* 2020;368:m1090. doi: 10.1136/bmj.m1090
10. Mulheirn I, Alvis S, Insall L, et al. A Sustainable Exit Strategy: Managing Uncertainty, Minimising Harm Tony Blair Institute for Global Change2020 [Available from: https://institute.global/policy/sustainable-exit-strategy-managing-uncertainty-minimising-harm accessed 1st May 2020 2020.
11. NHS Digital. COVID-19 – high risk shielded patient list identification methodology 2020 [Available from: https://digital.nhs.uk/coronavirus/shielded-patient-list/methodology accessed 1st May 2020 2020.
12. NHS England. Population Health and the Population Health Management Programme 2020 [Available from: https://www.england.nhs.uk/integratedcare/building-blocks/phm/.
13. Kindig D, Stoddart G. What is population health? *Am J Public Health* 2003;93(3):380-83. doi: 10.2105/ajph.93.3.380
14. Feldman K, Stiglic G, Dasgupta D, et al. Insights into Population Health Management Through Disease Diagnoses Networks. *Scientific Reports* 2016;6(1):30465. doi: 10.1038/srep30465

For peer review only - http://bmjopen.bmj.com/site/about/guidelines.xhtml

15. Asbridge J, Davies M. Clinical care in the home and population health management: improving the care of patients with long-term health conditions. *European Journal for Person Centered Healthcare*;5(1):148-53.
16. Hibbard JH, Greene J, Sacks RM, et al. Improving Population Health Management Strategies: Identifying Patients Who Are More Likely to Be Users of Avoidable Costly Care and Those More Likely to Develop a New Chronic Disease. *Health services research* 2017;52(4):1297-309. doi: 10.1111/1475-6773.12545 [published Online First: 2016/08/22]
17. Yan S, Seng BJJ, Kwan YH, et al. Identifying heterogeneous health profiles of primary care utilizers and their differential healthcare utilization and mortality - a retrospective cohort study. *BMC Fam Pract* 2019;20(1):54. doi: 10.1186/s12875-019-0939-2 [published Online First: 2019/04/25]
18. Chong JL, Lim KK, Matchar DB. Population segmentation based on healthcare needs: a systematic review. *Syst Rev* 2019;8(1):202. doi: 10.1186/s13643-019-1105-6 [published Online First: 2019/08/15]
19. Wood RM, Murch BJ, Betteridge RC. A comparison of population segmentation methods. *Operations Research for Health Care* 2019
20. Benchimol EI, Smeeth L, Guttmann A, et al. The REporting of studies Conducted using Observational Routinely-collected health Data (RECORD) statement. *PLoS Med* 2015;12(10):e1001885. doi: 10.1371/journal.pmed.1001885 [published Online First: 2015/10/07]
21. von Elm E, Altman DG, Egger M, et al. Strengthening the Reporting of Observational Studies in Epidemiology (STROBE) statement: guidelines for reporting observational studies. *Bmj* 2007;335(7624):806-8. doi: 10.1136/bmj.39335.541782.AD [published Online First: 2007/10/20]
22. Iacobucci G. Covid-19: UK lockdown is "crucial" to saving lives, say doctors and scientists. *BMJ* 2020;368:m1204. doi: 10.1136/bmj.m1204 [published Online First: 2020/03/27]
23. Healthier Together. A system-wide dataset for Bristol, North Somerset, and South Gloucestershire 2020 [Available from: https://bnssghealthiertogether.org.uk/population-health-management/.
24. Department of Health and Social Care. Coronavirus (COVID-19): notification to organisations to share information, 2020.
25. NHS Digital. RETIRED - shielded patient list identification methodology Version 1 2020 [Available from: https://digital.nhs.uk/coronavirus/shielded-patient-list/methodology/shielded-patient-list-methodology-version-1 accessed 1st May 2020 2020.
26. NHS Digital. Coronavirus (COVID-19): Shielded patients list 2020 [Available from: https://digital.nhs.uk/coronavirus/shielded-patient-list.
27. Charlson M, Szatrowski TP, Peterson J, et al. Validation of a combined comorbidity index. *J Clin Epidemiol* 1994;47(11):1245-51. doi: 10.1016/0895-4356(94)90129-5 [published Online First: 1994/11/01]
28. Huang Z. Extensions to the k-Means Algorithm for Clustering Large Data Sets with Categorical Values. *Data Mining and Knowledge Discovery* 1998;2(3):283-304. doi: 10.1023/A:1009769707641
29. Rousseeuw PJ. Silhouettes: A graphical aid to the interpretation and validation of cluster analysis. *Journal of Computational and Applied Mathematics* 1987;20:53-65. doi: https://doi.org/10.1016/0377-0427(87)90125-7
30. Vuik SI, Mayer E, Darzi A. Enhancing risk stratification for use in integrated care: a cluster analysis of high-risk patients in a retrospective cohort study. *BMJ Open* 2016;6(12):e012903. doi: 10.1136/bmjopen-2016-012903
31. Everitt BS, Landau S, Leese M, et al. Cluster analysis. 5th ed. Chichester: John Wiley & Sons 2011.
32. Office for National Statistics. Estimates of the population for the UK, England and Wales, Scotland and Northern Ireland. Mid-2019. 2020 [Available from: https://www.ons.gov.uk/peoplepopulationandcommunity/populationandmigration/populationestimates/datasets/populationestimatesforukenglandandwalesscotlandandnorthernireland accessed 1st June 2020.

For peer review only - http://bmjopen.bmj.com/site/about/guidelines.xhtml

33. Public Health England. Public Health Profile: NHS Bristol, North Somerset and South Gloucestershire CCG 2020 [Available from: https://fingertips.phe.org.uk/search/population#page/12/gid/1/pat/46/par/E39000043/ati/165/are/E38000222/cid/4/tbm/1/page-options/ovw-tdo-0 accessed 1st June 2020.
34. Fairweather D, Frisancho-Kiss S, Rose NR. Sex differences in autoimmune disease from a pathological perspective. *Am J Pathol* 2008;173(3):600-9. doi: 10.2353/ajpath.2008.071008 [published Online First: 2008/08/09]
35. Altemus M, Sarvaiya N, Neill Epperson C. Sex differences in anxiety and depression clinical perspectives. *Front Neuroendocrinol* 2014;35(3):320-30. doi: 10.1016/j.yfrne.2014.05.004 [published Online First: 2014/06/03]
36. World Health Organisation. Gender and Mental Health, 2002.
37. Leynaert B, Sunyer J, Garcia-Esteban R, et al. Gender differences in prevalence, diagnosis and incidence of allergic and non-allergic asthma: a population-based cohort. *Thorax* 2012;67(7):625. doi: 10.1136/thoraxjnl-2011-201249
38. Smittenaar CR, Petersen KA, Stewart K, et al. Cancer incidence and mortality projections in the UK until 2035. *Br J Cancer* 2016;115(9):1147-55. doi: 10.1038/bjc.2016.304 [published Online First: 2016/10/26]
39. Nuffield Trust. Summit 2020 session: Prof Chris Whitty on health trends and projections over the next 20 years 2020 [Available from: https://www.nuffieldtrust.org.uk/media/summit-2020-session-prof-chris-whitty-on-the-direction-of-health-trends-over-the-next-20-years accessed 1st June 2020.
40. Williamson E, Walker AJ, Bhaskaran KJ, et al. OpenSAFELY: factors associated with COVID-19-related hospital death in the linked electronic health records of 17 million adult NHS patients. *medRxiv* 2020:2020.05.06.20092999. doi: 10.1101/2020.05.06.20092999
41. Panch T, Mattie H, Atun R. Artificial intelligence and algorithmic bias: implications for health systems. *J Glob Health* 2019;9(2):010318-18. doi: 10.7189/jogh.09.020318
42. Public Health England. Disparities in the risk and outcomes of COVID-19, 2020.
43. Wang B, Li R, Lu Z, et al. Does comorbidity increase the risk of patients with COVID-19: evidence from meta-analysis. *Aging* 2020;12(7):6049-57. doi: 10.18632/aging.103000
44. Zhou F, Yu T, Du R, et al. Clinical course and risk factors for mortality of adult inpatients with COVID-19 in Wuhan, China: a retrospective cohort study. *Lancet* 2020 doi: 10.1016/s0140-6736(20)30566-3 [published Online First: 2020/03/15]
45. WHO. Coronavirus disease (COVID-2019) situation report—30. 2020
46. European Centre for Disease Prevention and Control S, ,. Novel coronavirus disease 2019 (COVID-19) pandemic: increased transmission in the EU/EEA and the UK – sixth update. 12th March 2020., 2020.
47. NHS England. Second phase of NHS response to COVID-19: Letter from Sir Simon Stevens and Amanda Pritchard 2020 [Available from: https://www.england.nhs.uk/coronavirus/publication/second-phase-of-nhs-response-to-covid-19-letter-from-simon-stevens-and-amanda-pritchard/ accessed 1st June 2020 2020.
48. Wynants L, Van Calster B, Bonten MMJ, et al. Prediction models for diagnosis and prognosis of covid-19 infection: systematic review and critical appraisal. *Bmj* 2020;369:m1328. doi: 10.1136/bmj.m1328 [published Online First: 2020/04/09]
49. Quint JK, Müllerova H, DiSantostefano RL, et al. Validation of chronic obstructive pulmonary disease recording in the Clinical Practice Research Datalink (CPRD-GOLD). *BMJ Open* 2014;4(7):e005540. doi: 10.1136/bmjopen-2014-005540

For peer review only - http://bmjopen.bmj.com/site/about/guidelines.xhtml

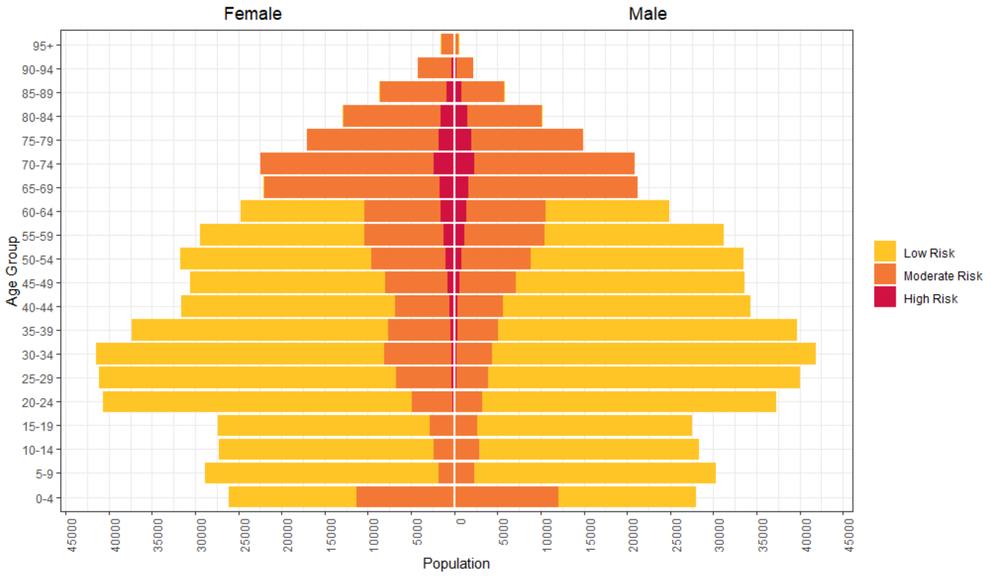

Figure 1. Population pyramid showing absolute numbers of the population in 5-year age bands stratified by high-risk (red), moderate risk (orange) and low risk (yellow).

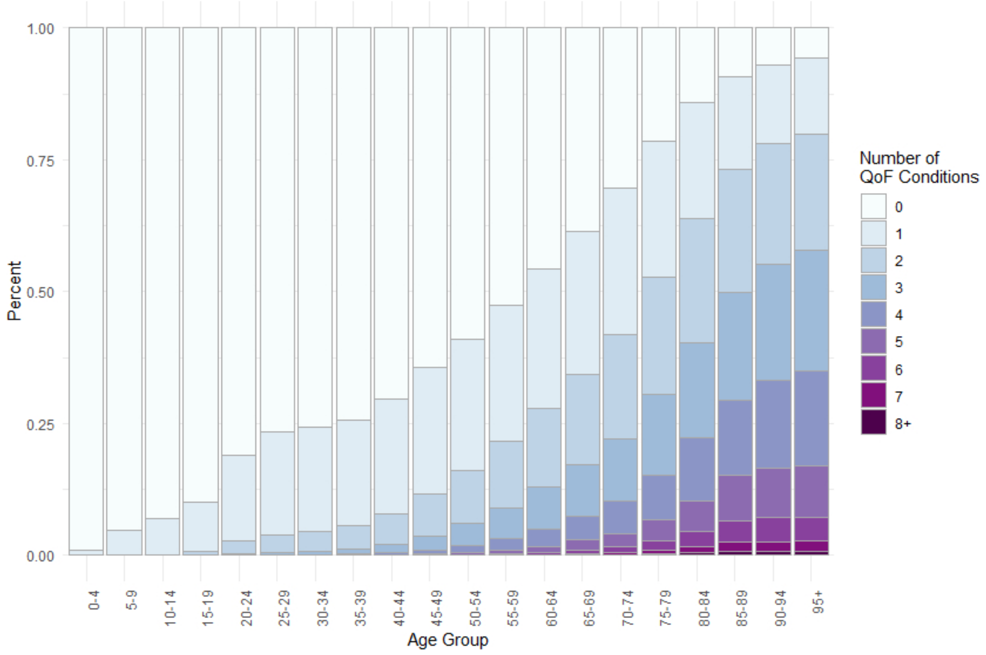

Figure 2. Number of long-term conditions by 5-year age band, with long-term conditions assessed through the Quality and Outcomes Framework (QOF) definitions.

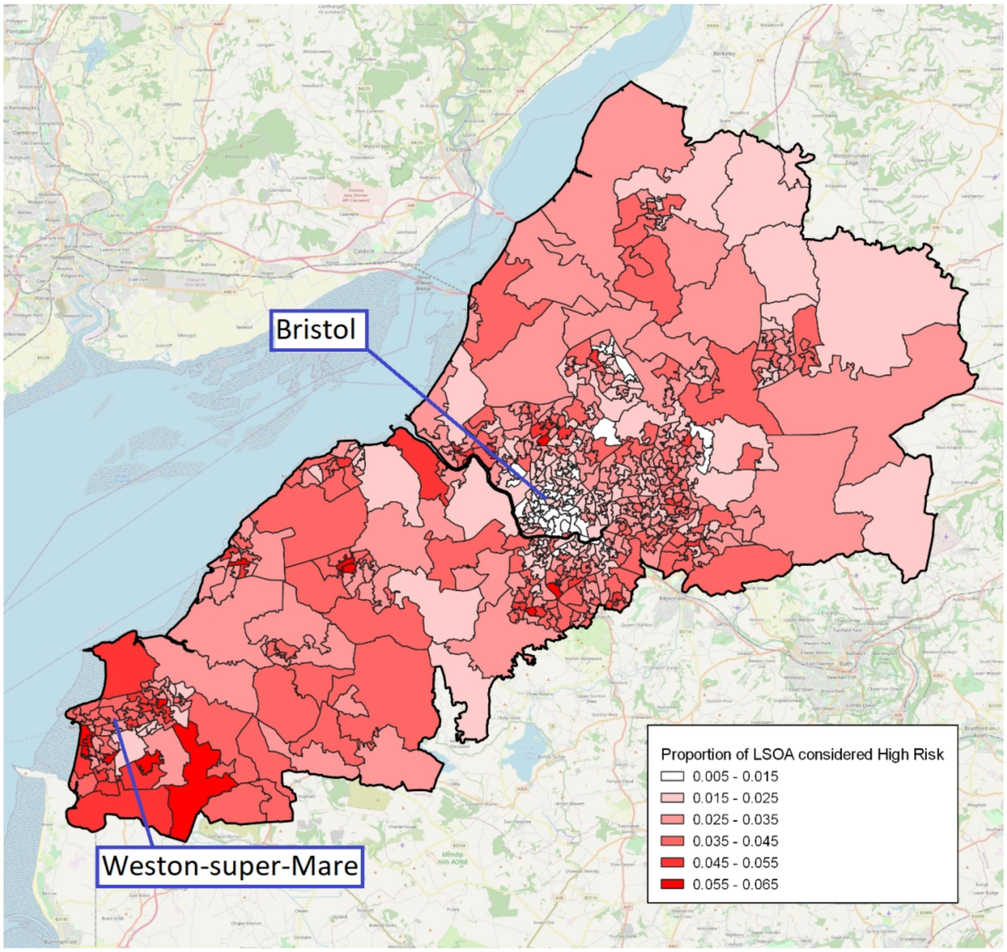

Figure 3. Geographical map of the Bristol, North Somerset and South Gloucestershire healthcare system illustrating the concentration of high-risk individuals at Lower Super Output Areas (LSOA) level.

Supplementary Material A: RECORD Statement

The RECORD statement – checklist of items, extended from the STROBE statement, that should be reported in observational studies using routinely collected health data.

	Item No.	STROBE items	Location in manuscript where items are reported	RECORD items	Location in manuscript where items are reported
**Title and abstract**					
	1	(a) Indicate the study's design with a commonly used term in the title or the abstract (b) Provide in the abstract an informative and balanced summary of what was done and what was found	Page 1 in title and abstract	RECORD 1.1: The type of data used should be specified in the title or abstract. When possible, the name of the databases used should be included.<br><br>RECORD 1.2: If applicable, the geographic region and timeframe within which the study took place should be reported in the title or abstract.<br><br>RECORD 1.3: If linkage between databases was conducted for the study, this should be clearly stated in the title or abstract.	1.1 Page 1 in the abstract.<br><br>1.2 Page 1 in the abstract.<br><br>1.3 Page 1 – data were linked in a system wide dataset.
**Introduction**					
Background rationale	2	Explain the scientific background and rationale for the investigation being reported	Page 3 under Introduction		
Objectives	3	State specific objectives, including any prespecified hypotheses	Page 3 at the bottom of the page.		
**Methods**					
Study Design	4	Present key elements of study design early in the paper	Page 4 under 2.1 Data, application and setting.		
Setting	5	Describe the setting, locations, and relevant dates, including periods of recruitment, exposure, follow-up, and data collection	Page 4		
Participants	6	*(a) Cohort study* - Give the eligibility criteria, and the sources and methods of selection of participants. Describe methods of follow-up<br>*Case-control study* - Give the eligibility criteria, and the sources and methods of case ascertainment and control selection. Give the rationale for the choice of cases and controls<br>*Cross-sectional study* - Give the eligibility criteria, and the sources and methods of selection of participants	Cross-sectional study – page 4 gives eligibility criteria and sources/method of selection.	RECORD 6.1: The methods of study population selection (such as codes or algorithms used to identify subjects) should be listed in detail. If this is not possible, an explanation should be provided.<br><br>RECORD 6.2: Any validation studies of the codes or algorithms used to select the population should be referenced. If validation was conducted for this study and not published elsewhere, detailed methods and results should be provided.<br><br>RECORD 6.3: If the study involved linkage of databases, consider use of a flow diagram or other graphical display to demonstrate the data linkage process, including the number	In this case all individuals within BNSSG registered to a contributing practice were included for analyses. Therefore codes to identify the overall study population were not used.<br><br>For the high-risk group population selection, all codes and criteria used are listed fully in Supplementary Material B and the methods described in section 2.2 (page 4).<br><br>Furthermore, the resulting list was compared with estimated expected numbers of high-risk individuals described in the Discussion under interpretation page 11.<br><br>The system-wide dataset at BNSSG CCG is already linked for analyses but a reference is included providing further

For peer review only - http://bmjopen.bmj.com/site/about/guidelines.xhtml

		(b) Cohort study - For matched studies, give matching criteria and number of exposed and unexposed *Case-control study* - For matched studies, give matching criteria and the number of controls per case		of individuals with linked data at each stage.	information to the dataset (Reference 25) and linking through a pseudonymised ID (unique individual identifier) was mentioned on page 4.
Variables	7	Clearly define all outcomes, exposures, predictors, potential confounders, and effect modifiers. Give diagnostic criteria, if applicable.	All outcomes and variables are fully defined including the use of algorithms and coding lists in Supplementary Material B for the high-risk criteria and Supplementary Material C for all clustering and non-clustering variables. Reference to the Supplementary material included on page 5 of the methods.	RECORD 7.1: A complete list of codes and algorithms used to classify exposures, outcomes, confounders, and effect modifiers should be provided. If these cannot be reported, an explanation should be provided.	All outcomes and variables are fully defined including the use of algorithms and coding lists in Supplementary Material B for the high-risk criteria and Supplementary Material C for all clustering and non-clustering variables.
Data sources/ measurement	8	For each variable of interest, give sources of data and details of methods of assessment (measurement). Describe comparability of assessment methods if there is more than one group	Table C.3 in Supplementary Material C.		
Bias	9	Describe any efforts to address potential sources of bias	To minimise misclassification and information bias, high risk criteria and coding lists were subject to double clinical review (page 5 Methods). The discussion page 10,11 discusses potential information bias as a limitation of using routine datasets.		
Study size	10	Explain how the study size was arrived at	Page 4 and 5		
Quantitative variables	11	Explain how quantitative variables were handled in the analyses. If applicable, describe which groupings were chosen, and why	How the variables were operationalised for analyses is detailed in Table C.3 Supplementary Material C.		
Statistical methods	12	(a) Describe all statistical methods, including those used to control for confounding<br>(b) Describe any methods used to examine subgroups and interactions<br>(c) Explain how missing data were addressed	a) Section 2.3 page 5. Further details on cluster analysis and selection of the number of clusters is included in Supplementary Material C.<br><br>b) Subgroups were investigated in Table		

For peer review only - http://bmjopen.bmj.com/site/about/guidelines.xhtml

		(d) *Cohort study* - If applicable, explain how loss to follow-up was addressed *Case-control study* - If applicable, explain how matching of cases and controls was addressed *Cross-sectional study* - If applicable, describe analytical methods taking account of sampling strategy (e) Describe any sensitivity analyses	1. Methods described on page 5.<br><br>c) Page 5<br><br>d) NA. The full BNSSG cohort from March 2019 was taken for analyses.<br><br>e) Sensitivity analyses in terms of the clustering solution chosen for segmentation is fully described in Supplementary Material C. Also described more briefly in the methods page 5.		
Data access and cleaning methods		..		RECORD 12.1: Authors should describe the extent to which the investigators had access to the database population used to create the study population.<br><br>RECORD 12.2: Authors should provide information on the data cleaning methods used in the study.	The full BNSSG database population for the SWD were used for analyses. This is included on page 4.<br><br>How variables were coded and operationalised is documented in Supplementary Material B and C and page 4 describes how a unique identifier is used to link data between the tables in the system wide dataset.
Linkage		..		RECORD 12.3: State whether the study included person-level, institutional-level, or other data linkage across two or more databases. The methods of linkage and methods of linkage quality evaluation should be provided.	Page 4 under section 2.1.
**Results**					
Participants	13	(a) Report the numbers of individuals at each stage of the study (*e.g.*, numbers potentially eligible, examined for eligibility, confirmed eligible, included in the study, completing follow-up, and analysed) (b) Give reasons for non-participation at each stage. (c) Consider use of a flow diagram	Page 6 for descriptive analyses. Page 8 for cluster analysis.	RECORD 13.1: Describe in detail the selection of the persons included in the study (*i.e.,* study population selection) including filtering based on data quality, data availability and linkage. The selection of included persons can be described in the text and/or by means of the study flow diagram.	Page 6 for descriptive analyses. Page 8 for cluster analysis.
Descriptive data	14	(a) Give characteristics of study participants (*e.g.*, demographic, clinical, social) and information on exposures and potential confounders (b) Indicate the number of participants with missing data for	Page 6 for descriptive analyses. Page 8 for cluster analysis.<br><br>Number of participants with missing data page 8.		

For peer review only - http://bmjopen.bmj.com/site/about/guidelines.xhtml

		each variable of interest<br>(c) *Cohort study* - summarise follow-up time (*e.g.*, average and total amount)			
Outcome data	15	*Cohort study* - Report numbers of outcome events or summary measures over time<br>*Case-control study* - Report numbers in each exposure category, or summary measures of exposure<br>*Cross-sectional study* - Report numbers of outcome events or summary measures	*Cross-sectional study* – Page 6, Table 1, Figure 1 and 2 report a comparative analysis of the low, moderate and high-risk groups with regard to demographic and socioeconomic information, historical healthcare utilisation, and burden of comorbidity. Figure 3 shows the geographical distribution of high risk individuals.		
Main results	16	(a) Give unadjusted estimates and, if applicable, confounder-adjusted estimates and their precision (e.g., 95% confidence interval). Make clear which confounders were adjusted for and why they were included<br>(b) Report category boundaries when continuous variables were categorized<br>(c) If relevant, consider translating estimates of relative risk into absolute risk for a meaningful time period	a) Main descriptive analyses reported on pages 6-8. Cluster analysis results reported on page 8-10.<br><br>b) Continuous variables kept as continuous and results reported as mean, median with interquartile range or proportion. Tables 1 and 2. Supplementary Material C discusses how variables are operationalised.<br><br>c) Not applicable.		
Other analyses	17	Report other analyses done—e.g., analyses of subgroups and interactions, and sensitivity analyses	Results are presented for low, moderate and high risk subgroups and for the segmentation analysis for clusters 1-6. Pages 6-8 for descriptive analyses and page 8-10 for cluster analyses. Sensitivity analyses in terms of the clustering solution were performed and documented in Supplementary Material C.		
**Discussion**					
Key results	18	Summarise key results with reference to study objectives	Page 10.		
Limitations	19	Discuss limitations of the study, taking into account sources of potential bias or imprecision. Discuss both direction and magnitude of any potential bias	Page 10-11.	RECORD 19.1: Discuss the implications of using data that were not created or collected to answer the specific research question(s). Include discussion of misclassification bias, unmeasured confounding, missing data, and changing eligibility over time, as they	Page 10-12 (i.e. misclassification bias and limitations relating to the data).

For peer review only

For peer review only - http://bmjopen.bmj.com/site/about/guidelines.xhtml

				pertain to the study being reported.	
Interpretation	20	Give a cautious overall interpretation of results considering objectives, limitations, multiplicity of analyses, results from similar studies, and other relevant evidence	Page 11-12.		
Generalisability	21	Discuss the generalisability (external validity) of the study results	Page 10-12.		
**Other Information**					
Funding	22	Give the source of funding and the role of the funders for the present study and, if applicable, for the original study on which the present article is based	Page 13.		
Accessibility of protocol, raw data, and programming code		..		RECORD 22.1: Authors should provide information on how to access any supplemental information such as the study protocol, raw data, or programming code.	Page 13. Technical appendix and coding lists available in online supplementary files. Local data used for the study are not publicly available.

*Reference: Benchimol EI, Smeeth L, Guttmann A, Harron K, Moher D, Petersen I, Sørensen HT, von Elm E, Langan SM, the RECORD Working Committee. The REporting of studies Conducted using Observational Routinely-collected health Data (RECORD) Statement. *PLoS Medicine* 2015; in press.

*Checklist is protected under Creative Commons Attribution (CC BY) license.

For peer review only

Supplementary Material B: High Risk Criteria Using BNSSG System Wide Dataset

1. High Risk Criteria Using BNSSG System Wide Dataset (SWD):

Local search terms are based on fields within the SWD. Disease search terms match to fields within the characteristics table of the SWD, which are based on READ code lists. These are either based the QOF business rules, indicated by 'qof_disease', or code sets published by the Cambridge CPRD group, (https://www.phpc.cam.ac.uk/pcu/research/research-groups/crmh/cprd_cam/codelists/) which were then clinically validated locally.

Category	Sub-category	Local search terms	Reference source
**Chronic liver disease, such as hepatitis**	Patients having undergone liver transplantation and taking immunosuppression	No local search, assumed picked up by NHS Digital central search.	https://www.bsg.org.uk/wp-content/uploads/2020/03/Hepatology-Risk-Groups-BSG-BASL-v2.0.pdf<br><br>https://www.bsg.org.uk/covid-19-advice/bsg-basl-advice-on-heptalogy-patient-risk-groups/
	Patients with autoimmune hepatitis taking immunosuppression	(cc_mild_liver_disease OR cc_moderate_or_severe_liver_disease) AND (IMMLIV OR IMMHiRS)	
	Any liver disease AND patients aged ≥70years of age	(cc_mild_liver_disease OR cc_moderate_or_severe_liver_disease) AND age >= 70 years	
	Any liver disease AND pregnancy	No local search, assumed picked up by NHS Digital central search.	
	Any liver disease AND patients with other co-morbidities: cardiac disease, respiratory disease, diabetes mellitus	(cc_mild_liver_disease OR cc_moderate_or_severe_liver_disease) AND (cc_myocardial_infarction OR cc_heart_failure OR qof_chd OR qof_hf OR cc_diabetes_no_complications OR cc_diabetes_end_complications OR qof_diabetes OR cc_ bronchiectasis OR cc_pulmonary_fibrosis OR ((cc_asthma OR qof_asthma) AND asthma products) OR cc_copd OR qof_copd)	
**Rheumatological conditions: Rheumatoid, inflammatory arthritis and other systemic inflammatory conditions.**	Corticosteroid dose of ≥20mg (0.5mg/kg) prednisolone (or equivalent) per day for more than four weeks	(inflam_arthritic OR qof_rheum) AND IMMHiRS	https://www.rheumatology.org.uk/News-Policy/Details/Covid19-Coronavirus-update-members

For peer review only - http://bmjopen.bmj.com/site/about/guidelines.xhtml

	Cyclophosphamide at any dose orally or within last six months IV	Cyclophosphamide >=1 within the last 6 months	
	Corticosteroid dose of ≥5mg prednisolone (or equivalent) per day for more than four weeks plus at least one other immunosuppressive medication*, biologic/monoclonal** or small molecule immunosuppressant (e.g. JAK inhibitors)***	IMMMoRS AND (IMMIMM OR IMMBIO)	
	Any two agents among immunosuppressive medications, biologics/monoclonals** or small molecule immunosuppressants with any co-morbidity****	any two separate drugs within: IMMIMM OR IMMBIO	
**Inflammatory bowel disease (IBD)**	AND a co-morbidity (respiratory, cardiac, hypertension or diabetes mellitus) and/or are ≥70 years old AND one of: Ustekinumab; Vedolizumab; Methotrexate; Anti-TNF alpha monotherapy (infliximab, adalimumab, golimumab); Thiopurines (azathioprine, mercaptopurine, tioguanine); Calcineurin inhibitors (tacrolimus or ciclosporin); Janus kinase (JAK) inhibition (tofacitinib); Combination therapy in stable patients**; Immunosuppressive/biologic trial medication	(age >=70 years OR cc_diabetes_no_complications OR cc_diabetes_end_complications OR qof_diabetes OR qof_ht OR cc_ bronchiectasis OR cc_pulmonary_fibrosis OR cc_copd OR ((qof_asthma OR cc_asthma) AND asthma products) OR qof_copd OR cc_cystic_fibrosis OR cc_myocardial_infarction OR cc_heart_failure OR qof_chd OR qof_hf OR (cc_egfr <30)) AND (IMMIMM OR IMMBIO)	https://www.bsg.org.uk/wp-content/uploads/2020/03/Hepatology-Risk-Groups-BSG-BASL-v2.0.pdf

For peer review only - http://bmjopen.bmj.com/site/about/guidelines.xhtml

	AND who meet one or more of the following criteria: on oral or intravenous prednisolone ≥20 mg per day (only while on this dose); new induction therapy with combo therapy (starting biologic within previous 6 weeks); moderate-to-severely active disease despite immunosuppression/ biologics; short gut syndrome requiring nutritional support; requirement for parenteral nutrition	ibd AND (IMMHiRS OR (first IMMBIO <started within most recent 6 weeks AND (IMMIMM OR IMMMoRS OR IMMHiRS)	
**Solid organ transplant recipients**		No local search, assumed picked up by NHS Digital central search.	https://digital.nhs.uk/coronavirus/shielded-patient-list/methodology
**Cancer**	active chemotherapy	(cancer_lung_year OR cancer_breast_year OR cancer_bowel_year OR cancer_prostate_year OR cancer_cervical_year OR cancer_ovarian_year OR cancer_melanoma_year OR cancer_headneck_year OR cancer_giliver_year OR cancer_other_year OR cancer_bladder_year OR cancer_kidney_year OR cancer_metase_year) AND any of the 'inpatient or outpatient oncology' codes occuring >=3 times in the past year	https://digital.nhs.uk/coronavirus/shielded-patient-list/methodology
	radical radiotherapy for lung cancer	Covered by other categories	
	leukaemia, lymphoma or myeloma who are at any stage of treatment	cancer_leuklymph_year	
	immunotherapy or other continuing antibody treatments for cancer	Covered by other categories	
	other targeted cancer treatments which can affect the immune system, such as protein kinase inhibitors or PARP inhibitors.	Covered by other categories	

For peer review only

For peer review only - http://bmjopen.bmj.com/site/about/guidelines.xhtml

	bone marrow or stem cell transplants in the last 6 months, or who are still taking immunosuppression drugs.	('blood and marrow transplantation code' EVER AND (at least one of: azathioprine, Leflunomide, methotrexate, mycophenolate mofetil or mycophenolic acid, ciclosporin, cyclophosphamide, tacrolimus, sirolimus, Rituximab prescribed in the last 6 months)) OR 'blood and marrow transplantation code' in the last 6 months.	
**Severe respiratory conditions**	Severe asthma	((qof_asthma OR cc_asthma) AND asthma product codes)) AND (4 or more prescriptions for prednisolone in last 6 months of 2019) **OR** Omalizumab with >=1 in most revent 6 months)	https://digital.nhs.uk/coronavirus/shielded-patient-list/methodology, plus local meds op advice
	Patients with Chronic Obstructive Pulmonary Disease (COPD) who have required hospitalisation in the last 12 months or patients who have required 2 or more courses of steroids and/or antibiotics in the last 12 months	(LAMA AND LABA AND ICS) OR home_oxygen OR ((qof_copd OR cc_copd) AND (prescribed Roflumilast (<6 months) OR fev1 <50% predicted OR MRC dyspnoea score 4-5))	
	Severe bronchiectasis	cc_bronchiectasis AND (MRC dyspnoea score 5 OR >=2 in-patient respiratory code in the last 12 months OR fev1 <30% predicted OR age >80 years)	https://www.bronchiectasis.eu/severity-assessment, http://www.bronchiectasisseverity.com/15-2/, https://thorax.bmj.com/content/74/Suppl_1/1
	Interstitial lung diseases	lung_restrict	https://www.brit-thoracic.org.uk/media/455101/bts-management-advice-for-ild-patients-v10-23-march-2020.pdf
	Cystic fibrosis	cc_cystic_fibrosis	https://digital.nhs.uk/coronavirus/shielded-patient-list/methodology

For peer review only

For peer review only - http://bmjopen.bmj.com/site/about/guidelines.xhtml

**Rare diseases and inborn errors of metabolism that significantly increase the risk of infections (such as SCID, homozygous sickle cell disease)**	Homozygous sickle cell disease	No local search, assumed picked up by NHS Digital central search.	https://digital.nhs.uk/coronavirus/shielded-patient-list/methodology
**Rare diseases and inborn errors of metabolism that significantly increase the risk of infections (such as SCID, homozygous sickle cell disease)**	Other including SCID	No local search, assumed picked up by NHS Digital central search.	
**People on immunosuppression therapies sufficient to significantly increase risk of infection**		Covered by other categories	https://digital.nhs.uk/coronavirus/shielded-patient-list/methodology
**Pregnant**	AND with significant heart disease, congenital or acquired	No local search, assumed picked up by NHS Digital central search.	https://digital.nhs.uk/coronavirus/shielded-patient-list/methodology

Gastroenterology Notes
No specific recommendations are being made regarding IBD and pregnancy, and pregnant women with IBD are encouraged to follow the guidance available from the UK government for pregnant women in the general population.
* i.e. at least one of (comorbidity listed above or age≥70) plus at least one therapy from the middle column ** Combination therapy may increase risk over monotherapy
These guidelines were formulated by the UK IBD COVID-19 working group on 20/03/2020 and were based on expert opinion and the available evidence at the time. Column headings updated 22/03/2020 to reflect latest government terminology. but there is no specific evidence for this situation
Rheumatology Notes
*Immunosuppressive medications include: Azathioprine, Leflunomide, methotrexate, Mycophenolate (mycophenolate mofetil or mycophenolic acid), ciclosporin, cyclophosphamide, tacrolimus, sirolimus. It does NOT include Hydroxychloroquine or Sulphasalazine either alone or in combination
** Biologic/monoclonal includes: Rituximab within last 12 months; all anti-TNF drugs (etanercept, adalimumab, infliximab, golimumab, certolizumab and biosimilar variants of all of these); Tociluzimab; Abatacept; Belimumab; Anakinra; Seukinumab; Ixekizumab; Ustekinumab; Sarilumumab; canakinumab
*** Small molecules includes: all JAK inhibitors – baracitinib, tofacitinib etc
**** Co-morbidity includes: age >70, Diabetes Mellitus, any pre-existing lung disease, renal impairment, any history of Ischaemic Heart Disease or hypertension NB This advice applies to both adults, children and young people with rheumatic disease. We do NOT advise that patients increase steroid dose if they become unwell
IMMIMM – at least one in the last 6 months
IMMLIV – at least one in the last 6 months
Roflumilast – at least one in the last 6 months

For peer review only - http://bmjopen.bmj.com/site/about/guidelines.xhtml

2. Prescriptions/drug lists to accompany the criteria above

Drug Name	Drug Type	Drug List		Temporal Aspect
**IMMIMM**	Immunosuppressant/immunomodulator	**Immunosuppressant/immunomodulator**	**Drug**	At least one prescription in the last 6 months
		Azathioprine	Thiopurines	
		mercaptopurine	Thiopurines	
		Tioguanine	Thiopurines	
		Leflunomide	Other	
		Thalidomide	Other	
		Methotrexate	Other	
		mycophenolate mofetil	Other	
		mycophenolic acid	Other	
		cyclophosphamide	Other	
		Tacrolimus	Calcineurin inhibitors	
		sirolimus	Calcineurin inhibitors	
		ciclosporin	Calcineurin inhibitors	
**IMMLIV**	Liver Transplant Drugs	Azathioprine<br>Mycophenolate Mofetil<br>Mycophenolic<br>Ciclosporin<br>Sirolimus<br>Tacrolimus		At least one prescription in the last 6 months
**IMMHiRS**	High Risk Steroids	Prednisolone AND (20mg OR 25mg OR 30mg)<br>Dexamethasone AND >=4mg AND tablets<br>Methylprednisolone AND (16mg OR 100mg OR injection)		2 or more in the past 3 months
**IMMMoRS**	Moderate Risk Steroids	Prednisolone AND 5mg<br>Betamethasone AND tablets<br>Deflazacort<br>Dexamethasone AND tablets AND <4mg<br>Hydrocortisone AND tablets and 20mg<br>Methylprednisolone AND (2mg OR 4mg)		2 or more in the past 3 months
**IMMBIO**	Biologic/monoclonal	**Biologic/monoclonal**	**Drug**	Rituximab one or more in the past 12 months<br><br>All other drugs one or more in the past 6 months
		rituximab	Other	
		tociluzimab	Other	
		abatacept	Other	
		belimumab	Other	
		anakinra	Other	
		seukinumab	Other	
		ixekizumab	Other	
		ustekinumab	Other	
		sarilumumab	Other	
		canakinumab	Other	
		etanercept	Anti-TNF	
		adalimumab	Anti-TNF	
		infliximab	Anti-TNF	
		golimumab	Anti-TNF	
		certolizumab	Anti-TNF	
		baricitinib	JAK_inhibitors	
		tofacitinib	JAK_inhibitors	
		vedolizumab	Other	
		apremilast	Other	
		brodalumab	Other	
		dupilumab	Other	
		guselkumab	Other	
**IMMBIOAST**	Biologic asthma	Omalizumab		One or more in the past 6 months
**Asthma COPD Products**	Asthma and COPD product name list	Product name list given below along with a label for LAMA, LABA and ICS.		One or more in the past 6 months
**Other drugs requiring monitoring in previous 2 months**	Mixed	Amiodarone<br>Flupentixol<br>Zuclopentixol<br>Haloperidol<br>Pipotiazine<br>Fluphenazine<br>Risperidone<br>Risperdal<br>Paliperidone<br>Olanzapine		One or more in previous 2 months

For peer review only

For peer review only - http://bmjopen.bmj.com/site/about/guidelines.xhtml

		Aripiprazole<br>Carbamazepine<br>Cinacalcet<br>Denosumab<br>Dexamfetamine<br>Entecavir<br>Guanfacine<br>Hydroxycarbamide<br>Lamivudine<br>Liothyronine<br>Lisdexamfetamine<br>Lithium<br>Mercaptopurine<br>Mesalazine<br>Metyrapone<br>Mexiletine<br>Modafinil<br>Penicillamine<br>Riluzole<br>Tenofovir<br>Tinzaparin<br>Enozaparin<br>Warfarin<br>Dalteparin<br>Valproic Acid<br>Sodium Valproate<br>Venlafaxine	

3. Product name list for Asthma and COPD drugs along with the type of medicine (LAMA, LABA or ICS) and their combinations

#	**Product Name**	**Type Label**
1	Accolate 20mg tablets (AstraZeneca UK Ltd)	nil
2	Aclidinium brom 396mcg/dose / Formoterol 11.8mcg/dose DP inh	LAMA_LABA
3	Aclidinium bromide 375micrograms/dose dry powder inhaler	LAMA
4	Aclidinium bromide 396micrograms/dose / Formoterol 11.8micrograms/dose dry powder inhaler	LAMA_LABA
5	Aclidinium bromide 396micrograms/dose / Formoterol 11.8micrograms/dose dry powder inhaler (Colorama Pharmaceuticals Ltd)	LAMA_LABA
6	Adrenaline and Atropine comp spray	other
7	Adrenaline and atropine inhalation solution	other
8	Aerivio Spiromax 50microg / 500microg/dose dry pdr inh	ICS_LABA
9	Aerivio Spiromax 50micrograms/dose / 500micrograms/dose dry powder inhaler (Teva UK Ltd)	ICS_LABA
10	AeroBec 100 Autohaler (Meda Pharmaceuticals Ltd)	nil
11	Aerobec 250microgram/actuation Pressurised inhalation (Meda Pharmaceuticals Ltd)	nil
12	AeroBec 50 Autohaler (Meda Pharmaceuticals Ltd)	nil
13	AeroBec Forte 250 Autohaler (Meda Pharmaceuticals Ltd)	nil
14	Aerolin 400 100microgram/actuation Inhalation powder (3M Health Care Ltd)	ICS
15	Aerolin autohaler 100microgram/actuation Pressurised inhalation (3M Health Care Ltd)	ICS
16	AirFluSal 25micrograms/dose / 125micrograms/dose inhaler	ICS_LABA
17	AirFluSal 25micrograms/dose / 250micrograms/dose inhaler	ICS_LABA
18	AirFluSal 25micrograms/dose / 250micrograms/dose inhaler (Sandoz Ltd)	ICS_LABA
19	AirFluSal Forspiro 50microg / 500microg/dose dry pdr inh	ICS_LABA
20	AirFluSal Forspiro 50micrograms/dose / 500micrograms/dose dry powder inhaler (Sandoz Ltd)	ICS_LABA
21	Airomir 100micrograms/dose Autohaler (Teva UK Ltd)	SABA
22	Airomir 100micrograms/dose inhaler (Teva UK Ltd)	SABA
23	AirSalb 100micrograms/dose inhaler CFC free (Sandoz Ltd)	SABA
24	Aloflute 25micrograms/dose / 125micrograms/dose inhaler	ICS_LABA
25	Aloflute 25micrograms/dose / 250micrograms/dose inhaler	ICS_LABA

For peer review only

For peer review only - http://bmjopen.bmj.com/site/about/guidelines.xhtml

26	Alupent 750microgram/inhalation Aerosol refill (Boehringer Ingelheim Ltd)	nil
27	Alupent 750microgram/inhalation Inhalation powder (Boehringer Ingelheim Ltd)	nil
28	Alupent expectorant Mixture (Boehringer Ingelheim Ltd)	nil
29	Alupent expectorant Tablet (Boehringer Ingelheim Ltd)	nil
30	Alvesco 160 inhaler	ICS
31	Alvesco 160 inhaler (AstraZeneca UK Ltd)	ICS
32	Alvesco 80 inhaler	ICS
33	Alvesco 80 inhaler (AstraZeneca UK Ltd)	ICS
34	Aminophylline 100mg modified-release tablets	other
35	Aminophylline 200mg tablets	other
36	Aminophylline 225mg Modified-release tablet (Actavis UK Ltd)	other
37	Aminophylline 225mg Modified-release tablet (Hillcross Pharmaceuticals Ltd)	other
38	Aminophylline 250mg/10ml injection	other
39	Aminophylline 250mg/10ml solution for injection ampoules	other
40	Aminophylline 250mg/10ml solution for injection ampoules (A A H Pharmaceuticals Ltd)	other
41	Aminophylline 250mg/10ml solution for injection ampoules (AMCo)	other
42	Aminophylline 250mg/10ml solution for injection ampoules (Martindale Pharmaceuticals Ltd)	other
43	Aminophylline 250mg/ml injection	other
44	Aminophylline 25mg/ml Injection (Celltech Pharma Europe Ltd)	other
45	Aminophylline 350mg modified-release tablets	other
46	Aminophylline 360mg suppositories	other
47	Aminophylline 360mg suppositories (Special Order)	other
48	Aminophylline hydrate 225mg modified-release tablets	other
49	Aminophylline hydrate 350mg modified-release tablets	other
50	Aminophylline sr 225mg Modified-release tablet (IVAX Pharmaceuticals UK Ltd)	other
51	Anoro Ellipta 55microg/dose / 22microg/dose dry pdr inh	LAMA_LABA
52	Anoro Ellipta 55micrograms/dose / 22micrograms/dose dry powder inhaler (GlaxoSmithKline UK Ltd)	LAMA_LABA
53	Asmabec 100 Clickhaler	ICS
54	Asmabec 100 Clickhaler (Focus Pharmaceuticals Ltd)	ICS
55	Asmabec 100microgram/actuation Spacehaler (Celltech Pharma Europe Ltd)	ICS
56	Asmabec 250 Clickhaler (Focus Pharmaceuticals Ltd)	ICS
57	Asmabec 250microgram/actuation Spacehaler (Celltech Pharma Europe Ltd)	ICS
58	Asmabec 50 Clickhaler (Focus Pharmaceuticals Ltd)	ICS
59	Asmabec 50microgram/actuation Spacehaler (Celltech Pharma Europe Ltd)	ICS
60	Asmanex 200micrograms/dose Twisthaler	ICS
61	Asmanex 200micrograms/dose Twisthaler (Merck Sharp & Dohme Ltd)	ICS
62	Asmanex 400micrograms/dose Twisthaler	ICS
63	Asmanex 400micrograms/dose Twisthaler (Merck Sharp & Dohme Ltd)	ICS
64	Asmasal 100microgram/inhalation Spacehaler (Celltech Pharma Europe Ltd)	nil
65	Asmasal 95micrograms/dose Clickhaler (Focus Pharmaceuticals Ltd)	nil
66	Asmaven 100microgram Inhalation powder (Berk Pharmaceuticals Ltd)	nil
67	Asmavent 100micrograms/dose inhaler CFC free (Kent Pharmaceuticals Ltd)	nil
68	Atimos Modulite 12micrograms/dose inhaler	LABA
69	Atimos Modulite 12micrograms/dose inhaler (Chiesi Ltd)	LABA
70	Atropine methonitrate with papaverine and adrenaline inhalation	other
71	Atrovent 20micrograms/dose Autohaler (Boehringer Ingelheim Ltd)	ipratropium

For peer review only - http://bmjopen.bmj.com/site/about/guidelines.xhtml

72	Atrovent 20micrograms/dose inhaler (Boehringer Ingelheim Ltd)	ipratropium
73	Atrovent 20micrograms/dose inhaler CFC free (Boehringer Ingelheim Ltd)	ipratropium
74	Atrovent 20micrograms/dose inhaler CFC free (DE Pharmaceuticals)	ipratropium
75	Atrovent 20micrograms/dose inhaler CFC free (Lexon (UK) Ltd)	ipratropium
76	Atrovent 20micrograms/dose inhaler CFC free (Sigma Pharmaceuticals Plc)	ipratropium
77	Atrovent 250micrograms/1ml nebuliser liquid UDVs (Boehringer Ingelheim Ltd)	ipratropium
78	Atrovent 40microgram Aerocaps (Boehringer Ingelheim Ltd)	ipratropium
79	Atrovent 40microgram Aerocaps with Aerohaler (Boehringer Ingelheim Ltd)	ipratropium
80	Atrovent 500micrograms/2ml nebuliser liquid UDVs (Boehringer Ingelheim Ltd)	ipratropium
81	Atrovent 500micrograms/2ml nebuliser liquid UDVs (Waymade Healthcare Plc)	ipratropium
82	Atrovent aerocaps 40microgram Inhalation powder (Boehringer Ingelheim Ltd)	ipratropium
83	Atrovent aerohaler 40microgram Inhalation powder (Boehringer Ingelheim Ltd)	ipratropium
84	Atrovent Forte 40micrograms/dose inhaler (Boehringer Ingelheim Ltd)	ipratropium
85	Atrovent udv 0.25mg/ml Nebuliser liquid (Boehringer Ingelheim Ltd)	ipratropium
86	Bambec 10mg tablets	LABA
87	Bambec 10mg tablets (AstraZeneca UK Ltd)	LABA
88	Bambec 20mg tablets	LABA
89	Bambec 20mg tablets (AstraZeneca UK Ltd)	LABA
90	Bambuterol 10mg tablets	LABA
91	Bambuterol 20mg tablets	LABA
92	Bdp 100microgram/actuation Spacehaler (Celltech Pharma Europe Ltd)	ICS
93	Bdp 250microgram/actuation Spacehaler (Celltech Pharma Europe Ltd)	ICS
94	Bdp 50microgram/actuation Spacehaler (Celltech Pharma Europe Ltd)	ICS
95	Beclazone 100 Easi-Breathe inhaler (Teva UK Ltd)	ICS
96	Beclazone 100 inhaler	ICS
97	Beclazone 100 inhaler (Teva UK Ltd)	ICS
98	Beclazone 100microgram/actuation Inhalation powder (Actavis UK Ltd)	ICS
99	Beclazone 200 inhaler	ICS
100	Beclazone 200 inhaler (Teva UK Ltd)	ICS
101	Beclazone 250 Easi-Breathe inhaler (Teva UK Ltd)	ICS
102	Beclazone 250 inhaler	ICS
103	Beclazone 250 inhaler (Teva UK Ltd)	ICS
104	Beclazone 250microgram/actuation Inhalation powder (Actavis UK Ltd)	ICS
105	Beclazone 50 Easi-Breathe inhaler	ICS
106	Beclazone 50 Easi-Breathe inhaler (Teva UK Ltd)	ICS
107	Beclazone 50 inhaler (Teva UK Ltd)	ICS
108	Beclazone 50microgram/actuation Inhalation powder (Actavis UK Ltd)	ICS
109	Beclazone easi-breathe (roi) 100microgram/actuation Pressurised inhalation (Ivax Pharmaceuticals Ireland)	ICS
110	Beclazone easi-breathe (roi) 250microgram/actuation Pressurised inhalation (Ivax Pharmaceuticals Ireland)	ICS
111	Becloforte 250micrograms/dose inhaler (Dowelhurst Ltd)	ICS
112	Becloforte 250micrograms/dose inhaler (GlaxoSmithKline UK Ltd)	ICS
113	Becloforte 400microgram disks (GlaxoSmithKline UK Ltd)	ICS
114	Becloforte 400microgram disks with Diskhaler (GlaxoSmithKline UK Ltd)	ICS
115	Becloforte easi-breathe 250microgram/actuation Pressurised inhalation (Allen & Hanburys Ltd)	ICS
116	Becloforte integra 250microgram/actuation Inhaler with compact spacer (Glaxo Laboratories Ltd)	ICS

For peer review only

For peer review only - http://bmjopen.bmj.com/site/about/guidelines.xhtml

117	Becloforte vm 250microgram/actuation VM pack (Allen & Hanburys Ltd)	ICS
118	Beclometasone 100 micrograms/actuation vortex inhaler	ICS
119	Beclometasone 100mcg/dose breath actuated inhaler CFC free	ICS
120	Beclometasone 100microg/Formoterol 6microg/dose dry pdr inh	ICS_LABA
121	Beclometasone 100microg/Formoterol 6microg/dose inh CFCfree	ICS_LABA
122	Beclometasone 100microgram inhalation powder blisters	ICS
123	Beclometasone 100microgram inhalation powder blisters with device	ICS
124	Beclometasone 100microgram inhalation powder capsules	ICS
125	Beclometasone 100microgram/actuation Inhalation powder (Actavis UK Ltd)	ICS
126	Beclometasone 100microgram/actuation Inhalation powder (Neo Laboratories Ltd)	ICS
127	Beclometasone 100microgram/actuation Pressurised inhalation (Approved Prescription Services Ltd)	ICS
128	Beclometasone 100micrograms disc	ICS
129	Beclometasone 100micrograms with Salbutamol 200micrograms inhalation capsules	ICS_SABA
130	Beclometasone 100micrograms/actuation extrafine particle cfc free inhaler	ICS
131	Beclometasone 100micrograms/dose / Formoterol 6micrograms/dose dry powder inhaler	ICS_LABA
132	Beclometasone 100micrograms/dose / Formoterol 6micrograms/dose inhaler CFC free	ICS_LABA
133	Beclometasone 100micrograms/dose breath actuated inhaler	ICS
134	Beclometasone 100micrograms/dose breath actuated inhaler CFC free	ICS
135	Beclometasone 100micrograms/dose dry powder inhaler	ICS
136	Beclometasone 100micrograms/dose inhaler	ICS
137	Beclometasone 100micrograms/dose inhaler (A A H Pharmaceuticals Ltd)	ICS
138	Beclometasone 100micrograms/dose inhaler (Mylan)	ICS
139	Beclometasone 100micrograms/dose inhaler (Teva UK Ltd)	ICS
140	Beclometasone 100micrograms/dose inhaler CFC free	ICS
141	Beclometasone 100micrograms/dose inhaler CFC free (Ennogen Healthcare Ltd)	ICS
142	Beclometasone 200 Cyclocaps (Teva UK Ltd)	ICS
143	Beclometasone 200microg/Formoterol 6microg/dose dry pdr inh	ICS_LABA
144	Beclometasone 200microg/Formoterol 6microg/dose inh CFC free	ICS_LABA
145	Beclometasone 200microgram inhalation powder blisters	ICS
146	Beclometasone 200microgram inhalation powder blisters with device	ICS
147	Beclometasone 200microgram inhalation powder capsules	ICS
148	Beclometasone 200micrograms disc	ICS
149	Beclometasone 200micrograms with Salbutamol 400micrograms inhalation capsules	ICS_SABA
150	Beclometasone 200micrograms/dose / Formoterol 6micrograms/dose dry powder inhaler	ICS_LABA
151	Beclometasone 200micrograms/dose / Formoterol 6micrograms/dose inhaler CFC free	ICS_LABA
152	Beclometasone 200micrograms/dose dry powder inhaler	ICS
153	Beclometasone 200micrograms/dose inhaler	ICS
154	Beclometasone 200micrograms/dose inhaler (A A H Pharmaceuticals Ltd)	ICS
155	Beclometasone 200micrograms/dose inhaler CFC free	ICS
156	Beclometasone 250microgram/actuation Inhalation powder (Actavis UK Ltd)	ICS
157	Beclometasone 250microgram/actuation Inhalation powder (Neo Laboratories Ltd)	ICS
158	Beclometasone 250microgram/actuation Pressurised inhalation (Approved Prescription Services Ltd)	ICS
159	Beclometasone 250micrograms/actuation inhaler and compact spacer	ICS
160	Beclometasone 250micrograms/actuation vortex inhaler	ICS
161	Beclometasone 250micrograms/dose breath actuated inhaler	ICS
162	Beclometasone 250micrograms/dose dry powder inhaler	ICS

For peer review only

163	Beclometasone 250micrograms/dose inhaler	ICS
164	Beclometasone 250micrograms/dose inhaler (A A H Pharmaceuticals Ltd)	ICS
165	Beclometasone 250micrograms/dose inhaler (Mylan)	ICS
166	Beclometasone 250micrograms/dose inhaler (Teva UK Ltd)	ICS
167	Beclometasone 250micrograms/dose inhaler CFC free	ICS
168	Beclometasone 400 Cyclocaps (Teva UK Ltd)	ICS
169	Beclometasone 400microgram disc	ICS
170	Beclometasone 400microgram inhalation powder blisters	ICS
171	Beclometasone 400microgram inhalation powder blisters with device	ICS
172	Beclometasone 400microgram inhalation powder capsules	ICS
173	Beclometasone 400micrograms/actuation inhaler	ICS
174	Beclometasone 400micrograms/dose dry powder inhaler	ICS
175	Beclometasone 50microgram/actuation Inhalation powder (Actavis UK Ltd)	ICS
176	Beclometasone 50microgram/actuation Inhalation powder (Neo Laboratories Ltd)	ICS
177	Beclometasone 50microgram/actuation Pressurised inhalation (Approved Prescription Services Ltd)	ICS
178	Beclometasone 50micrograms with salbutamol 100micrograms/inhalation inhaler	ICS_SABA
179	Beclometasone 50micrograms/actuation extrafine particle cfc free inhaler	ICS
180	Beclometasone 50micrograms/dose breath actuated inh CFC free	ICS
181	Beclometasone 50micrograms/dose breath actuated inhaler	ICS
182	Beclometasone 50micrograms/dose breath actuated inhaler CFC free	ICS
183	Beclometasone 50micrograms/dose dry powder inhaler	ICS
184	Beclometasone 50micrograms/dose inhaler	ICS
185	Beclometasone 50micrograms/dose inhaler (A A H Pharmaceuticals Ltd)	ICS
186	Beclometasone 50micrograms/dose inhaler (Almus Pharmaceuticals Ltd)	ICS
187	Beclometasone 50micrograms/dose inhaler (Mylan)	ICS
188	Beclometasone 50micrograms/dose inhaler (Teva UK Ltd)	ICS
189	Beclometasone 50micrograms/dose inhaler CFC free	ICS
190	Beclometasone 50micrograms/ml nebuliser suspension	ICS
191	Beclometasons 50 micrograms/actuation vortex inhaler	ICS
192	BECLOMETHASONE /SALBUTAMOL	ICS_SABA
193	Becodisks 100microgram	ICS
194	Becodisks 100microgram (GlaxoSmithKline UK Ltd)	ICS
195	Becodisks 100microgram Disc (Allen & Hanburys Ltd)	ICS
196	Becodisks 100microgram with Diskhaler (GlaxoSmithKline UK Ltd)	ICS
197	Becodisks 200microgram (GlaxoSmithKline UK Ltd)	ICS
198	Becodisks 200microgram (Lexon (UK) Ltd)	ICS
199	Becodisks 200microgram (Mawdsley-Brooks & Company Ltd)	ICS
200	Becodisks 200microgram Disc (Allen & Hanburys Ltd)	ICS
201	Becodisks 200microgram with Diskhaler (GlaxoSmithKline UK Ltd)	ICS
202	Becodisks 400microgram (GlaxoSmithKline UK Ltd)	ICS
203	Becodisks 400microgram (Waymade Healthcare Plc)	ICS
204	Becodisks 400microgram Disc (Allen & Hanburys Ltd)	ICS
205	Becodisks 400microgram with Diskhaler (GlaxoSmithKline UK Ltd)	ICS
206	BECOTIDE 100	ICS
207	Becotide 100 inhaler (GlaxoSmithKline UK Ltd)	ICS
208	Becotide 100 inhaler (Waymade Healthcare Plc)	ICS

For peer review only - http://bmjopen.bmj.com/site/about/guidelines.xhtml

209	Becotide 100microgram Rotacaps (GlaxoSmithKline UK Ltd)	ICS
210	Becotide 200 inhaler (GlaxoSmithKline UK Ltd)	ICS
211	Becotide 200microgram Rotacaps (GlaxoSmithKline UK Ltd)	ICS
212	Becotide 400microgram Rotacaps (GlaxoSmithKline UK Ltd)	ICS
213	BECOTIDE 50	ICS
214	Becotide 50 inhaler (Dowelhurst Ltd)	ICS
215	Becotide 50 inhaler (GlaxoSmithKline UK Ltd)	ICS
216	Becotide 50microgram/ml Nebuliser liquid (Allen & Hanburys Ltd)	ICS
217	Becotide easi-breathe 100microgram/actuation Pressurised inhalation (Allen & Hanburys Ltd)	ICS
218	Becotide easi-breathe 50microgram/actuation Pressurised inhalation (Allen & Hanburys Ltd)	ICS
219	BECOTIDE FOR NEBULISER	ICS
220	BECOTIDE ROTACAPS	ICS
221	Becotide Rotahaler (GlaxoSmithKline UK Ltd)	ICS
222	BECOTIDE SUSP FOR NEBULISATION	ICS
223	Berotec 100microgram/actuation Inhalation powder (Boehringer Ingelheim Ltd)	nil
224	Berotec 5mg/ml Nebuliser liquid (Boehringer Ingelheim Ltd)	nil
225	Betamethasone valerate 100micrograms/actuation inhaler	ICS
226	Bextasol Inhalation powder (Allen & Hanburys Ltd)	ICS
227	Biophylline 125mg/5ml Oral solution (Lorex Synthelabo Ltd)	other
228	Biophylline 350mg Tablet (Lorex Synthelabo Ltd)	other
229	Biophylline 500mg Tablet (Lorex Synthelabo Ltd)	other
230	Braltus 10microgram inhalation pdr caps with Zonda inhaler	LAMA
231	Braltus 10microgram inhalation powder capsules with Zonda inhaler (Teva UK Ltd)	LAMA
232	Brelomax 2mg Tablet (Abbott Laboratories Ltd)	nil
233	Bricanyl 1.5mg/5ml syrup (AstraZeneca UK Ltd)	SABA
234	Bricanyl 2.5mg/5ml solution for injection ampoules (AstraZeneca UK Ltd)	SABA
235	Bricanyl 500microgram/ml Injection (AstraZeneca UK Ltd)	SABA
236	Bricanyl 500micrograms/1ml solution for injection ampoules (AstraZeneca UK Ltd)	SABA
237	Bricanyl 500micrograms/dose Turbohaler (AstraZeneca UK Ltd)	SABA
238	Bricanyl 500micrograms/dose Turbohaler (DE Pharmaceuticals)	SABA
239	Bricanyl 500micrograms/dose Turbohaler (Necessity Supplies Ltd)	SABA
240	Bricanyl 500micrograms/dose Turbohaler (Waymade Healthcare Plc)	SABA
241	Bricanyl 5mg tablets (AstraZeneca UK Ltd)	SABA
242	Bricanyl 5mg/2ml Nebuliser liquid (AstraZeneca UK Ltd)	SABA
243	Bricanyl 5mg/2ml Respules (AstraZeneca UK Ltd)	SABA
244	Bricanyl Oral solution (AstraZeneca UK Ltd)	SABA
245	Bricanyl Refill canister (AstraZeneca UK Ltd)	SABA
246	Bricanyl Tablet (AstraZeneca UK Ltd)	SABA
247	Bricanyl turbohaler 500 500microgram Turbohaler (AstraZeneca UK Ltd)	SABA
248	Bronchilator Aerosol generator (Sanofi-Synthelabo Ltd)	nil
249	Bronchodil 10mg/5ml Oral solution (Viatris Pharmaceuticals Ltd)	nil
250	Bronchodil 20mg Tablet (Viatris Pharmaceuticals Ltd)	nil
251	Bronchodil 500microgram/dose Inhalation powder (Viatris Pharmaceuticals Ltd)	nil
252	Brovon Inhalation vapour (Torbet Laboratories Ltd)	nil
253	Brovon midget Inhalation powder (Torbet Laboratories Ltd)	nil
254	Budelin Novolizer 200micrograms/dose inhalation pdr refill	ICS

For peer review only

For peer review only - http://bmjopen.bmj.com/site/about/guidelines.xhtml

255	Budelin Novolizer 200micrograms/dose inhalation powder	ICS
256	Budelin Novolizer 200micrograms/dose inhalation powder (Meda Pharmaceuticals Ltd)	ICS
257	Budelin Novolizer 200micrograms/dose inhalation powder refill (Meda Pharmaceuticals Ltd)	ICS
258	Budesonide 100microg / Formoterol 6microg/dose dry pdr inh	ICS_LABA
259	Budesonide 100micrograms/actuation inhaler	ICS
260	Budesonide 100micrograms/dose / Formoterol 6micrograms/dose dry powder inhaler	ICS_LABA
261	Budesonide 100micrograms/dose dry powder inhaler	ICS
262	Budesonide 1mg/2ml nebuliser liquid unit dose vials	ICS
263	Budesonide 1mg/2ml nebuliser liquid unit dose vials (A A H Pharmaceuticals Ltd)	ICS
264	Budesonide 200mcg/dose / Formoterol 6mcg/dose dry powder inh	ICS_LABA
265	Budesonide 200microg / Formoterol 6microg/dose inh CFC free	ICS_LABA
266	Budesonide 200microg/dose dry pdr inhalation cart with dev	ICS
267	Budesonide 200micrograms/actuation breath actuated powder inhaler	ICS
268	Budesonide 200micrograms/actuation refill canister	ICS
269	Budesonide 200micrograms/dose / Formoterol 6micrograms/dose dry powder inhaler	ICS_LABA
270	Budesonide 200micrograms/dose / Formoterol 6micrograms/dose inhaler CFC free	ICS_LABA
271	Budesonide 200micrograms/dose dry pdr inhalation cartridge	ICS
272	Budesonide 200micrograms/dose dry powder inhalation cartridge	ICS
273	Budesonide 200micrograms/dose dry powder inhalation cartridge with device	ICS
274	Budesonide 200micrograms/dose dry powder inhaler	ICS
275	Budesonide 200micrograms/dose inhaler	ICS
276	Budesonide 200micrograms/dose inhaler CFC free	ICS
277	Budesonide 400mcg/dose /Formoterol 12mcg/dose dry powder inh	ICS_LABA
278	Budesonide 400micrograms/actuation inhaler	ICS
279	Budesonide 400micrograms/dose / Formoterol 12micrograms/dose dry powder inhaler	ICS_LABA
280	Budesonide 400micrograms/dose dry powder inhaler	ICS
281	Budesonide 400micrograms/dose Turbohaler (Waymade Healthcare Plc)	ICS
282	Budesonide 500micrograms/2ml neb liq unit dose vials	ICS
283	Budesonide 500micrograms/2ml nebuliser liquid unit dose vials	ICS
284	Budesonide 500micrograms/2ml nebuliser liquid unit dose vials (Almus Pharmaceuticals Ltd)	ICS
285	Budesonide 50micrograms/actuation refill canister	ICS
286	Budesonide 50micrograms/dose inhaler	ICS
287	CAM Mixture (Cambridge Healthcare Supplies Ltd)	nil
288	Choledyl 100mg Tablet (Parke-davis Research Laboratories)	nil
289	Choledyl 200mg Tablet (Parke-davis Research Laboratories)	nil
290	Choledyl 62.5mg/5ml Oral solution (Parke-davis Research Laboratories)	nil
291	Choline theophyllinate 100mg tablets	other
292	Choline theophyllinate 200mg tablets	other
293	Choline theophyllinate 62.5mg/5ml oral solution	other
294	Ciclesonide 160micrograms/dose inhaler CFC free	ICS
295	Ciclesonide 80micrograms/dose inhaler CFC free	ICS
296	Clenil Modulite 100micrograms/dose inhaler	ICS
297	Clenil Modulite 100micrograms/dose inhaler (Chiesi Ltd)	ICS
298	Clenil Modulite 100micrograms/dose inhaler (Mawdsley-Brooks & Company Ltd)	ICS
299	Clenil Modulite 200micrograms/dose inhaler	ICS
300	Clenil Modulite 200micrograms/dose inhaler (Chiesi Ltd)	ICS

For peer review only - http://bmjopen.bmj.com/site/about/guidelines.xhtml

301	Clenil Modulite 200micrograms/dose inhaler (Mawdsley-Brooks & Company Ltd)	ICS
302	Clenil Modulite 250micrograms/dose inhaler	ICS
303	Clenil Modulite 250micrograms/dose inhaler (Chiesi Ltd)	ICS
304	Clenil Modulite 250micrograms/dose inhaler (Waymade Healthcare Plc)	ICS
305	Clenil Modulite 50micrograms/dose inhaler	ICS
306	Clenil Modulite 50micrograms/dose inhaler (Chiesi Ltd)	ICS
307	Clenil Modulite 50micrograms/dose inhaler (Mawdsley-Brooks & Company Ltd)	ICS
308	Cobutolin 2mg Tablet (Actavis UK Ltd)	nil
309	Combisal 25micrograms/dose / 125micrograms/dose inhaler	ICS_LABA
310	Combisal 25micrograms/dose / 250micrograms/dose inhaler	ICS_LABA
311	Combisal 25micrograms/dose / 50micrograms/dose inhaler	ICS_LABA
312	Combivent nebuliser liquid 2.5ml UDVs (Boehringer Ingelheim Ltd)	other
313	Combivent nebuliser liquid 2.5ml UDVs (DE Pharmaceuticals)	other
314	Combivent nebuliser liquid 2.5ml UDVs (Lexon (UK) Ltd)	other
315	Dexamethasone 100microgram capsules	ICS
316	Dexamethasone 10mg capsules	ICS
317	Dexamethasone 10mg/5ml oral solution	ICS
318	Dexamethasone 10mg/5ml oral solution sugar free	ICS
319	Dexamethasone 1mg/5ml oral solution	ICS
320	Dexamethasone 20mg/5ml oral solution sugar free	ICS
321	Dexamethasone 2mg/5ml oral solution	ICS
322	Dexamethasone 2mg/5ml oral solution sugar free	ICS
323	Dexamethasone 2mg/5ml oral solution sugar free (A A H Pharmaceuticals Ltd)	ICS
324	Dexamethasone 2mg/5ml oral suspension	ICS
325	Dexamethasone 4mg/5ml oral suspension	ICS
326	Dexamethasone 500micrograms/5ml oral solution	ICS
327	Dexamethasone 5mg/5ml oral solution	ICS
328	Dexamethasone 5mg/5ml oral suspension	ICS
329	Dexsol 2mg/5ml oral solution (Rosemont Pharmaceuticals Ltd)	ICS
330	Duaklir 340micrograms/dose / 12micrograms/dose Genuair	LAMA_LABA
331	Duaklir 340micrograms/dose / 12micrograms/dose Genuair (AstraZeneca UK Ltd)	LAMA_LABA
332	Duo-autohaler Inhalation powder (3M Health Care Ltd)	ICS_LABA
333	DuoResp Spiromax 160microg/dose / 4.5microg/dose dry pdr inh	ICS_LABA
334	DuoResp Spiromax 160micrograms/dose / 4.5micrograms/dose dry powder inhaler (Teva UK Ltd)	ICS_LABA
335	DuoResp Spiromax 320microg/dose / 9microg/dose dry pdr inh	ICS_LABA
336	DuoResp Spiromax 320micrograms/dose / 9micrograms/dose dry powder inhaler (Teva UK Ltd)	ICS_LABA
337	Duovent UDVs nebuliser liquid 4ml (Boehringer Ingelheim Ltd)	other
338	Easyhaler Beclometasone 200micrograms/dose dry pdr inhaler	ICS
339	Easyhaler Beclometasone 200micrograms/dose dry powder inhaler (Orion Pharma (UK) Ltd)	ICS
340	Easyhaler Budesonide 100micrograms/dose dry powder inhaler	ICS
341	Easyhaler Budesonide 100micrograms/dose dry powder inhaler (Orion Pharma (UK) Ltd)	ICS
342	Easyhaler Budesonide 200micrograms/dose dry powder inhaler	ICS
343	Easyhaler Budesonide 200micrograms/dose dry powder inhaler (Orion Pharma (UK) Ltd)	ICS
344	Easyhaler Budesonide 400micrograms/dose dry powder inhaler	ICS
345	Easyhaler Budesonide 400micrograms/dose dry powder inhaler (Orion Pharma (UK) Ltd)	ICS

For peer review only - http://bmjopen.bmj.com/site/about/guidelines.xhtml

346	Easyhaler Salbutamol sulfate 100micrograms/dose dry powder inhaler (Orion Pharma (UK) Ltd)	SABA
347	Easyhaler Salbutamol sulfate 200micrograms/dose dry powder inhaler (Orion Pharma (UK) Ltd)	SABA
348	Eklira 322micrograms/dose Genuair	LAMA
349	Eklira 322micrograms/dose Genuair (AstraZeneca UK Ltd)	LAMA
350	Eklira 322micrograms/dose Genuair (Waymade Healthcare Plc)	LAMA
351	Ephedrine 15mg tablets	other
352	Ephedrine 15mg/5ml oral solution	other
353	Ephedrine 30mg tablets	other
354	Ephedrine 4mg/5ml oral solution sugar free	other
355	Ephedrine 60mg tablets	other
356	Ephedrine HCl 15mg Tablet (C P Pharmaceuticals Ltd)	other
357	Ephedrine HCl 30mg Tablet (C P Pharmaceuticals Ltd)	other
358	Ephedrine HCl with Chlorphenamine 4mg with 1mg/5ml oral solution sugar free	other
359	Ephedrine hydrochloride 15mg tablets	other
360	Ephedrine hydrochloride 15mg tablets (Wockhardt UK Ltd)	other
361	Ephedrine hydrochloride 30mg tablets	other
362	Ephedrine hydrochloride 30mg tablets (Wockhardt UK Ltd)	other
363	Exirel 10mg Capsule (3M Health Care Ltd)	nil
364	Exirel 15mg Capsule (3M Health Care Ltd)	nil
365	Exirel 7.5mg/5ml Oral solution (3M Health Care Ltd)	nil
366	Exirel Inhalation powder (3M Health Care Ltd)	nil
367	Expulin decongestant sugar free Oral solution (Shire Pharmaceuticals Ltd)	nil
368	Fenoterol 1.25mg/4ml / Ipratropium 500micrograms/4ml nebuliser liquid unit dose vials	other
369	Fenoterol 100microgram/actuation inhaler	other
370	Filair 100 inhaler (Meda Pharmaceuticals Ltd)	nil
371	Filair 50 inhaler (Meda Pharmaceuticals Ltd)	nil
372	Filair Forte 250micrograms/dose inhaler (Meda Pharmaceuticals Ltd)	nil
373	Flixotide 0.5mg/2ml Nebules	ICS
374	Flixotide 0.5mg/2ml Nebules (GlaxoSmithKline UK Ltd)	ICS
375	Flixotide 0.5mg/2ml Nebules (Waymade Healthcare Plc)	ICS
376	Flixotide 100microgram Disc (Allen & Hanburys Ltd)	ICS
377	Flixotide 100microgram disks (GlaxoSmithKline UK Ltd)	ICS
378	Flixotide 100microgram disks with Diskhaler (GlaxoSmithKline UK Ltd)	ICS
379	Flixotide 100micrograms/dose Accuhaler	ICS
380	Flixotide 100micrograms/dose Accuhaler (GlaxoSmithKline UK Ltd)	ICS
381	Flixotide 100micrograms/dose Accuhaler (Waymade Healthcare Plc)	ICS
382	Flixotide 125microgram/actuation Inhalation powder (Allen & Hanburys Ltd)	ICS
383	Flixotide 125micrograms/dose Evohaler	ICS
384	Flixotide 125micrograms/dose Evohaler (DE Pharmaceuticals)	ICS
385	Flixotide 125micrograms/dose Evohaler (Dowelhurst Ltd)	ICS
386	Flixotide 125micrograms/dose Evohaler (GlaxoSmithKline UK Ltd)	ICS
387	Flixotide 125micrograms/dose Evohaler (Lexon (UK) Ltd)	ICS
388	Flixotide 250microgram Disc (Allen & Hanburys Ltd)	ICS
389	Flixotide 250microgram disks (GlaxoSmithKline UK Ltd)	ICS
390	Flixotide 250microgram disks with Diskhaler (GlaxoSmithKline UK Ltd)	ICS
391	Flixotide 250microgram/actuation Inhalation powder (Allen & Hanburys Ltd)	ICS

For peer review only - http://bmjopen.bmj.com/site/about/guidelines.xhtml

392	Flixotide 250micrograms/dose Accuhaler	ICS
393	Flixotide 250micrograms/dose Accuhaler (GlaxoSmithKline UK Ltd)	ICS
394	Flixotide 250micrograms/dose Accuhaler (Stephar (U.K.) Ltd)	ICS
395	Flixotide 250micrograms/dose Accuhaler (Waymade Healthcare Plc)	ICS
396	Flixotide 250micrograms/dose Evohaler	ICS
397	Flixotide 250micrograms/dose Evohaler (GlaxoSmithKline UK Ltd)	ICS
398	Flixotide 250micrograms/dose Evohaler (Waymade Healthcare Plc)	ICS
399	Flixotide 25micrograms/dose inhaler (GlaxoSmithKline UK Ltd)	ICS
400	Flixotide 2mg/2ml Nebules	ICS
401	Flixotide 2mg/2ml Nebules (GlaxoSmithKline UK Ltd)	ICS
402	Flixotide 500microgram Disc (Allen & Hanburys Ltd)	ICS
403	Flixotide 500microgram disks (GlaxoSmithKline UK Ltd)	ICS
404	Flixotide 500microgram disks with Diskhaler (GlaxoSmithKline UK Ltd)	ICS
405	Flixotide 500micrograms/dose Accuhaler	ICS
406	Flixotide 500micrograms/dose Accuhaler (GlaxoSmithKline UK Ltd)	ICS
407	Flixotide 500micrograms/dose Accuhaler (Waymade Healthcare Plc)	ICS
408	Flixotide 50microgram Disc (Allen & Hanburys Ltd)	ICS
409	Flixotide 50microgram disks (GlaxoSmithKline UK Ltd)	ICS
410	Flixotide 50microgram disks with Diskhaler (GlaxoSmithKline UK Ltd)	ICS
411	Flixotide 50microgram/actuation Inhalation powder (Allen & Hanburys Ltd)	ICS
412	Flixotide 50micrograms/dose Accuhaler	ICS
413	Flixotide 50micrograms/dose Accuhaler (DE Pharmaceuticals)	ICS
414	Flixotide 50micrograms/dose Accuhaler (GlaxoSmithKline UK Ltd)	ICS
415	Flixotide 50micrograms/dose Accuhaler (Mawdsley-Brooks & Company Ltd)	ICS
416	Flixotide 50micrograms/dose Accuhaler (Sigma Pharmaceuticals Plc)	ICS
417	Flixotide 50micrograms/dose Evohaler	ICS
418	Flixotide 50micrograms/dose Evohaler (GlaxoSmithKline UK Ltd)	ICS
419	Flixotide 50micrograms/dose Evohaler (Lexon (UK) Ltd)	ICS
420	Flixotide accuhaler 100 100microgram/inhalation Inhalation powder (Allen & Hanburys Ltd)	ICS
421	Flixotide accuhaler 250 250microgram/inhalation Inhalation powder (Allen & Hanburys Ltd)	ICS
422	Flixotide accuhaler 50 50microgram/inhalation Inhalation powder (Allen & Hanburys Ltd)	ICS
423	Flixotide accuhaler 500 500microgram/inhalation Inhalation powder (Allen & Hanburys Ltd)	ICS
424	Fluticasone 100microgram Disc	ICS
425	Fluticasone 125mcg/dose / Salmeterol 25mcg/dose inh CFC free	ICS_LABA
426	Fluticasone 125microg / Formoterol 5microg/dose inh CFC free	ICS_LABA
427	Fluticasone 125microg/Formoterol 5microg/dose BA inh CFCfree	ICS_LABA
428	Fluticasone 125microgram/actuation Pressurised inhalation	ICS
429	Fluticasone 125micrograms/dose / Formoterol 5micrograms/dose inhaler CFC free	ICS_LABA
430	Fluticasone 125micrograms/dose / Salmeterol 25micrograms/dose inhaler CFC free	ICS_LABA
431	Fluticasone 125micrograms/dose inhaler CFC free	ICS
432	Fluticasone 250mcg/dose / Salmeterol 25mcg/dose inh CFC free	ICS_LABA
433	Fluticasone 250microg / Formoterol 10microg/dose inh CFCfree	ICS_LABA
434	Fluticasone 250microgram Disc	ICS
435	Fluticasone 250microgram/actuation Pressurised inhalation	ICS
436	Fluticasone 250micrograms/dose / Formoterol 10micrograms/dose inhaler CFC free	ICS_LABA
437	Fluticasone 250micrograms/dose / Salmeterol 25micrograms/dose inhaler CFC free	ICS_LABA

For peer review only - http://bmjopen.bmj.com/site/about/guidelines.xhtml

438	Fluticasone 250micrograms/dose / Salmeterol 25micrograms/dose inhaler CFC free (A A H Pharmaceuticals Ltd)	ICS_LABA
439	Fluticasone 250micrograms/dose Evohaler (Sigma Pharmaceuticals Plc)	ICS_LABA
440	Fluticasone 250micrograms/dose inhaler CFC free	ICS
441	Fluticasone 25micrograms/dose inhaler	ICS
442	Fluticasone 2mg/2ml nebuliser liquid unit dose vials	ICS
443	Fluticasone 500microgram Disc	ICS
444	Fluticasone 500micrograms/2ml neb liq unit dose vials	ICS
445	Fluticasone 500micrograms/2ml nebuliser liquid unit dose vials	ICS
446	Fluticasone 50microg / Formoterol 5microg/dose inh CFC free	ICS_LABA
447	Fluticasone 50microg / Salmeterol 25microg/dose inh CFC free	ICS_LABA
448	Fluticasone 50microg/Formoterol 5microg/dose BA inh CFC free	ICS_LABA
449	Fluticasone 50microgram Disc	ICS
450	Fluticasone 50microgram/actuation Pressurised inhalation	ICS
451	Fluticasone 50micrograms/dose / Formoterol 5micrograms/dose inhaler CFC free	ICS_LABA
452	Fluticasone 50micrograms/dose / Salmeterol 25micrograms/dose inhaler CFC free	ICS_LABA
453	Fluticasone 50micrograms/dose inhaler CFC free	ICS
454	Fluticasone furoate 184microg/Vilanterol 22microg/dose	ICS_LABA
455	Fluticasone furoate 184micrograms/dose / Vilanterol 22micrograms/dose dry powder inhaler	ICS_LABA
456	Fluticasone furoate 92microg/Vilanterol 22microg/dose	ICS_LABA
457	Fluticasone furoate 92micrograms/dose / Vilanterol 22micrograms/dose dry powder inhaler	ICS_LABA
458	Fluticasone propion 500mcg/Salmeterol 50mcg/dose dry pdr inh	ICS_LABA
459	Fluticasone propionate 100microg/Salmeterol 50microg/dose	ICS_LABA
460	Fluticasone propionate 100microgram inhalation powder blisters	ICS
461	Fluticasone propionate 100microgram inhalation powder blisters with device	ICS
462	Fluticasone propionate 100micrograms/dose / Salmeterol 50micrograms/dose dry powder inhaler	ICS_LABA
463	Fluticasone propionate 100micrograms/dose dry powder inhaler	ICS
464	Fluticasone propionate 250microg/Salmeterol 50microg/dose	ICS_LABA
465	Fluticasone propionate 250microgram inhalation powder blisters	ICS
466	Fluticasone propionate 250microgram inhalation powder blisters with device	ICS
467	Fluticasone propionate 250micrograms/dose / Salmeterol 50micrograms/dose dry powder inhaler	ICS_LABA
468	Fluticasone propionate 250micrograms/dose dry powder inhaler	ICS
469	Fluticasone propionate 500microgram inhalation powder blisters	ICS
470	Fluticasone propionate 500microgram inhalation powder blisters with device	ICS
471	Fluticasone propionate 500micrograms/dose / Salmeterol 50micrograms/dose dry powder inhaler	ICS_LABA
472	Fluticasone propionate 500micrograms/dose / Salmeterol 50micrograms/dose dry powder inhaler (A A H Pharmaceuticals Ltd)	ICS_LABA
473	Fluticasone propionate 500micrograms/dose dry powder inhaler	ICS
474	Fluticasone propionate 50microgram inhalation powder blisters	ICS
475	Fluticasone propionate 50microgram inhalation powder blisters with device	ICS
476	Fluticasone propionate 50micrograms/dose dry powder inhaler	ICS
477	Flutiform 125micrograms/dose / 5micrograms/dose inhaler	ICS_LABA
478	Flutiform 125micrograms/dose / 5micrograms/dose inhaler (Napp Pharmaceuticals Ltd)	ICS_LABA
479	Flutiform 125micrograms/dose / 5micrograms/dose inhaler (Waymade Healthcare Plc)	ICS_LABA
480	Flutiform 250micrograms/dose / 10micrograms/dose inhaler	ICS_LABA
481	Flutiform 250micrograms/dose / 10micrograms/dose inhaler (Napp Pharmaceuticals Ltd)	ICS_LABA
482	Flutiform 250micrograms/dose / 10micrograms/dose inhaler (Waymade Healthcare Plc)	ICS_LABA

For peer review only - http://bmjopen.bmj.com/site/about/guidelines.xhtml

483	Flutiform 50micrograms/dose / 5micrograms/dose inhaler	ICS_LABA
484	Flutiform 50micrograms/dose / 5micrograms/dose inhaler (Napp Pharmaceuticals Ltd)	ICS_LABA
485	Flutiform 50micrograms/dose / 5micrograms/dose inhaler (Waymade Healthcare Plc)	ICS_LABA
486	Flutiform K-haler 125microg/dose / 5microg/dose BA inhaler	ICS_LABA
487	Flutiform K-haler 50microg/dose / 5microg/dose BA inh	ICS_LABA
488	Fobumix Easyhaler 160microg / 4.5microg/dose dry pdr inh	ICS_LABA
489	Fobumix Easyhaler 320microg/dose / 9microg/dose dry pdr inh	ICS_LABA
490	Fobumix Easyhaler 80microg/dose / 4.5microg/dose dry pdr inh	ICS_LABA
491	Foradil 12microgram inhalation powder capsules with device	LABA
492	Foradil 12microgram inhalation powder capsules with device (Novartis Pharmaceuticals UK Ltd)	LABA
493	Foradil 12microgram inhalation powder capsules with device (Sigma Pharmaceuticals Plc)	LABA
494	Formoterol 12microgram inhalation pdr caps with device	LABA
495	Formoterol 12microgram inhalation powder capsules with device	LABA
496	Formoterol 12micrograms/dose dry powder inhaler	LABA
497	Formoterol 12micrograms/dose inhaler CFC free	LABA
498	Formoterol 6micrograms/dose dry powder inhaler	LABA
499	Formoterol Easyhaler 12micrograms/dose dry powder inhaler	LABA
500	Formoterol Easyhaler 12micrograms/dose dry powder inhaler (Orion Pharma (UK) Ltd)	LABA
501	Fostair 100micrograms/dose / 6micrograms/dose inhaler	ICS_LABA
502	Fostair 100micrograms/dose / 6micrograms/dose inhaler (Chiesi Ltd)	ICS_LABA
503	Fostair 200micrograms/dose / 6micrograms/dose inhaler	ICS_LABA
504	Fostair 200micrograms/dose / 6micrograms/dose inhaler (Chiesi Ltd)	ICS_LABA
505	Fostair NEXThaler 100microg/dose / 6microg/dose dry pdr inh	ICS_LABA
506	Fostair NEXThaler 100micrograms/dose / 6micrograms/dose dry powder inhaler (Chiesi Ltd)	ICS_LABA
507	Fostair NEXThaler 200microg/dose / 6microg/dose dry pdr inh	ICS_LABA
508	Fostair NEXThaler 200micrograms/dose / 6micrograms/dose dry powder inhaler (Chiesi Ltd)	ICS_LABA
509	Fusacomb Easyhaler 50microg / 250microg/dose dry pdr inh	ICS_LABA
510	Fusacomb Easyhaler 50microg / 500microg/dose dry pdr inh	ICS_LABA
511	Glycopyrronium bromide 55microg inhalation pdr caps with dev	LAMA
512	Glycopyrronium bromide 55microgram inhalation powder capsules with device	LAMA
513	Glycopyrronium bromide 55microgram inhalation powder capsules with device (J M McGill Ltd)	LAMA
514	Incruse Ellipta 55micrograms/dose dry powder inhaler	LAMA
515	Incruse Ellipta 55micrograms/dose dry powder inhaler (GlaxoSmithKline UK Ltd)	LAMA
516	Indacaterol 150microgram inhalation pdr caps with device	LABA
517	Indacaterol 150microgram inhalation powder capsules with device	LABA
518	Indacaterol 300microgram inhalation pdr caps with device	LABA
519	Indacaterol 300microgram inhalation powder capsules with device	LABA
520	Indacaterol 85microg/Glycopyrronium brom 54microg/dose	LAMA_LABA
521	Indacaterol 85micrograms/dose / Glycopyrronium bromide 54micrograms/dose inhalation powder capsules with device	LAMA_LABA
522	Intal 20mg/2ml nebuliser solution unit dose vials (Aventis Pharma)	nil
523	Intal 5mg/dose inhaler CFC free (Sanofi)	nil
524	Intal autohaler 5 5mg/inhalation Pressurised inhalation (Aventis Pharma)	nil
525	Intal compound Capsule (Rhone-Poulenc Rorer Ltd)	nil
526	Ipramol nebuliser solution 2.5ml Steri-Neb unit dose vials (Teva UK Ltd)	other
527	Ipratropium 250micrograms/1ml nebuliser liquid Steri-Neb unit dose vials (Teva UK Ltd)	ipratropium

For peer review only - http://bmjopen.bmj.com/site/about/guidelines.xhtml

528	Ipratropium 250micrograms/1ml nebuliser liquid unit dose Steripoule vials (Galen Ltd)	ipratropium
529	Ipratropium 500micrograms/2ml nebuliser liquid Steri-Neb unit dose vials (Teva UK Ltd)	ipratropium
530	Ipratropium 500micrograms/2ml nebuliser liquid unit dose Steripoule vials (Galen Ltd)	ipratropium
531	Ipratropium bromide 0.25mg/ml	ipratropium
532	Ipratropium bromide 20micrograms/dose breath actuated inhaler	ipratropium
533	Ipratropium bromide 20micrograms/dose inhaler	ipratropium
534	Ipratropium bromide 20micrograms/dose inhaler CFC free	ipratropium
535	Ipratropium bromide 250microgram/ml	ipratropium
536	Ipratropium bromide 250microgram/ml Inhalation vapour (Galen Ltd)	ipratropium
537	Ipratropium bromide 250microgram/ml Nebuliser liquid (Approved Prescription Services Ltd)	ipratropium
538	Ipratropium bromide 250microgram/ml Nebuliser liquid (Galen Ltd)	ipratropium
539	Ipratropium bromide 250microgram/ml Nebuliser liquid (Hillcross Pharmaceuticals Ltd)	ipratropium
540	Ipratropium bromide 250micrograms/1ml nebuliser liquid unit dose vials	ipratropium
541	Ipratropium bromide 250micrograms/ml	ipratropium
542	Ipratropium bromide 40microgram inhalation powder capsules	ipratropium
543	Ipratropium bromide 40microgram inhalation powder capsules with device	ipratropium
544	Ipratropium bromide 40micrograms/dose inhaler	ipratropium
545	Ipratropium bromide 500micrograms/2ml nebuliser liquid unit dose vials	ipratropium
546	Ipratropium bromide 500micrograms/2ml nebuliser liquid unit dose vials (A A H Pharmaceuticals Ltd)	ipratropium
547	Ipratropium bromide with fenoterol hydrobromide 0micrograms + 100micrograms/actuation	other
548	Ipratropium bromide with fenoterol hydrobromide 40micrograms + 100micrograms/actuation	other
549	Ipratropium bromide with fenoterol hydrobromide 500micrograms + 1.25mg/4ml	other
550	Ipratropium bromide with salbutamol 20mcg + 100mcg	other
551	Ipratropium bromide with salbutamol 500micrograms + 2.5mg/2.5ml	other
552	Iso-autohaler Inhalation powder (3M Health Care Ltd)	other
553	Isoprenaline hc 500micrograms + 50micrograms/metered Pressurised inhalation	other
554	Isoprenaline sulphate 400micrograms/actuation Pressurised inhalation	other
555	Isoprenaline sulphate 80micrograms/actuation Pressurised inhalation	other
556	Isoprenaline sulphate with sodium cromoglicate inhalation Capsule	other
557	Kelhale 100micrograms/dose inhaler	ICS
558	Kelhale 50micrograms/dose inhaler	ICS
559	Lasma 300mg Tablet (Pharmax Ltd)	other
560	Martapan 2mg/5ml oral solution (Martindale Pharmaceuticals Ltd)	Nil
561	Maxivent 100microgram/inhalation Inhalation powder (Ashbourne Pharmaceuticals Ltd)	SABA
562	Maxivent 2.5mg/2.5ml nebuliser liquid unit dose Steripoule vials (Ashbourne Pharmaceuticals Ltd)	SABA
563	Maxivent 5mg/2.5ml nebuliser liquid unit dose Steripoule vials (Ashbourne Pharmaceuticals Ltd)	SABA
564	Medihaler -duo Inhalation powder (3M Health Care Ltd)	SABA
565	Medihaler -iso Aerosol generator (3M Health Care Ltd)	SABA
566	Medihaler -iso forte Inhalation powder (3M Health Care Ltd)	SABA
567	Mometasone 200micrograms/dose dry powder inhaler	ICS
568	Mometasone 400micrograms/dose dry powder inhaler	ICS
569	Monovent 1.5mg/5ml Oral solution (Lagap)	nil
570	Montelukast 10mg tablets	montelukast
571	Montelukast 10mg tablets (A A H Pharmaceuticals Ltd)	montelukast
572	Montelukast 10mg tablets (Accord Healthcare Ltd)	montelukast

For peer review only - http://bmjopen.bmj.com/site/about/guidelines.xhtml

573	Montelukast 10mg tablets (Actavis UK Ltd)	montelukast
574	Montelukast 10mg tablets (Alliance Healthcare (Distribution) Ltd)	montelukast
575	Montelukast 10mg tablets (Dr Reddy's Laboratories (UK) Ltd)	montelukast
576	Montelukast 10mg tablets (Milpharm Ltd)	montelukast
577	Montelukast 10mg tablets (Ranbaxy (UK) Ltd)	montelukast
578	Montelukast 10mg tablets (Teva UK Ltd)	montelukast
579	Montelukast 4mg chewable tablets sugar free	montelukast
580	Montelukast 4mg chewable tablets sugar free (Accord Healthcare Ltd)	montelukast
581	Montelukast 4mg chewable tablets sugar free (Actavis UK Ltd)	montelukast
582	Montelukast 4mg chewable tablets sugar free (Alliance Healthcare (Distribution) Ltd)	montelukast
583	Montelukast 4mg granules sachets sugar free	montelukast
584	Montelukast 4mg granules sachets sugar free (A A H Pharmaceuticals Ltd)	montelukast
585	Montelukast 4mg granules sachets sugar free (Teva UK Ltd)	montelukast
586	Montelukast 5mg chewable tablets sugar free	montelukast
587	Montelukast 5mg chewable tablets sugar free (Accord Healthcare Ltd)	montelukast
588	Montelukast 5mg chewable tablets sugar free (Actavis UK Ltd)	montelukast
589	Montelukast 5mg chewable tablets sugar free (Teva UK Ltd)	montelukast
590	Nedocromil 2mg/dose inhaler CFC free	nil
591	Nedocromil sodium 1% nasal spray	nil
592	Nedocromil sodium 2mg/inhalation inhaler	nil
593	Neovent 25micrograms/dose inhaler CFC free (Kent Pharm)	LABA
594	Neovent 25micrograms/dose inhaler CFC free (Kent Pharmaceuticals Ltd)	LABA
595	Nethaprin dospan Tablet (Marion Merrell Dow Ltd)	nil
596	Noradran 7.5mg+15mg/5ml Oral solution (Norma Chemicals Ltd)	nil
597	Novolizer budesonide 200microgram/actuation Pressurised inhalation (Meda Pharmaceuticals Ltd)	nil
598	Nuelin 60mg/5ml liquid (3M Health Care Ltd)	nil
599	Nuelin SA 175mg tablets (Meda Pharmaceuticals Ltd)	nil
600	Nuelin SA 250 tablets (Meda Pharmaceuticals Ltd)	nil
601	Numotac 10mg Tablet (3M Health Care Ltd)	nil
602	Olodaterol 2.5microg/dose soln for inhalation CFCfree	LABA
603	Olodaterol 2.5micrograms/dose inhalation soln cart CFC free	LABA
604	Olodaterol 2.5micrograms/dose solution for inhalation cartridge with device CFC free	LABA
605	Onbrez Breezhaler 150microgram inhalation pdr caps with dev	LABA
606	Onbrez Breezhaler 150microgram inhalation powder capsules with device (Novartis Pharmaceuticals UK Ltd)	LABA
607	Onbrez Breezhaler 300microgram inhalation pdr caps with dev	LABA
608	Onbrez Breezhaler 300microgram inhalation powder capsules with device (Novartis Pharmaceuticals UK Ltd)	LABA
609	Orciprenaline 0.5mg/ml Injection	other
610	Orciprenaline 750micrograms/inhalation Aerosol refill	other
611	Orciprenaline 750micrograms/inhalation inhaler	other
612	Orciprenaline with bromhexine hcl mixture	other
613	Oxis 12 Turbohaler	LABA
614	Oxis 12 Turbohaler (AstraZeneca UK Ltd)	LABA
615	Oxis 12 Turbohaler (DE Pharmaceuticals)	LABA
616	Oxis 12 Turbohaler (Waymade Healthcare Plc)	LABA
617	Oxis 6 Turbohaler	LABA

For peer review only - http://bmjopen.bmj.com/site/about/guidelines.xhtml

618	Oxis 6 Turbohaler (AstraZeneca UK Ltd)	LABA
619	Oxis 6 Turbohaler (Lexon (UK) Ltd)	LABA
620	Pecram 225mg Modified-release tablet (Novartis Consumer Health UK Ltd)	nil
621	Phyllocontin Continus 225mg tablets (Napp Pharmaceuticals Ltd)	other
622	Phyllocontin continus 350mg Tablet (Napp Pharmaceuticals Ltd)	other
623	Phyllocontin Forte Continus 350mg tablets (Napp Pharmaceuticals Ltd)	other
624	Pirbuterol 10mg capsule	SABA
625	Pirbuterol 15mg capsule	SABA
626	Pirbuterol 7.5mg/5ml oral solution	SABA
627	Pirbuterol acetate inhaler	SABA
628	Prednisolone 1mg/ml oral solution (Logixx Pharma Solutions Ltd)	other
629	Prednisolone 5mg/5ml oral solution	other
630	Prednisolone 5mg/5ml oral solution unit dose	other
631	Prednisolone 5mg/5ml oral solution unit dose (A A H Pharmaceuticals Ltd)	other
632	Prednisolone 5mg/5ml oral solution unit dose (Logixx Pharma Solutions Ltd)	other
633	Prednisolone Dompe 5mg/5ml oral solution unit dose (Logixx Pharma Solutions Ltd)	other
634	Pro-vent 300mg Capsule (Wellcome Medical Division)	other
635	Pulmadil auto Inhalation powder (3M Health Care Ltd)	nil
636	Pulmadil Inhalation powder (3M Health Care Ltd)	nil
637	Pulmicort 0.5mg Respules	ICS
638	Pulmicort 0.5mg Respules (AstraZeneca UK Ltd)	ICS
639	Pulmicort 0.5mg Respules (Necessity Supplies Ltd)	ICS
640	Pulmicort 0.5mg Respules (Waymade Healthcare Plc)	ICS
641	Pulmicort 100 Turbohaler	ICS
642	Pulmicort 100 Turbohaler (AstraZeneca UK Ltd)	ICS
643	Pulmicort 100 Turbohaler (Waymade Healthcare Plc)	ICS
644	Pulmicort 1mg Respules	ICS
645	Pulmicort 1mg Respules (AstraZeneca UK Ltd)	ICS
646	Pulmicort 1mg Respules (Sigma Pharmaceuticals Plc)	ICS
647	Pulmicort 200 Turbohaler	ICS
648	Pulmicort 200 Turbohaler (AstraZeneca UK Ltd)	ICS
649	Pulmicort 200 Turbohaler (Dowelhurst Ltd)	ICS
650	Pulmicort 200 Turbohaler (Waymade Healthcare Plc)	ICS
651	Pulmicort 200microgram Inhaler (AstraZeneca UK Ltd)	ICS
652	Pulmicort 200microgram Refill canister (AstraZeneca UK Ltd)	ICS
653	Pulmicort 200micrograms/dose inhaler (AstraZeneca UK Ltd)	ICS
654	Pulmicort 200micrograms/dose inhaler CFC free	ICS
655	Pulmicort 200micrograms/dose inhaler CFC free (AstraZeneca UK Ltd)	ICS
656	Pulmicort 400 Turbohaler	ICS
657	Pulmicort 400 Turbohaler (AstraZeneca UK Ltd)	ICS
658	Pulmicort 400 Turbohaler (Waymade Healthcare Plc)	ICS
659	Pulmicort LS 50microgram Refill canister (AstraZeneca UK Ltd)	ICS
660	Pulmicort LS 50micrograms/dose inhaler (AstraZeneca UK Ltd)	ICS
661	Pulvinal Beclometasone Dipropionate 100micrograms/dose dry powder inhaler (Chiesi Ltd)	ICS
662	Pulvinal Beclometasone Dipropionate 200micrograms/dose dry powder inhaler (Chiesi Ltd)	ICS
663	Pulvinal Beclometasone Dipropionate 400micrograms/dose dry powder inhaler (Chiesi Ltd)	ICS

For peer review only

For peer review only - http://bmjopen.bmj.com/site/about/guidelines.xhtml

664	Pulvinal Salbutamol 200micrograms/dose dry powder inhaler (Chiesi Ltd)	SABA
665	Qvar 100 Autohaler	ICS
666	Qvar 100 Autohaler (DE Pharmaceuticals)	ICS
667	Qvar 100 Autohaler (Lexon (UK) Ltd)	ICS
668	Qvar 100 Autohaler (Sigma Pharmaceuticals Plc)	ICS
669	Qvar 100 Autohaler (Stephar (U.K.) Ltd)	ICS
670	Qvar 100 Autohaler (Teva UK Ltd)	ICS
671	Qvar 100 inhaler	ICS
672	Qvar 100 inhaler (DE Pharmaceuticals)	ICS
673	Qvar 100 inhaler (Sigma Pharmaceuticals Plc)	ICS
674	Qvar 100 inhaler (Teva UK Ltd)	ICS
675	Qvar 100 inhaler (Waymade Healthcare Plc)	ICS
676	Qvar 100micrograms/dose Easi-Breathe inhaler	ICS
677	Qvar 100micrograms/dose Easi-Breathe inhaler (DE Pharmaceuticals)	ICS
678	Qvar 100micrograms/dose Easi-Breathe inhaler (Sigma Pharmaceuticals Plc)	ICS
679	Qvar 100micrograms/dose Easi-Breathe inhaler (Teva UK Ltd)	ICS
680	Qvar 50 Autohaler	ICS
681	Qvar 50 Autohaler (Teva UK Ltd)	ICS
682	Qvar 50 inhaler	ICS
683	Qvar 50 inhaler (DE Pharmaceuticals)	ICS
684	Qvar 50 inhaler (Mawdsley-Brooks & Company Ltd)	ICS
685	Qvar 50 inhaler (Teva UK Ltd)	ICS
686	Qvar 50micrograms/dose Easi-Breathe inhaler	ICS
687	Qvar 50micrograms/dose Easi-Breathe inhaler (Sigma Pharmaceuticals Plc)	ICS
688	Qvar 50micrograms/dose Easi-Breathe inhaler (Teva UK Ltd)	ICS
689	Relvar Ellipta 184microg/dose / 22microg/dose dry pdr inh	ICS_LABA
690	Relvar Ellipta 184micrograms/dose / 22micrograms/dose dry powder inhaler (GlaxoSmithKline UK Ltd)	ICS_LABA
691	Relvar Ellipta 92microg/dose / 22microg/dose dry pdr inh	ICS_LABA
692	Relvar Ellipta 92micrograms/dose / 22micrograms/dose dry powder inhaler (GlaxoSmithKline UK Ltd)	ICS_LABA
693	Reproterol 10mg/5ml oral solution	SABA
694	Reproterol 10mg/ml respirator solution	SABA
695	Reproterol 500micrograms/dose inhaler	SABA
696	Respacal 2mg Tablet (UCB Pharma Ltd)	LABA
697	Respontin 250micrograms/1ml Nebules (GlaxoSmithKline UK Ltd)	ipratropium
698	Respontin 500micrograms/2ml Nebules (GlaxoSmithKline UK Ltd)	ipratropium
699	Respontin nebules 250microgram/ml Nebuliser liquid (Glaxo Wellcome UK Ltd)	ipratropium
700	Rimiterol inhaler	SABA
701	Rybarvin Inhalation vapour (Rybar Laboratories Ltd)	other
702	Salamol 100microgram/actuation Inhalation powder (IVAX Pharmaceuticals UK Ltd)	SABA
703	Salamol 100microgram/inhalation Inhalation powder (Kent Pharmaceuticals Ltd)	SABA
704	Salamol 100microgram/inhalation Inhalation powder (Sandoz Ltd)	SABA
705	Salamol 100micrograms/dose Easi-Breathe inhaler (DE Pharmaceuticals)	SABA
706	Salamol 100micrograms/dose Easi-Breathe inhaler (Teva UK Ltd)	SABA
707	Salamol 100micrograms/dose inhaler CFC free (Arrow Generics Ltd)	SABA
708	Salamol 100micrograms/dose inhaler CFC free (Teva UK Ltd)	SABA
709	Salamol 2.5mg/2.5ml nebuliser liquid Steri-Neb unit dose vials (Teva UK Ltd)	SABA

For peer review only - http://bmjopen.bmj.com/site/about/guidelines.xhtml

710	Salamol 5mg/2.5ml nebuliser liquid Steri-Neb unit dose vials (Teva UK Ltd)	SABA
711	Salamol easi-breathe 100microgram/actuation Pressurised inhalation (IVAX Pharmaceuticals UK Ltd)	SABA
712	Salamol steri-neb 2.5mg/2.5ml Nebuliser liquid (Numark Management Ltd)	SABA
713	Salamol steri-neb 5mg/2.5ml Nebuliser liquid (Numark Management Ltd)	SABA
714	Salapin 2mg/5ml syrup (Pinewood Healthcare)	nil
715	Salbulin 100micrograms/dose inhaler (3M Health Care Ltd)	nil
716	Salbulin 2mg Tablet (3M Health Care Ltd)	nil
717	Salbulin 2mg/5ml Oral solution (3M Health Care Ltd)	nil
718	Salbulin 4mg Tablet (3M Health Care Ltd)	nil
719	SALBULIN CFC FREE	nil
720	Salbulin Inhalation powder (3M Health Care Ltd)	nil
721	Salbulin Novolizer 100micrograms/dose inhalation powder (Meda Pharmaceuticals Ltd)	nil
722	Salbulin Novolizer 100micrograms/dose inhalation powder refill (Meda Pharmaceuticals Ltd)	nil
723	SALBUTAMOL	SABA
724	SALBUTAMOL .25 MG INJ	SABA
725	Salbutamol 0.05mg/ml injection	SABA
726	Salbutamol 100microgram/inhalation Inhalation powder (Berk Pharmaceuticals Ltd)	SABA
727	Salbutamol 100microgram/inhalation Inhalation powder (C P Pharmaceuticals Ltd)	SABA
728	Salbutamol 100microgram/inhalation Inhalation powder (Celltech Pharma Europe Ltd)	SABA
729	Salbutamol 100microgram/inhalation Inhalation powder (IVAX Pharmaceuticals UK Ltd)	SABA
730	Salbutamol 100microgram/inhalation Inhalation powder (Kent Pharmaceuticals Ltd)	SABA
731	Salbutamol 100microgram/inhalation Inhalation powder (Neo Laboratories Ltd)	SABA
732	Salbutamol 100microgram/inhalation Spacehaler (Celltech Pharma Europe Ltd)	SABA
733	Salbutamol 100micrograms/actuation breath actuated inhaler	SABA
734	Salbutamol 100micrograms/dose / Beclometasone 50micrograms/dose inhaler	ICS_SABA
735	Salbutamol 100micrograms/dose / Ipratropium 20micrograms/dose inhaler	other
736	Salbutamol 100micrograms/dose breath actuated inhaler	SABA
737	Salbutamol 100micrograms/dose breath actuated inhaler CFC free	SABA
738	Salbutamol 100micrograms/dose dry powder inhalation cartridge	SABA
739	Salbutamol 100micrograms/dose dry powder inhalation cartridge with device	SABA
740	Salbutamol 100micrograms/dose dry powder inhaler	SABA
741	Salbutamol 100micrograms/dose inhaler	SABA
742	Salbutamol 100micrograms/dose inhaler (A A H Pharmaceuticals Ltd)	SABA
743	Salbutamol 100micrograms/dose inhaler (Kent Pharmaceuticals Ltd)	SABA
744	Salbutamol 100micrograms/dose inhaler (Mylan)	SABA
745	Salbutamol 100micrograms/dose inhaler CFC free	SABA
746	Salbutamol 100micrograms/dose inhaler CFC free (A A H Pharmaceuticals Ltd)	SABA
747	Salbutamol 100micrograms/dose inhaler CFC free (Actavis UK Ltd)	SABA
748	Salbutamol 100micrograms/dose inhaler CFC free (Alliance Healthcare (Distribution) Ltd)	SABA
749	Salbutamol 100micrograms/dose inhaler CFC free (AM Distributions (Yorkshire) Ltd)	SABA
750	Salbutamol 100micrograms/dose inhaler CFC free (DE Pharmaceuticals)	SABA
751	Salbutamol 100micrograms/dose inhaler CFC free (Mawdsley-Brooks & Company Ltd)	SABA
752	Salbutamol 100micrograms/dose inhaler CFC free (Mylan)	SABA
753	Salbutamol 100micrograms/dose inhaler CFC free (Phoenix Healthcare Distribution Ltd)	SABA
754	Salbutamol 100micrograms/dose inhaler CFC free (Sandoz Ltd)	SABA
755	Salbutamol 100micrograms/dose inhaler CFC free (Teva UK Ltd)	SABA

For peer review only - http://bmjopen.bmj.com/site/about/guidelines.xhtml

756	Salbutamol 100micrograms/dose inhaler CFC free (Waymade Healthcare Plc)	SABA
757	Salbutamol 100micrograms/inhalation vortex inhaler	SABA
758	SALBUTAMOL 2 MG/5ML SYR	SABA
759	Salbutamol 2.5mg with ipratropium bromide 500micrograms/2.5ml unit dose nebuilser solution	other
760	Salbutamol 2.5mg/2.5ml / Ipratropium bromide 500micrograms/2.5ml nebuliser liquid ampoules	other
761	Salbutamol 2.5mg/2.5ml / Ipratropium bromide 500micrograms/2.5ml nebuliser liquid unit dose vials	other
762	Salbutamol 2.5mg/2.5ml Nebuliser liquid (Galen Ltd)	SABA
763	Salbutamol 2.5mg/2.5ml nebuliser liquid unit dose Steripoule vials (Galen Ltd)	SABA
764	Salbutamol 2.5mg/2.5ml nebuliser liquid unit dose vials	SABA
765	Salbutamol 2.5mg/2.5ml nebuliser liquid unit dose vials (A A H Pharmaceuticals Ltd)	SABA
766	Salbutamol 2.5mg/2.5ml nebuliser liquid unit dose vials (Alliance Healthcare (Distribution) Ltd)	SABA
767	Salbutamol 200 Cyclocaps (Teva UK Ltd)	SABA
768	Salbutamol 200microgram / Beclometasone 100microgram inhalation powder capsules	SABA
769	Salbutamol 200microgram inhalation powder blisters	SABA
770	Salbutamol 200microgram inhalation powder blisters with device	SABA
771	Salbutamol 200microgram inhalation powder capsules	SABA
772	Salbutamol 200micrograms disc	SABA
773	Salbutamol 200micrograms inahalation capsules	SABA
774	Salbutamol 200micrograms/dose dry powder inhaler	SABA
775	Salbutamol 2mg Tablet (C P Pharmaceuticals Ltd)	SABA
776	Salbutamol 2mg tablets	SABA
777	Salbutamol 2mg tablets (Actavis UK Ltd)	SABA
778	Salbutamol 2mg tablets (Approved Prescription Services Ltd)	SABA
779	Salbutamol 2mg/5ml Oral solution (Lagap)	SABA
780	Salbutamol 2mg/5ml oral solution sugar free	SABA
781	Salbutamol 2mg/5ml oral solution sugar free (A A H Pharmaceuticals Ltd)	SABA
782	Salbutamol 2mg/5ml oral solution sugar free (Pinewood Healthcare)	SABA
783	Salbutamol 2mg/5ml oral solution sugar free (Sandoz Ltd)	SABA
784	Salbutamol 400 Cyclocaps (Teva UK Ltd)	SABA
785	SALBUTAMOL 400MCG/BECLOMETH.100MCG R/CAP INH	ICS_SABA
786	Salbutamol 400microgram / Beclometasone 200microgram inhalation powder capsules	ICS_SABA
787	Salbutamol 400microgram inhalation powder blisters	SABA
788	Salbutamol 400microgram inhalation powder blisters with device	SABA
789	Salbutamol 400microgram inhalation powder capsules	SABA
790	Salbutamol 400micrograms disc	SABA
791	Salbutamol 400micrograms inahalation capsules	SABA
792	Salbutamol 4mg modified-release capsules	SABA
793	Salbutamol 4mg modified-release tablets	SABA
794	Salbutamol 4mg tablets	SABA
795	Salbutamol 4mg tablets (A A H Pharmaceuticals Ltd)	SABA
796	Salbutamol 4mg tablets (Actavis UK Ltd)	SABA
797	Salbutamol 500micrograms/1ml solution for injection ampoules	SABA
798	Salbutamol 5mg/2.5ml Nebuliser liquid (Galen Ltd)	SABA
799	Salbutamol 5mg/2.5ml Nebuliser liquid (Generics (UK) Ltd)	SABA
800	Salbutamol 5mg/2.5ml nebuliser liquid unit dose Steripoule vials (Galen Ltd)	SABA

For peer review only

801	Salbutamol 5mg/2.5ml nebuliser liquid unit dose vials	SABA
802	Salbutamol 5mg/2.5ml nebuliser liquid unit dose vials (Actavis UK Ltd)	SABA
803	Salbutamol 5mg/2.5ml nebuliser liquid unit dose vials (Alliance Healthcare (Distribution) Ltd)	SABA
804	Salbutamol 5mg/50ml solution for infusion vials	SABA
805	Salbutamol 5mg/5ml solution for infusion ampoules	SABA
806	Salbutamol 5mg/ml nebuliser liquid	SABA
807	Salbutamol 8mg modified-release capsules	SABA
808	Salbutamol 8mg modified-release tablets	SABA
809	Salbutamol 8mg tablet	SABA
810	Salbutamol 95micrograms/dose dry powder inhaler	SABA
811	SALBUTAMOL CFC/FREE B/A	SABA
812	Salbutamol cyclocaps 200microgram Inhalation powder (DuPont Pharmaceuticals Ltd)	SABA
813	Salbutamol cyclocaps 400microgram Inhalation powder (DuPont Pharmaceuticals Ltd)	SABA
814	Salbutamol cyclohaler	SABA
815	SALBUTAMOL INHALER	SABA
816	SALBUTAMOL RESPIRATOR SOLN	SABA
817	Salbutamol rondo 100micrograms/actuation inhaler and spacer	SABA
818	SALBUTAMOL ROTAHALER COMPLETE UNIT	SABA
819	SALBUTAMOL U.DOSE NEBULISING 2.5MG/2.5ML	SABA
820	Salbuvent 0.5mg/ml Injection (Pharmacia Ltd)	SABA
821	Salbuvent 100microgram/actuation Inhalation powder (Pharmacia Ltd)	SABA
822	Salbuvent 2mg Tablet (Pharmacia Ltd)	SABA
823	Salbuvent 2mg/5ml Oral solution (Pharmacia Ltd)	SABA
824	Salbuvent 4mg Tablet (Pharmacia Ltd)	SABA
825	Salbuvent 5mg/ml Respirator solution (Pharmacia Ltd)	SABA
826	SALBUVENT inh INH	SABA
827	SALBUVENT RONDO	SABA
828	Salipraneb 0.5mg/2.5mg nebuliser solution 2.5ml ampoules (Actavis UK Ltd)	other
829	Salipraneb 0.5mg/2.5mg nebuliser solution 2.5ml ampoules (Arrow Generics Ltd)	other
830	Salmeterol 25micrograms with fluticasone 125micrograms CFC free inhaler	ICS_LABA
831	Salmeterol 25micrograms with fluticasone 250micrograms CFC free inhaler	ICS_LABA
832	Salmeterol 25micrograms with fluticasone 50micrograms CFC free inhaler	ICS_LABA
833	Salmeterol 25micrograms/dose inhaler	LABA
834	Salmeterol 25micrograms/dose inhaler CFC free	LABA
835	Salmeterol 25micrograms/dose inhaler CFC free (A A H Pharmaceuticals Ltd)	LABA
836	Salmeterol 50microgram Diskhaler (Dowelhurst Ltd)	LABA
837	Salmeterol 50microgram inhalation pdr blist with device	LABA
838	Salmeterol 50microgram inhalation powder blisters	LABA
839	Salmeterol 50microgram inhalation powder blisters with device	LABA
840	Salmeterol 50micrograms disc	LABA
841	Salmeterol 50micrograms with fluticasone 100micrograms dry powder inhaler	ICS_LABA
842	Salmeterol 50micrograms with fluticasone 250micrograms CFC free inhaler	ICS_LABA
843	Salmeterol 50micrograms with fluticasone 500micrograms CFC free inhaler	ICS_LABA
844	Salmeterol 50micrograms/dose dry powder inhaler	LABA
845	Seebri Breezhaler 44microgram inhalation pdr caps with dev	LAMA
846	Seebri Breezhaler 44microgram inhalation powder capsules with device (Novartis Pharmaceuticals UK Ltd)	LAMA

For peer review only - http://bmjopen.bmj.com/site/about/guidelines.xhtml

847	Sereflo 25micrograms/dose / 125micrograms/dose inhaler	ICS_LABA
848	Sereflo 25micrograms/dose / 125micrograms/dose inhaler (Kent Pharmaceuticals Ltd)	ICS_LABA
849	Sereflo 25micrograms/dose / 250micrograms/dose inhaler	ICS_LABA
850	Sereflo 25micrograms/dose / 250micrograms/dose inhaler (Kent Pharmaceuticals Ltd)	ICS_LABA
851	Seretide 100 Accuhaler	ICS_LABA
852	Seretide 100 Accuhaler (DE Pharmaceuticals)	ICS_LABA
853	Seretide 100 Accuhaler (GlaxoSmithKline UK Ltd)	ICS_LABA
854	Seretide 100 Accuhaler (Waymade Healthcare Plc)	ICS_LABA
855	Seretide 125 Evohaler	ICS_LABA
856	Seretide 125 Evohaler (DE Pharmaceuticals)	ICS_LABA
857	Seretide 125 Evohaler (GlaxoSmithKline UK Ltd)	ICS_LABA
858	Seretide 125 Evohaler (Lexon (UK) Ltd)	ICS_LABA
859	Seretide 125 Evohaler (Mawdsley-Brooks & Company Ltd)	ICS_LABA
860	Seretide 125 Evohaler (Waymade Healthcare Plc)	ICS_LABA
861	Seretide 250 Accuhaler	ICS_LABA
862	Seretide 250 Accuhaler (DE Pharmaceuticals)	ICS_LABA
863	Seretide 250 Accuhaler (GlaxoSmithKline UK Ltd)	ICS_LABA
864	Seretide 250 Accuhaler (Lexon (UK) Ltd)	ICS_LABA
865	Seretide 250 Accuhaler (Sigma Pharmaceuticals Plc)	ICS_LABA
866	Seretide 250 Accuhaler (Waymade Healthcare Plc)	ICS_LABA
867	Seretide 250 Evohaler	ICS_LABA
868	Seretide 250 Evohaler (DE Pharmaceuticals)	ICS_LABA
869	Seretide 250 Evohaler (GlaxoSmithKline UK Ltd)	ICS_LABA
870	Seretide 250 Evohaler (Lexon (UK) Ltd)	ICS_LABA
871	Seretide 250 Evohaler (Necessity Supplies Ltd)	ICS_LABA
872	Seretide 250 Evohaler (Stephar (U.K.) Ltd)	ICS_LABA
873	Seretide 250 Evohaler (Waymade Healthcare Plc)	ICS_LABA
874	Seretide 50 Evohaler	ICS_LABA
875	Seretide 50 Evohaler (GlaxoSmithKline UK Ltd)	ICS_LABA
876	Seretide 500 Accuhaler	ICS_LABA
877	Seretide 500 Accuhaler (DE Pharmaceuticals)	ICS_LABA
878	Seretide 500 Accuhaler (GlaxoSmithKline UK Ltd)	ICS_LABA
879	Seretide 500 Accuhaler (Lexon (UK) Ltd)	ICS_LABA
880	Seretide 500 Accuhaler (Mawdsley-Brooks & Company Ltd)	ICS_LABA
881	Seretide 500 Accuhaler (Necessity Supplies Ltd)	ICS_LABA
882	Seretide 500 Accuhaler (Waymade Healthcare Plc)	ICS_LABA
883	Serevent 25micrograms/dose Evohaler	LABA
884	Serevent 25micrograms/dose Evohaler (GlaxoSmithKline UK Ltd)	LABA
885	Serevent 25micrograms/dose Evohaler (Lexon (UK) Ltd)	LABA
886	Serevent 25micrograms/dose Evohaler (Waymade Healthcare Plc)	LABA
887	Serevent 25micrograms/dose inhaler	LABA
888	Serevent 50microgram disks (GlaxoSmithKline UK Ltd)	LABA
889	Serevent 50microgram disks with Diskhaler	LABA
890	Serevent 50microgram disks with Diskhaler (GlaxoSmithKline UK Ltd)	LABA
891	Serevent 50micrograms/dose Accuhaler	LABA
892	Serevent 50micrograms/dose Accuhaler (DE Pharmaceuticals)	LABA

For peer review only

For peer review only - http://bmjopen.bmj.com/site/about/guidelines.xhtml

893	Serevent 50micrograms/dose Accuhaler (GlaxoSmithKline UK Ltd)	LABA
894	Serevent 50micrograms/dose Accuhaler (Mawdsley-Brooks & Company Ltd)	LABA
895	Serevent 50micrograms/dose Accuhaler (Waymade Healthcare Plc)	LABA
896	Serevent diskhaler 50microgram Inhalation powder (Glaxo Wellcome UK Ltd)	LABA
897	Singulair 10mg tablets (DE Pharmaceuticals)	montelukast
898	Singulair 10mg tablets (Merck Sharp & Dohme Ltd)	montelukast
899	Singulair 10mg tablets (Necessity Supplies Ltd)	montelukast
900	Singulair Paediatric 4mg chewable tablets (Merck Sharp & Dohme Ltd)	montelukast
901	Singulair Paediatric 4mg granules sachets (Mawdsley-Brooks & Company Ltd)	montelukast
902	Singulair Paediatric 4mg granules sachets (Merck Sharp & Dohme Ltd)	montelukast
903	Singulair Paediatric 5mg chewable tablets (Merck Sharp & Dohme Ltd)	montelukast
904	Sirdupla 25micrograms/dose / 125micrograms/dose inhaler	ICS_LABA
905	Sirdupla 25micrograms/dose / 125micrograms/dose inhaler (Mylan)	ICS_LABA
906	Sirdupla 25micrograms/dose / 250micrograms/dose inhaler	ICS_LABA
907	Sirdupla 25micrograms/dose / 250micrograms/dose inhaler (Mylan)	ICS_LABA
908	Sirdupla 25micrograms/dose / 250micrograms/dose inhaler (Waymade Healthcare Plc)	ICS_LABA
909	Slo-phyllin 125mg Capsule (Lipha Pharmaceuticals Ltd)	other
910	Slo-Phyllin 125mg capsules (Merck Serono Ltd)	other
911	Slo-phyllin 250mg Capsule (Lipha Pharmaceuticals Ltd)	other
912	Slo-Phyllin 250mg capsules (Merck Serono Ltd)	other
913	Slo-phyllin 60mg Capsule (Lipha Pharmaceuticals Ltd)	other
914	Slo-Phyllin 60mg capsules (Merck Serono Ltd)	other
915	Sodium cromoglicate 1mg/dose / Salbutamol 100micrograms/dose inhaler	other
916	Sodium cromoglicate 1mg/dose / Salbutamol 100micrograms/dose inhaler with spacer	other
917	Sodium cromoglicate 1mg/inhalation inhaler	other
918	Sodium cromoglicate 20mg/2ml nebuliser liquid unit dose vials	other
919	Sodium cromoglicate 5mg/dose inhaler CFC free	other
920	Sodium cromoglicate 5mg/inhalation inhaler & spacer	other
921	Sodium cromoglicate with isoprenaline inhalation capsules	other
922	Soltel 25micrograms/dose inhaler CFC free	LABA
923	Soltel 25micrograms/dose inhaler CFC free (Kent Pharmaceuticals Ltd)	LABA
924	Soprobec 100micrograms/dose inhaler	ICS
925	Soprobec 200micrograms/dose inhaler	ICS
926	Soprobec 250micrograms/dose inhaler	ICS
927	Soprobec 50micrograms/dose inhaler	ICS
928	Spacehaler BDP 100microgram/actuation Spacehaler (Celltech Pharma Europe Ltd)	other
929	Spacehaler BDP 250microgram/actuation Spacehaler (Celltech Pharma Europe Ltd)	other
930	Spacehaler BDP 50microgram/actuation Spacehaler (Celltech Pharma Europe Ltd)	other
931	Spacehaler salbutamol 100microgram/inhalation Spacehaler (Celltech Pharma Europe Ltd)	other
932	Spiolto Respimat 2.5microg/dose / 2.5microg/dose soln refill	LAMA_LABA
933	Spiolto Respimat 2.5microg/dose/2.5microg/dose soln	LAMA_LABA
934	Spiolto Respimat 2.5micrograms/dose / 2.5micrograms/dose solution for inhalation cartridge with device (Boehringer Ingelheim Ltd)	LAMA_LABA
935	Spiriva 18 microgram Capsule (Boehringer Ingelheim Ltd)	LAMA
936	Spiriva 18microgram inhalation pdr caps with HandiHaler	LAMA
937	Spiriva 18microgram inhalation powder capsules	LAMA
938	Spiriva 18microgram inhalation powder capsules (Boehringer Ingelheim Ltd)	LAMA

For peer review only - http://bmjopen.bmj.com/site/about/guidelines.xhtml

939	Spiriva 18microgram inhalation powder capsules (Mawdsley-Brooks & Company Ltd)	LAMA
940	Spiriva 18microgram inhalation powder capsules (Sigma Pharmaceuticals Plc)	LAMA
941	Spiriva 18microgram inhalation powder capsules with HandiHaler (Boehringer Ingelheim Ltd)	LAMA
942	Spiriva 18microgram inhalation powder capsules with HandiHaler (DE Pharmaceuticals)	LAMA
943	Spiriva 18microgram inhalation powder capsules with HandiHaler (Sigma Pharmaceuticals Plc)	LAMA
944	Spiriva 18microgram inhalation powder capsules with HandiHaler (Waymade Healthcare Plc)	LAMA
945	Spiriva Respimat 2.5microg/dose inhalation soln	LAMA
946	Spiriva Respimat 2.5microg/dose inhalation soln refill cart	LAMA
947	Spiriva Respimat 2.5micrograms/dose solution for inhalation cartridge with device (Boehringer Ingelheim Ltd)	LAMA
948	Spiriva Respimat 2.5micrograms/dose solution for inhalation cartridge with device (Waymade Healthcare Plc)	LAMA
949	Stalpex 50microg/dose / 500microg/dose dry powder inh	ICS_LABA
950	Steri-neb cromogen 10mg/ml Nebuliser liquid (IVAX Pharmaceuticals UK Ltd)	other
951	Steri-neb ipratropium 250microgram/ml Nebuliser liquid (IVAX Pharmaceuticals UK Ltd)	LAMA
952	Striverdi Respimat 2.5microg/dose inhalation soln	LABA
953	Striverdi Respimat 2.5microg/dose inhalation soln refill	LABA
954	Striverdi Respimat 2.5micrograms/dose solution for inhalation cartridge with device (Boehringer Ingelheim Ltd)	LABA
955	Symbicort 100/6 Turbohaler	ICS_LABA
956	Symbicort 100/6 Turbohaler (AstraZeneca UK Ltd)	ICS_LABA
957	Symbicort 100/6 Turbohaler (Mawdsley-Brooks & Company Ltd)	ICS_LABA
958	Symbicort 100/6 Turbohaler (Sigma Pharmaceuticals Plc)	ICS_LABA
959	Symbicort 200/6 Turbohaler	ICS_LABA
960	Symbicort 200/6 Turbohaler (AstraZeneca UK Ltd)	ICS_LABA
961	Symbicort 200/6 Turbohaler (DE Pharmaceuticals)	ICS_LABA
962	Symbicort 200/6 Turbohaler (Mawdsley-Brooks & Company Ltd)	ICS_LABA
963	Symbicort 200/6 Turbohaler (Necessity Supplies Ltd)	ICS_LABA
964	Symbicort 200/6 Turbohaler (Sigma Pharmaceuticals Plc)	ICS_LABA
965	Symbicort 200microg/dose / 6microg/dose pressurised inh	ICS_LABA
966	Symbicort 200micrograms/dose / 6micrograms/dose pressurised inhaler (AstraZeneca UK Ltd)	ICS_LABA
967	Symbicort 400/12 Turbohaler	ICS_LABA
968	Symbicort 400/12 Turbohaler (AstraZeneca UK Ltd)	ICS_LABA
969	Symbicort 400/12 Turbohaler (DE Pharmaceuticals)	ICS_LABA
970	Symbicort 400/12 Turbohaler (Mawdsley-Brooks & Company Ltd)	ICS_LABA
971	Tedral Oral solution (Parke-davis Research Laboratories)	other
972	Tedral Tablet (Parke-davis Research Laboratories)	other
973	Terbutaline 1.5mg/5ml Oral solution (Sandoz Ltd)	SABA
974	Terbutaline 1.5mg/5ml oral solution sugar free	SABA
975	Terbutaline 2.5mg/5ml solution for injection ampoules	SABA
976	Terbutaline 250micrograms/actuation refill canister	SABA
977	Terbutaline 500micrograms/1ml solution for injection ampoules	SABA
978	Terbutaline 500micrograms/dose dry powder inhaler	SABA
979	Terbutaline 500micrograms/ml injection	SABA
980	Terbutaline 5mg tablets	SABA
981	Terbutaline 5mg/2ml nebuliser liquid unit dose vials	SABA
982	Terbutaline with guafenesin expectorant	SABA
983	Theodrox Tablet (3M Health Care Ltd)	other

For peer review only - http://bmjopen.bmj.com/site/about/guidelines.xhtml

984	Theo-Dur 200mg tablets (AstraZeneca UK Ltd)	other
985	Theo-Dur 300mg tablets (AstraZeneca UK Ltd)	other
986	Theograd 350mg Tablet (Abbott Laboratories Ltd)	other
987	Theophylline 10mg/5ml SF elixir	other
988	Theophylline 125mg modified-release capsules	other
989	Theophylline 125mg/5ml syrup	other
990	Theophylline 175mg modified-release tablets	other
991	Theophylline 200mg modified-release tablets	other
992	Theophylline 250mg modified-release capsules	other
993	Theophylline 250mg modified-release tablets	other
994	Theophylline 250mg/5ml oral suspension	other
995	Theophylline 300mg modified release capsules	other
996	Theophylline 300mg modified-release tablets	other
997	Theophylline 350mg modified release tablets	other
998	Theophylline 400mg modified-release tablets	other
999	Theophylline 500mg modified release tablets	other
1000	Theophylline 60mg modified-release capsules	other
1001	Theophylline 60mg/5ml oral solution	other
1002	Theophylline 60mg/5ml oral suspension	other
1003	Theophylline with ephedrine & caffeine tablets	other
1004	Tilade 2mg/dose inhaler CFC free (Sanofi)	nil
1005	Tilade 2mg/inhalation Inhalation powder (Sanofi)	nil
1006	Tilarin 1% Nasal spray suspension (Rhone-Poulenc Rorer Ltd)	nil
1007	Tiotropium 18 microgram Capsule	LAMA
1008	Tiotropium brom 2.5microg/dose soln for inhalation CFCfree	LAMA
1009	Tiotropium brom 2.5microg/Olodaterol 2.5microg/dose soln	LAMA_LABA
1010	Tiotropium brom 2.5microg/Olodaterol 2.5microg/dose soln&dev	LAMA_LABA
1011	Tiotropium bromide 10microgram inhalation pdr caps with dev	LAMA
1012	Tiotropium bromide 10microgram inhalation powder capsules with device	LAMA
1013	Tiotropium bromide 18microgram inhalation pdr caps with dev	LAMA
1014	Tiotropium bromide 18microgram inhalation powder capsules	LAMA
1015	Tiotropium bromide 18microgram inhalation powder capsules with device	LAMA
1016	Tiotropium bromide 2.5micrograms/dose / Olodaterol 2.5micrograms/dose solution for inhalation cartridge with device CFC free	LAMA_LABA
1017	Tiotropium bromide 2.5micrograms/dose inhalation soln cart	LAMA
1018	Tiotropium bromide 2.5micrograms/dose solution for inhalation cartridge with device CFC free	LAMA
1019	Tiotropium bromide 2.5micrograms/dose solution for inhalation cartridge with device CFC free (AM Distributions (Yorkshire) Ltd)	LAMA
1020	Trelegy Ellipta 92microg/55microg/22microg/dose dry pdr inh	triple
1021	Trimbow 87microg/dose / 5microg/dose / 9microg /dose inh	triple
1022	Tropiovent steripoule 250microgram/ml Nebuliser liquid (Ashbourne Pharmaceuticals Ltd)	LAMA
1023	Tulobuterol 1mg/5ml sugar free syrup	LABA
1024	Tulobuterol 2mg	LABA
1025	Ultibro Breezhaler 85microg/43microg pdr caps with dev	LAMA_LABA
1026	Ultibro Breezhaler 85microgram/43microgram inhalation powder capsules with device (Novartis Pharmaceuticals UK Ltd)	LAMA_LABA
1027	Umeclidinium brom 65microg/Vilanterol 22microg/dose dry pdr	LAMA_LABA
1028	Umeclidinium bromide 65micrograms/dose / Vilanterol 22micrograms/dose dry powder inhaler	LAMA_LABA

For peer review only - http://bmjopen.bmj.com/site/about/guidelines.xhtml

1029	Umeclidinium bromide 65micrograms/dose dry powder inhaler	LAMA
1030	Uniphyllin Continus 200mg tablets (Napp Pharmaceuticals Ltd)	other
1031	Uniphyllin Continus 300mg tablets (Napp Pharmaceuticals Ltd)	other
1032	Uniphyllin Continus 400mg tablets (Napp Pharmaceuticals Ltd)	other
1033	Ventmax SR 4mg capsules (Chiesi Ltd)	SABA
1034	Ventmax SR 8mg capsules (Chiesi Ltd)	SABA
1035	Ventodisks 200microgram/blister Disc (Allen & Hanburys Ltd)	SABA
1036	Ventodisks 400microgram/blister Disc (Allen & Hanburys Ltd)	SABA
1037	VENTOLIN	SABA
1038	VENTOLIN .25 MG INJ	SABA
1039	Ventolin 100microgram/inhalation Inhalation powder (Glaxo Wellcome UK Ltd)	SABA
1040	Ventolin 100micrograms/dose Evohaler (DE Pharmaceuticals)	SABA
1041	Ventolin 100micrograms/dose Evohaler (GlaxoSmithKline UK Ltd)	SABA
1042	Ventolin 100micrograms/dose Evohaler (Mawdsley-Brooks & Company Ltd)	SABA
1043	Ventolin 100micrograms/dose Evohaler (Waymade Healthcare Plc)	SABA
1044	Ventolin 2.5mg Nebules (GlaxoSmithKline UK Ltd)	SABA
1045	Ventolin 2.5mg Nebules (Mawdsley-Brooks & Company Ltd)	SABA
1046	Ventolin 200microgram Rotacaps (GlaxoSmithKline UK Ltd)	SABA
1047	Ventolin 200micrograms/dose Accuhaler (DE Pharmaceuticals)	SABA
1048	Ventolin 200micrograms/dose Accuhaler (Dowelhurst Ltd)	SABA
1049	Ventolin 200micrograms/dose Accuhaler (GlaxoSmithKline UK Ltd)	SABA
1050	Ventolin 200micrograms/dose Accuhaler (Lexon (UK) Ltd)	SABA
1051	Ventolin 200micrograms/dose Accuhaler (Mawdsley-Brooks & Company Ltd)	SABA
1052	Ventolin 200micrograms/dose Accuhaler (Sigma Pharmaceuticals Plc)	SABA
1053	Ventolin 200micrograms/dose Accuhaler (Waymade Healthcare Plc)	SABA
1054	Ventolin 2mg Tablet (Allen & Hanburys Ltd)	SABA
1055	Ventolin 2mg/5ml syrup (GlaxoSmithKline UK Ltd)	SABA
1056	Ventolin 400microgram Rotacaps (GlaxoSmithKline UK Ltd)	SABA
1057	Ventolin 4mg Tablet (Allen & Hanburys Ltd)	SABA
1058	Ventolin 500micrograms/1ml solution for injection ampoules (GlaxoSmithKline UK Ltd)	SABA
1059	Ventolin 50microgram/ml Injection (Allen & Hanburys Ltd)	SABA
1060	Ventolin 5mg Nebules (GlaxoSmithKline UK Ltd)	SABA
1061	Ventolin 5mg/5ml solution for infusion ampoules (GlaxoSmithKline UK Ltd)	SABA
1062	Ventolin 5mg/ml respirator solution (GlaxoSmithKline UK Ltd)	SABA
1063	Ventolin accuhaler 200 200microgram/actuation Inhalation powder (Glaxo Wellcome UK Ltd)	SABA
1064	Ventolin cr 4mg Tablet (Allen & Hanburys Ltd)	SABA
1065	Ventolin cr 8mg Tablet (Allen & Hanburys Ltd)	SABA
1066	Ventolin easi-breathe 100microgram/actuation Pressurised inhalation (Allen & Hanburys Ltd)	SABA
1067	Ventolin evohaler 100 100microgram/inhalation Pressurised inhalation (Glaxo Wellcome UK Ltd)	SABA
1068	VENTOLIN I/V 5 MG INJ	SABA
1069	VENTOLIN NEBULES	SABA
1070	VENTOLIN RESPIRATOR	SABA
1071	VENTOLIN ROTACAPS	SABA
1072	VENTOLIN ROTAHALER	SABA
1073	Ventolin Rotahaler (GlaxoSmithKline UK Ltd)	SABA
1074	VENTOLIN S/R	SABA

For peer review only - http://bmjopen.bmj.com/site/about/guidelines.xhtml

1075	VENTOLIN S/R 8 MG SPA	SABA
1076	Vertine 25micrograms/dose inhaler CFC free	LABA
1077	Vertine 25micrograms/dose inhaler CFC free (Teva UK Ltd)	LABA
1078	Yanimo Respimat 2.5microg/dose / 2.5microg/dose soln	LAMA_LABA
1079	Zafirlukast 20mg tablets	other

For peer review only

For peer review only - http://bmjopen.bmj.com/site/about/guidelines.xhtml

Supplementary Material C: Detail of Approach to Cluster Analysis

Data were analysed in *RStudio* version 1.2.5033 (driven by *R* version 3.6.0) on a Windows 10 desktop computer.[1] Additional packages were loaded from The Comprehensive R Archive Network (https://cran.r-project.org/).[2]

There are a variety of methods and tools which can be used for population segmentation to help enable integrated healthcare models.[3] A comprehensive review of population segmentation methods is provided by Wood, Murch and Betteridge.[4] Data driven population segmentation has been applied for Population Health Management (PHM) for a wide variety of different clinical contexts.[5] The most commonly used data driven segmentation analysis methods are latent class, k-means cluster analysis and hierarchical analysis.[5] Cluster analysis is an unsupervised machine learning approach which can be used to identify patterns and attributes between different segments of people. Clustering is used to group data into similar groups which are designated as 'clusters'. Machine learning methods are only recently being applied to PHM. The linked dataset can be considered big data with different types of variables in which nonparametric machine learning approaches may be best placed for certain types of analyses. Although more statistical based approaches such as decision trees are recommended for utilisation based population segmentation studies,[4] cluster analysis is a useful non-objective exploratory tool when there are no a priori hypotheses or particular outcomes to model. There are several clustering methods which can be applied to larger datasets including k-means, k-modes and k-prototypes (which is in essence a combination of both the k-means and k-modes algorithms). K-means is typically used for continuous data types and requires the value of k (the number of clusters) to be determined upfront. K-modes is an analogue of k-means but requires categorical data.

The clustering method used for this analysis was k-prototypes. This method allows clustering on mixed data types (both continuous variables and categorical). This algorithm can also handle larger datasets where hierarchical clustering methods may be impractical. The R package 'clustMixType' was used for analyses which is based on Huang's k-prototypes algorithm.[2 6 7] This algorithm uses the Euclidean distance measure (a dissimilarity metric to measure similarity between observations) for numerical data and simple matching dissimilarity measures for categorical data. K-prototypes has been previously employed to identify vulnerable populations for malaria in the Yunnan Province of China.[8] A summary of the cluster analysis methods is given in Table C.1 below which covers the 5 reporting criteria by Aldenderfer and Blashfield.[9 10]

Reporting Criteria	
1 – Computer Software	R, clustMixType Package,[6] based on Huang's k-prototypes algorithm[7]
2 – Similarity Measure	The Euclidean distance measure for numerical data and simple matching dissimilarity measures for categorical data
3 – Cluster Method	k-prototypes
4 – Decision on number of clusters	Both data driven and through interpretation. Scree plot showing elbow point for total within sum of squares by number of clusters and Silhouette Index. Use of 6 criteria reported by Chong, Lim and Matchar for healthcare segmentation including actionability, identifiability and responsiveness.
5 – Evidence of Cluster Validity	Expert Review - Clinician Review of clusters produced for 6,7,8 and 9 cluster solutions, Silhouette Index, external comparison with literature.

Table C.1: 5 Reporting Criteria for cluster analysis by Aldenderfer and Blashfield.[9 10]

The number of clusters (or segments) was determined both empirically from the data and by interpretation of the segments and clinical context. The total within sum of squares was plotted across different numbers of clusters (1-10) to identify a point in which the curve begins to flatten out. In addition, the Silhouette index was applied to assess cluster validity where the maximum value of the index was used to determine the optimal number of clusters.[11] A consideration of the healthcare context was used in order that each segment has a 'relatively substantial' portion of the sample population as well as being responsive and actionable in order to benefit from service based intervention.[3] A population segmentation scheme for healthcare policy can be determined through 6

For peer review only - http://bmjopen.bmj.com/site/about/guidelines.xhtml

criteria described by Chong, Lim and Matchar (2019) as substantiality, responsiveness, actionability, identifiability and accessibility.[3]

The k-protoypes algorithm repeats until a stopping criterion is reached, therefore the maximum number of iterations used for the algorithm was 1,000,000 to enable the algorithm to converge. In addition, to overcome the random component of the algorithm (variation arising due to random choice of the initial prototype observations) it was run 1000 times with the best outcome across all runs being selected as the single outcome. As well as the number of clusters needing to be determined, another parameter 'λ' which represents the trade-off between numerical data (Euclidean distance) and categorical data (simple matching distance) also needs to be selected. 'λ' was determined as 3.06 based on the average variance over all numeric variables related to the average concentration of all categorical variables.[2]

The k-prototypes clustering method was applied to 6,7,8 and 9 clusters and these were reviewed by two clinicians and assessed for their usefulness in terms of the criteria discussed above including identifiability and actionability. Cluster profiles were also produced to provide further information on the homogeneity of clusters (cluster profiles for the 6 cluster solution is given in Figure C.2 below). Cluster stability was observed since 7 clusters reproduced the groups identified for the 6 cluster solution but with an additional cancer group with a higher occurrence of non-elective admissions. Similar groupings were found for 8 and 9 clusters, but they produced separate groups for the same condition for male and female which could be better captured within one cluster. Ultimately 6 clusters were selected based on a combination of these investigations (see Table C.4-C.6 for the results of this clustering).

Number of Clusters	Within Sum of Squares	Silhouette Index
2	309,757.1	0.160
3	286,488.8	0.174
4	264,354.7	0.190
5	243,689.9	0.187
6	227,313.3	0.192
7	217,342.0	0.196
8	206,578.7	0.178
9	196,894.5	0.193
10	191,006.9	0.184

Table C.2: Indexes to assess cluster validity. The maximum value of the Silhouette index is used to determine the optimal number of clusters (20 nstarts, 100,000 iterations). The within sum of squares values are plotted on a scree plot to determine an elbow point when the curve begins to flatten out.

Data were pre-processed before cluster analyses to ensure variables were on the same scale (continuous variables were scaled). A smaller proportion of the population use a large proportion of available healthcare which can skew the data. As such, the top 0.1% of people with the highest healthcare utilisation were excluded from the analyses to aid with the clustering process as clustering algorithms can be sensitive to outliers. Removing the top utilisers (0.4%) left a high-risk population of 29,454. The attributes considered included those cited as core indicators[12] and through discussion with clinicians. Previous work by the authors suggest including utilisation variables to add increased discriminative ability. Using the number of co-morbid conditions has been shown to act as a proxy for the care needs of a group.[13] Mental health needs of the high-risk group who have been asked to isolate for 12 weeks will be included by assessing number of previous mental health appointments in the past 12 months along with community support accessed during the last 12 months. Utilisation variables consisted of combined appointments since the more variables included in the clustering, the more they will dilute other variables and so the following strategy was employed to maintain a good balance of information: Primary care and community care contacts were combined, secondary care elective appointments included both outpatient and in-patient appointments and secondary care non-elective appointments included A&E and non-elective in- patient appointments. Clustering variables were included based on

For peer review only

whether the variable could assist the following: targeting advice/guidance, identification of those most in need to home deliveries of critical supplies, informing distribution of healthcare resources and services. Variables were grouped where appropriate to ensure there were not too many variables which may dilute the effects of others. Full List shown in **Table 1** below.

Variable	How the Variable is Operationalised
**Clustering Variables**	
**Demographics**	
Age	Continuous: Current age
Sex	Categorical: Male/Female
**Healthcare Utilisation (calendar year 2019)**	
Primary Care (GP) and Community contacts (previous calendar year 2019)	Continuous count
Mental health attendances (previous calendar year 2019)	Continuous count
Secondary care elective (outpatient and elective in patient appointments) (previous calendar year 2019)	Continuous count
Secondary care non-elective (accident and emergency and non-elective in patient appointments) (previous calendar year 2019)	Continuous count
**Co-morbidities and other health conditions**	
Cardiovascular condition (current)	Binary: Based on the presence of myocardial infarction or heart failure or peripheral vascular disease or stroke
Cancer diagnosed in the past 5 years	Binary: Based on the presence of the following in the past 5 years: lung cancer, breast cancer, bowel cancer, prostate cancer, leukaemia/lymphoma, cervical cancer, ovarian cancer, melanoma, head and neck cancer, upper GI/liver cancer, other cancer, metastatic cancer, bladder cancer, kidney cancer
Mental Health	Binary: Based on the presence of depression or QoF mental health
Diabetes	Binary: Based on diabetes no complications, diabetes end complications, QoF diabetes
Dementia	Binary: Based on a record of Dementia
Asthma	Binary: Based on if there is a record for asthma and an asthma related prescription in the preceding 12 months (March 2019 to March 2020)
COPD	Binary: Based on a record of COPD
**Other**	
Drugs that require monitoring or administrating	Binary: Based on a record of medication that requires monitoring in the previous 2 months ('Other' drugs requiring monitoring in the previous 2 months – full list of drugs in **Supplementary Material B**)<br><br>Or a record of a prescription of an immunosuppressant/immunomodulator (IMMIMM) or biologic/monoclonal (IMMBIO) in the previous 6 months (or previous 12 months for rituximab).<br><br>Full lists of drugs included in **Supplementary Material B**
**Non clustering variables**	
**Lifestyle Factors**	
Smoking	Categorical: current smoker, non-smoker
IMD Decile	Continuous: Based on current LSOA as recorded by GP practice<br>The Index of Multiple Deprivation (IMD) ranks LSOAs from most to least deprived on a scale from one to ten
Urban/Rural	Categorical: Urban City and Town/Rural town and fringe/Rural village and dispersed<br>Using the Rural Urban Classification (2011) of Lower Layer Super Output Areas in England and Wales.
Learning disabilities and autism	Based on a QoF record of a learning disability and a record of autism
Housebound	Binary variable
Has a carer	Binary variable
Is a carer	Binary variable
Charlson comorbidity score[14]	Continuous

Table C.3: Clustering and non-clustering variables used for analyses and how they are operationalised.

The resulting segments/clusters were then analysed by assessing the different attributes, comorbidities and utilisation variables. Additional factors of interest were also summarised for each of the clusters (non-cluster variables). These included social status factors such as whether someone is housebound or whether they have a carer to determine if a particular cluster may benefit from having medications or food delivered or to ensure they are still receiving appropriate healthcare. Both clustering variables and

For peer review only - http://bmjopen.bmj.com/site/about/guidelines.xhtml

non-clustering variables were assessed for whether they were statistically different from other clusters, an approach used by other studies to verify results and to indicate how segments differ on each clustering variable.[15 16] Firstly an omnibus statistical test was applied (ANOVA, Kruskal-Wallis, Chi square test) to confirm differences across clusters. ANOVA for example determines if at least one group mean differs from the others and the Kruskal-Wallis test is the non-parametric equivalent. This omnibus testing was followed by post hoc pairwise comparisons. 15 pairwise tests between all other clusters were used (t-test, Mann-Whitney U test and z-test for proportions) to assign whether the cluster was significantly different from 5, 4, or 3 other clusters indicated with a superscript a,b or c. A Bonferroni adjustment was made to the significance level of 0.05 due to using multiple test comparisons which increases the probability of at least one false rejection of the null hypothesis (0.05/15 = 0.0033 (based on 15 pairwise tests).

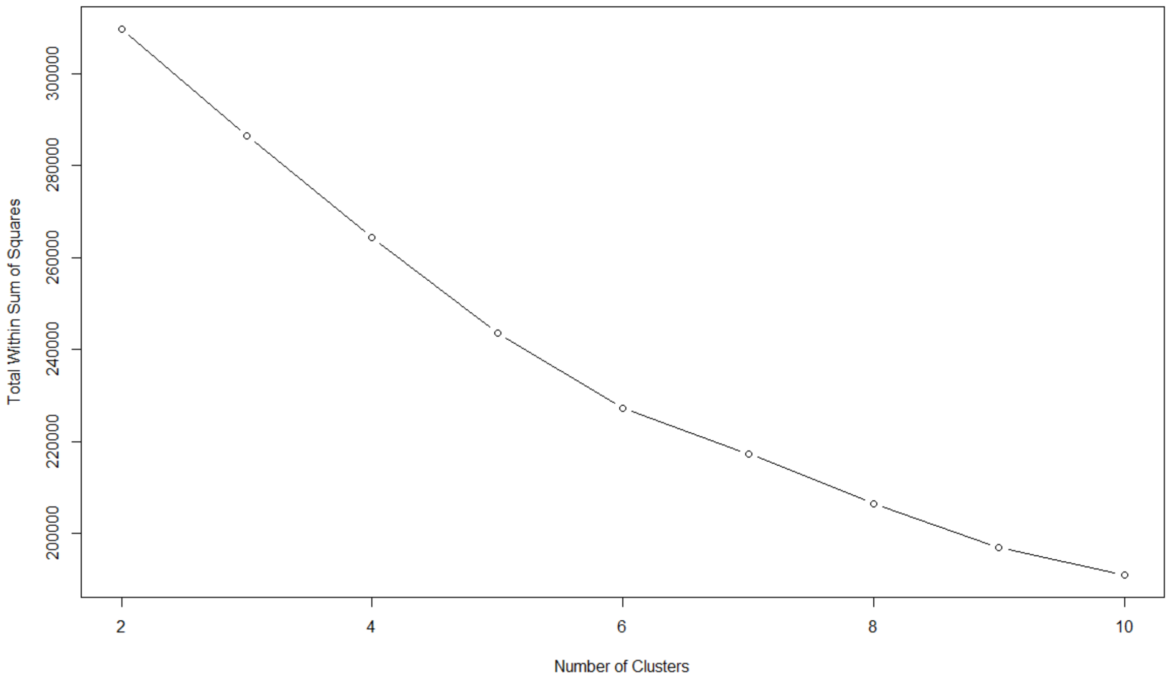

Figure C.1: Scree plot showing the Total within sum of squares for differing numbers of clusters used in the K-prototypes clustering model. There is an elbow point for 6 clusters and so this was investigated for population segmentation. The models used 50 nstarts (repetitive computations with random initialisations with selection of the result with minimum distance) and 100,000 iterations to produce this plot.

For peer review only - http://bmjopen.bmj.com/site/about/guidelines.xhtml

7 Clusters

Cluster	Cluster 1	Cluster 2	Cluster 3	Cluster 4	Cluster 5	Cluster 6	Cluster 7	Total Population
**Total**	**5066**	**1874**	**6772**	**8649**	**5684**	**1241**	**168**	**29,454**
**Proportion of population**	**17.20%**	**6.36%**	**22.99%**	**29.36%**	**19.30%**	**4.21%**	**0.57%**	**100.00%**
**Clustering Variables**								
Age	52	61	69	73	68	77	58	68
Median, (IQR)	35-65	49-71	57-77	65-80	56-76	68-84	49.75-70.25	55-77
Sex								
-Female	3760 (74.22)	1420 (75.77)	4725 (69.77)	2856 (33.02)	1626 (28.61)	782 (63.01)	105 (62.5)	15274 (51.86)
-Male	1306 (25.78)	454 (24.23)	2047 (30.23)	5793 (66.98)	4058 (71.39)	459 (36.99)	63 (37.5)	14180 (48.14)
Primary Care (GP) and Community contacts	4	6	4	5	4	54	10	5
Median, (IQR)	2-9	2.25-13	2-8	2-9	1-7	29-87	5-20.25	2-10
Mental health attendances	0	0	0	0	0	0	46	0
Median, (IQR)	0-0	0-0	0-0	0-0	0-0	0-0	34-62.5	0-0
Secondary care elective (outpatient and elective in patient appointments)	5	42	6	3	11	10	6	6
Median, (IQR)	1-11	32-54	3-12	0-7	5-17	5-19	2-13	2-14
Secondary care non-elective (accident and emergency and non-elective in patient appointments)	0	1	0	0	0	6	2	0
Median, (IQR)	0-2	0-3	0-1	0-1	0-1	3-9	0-3	0-2
Cardiovascular condition (current)	449 (8.86)	209 (11.15)	1082 (15.98)	2492 (28.81)	923 (16.24)	567 (45.69)	27 (16.07)	5749 (19.52)
Cancer diagnosed in the past 5 years	260 (5.13)	1397 (74.55)	198 (2.92)	360 (4.16)	4641 (81.65)	200 (16.12)	25 (14.88)	7081 (24.04)
Mental Health	1131 (22.33)	311 (16.6)	1034 (15.27)	1417 (16.38)	661 (11.63)	286 (23.05)	132 (78.57)	4972 (16.88)
Diabetes	734 (14.49)	259 (13.82)	1368 (20.2)	1959 (22.65)	1108 (19.49)	387 (31.18)	39 (23.21)	5854 (19.88)
Dementia	39 (0.77)	8 (0.43)	190 (2.81)	337 (3.9)	81 (1.43)	104 (8.38)	18 (10.71)	777 (2.64)
Asthma	3969 (78.35)	186 (9.93)	492 (7.27)	1331 (15.39)	240 (4.22)	297 (23.93)	54 (32.14)	6569 (22.3)
COPD	1076 (21.24)	134 (7.15)	400 (5.91)	7617 (88.07)	247 (4.35)	739 (59.55)	76 (45.24)	10289 (34.93)
Drugs that require monitoring	644 (12.71)	185 (9.87)	5330 (78.71)	404 (4.67)	241 (4.24)	229 (18.45)	67 (39.88)	7100 (24.11)
Smoking								
-Non smoker	4204 (82.98)	1691 (90.23)	6105 (90.15)	6442 (74.48)	5066 (89.13)	1056 (85.09)	106 (63.1)	24670 (83.76)
-Current smoker	862 (17.02)	183 (9.77)	667 (9.85)	2207 (25.52)	618 (10.87)	185 (14.91)	62 (36.9)	4784 (16.24)
**Non Clustering Variables**								
Urban/Rural								
-Rural town and fringe	125 (2.47)	60 (3.2)	227 (3.35)	226 (2.61)	191 (3.36)	38 (3.06)	6 (3.57)	873 (2.96)

For peer review only - http://bmjopen.bmj.com/site/about/guidelines.xhtml

-Rural village and dispersed	200 (3.95)	124 (6.62)	399 (5.89)	339 (3.92)	320 (5.63)	33 (2.66)	5 (2.98)	1420 (4.82)
-Urban city and town	4741 (93.58)	1690 (90.18)	6146 (90.76)	8084 (93.47)	5173 (91.01)	1170 (94.28)	157 (93.45)	27161 (92.21)
IMD Decile	5	7	7	5	7	5	4	6
Median, (IQR)	3-8	4-9	4-9	2-8	4-9	3-8	2-7	3-8
Learning disabilities and autism	100 (1.97)	9 (0.48)	30 (0.44)	31 (0.36)	34 (0.6)	19 (1.53)	7 (4.17)	230 (0.78)
Housebound	59 (1.16)	19 (1.01)	181 (2.67)	396 (4.58)	86 (1.51)	321 (25.87)	19 (11.31)	1081 (3.67)
Has a carer	75 (1.48)	40 (2.13)	146 (2.16)	340 (3.93)	107 (1.88)	127 (10.23)	10 (5.95)	845 (2.87)
Is a carer	161 (3.18)	64 (3.42)	266 (3.93)	331 (3.83)	169 (2.97)	63 (5.08)	3 (1.79)	1057 (3.59)
Charlson Score	2	4	4	5	5	6	3	4
Median, (IQR)	(1-4)	(2-6)	(2-5)	(4-6)	(3-6)	(4-7)	(2-5)	(3-6)

Table C.4: Segmentation of high-risk cohort (*n*=29,454) into 7 clusters.

For peer review only

For peer review only - http://bmjopen.bmj.com/site/about/guidelines.xhtml

8 Clusters

Cluster	Cluster 1	Cluster 2	Cluster 3	Cluster 4	Cluster 5	Cluster 6	Cluster 7	Cluster 8	Total Population
**Total**	**5853**	**1925**	**1038**	**5917**	**4893**	**172**	**4113**	**5543**	**29,454**
**Proportion of population**	**19.87%**	**6.54%**	**3.52%**	**20.09%**	**16.61%**	**0.58%**	**13.96%**	**18.82%**	**100.00%**
**Clustering Variables**									
Age	74	60	77	64	72	58	39	73	68
Median, (IQR)	67-81	49-71	68-84	54-73	63-79	49.75-70.25	26-49	65-80	55-77
Sex									
-Female	5853 (100)	1503 (78.08)	623 (60.02)	4358 (73.65)	1558 (31.84)	106 (61.63)	1273 (30.95)	0 (0)	15274 (51.86)
-Male	0 (0)	422 (21.92)	415 (39.98)	1559 (26.35)	3335 (68.16)	66 (38.37)	2840 (69.05)	5543 (100)	14180 (48.14)
Primary Care (GP) and Community contacts	6	7	61	4	4	10	3	4	5
Median, (IQR)	2-12	3-12	34-94.75	2-8	2-8	5-20.25	1-6	2-9	2-10
Mental health attendances	0	0	0	0	0	45	0	0	0
Median, (IQR)	0-0	0-0	0-0	0-0	0-0	32.75-62	0-0	0-0	0-0
Secondary care elective (outpatient and elective in patient appointments)	3	41	10.5	7	11	6	6	3	6
Median, (IQR)	0-8	31-53	5-19	3-13	6-18	2-13	2-12	0-7	2-14
Secondary care non-elective (accident and emergency and non-elective in patient appointments)	0	1	6	0	0	2	0	0	0
Median, (IQR)	0-2	0-3	3-10	0-1	0-1	0-3	0-2	0-1	0-2
Cardiovascular condition (current)	1307 (22.33)	202 (10.49)	566 (54.53)	798 (13.49)	884 (18.07)	28 (16.28)	246 (5.98)	1718 (30.99)	5749 (19.52)
Cancer diagnosed in the past 5 years	284 (4.85)	1435 (74.55)	169 (16.28)	189 (3.19)	4459 (91.13)	25 (14.53)	327 (7.95)	193 (3.48)	7081 (24.04)
Mental Health	1135 (19.39)	344 (17.87)	241 (23.22)	1084 (18.32)	473 (9.67)	136 (79.07)	740 (17.99)	819 (14.78)	4972 (16.88)
Diabetes	1112 (19)	280 (14.55)	340 (32.76)	1192 (20.15)	881 (18.01)	40 (23.26)	678 (16.48)	1331 (24.01)	5854 (19.88)
Dementia	276 (4.72)	8 (0.42)	88 (8.48)	90 (1.52)	88 (1.8)	18 (10.47)	4 (0.1)	205 (3.7)	777 (2.64)
Asthma	1698 (29.01)	257 (13.35)	249 (23.99)	1347 (22.76)	418 (8.54)	55 (31.98)	1304 (31.7)	1241 (22.39)	6569 (22.3)
COPD	4346 (74.25)	116 (6.03)	584 (56.26)	199 (3.36)	224 (4.58)	79 (45.93)	22 (0.53)	4719 (85.13)	10289 (34.93)
Drugs that require monitoring	247 (4.22)	174 (9.04)	210 (20.23)	4975 (84.08)	243 (4.97)	67 (38.95)	784 (19.06)	400 (7.22)	7100 (24.11)
Smoking									
-Non smoker	4573 (78.13)	1741 (90.44)	887 (85.45)	5244 (88.63)	4464 (91.23)	108 (62.79)	3453 (83.95)	4200 (75.77)	24670 (83.76)
-Current smoker	1280 (21.87)	184 (9.56)	151 (14.55)	673 (11.37)	429 (8.77)	64 (37.21)	660 (16.05)	1343 (24.23)	4784 (16.24)
**Non Clustering Variables**									
Urban/Rural									

For peer review only - http://bmjopen.bmj.com/site/about/guidelines.xhtml

-Rural town and fringe	143 (2.44)	60 (3.12)	35 (3.37)	190 (3.21)	183 (3.74)	6 (3.49)	93 (2.26)	163 (2.94)	873 (2.96)
-Rural village and dispersed	239 (4.08)	123 (6.39)	30 (2.89)	332 (5.61)	304 (6.21)	5 (2.91)	145 (3.53)	242 (4.37)	1420 (4.82)
-Urban city and town	5471 (93.47)	1742 (90.49)	973 (93.74)	5395 (91.18)	4406 (90.05)	161 (93.6)	3875 (94.21)	5138 (92.69)	27161 (92.21)
IMD Decile<br>Median, (IQR)	5<br>2-8	7<br>4-9	5<br>3-8	6<br>4-9	7<br>4-9	4<br>2-7	5<br>3-8	5<br>2-8	6<br>3-8
Learning disabilities and autism	9 (0.15)	8 (0.42)	15 (1.45)	28 (0.47)	12 (0.25)	7 (4.07)	125 (3.04)	26 (0.47)	230 (0.78)
Housebound	363 (6.2)	19 (0.99)	283 (27.26)	79 (1.34)	90 (1.84)	19 (11.05)	19 (0.46)	209 (3.77)	1081 (3.67)
Has a carer	230 (3.93)	40 (2.08)	111 (10.69)	89 (1.5)	113 (2.31)	10 (5.81)	36 (0.88)	216 (3.9)	845 (2.87)
Is a carer	289 (4.94)	68 (3.53)	53 (5.11)	225 (3.8)	168 (3.43)	4 (2.33)	60 (1.46)	190 (3.43)	1057 (3.59)
Charlson Score<br>Median, (IQR)	5<br>(4-6)	4<br>(2-6)	6<br>(5-7)	3<br>(2-5)	5<br>(4-7)	3<br>(2-5)	1<br>(0-2)	5<br>(4-6)	4<br>(3-6)

Table C.5: Segmentation of high-risk cohort (*n*=29,454) into 8 clusters.

For peer review only

9 Clusters

Variables	Cluster 1	Cluster 2	Cluster 3	Cluster 4	Cluster 5	Cluster 6	Cluster 7	Cluster 8	Cluster 9	Total Population
**Total**	**170**	**1744**	**3028**	**990**	**4890**	**3483**	**4857**	**4815**	**5477**	**29,454**
**Proportion of population**	**0.58%**	**5.92%**	**10.28%**	**3.36%**	**16.60%**	**11.83%**	**16.49%**	**16.35%**	**18.60%**	**100.00%**
**Clustering Variables**										
Age	58	61	35	77	68	56	75	71	73	68
Median, (IQR)	50.25-70	49-71	22-45	68-85	58-75	48-65	69-82	63-78	65-79	55-77
Sex										
-Female	105 (61.76)	1332 (76.38)	720 (23.78)	583 (58.89)	3400 (69.53)	2774 (79.64)	4857 (100)	1503 (31.21)	0 (0)	15274 (51.86)
-Male	65 (38.24)	412 (23.62)	2308 (76.22)	407 (41.11)	1490 (30.47)	709 (20.36)	0 (0)	3312 (68.79)	5477 (100)	14180 (48.14)
Primary Care (GP) and Community contacts	10	6	2	64	4	5	6	4	4	5
Median, (IQR)	5-20	3-13	1-5	36-96	2-8	2-11	2-12	2-8	1-9	2-10
Mental health attendances	45.5	0	0	0	0	0	0	0	0	0
Median, (IQR)	34-62	0-0	0-0	0-0	0-0	0-0	0-0	0-0	0-0	0-0
Secondary care elective (outpatient and elective in patient appointments)	6	43	6	11	7	5	3	12	3	6
Median, (IQR)	2-13	33-55	2-13	5-20	4-13	1-11	0-8	6-18	0-7	2-14
Secondary care non-elective (accident and emergency and non-elective in patient appointments)	2	1	0	6	0	0	0	0	0	0
Median, (IQR)	0-3	0-3	0-2	3-10	0-1	0-2	0-2	0-1	0-1	0-2
Cardiovascular condition (current)	27 (15.88)	187 (10.72)	172 (5.68)	542 (54.75)	763 (15.6)	335 (9.62)	1161 (23.9)	864 (17.94)	1698 (31)	5749 (19.52)
Cancer diagnosed in the past 5 years	25 (14.71)	1320 (75.69)	312 (10.3)	169 (17.07)	186 (3.8)	202 (5.8)	262 (5.39)	4409 (91.57)	196 (3.58)	7081 (24.04)
Mental Health	134 (78.82)	295 (16.92)	460 (15.19)	229 (23.13)	783 (16.01)	904 (25.95)	880 (18.12)	461 (9.57)	826 (15.08)	4972 (16.88)
Diabetes	39 (22.94)	242 (13.88)	476 (15.72)	331 (33.43)	1033 (21.12)	610 (17.51)	927 (19.09)	866 (17.99)	1330 (24.28)	5854 (19.88)
Dementia	18 (10.59)	5 (0.29)	2 (0.07)	86 (8.69)	92 (1.88)	29 (0.83)	256 (5.27)	89 (1.85)	200 (3.65)	777 (2.64)
Asthma	54 (31.76)	175 (10.03)	399 (13.18)	227 (22.93)	396 (8.1)	3192 (91.65)	807 (16.62)	231 (4.8)	1088 (19.86)	6569 (22.3)
COPD	78 (45.88)	112 (6.42)	22 (0.73)	562 (56.77)	175 (3.58)	531 (15.25)	3860 (79.47)	221 (4.59)	4728 (86.32)	10289 (34.93)
Drugs that require monitoring	67 (39.41)	169 (9.69)	763 (25.2)	203 (20.51)	4488 (91.78)	545 (15.65)	222 (4.57)	243 (5.05)	400 (7.3)	7100 (24.11)
Smoking										
-Non smoker	107 (62.94)	1577 (90.42)	2589 (85.5)	843 (85.15)	4391 (89.8)	2855 (81.97)	3793 (78.09)	4388 (91.13)	4127 (75.35)	24670 (83.76)
-Current smoker	63 (37.06)	167 (9.58)	439 (14.5)	147 (14.85)	499 (10.2)	628 (18.03)	1064 (21.91)	427 (8.87)	1350 (24.65)	4784 (16.24)
**Non Clustering Variables**										
Urban/Rural										

For peer review only

-Rural town and fringe	6 (3.53)	56 (3.21)	72 (2.38)	34 (3.43)	164 (3.35)	91 (2.61)	113 (2.33)	177 (3.68)	160 (2.92)	873 (2.96)
-Rural village and dispersed	5 (2.94)	118 (6.77)	108 (3.57)	27 (2.73)	295 (6.03)	143 (4.11)	200 (4.12)	294 (6.11)	230 (4.2)	1420 (4.82)
-Urban city and town	159 (93.53)	1570 (90.02)	2848 (94.06)	929 (93.84)	4431 (90.61)	3249 (93.28)	4544 (93.56)	4344 (90.22)	5087 (92.88)	27161 (92.21)
IMD Decile Median, (IQR)	4 2-7	7 4-9	5 3-8	5 3-8	7 4-9	5 3-8	5 3-8	7 4-9	5 2-8	6 3-8
Learning disabilities and autism	7 (4.12)	8 (0.46)	110 (3.63)	15 (1.52)	19 (0.39)	27 (0.78)	6 (0.12)	12 (0.25)	26 (0.47)	230 (0.78)
Housebound	19 (11.18)	17 (0.97)	13 (0.43)	276 (27.88)	73 (1.49)	45 (1.29)	342 (7.04)	90 (1.87)	206 (3.76)	1081 (3.67)
Has a carer	10 (5.88)	38 (2.18)	27 (0.89)	109 (11.01)	78 (1.6)	54 (1.55)	202 (4.16)	112 (2.33)	215 (3.93)	845 (2.87)
Is a carer	3 (1.76)	59 (3.38)	35 (1.16)	53 (5.35)	196 (4.01)	124 (3.56)	236 (4.86)	163 (3.39)	188 (3.43)	1057 (3.59)
Charlson Score Median, (IQR)	3 (2-5)	4 (2-6)	1 (0-2)	6 (5-7)	4 (2-5)	3 (2-4)	5 (4-6)	5 (4-7)	5 (4-6)	4 (3-6)

Table C.6: Segmentation of high-risk cohort (*n*=29,454) into 9 clusters.

For peer review only

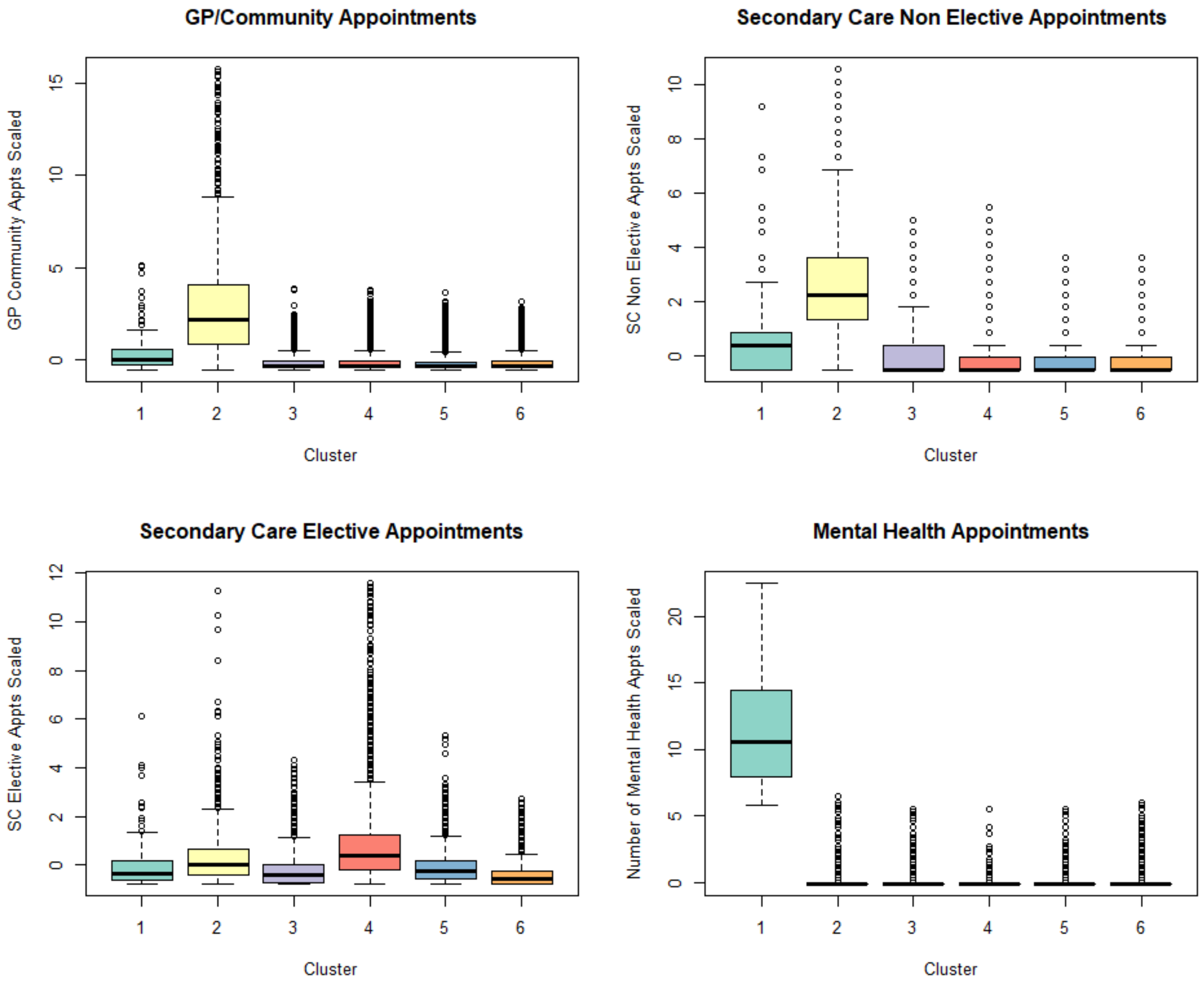

For peer review only - http://bmjopen.bmj.com/site/about/guidelines.xhtml

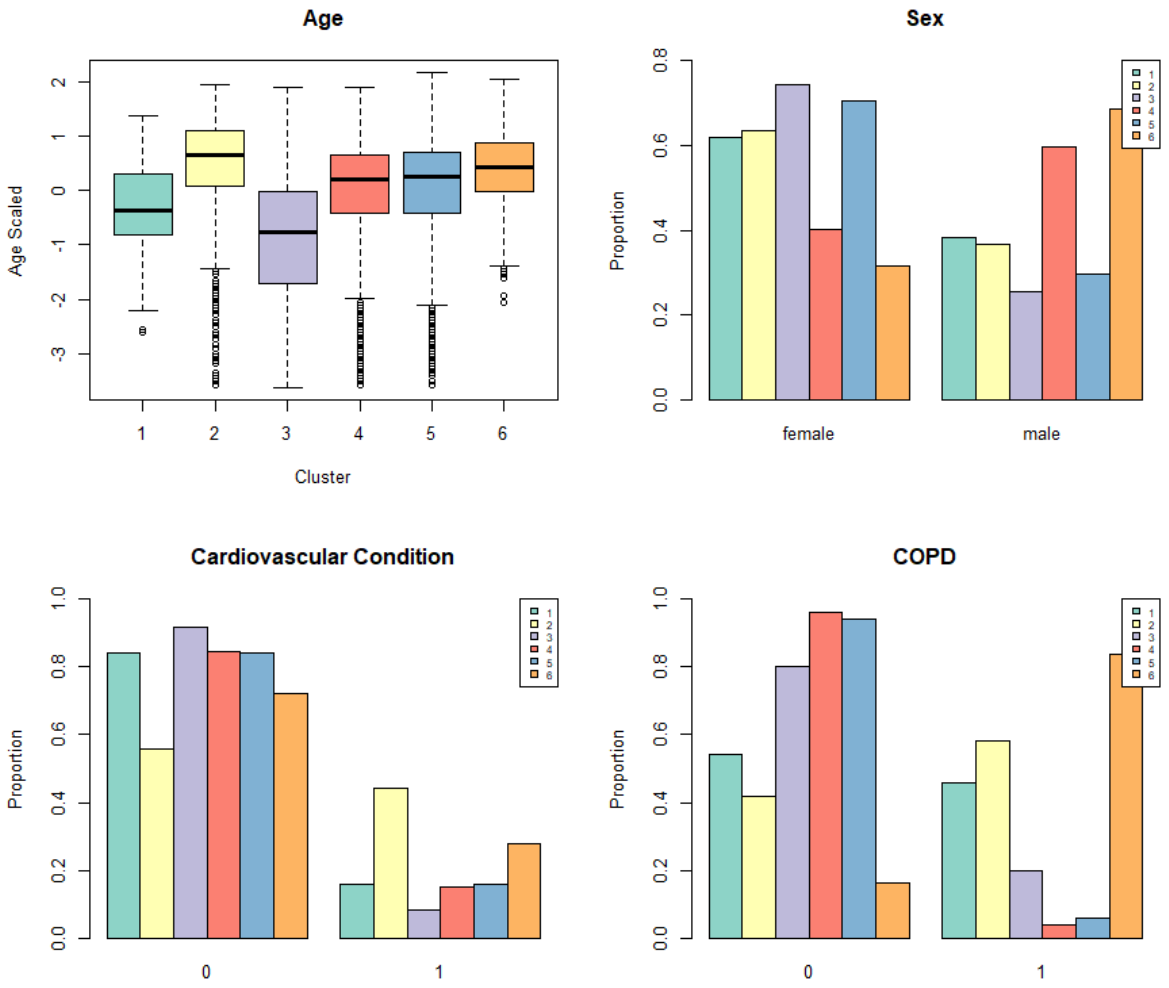

w only

For peer review only - http://bmjopen.bmj.com/site/about/guidelines.xhtml

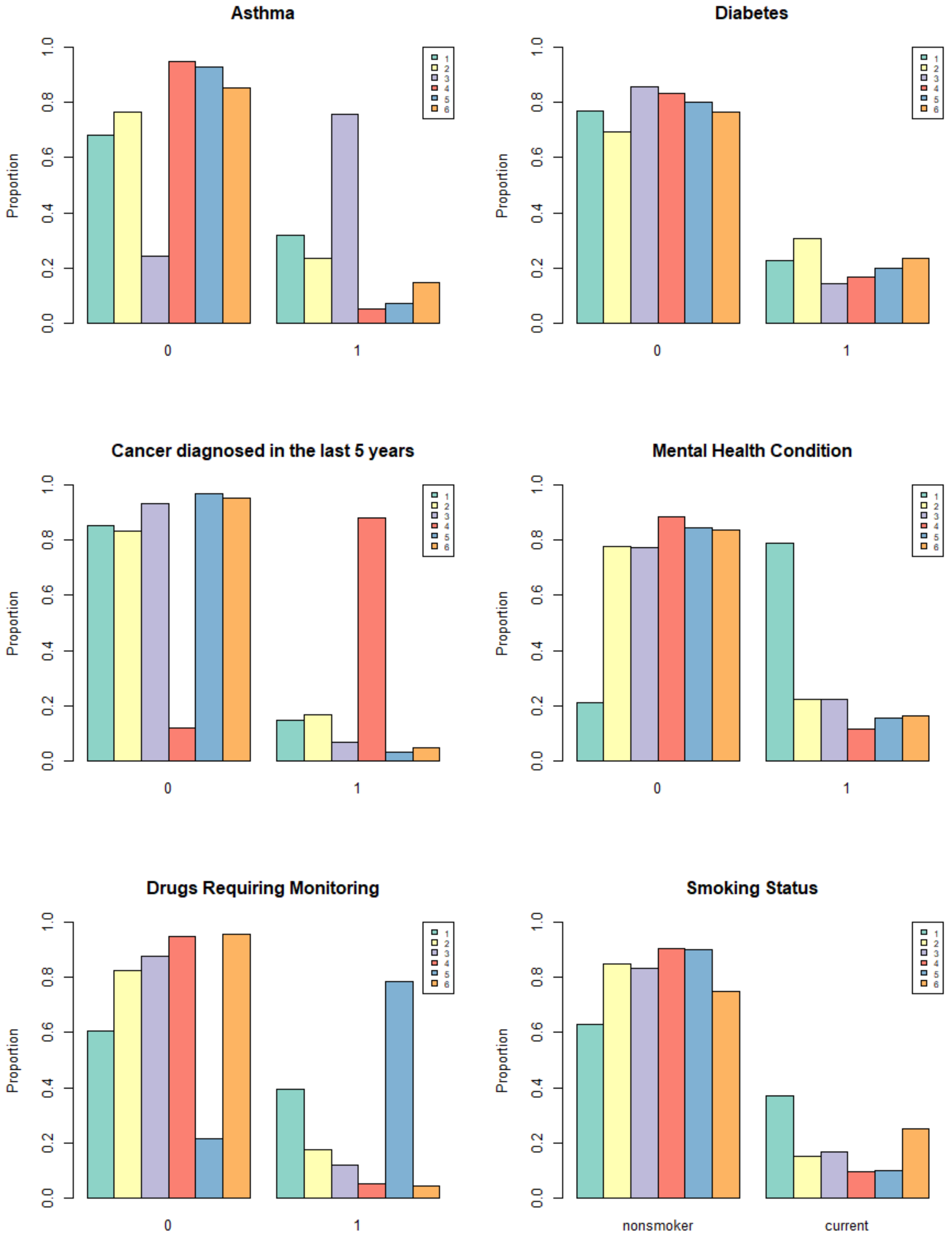

Figure C.2: Cluster profiles produced for the 6 cluster solution to aid interpretability and differentiation of segments. The proportions are shown for binary variables and boxplots for continuous variables.

References for Supplementary Material B:

1. R Core Team. R: A language and environment for statistical computing. R Foundation for Statistical Computing, Vienna, Austria. 2019
2. Szepannek G. clustMixType: User-Friendly Clustering of Mixed-Type Data in R. *The R Journal* 2018;10(2)
3. Chong JL, Lim KK, Matchar DB. Population segmentation based on healthcare needs: a systematic review. *Syst Rev* 2019;8(1):202. doi: 10.1186/s13643-019-1105-6 [published Online First: 2019/08/15]
4. Wood RM, Murch BJ, Betteridge RC. A comparison of population segmentation methods. *Operations Research for Health Care* 2019
5. Yan S, Kwan YH, Tan CS, et al. A systematic review of the clinical application of data-driven population segmentation analysis. *BMC Med Res Methodol* 2018;18(1):121. doi: 10.1186/s12874-018-0584-9 [published Online First: 2018/11/06]
6. Szepannek GA, R. clustMixType: k-Prototypes Clustering for Mixed Variable-Type Data. April 23 2020. *The Comprehensive R Archive Network* 2020
7. Huang Z. Extensions to the k-Means Algorithm for Clustering Large Data Sets with Categorical Values. *Data Mining and Knowledge Discovery* 1998;2(3):283-304. doi: 10.1023/A:1009769707641
8. Li C, Wu X, Cheng X, et al. Identification and analysis of vulnerable populations for malaria based on K-prototypes clustering. *Environmental Research* 2019;176:108568. doi: https://doi.org/10.1016/j.envres.2019.108568
9. Aldenderfer MS, Blashfield RK. Cluster Analysis. California: SAGE Publications Inc 1984.
10. Clatworthy J, Buick D, Hankins M, et al. The use and reporting of cluster analysis in health psychology: a review. *Br J Health Psychol* 2005;10(Pt 3):329-58. doi: 10.1348/135910705x25697 [published Online First: 2005/10/22]
11. Rousseeuw PJ. Silhouettes: A graphical aid to the interpretation and validation of cluster analysis. *Journal of Computational and Applied Mathematics* 1987;20:53-65. doi: https://doi.org/10.1016/0377-0427(87)90125-7
12. Yoon S, Goh H, Kwan YH, et al. Identifying optimal indicators and purposes of population segmentation through engagement of key stakeholders: a qualitative study. *Health Res Policy Syst* 2020;18(1):26. doi: 10.1186/s12961-019-0519-x [published Online First: 2020/02/23]
13. Vuik SI, Mayer E, Darzi A. A quantitative evidence base for population health: applying utilization-based cluster analysis to segment a patient population. *Popul Health Metr* 2016;14:44. doi: 10.1186/s12963-016-0115-z [published Online First: 2016/12/03]
14. Charlson M, Szatrowski TP, Peterson J, et al. Validation of a combined comorbidity index. *J Clin Epidemiol* 1994;47(11):1245-51. doi: 10.1016/0895-4356(94)90129-5 [published Online First: 1994/11/01]
15. Vuik SI, Mayer E, Darzi A. Enhancing risk stratification for use in integrated care: a cluster analysis of high-risk patients in a retrospective cohort study. *BMJ Open* 2016;6(12):e012903. doi: 10.1136/bmjopen-2016-012903
16. Everitt BS, Landau S, Leese M, et al. Cluster analysis. 5th ed. Chichester: John Wiley & Sons 2011.

For peer review only - http://bmjopen.bmj.com/site/about/guidelines.xhtml

Supplementary Material D: Comparison of the low, moderate and high risk groups

Age Group	Female			Male		
	% at high risk in this group	% at moderate risk in this group	% at low risk in this group	% at high risk in this group	% at moderate risk in this group	% at low risk in this group
0-4	0.3	43	56.8	0.3	42.7	57
5-9	0.3	5.8	93.9	0.4	7.3	92.3
10-14	0.4	8.2	91.4	0.4	10.1	89.6
15-19	0.4	9.7	89.8	0.4	9.4	90.2
20-24	0.4	11.6	88	0.4	8.4	91.2
25-29	0.6	15.8	83.6	0.5	9.4	90
30-34	0.9	18.8	80.3	0.5	9.8	89.6
35-39	1.2	19.4	79.5	0.8	12	87.2
40-44	1.6	20	78.4	1.2	15	83.7
45-49	2.4	23.6	74	1.7	19.4	78.9
50-54	3.3	27	69.8	2.5	23.7	73.8
55-59	4.3	30.8	64.8	3.9	29.7	66.4
60-64	6.1	35.8	58.1	5.6	36.9	57.5
65-69	7.6	92.3	0.1	7.9	92	0.1
70-74	10.3	89.5	0.1	11	88.8	0.1
75-79	10.5	89.4	0.2	13.3	86.4	0.3
80-84	12.2	87.5	0.3	14.5	85.1	0.4
85-89	10.8	88.5	0.6	14	85.2	0.8
90-94	8.3	90.4	1.3	11.7	86.6	1.7
95+	5.3	92.1	2.6	11	86.3	2.7

Table D.1: Percentage of high, moderate and low risk individuals by age group and sex.

For peer review only

Plots comparing the distribution of age and Charlson Score for low, moderate and high risk groups

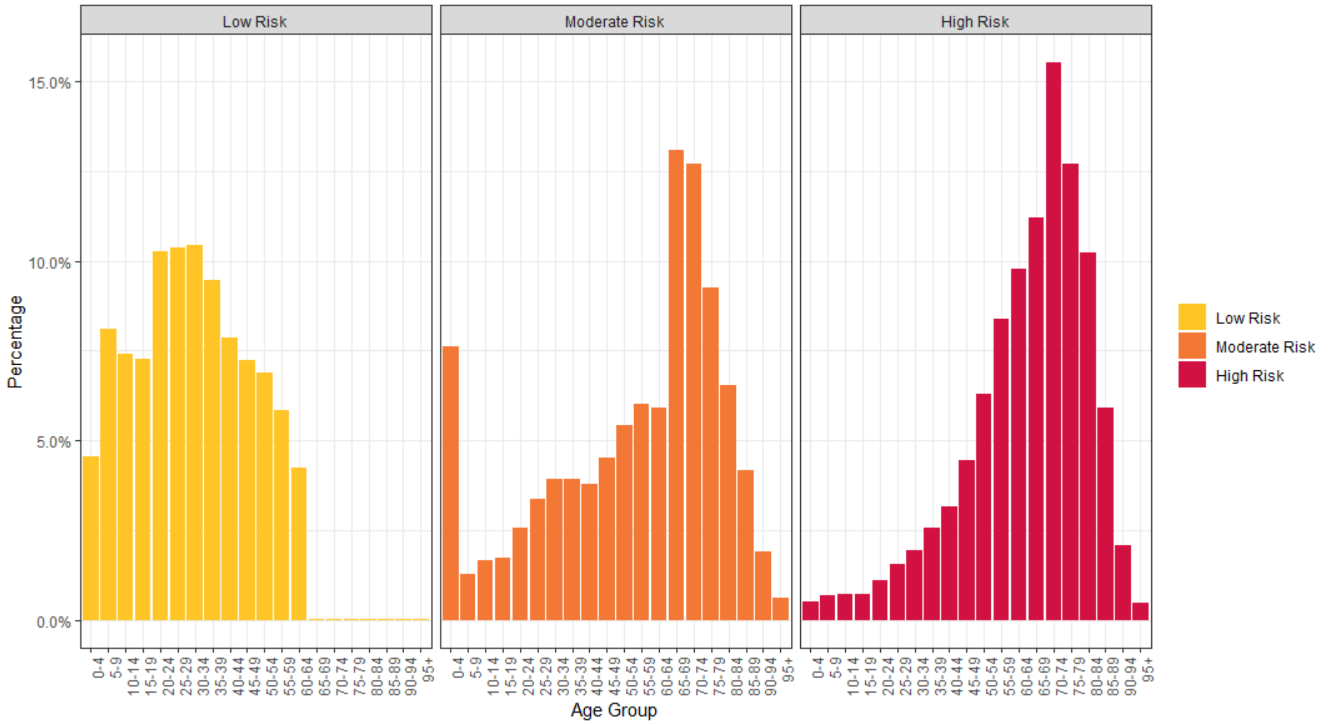

Figure D.1: Distribution of age groups by high, moderate and low risk groups.

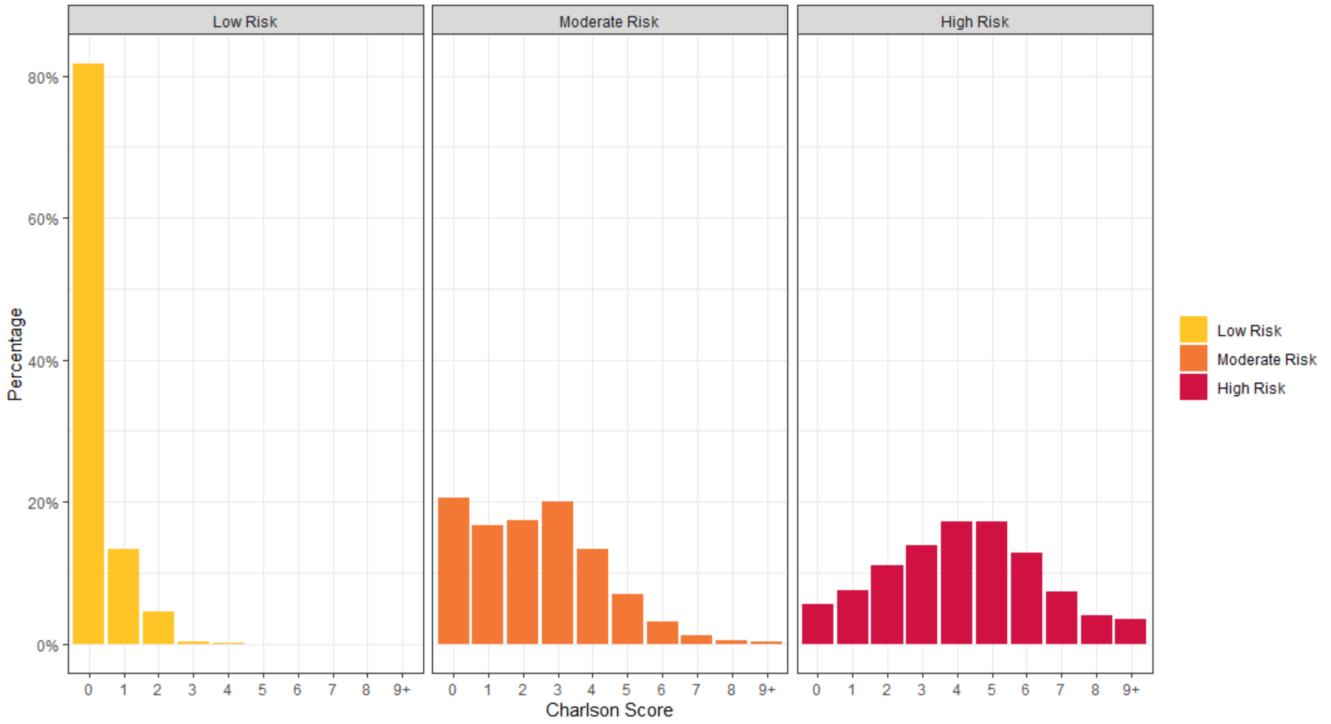

Figure D.2: Distribution of Charlson Score by high, moderate and low risk groups.

For peer review only - http://bmjopen.bmj.com/site/about/guidelines.xhtml

Supplementary Material A: RECORD Statement

The RECORD statement – checklist of items, extended from the STROBE statement, that should be reported in observational studies using routinely collected health data.

	Item No.	STROBE items	Location in manuscript where items are reported	RECORD items	Location in manuscript where items are reported
**Title and abstract**					
	1	(a) Indicate the study's design with a commonly used term in the title or the abstract (b) Provide in the abstract an informative and balanced summary of what was done and what was found	Page 1 in title and abstract	RECORD 1.1: The type of data used should be specified in the title or abstract. When possible, the name of the databases used should be included.<br><br>RECORD 1.2: If applicable, the geographic region and timeframe within which the study took place should be reported in the title or abstract.<br><br>RECORD 1.3: If linkage between databases was conducted for the study, this should be clearly stated in the title or abstract.	1.1 Page 1 in the abstract.<br><br>1.2 Page 1 in the abstract.<br><br>1.3 Page 1 – data were linked in a system wide dataset.
**Introduction**					
Background rationale	2	Explain the scientific background and rationale for the investigation being reported	Page 3 under Introduction		
Objectives	3	State specific objectives, including any prespecified hypotheses	Page 3 at the bottom of the page.		
**Methods**					
Study Design	4	Present key elements of study design early in the paper	Page 4 under 2.1 Data, application and setting.		
Setting	5	Describe the setting, locations, and relevant dates, including periods of recruitment, exposure, follow-up, and data collection	Page 4		
Participants	6	*(a) Cohort study* - Give the eligibility criteria, and the sources and methods of selection of participants. Describe methods of follow-up<br>*Case-control study* - Give the eligibility criteria, and the sources and methods of case ascertainment and control selection. Give the rationale for the choice of cases and controls<br>*Cross-sectional study* - Give the eligibility criteria, and the sources and methods of selection of participants	Cross-sectional study – page 4 gives eligibility criteria and sources/method of selection.	RECORD 6.1: The methods of study population selection (such as codes or algorithms used to identify subjects) should be listed in detail. If this is not possible, an explanation should be provided.<br><br>RECORD 6.2: Any validation studies of the codes or algorithms used to select the population should be referenced. If validation was conducted for this study and not published elsewhere, detailed methods and results should be provided.<br><br>RECORD 6.3: If the study involved linkage of databases, consider use of a flow diagram or other graphical display to demonstrate the data linkage process, including the number of individuals with linked data at each stage.	In this case all individuals within BNSSG registered to a contributing practice were included for analyses. Therefore codes to identify the overall study population were not used.<br><br>For the high-risk group population selection, all codes and criteria used are listed fully in Supplementary Material B and the methods described in section 2.2 (page 4).<br><br>Furthermore, the resulting list was compared with estimated expected numbers of high-risk individuals described in the Discussion under interpretation page 11.<br><br>The system-wide dataset at BNSSG CCG is already linked for analyses but a reference is included providing further information to the dataset

For peer review only

		(b) Cohort study - For matched studies, give matching criteria and number of exposed and unexposed<br>*Case-control study* - For matched studies, give matching criteria and the number of controls per case			(Reference 25) and linking through a pseudonymised ID (unique individual identifier) was mentioned on page 4.
Variables	7	Clearly define all outcomes, exposures, predictors, potential confounders, and effect modifiers. Give diagnostic criteria, if applicable.	All outcomes and variables are fully defined including the use of algorithms and coding lists in Supplementary Material B for the high-risk criteria and Supplementary Material C for all clustering and non-clustering variables. Reference to the Supplementary material included on page 5 of the methods.	RECORD 7.1: A complete list of codes and algorithms used to classify exposures, outcomes, confounders, and effect modifiers should be provided. If these cannot be reported, an explanation should be provided.	All outcomes and variables are fully defined including the use of algorithms and coding lists in Supplementary Material B for the high-risk criteria and Supplementary Material C for all clustering and non-clustering variables.
Data sources/ measurement	8	For each variable of interest, give sources of data and details of methods of assessment (measurement). Describe comparability of assessment methods if there is more than one group	Table C.3 in Supplementary Material C.		
Bias	9	Describe any efforts to address potential sources of bias	To minimise misclassification and information bias, high risk criteria and coding lists were subject to double clinical review (page 5 Methods). The discussion page 10,11 discusses potential information bias as a limitation of using routine datasets.		
Study size	10	Explain how the study size was arrived at	Page 4 and 5		
Quantitative variables	11	Explain how quantitative variables were handled in the analyses. If applicable, describe which groupings were chosen, and why	How the variables were operationalised for analyses is detailed in Table C.3 Supplementary Material C.		
Statistical methods	12	(a) Describe all statistical methods, including those used to control for confounding<br>(b) Describe any methods used to examine subgroups and interactions<br>(c) Explain how missing data were addressed	a) Section 2.3 page 5. Further details on cluster analysis and selection of the number of clusters is included in Supplementary Material C.<br><br>b) Subgroups were investigated in Table		

For peer review only

		(d) *Cohort study* - If applicable, explain how loss to follow-up was addressed<br>*Case-control study* - If applicable, explain how matching of cases and controls was addressed<br>*Cross-sectional study* - If applicable, describe analytical methods taking account of sampling strategy<br>(e) Describe any sensitivity analyses	1. Methods described on page 5.<br><br>c) Page 5<br><br>d) NA. The full BNSSG cohort from March 2019 was taken for analyses.<br><br>e) Sensitivity analyses in terms of the clustering solution chosen for segmentation is fully described in Supplementary Material C. Also described more briefly in the methods page 5.		
Data access and cleaning methods		..		RECORD 12.1: Authors should describe the extent to which the investigators had access to the database population used to create the study population.<br><br>RECORD 12.2: Authors should provide information on the data cleaning methods used in the study.	The full BNSSG database population for the SWD were used for analyses. This is included on page 4.<br><br>How variables were coded and operationalised is documented in Supplementary Material B and C and page 4 describes how a unique identifier is used to link data between the tables in the system wide dataset.
Linkage		..		RECORD 12.3: State whether the study included person-level, institutional-level, or other data linkage across two or more databases. The methods of linkage and methods of linkage quality evaluation should be provided.	Page 4 under section 2.1.
**Results**					
Participants	13	(a) Report the numbers of individuals at each stage of the study (*e.g.*, numbers potentially eligible, examined for eligibility, confirmed eligible, included in the study, completing follow-up, and analysed)<br>(b) Give reasons for non-participation at each stage.<br>(c) Consider use of a flow diagram	Page 6 for descriptive analyses.<br>Page 8 for cluster analysis.	RECORD 13.1: Describe in detail the selection of the persons included in the study (*i.e.,* study population selection) including filtering based on data quality, data availability and linkage. The selection of included persons can be described in the text and/or by means of the study flow diagram.	Page 6 for descriptive analyses.<br>Page 8 for cluster analysis.
Descriptive data	14	(a) Give characteristics of study participants (*e.g.*, demographic, clinical, social) and information on exposures and potential confounders<br>(b) Indicate the number of participants with missing data for	Page 6 for descriptive analyses.<br>Page 8 for cluster analysis.<br><br>Number of participants with missing data page 8.		

For peer review only

		each variable of interest<br>(c) *Cohort study* - summarise follow-up time (*e.g.*, average and total amount)			
Outcome data	15	*Cohort study* - Report numbers of outcome events or summary measures over time<br>*Case-control study* - Report numbers in each exposure category, or summary measures of exposure<br>*Cross-sectional study* - Report numbers of outcome events or summary measures	*Cross-sectional study* – Page 6, Table 1, Figure 1 and 2 report a comparative analysis of the low, moderate and high-risk groups with regard to demographic and socioeconomic information, historical healthcare utilisation, and burden of comorbidity. Figure 3 shows the geographical distribution of high risk individuals.		
Main results	16	(a) Give unadjusted estimates and, if applicable, confounder-adjusted estimates and their precision (e.g., 95% confidence interval). Make clear which confounders were adjusted for and why they were included<br>(b) Report category boundaries when continuous variables were categorized<br>(c) If relevant, consider translating estimates of relative risk into absolute risk for a meaningful time period	a) Main descriptive analyses reported on pages 6-8. Cluster analysis results reported on page 8-10.<br><br>b) Continuous variables kept as continuous and results reported as mean, median with interquartile range or proportion. Tables 1 and 2. Supplementary Material C discusses how variables are operationalised.<br><br>c) Not applicable.		
Other analyses	17	Report other analyses done—e.g., analyses of subgroups and interactions, and sensitivity analyses	Results are presented for low, moderate and high risk subgroups and for the segmentation analysis for clusters 1-6. Pages 6-8 for descriptive analyses and page 8-10 for cluster analyses. Sensitivity analyses in terms of the clustering solution were performed and documented in Supplementary Material C.		
**Discussion**					
Key results	18	Summarise key results with reference to study objectives	Page 10.		
Limitations	19	Discuss limitations of the study, taking into account sources of potential bias or imprecision. Discuss both direction and magnitude of any potential bias	Page 10-11.	RECORD 19.1: Discuss the implications of using data that were not created or collected to answer the specific research question(s). Include discussion of misclassification bias, unmeasured confounding, missing data, and changing eligibility over time, as they	Page 10-12 (i.e. misclassification bias and limitations relating to the data).

For peer review only

				pertain to the study being reported.	
Interpretation	20	Give a cautious overall interpretation of results considering objectives, limitations, multiplicity of analyses, results from similar studies, and other relevant evidence	Page 11-12.		
Generalisability	21	Discuss the generalisability (external validity) of the study results	Page 10-12.		
**Other Information**					
Funding	22	Give the source of funding and the role of the funders for the present study and, if applicable, for the original study on which the present article is based	Page 13.		
Accessibility of protocol, raw data, and programming code		..		RECORD 22.1: Authors should provide information on how to access any supplemental information such as the study protocol, raw data, or programming code.	Page 13. Technical appendix and coding lists available in online supplementary files. Local data used for the study are not publicly available.

*Reference: Benchimol EI, Smeeth L, Guttmann A, Harron K, Moher D, Petersen I, Sørensen HT, von Elm E, Langan SM, the RECORD Working Committee. The REporting of studies Conducted using Observational Routinely-collected health Data (RECORD) Statement. *PLoS Medicine* 2015; in press.

*Checklist is protected under Creative Commons Attribution (CC BY) license.
